# ST8SIA6-AS1 contributes to hepatocellular carcinoma progression by targeting miR-142-3p/HMGA1 axis

**DOI:** 10.1038/s41598-022-26643-8

**Published:** 2023-01-12

**Authors:** Tianhang Feng, Yutong Yao, Le Luo, Haibo Zou, Guangming Xiang, Lingling Wei, Qinyan Yang, Ying Shi, Xiaolun Huang, Chunyou Lai

**Affiliations:** 1 Department of Hepatobiliary and Pancreatic Surgery Center, Cell Transplantation Center, Sichuan Academy of Medical Sciences, Sichuan Provincial People’s Hospital, School of Medicine, University of Electronic Science and Technology of China, No.32, West Section 1, Yihuan Road, Qingyang District, Chengdu, 610000 Sichuan China; 2 Department of Hepatobiliary and Pancreatic Surgery Center, Cell Transplantation Center, Sichuan Academy of Medical Sciences, Sichuan Provincial People’s Hospital, School of Medicine, University of Electronic Science and Technology of China, No.4, Section 2, Jianshe North Road, Chengdu, 610000 Sichuan China

**Keywords:** Cancer, Cell biology, Molecular biology

## Abstract

Hepatocellular carcinoma (LIHC) accounts for 90% of all liver cancers and is a serious health concern worldwide. Long noncoding RNAs (lncRNAs) have been observed to sponge microRNAs (miRNAs) and participate in the biological processes of LIHC. This study aimed to evaluate the role of the ST8SIA6-AS1-miR-142-3p-HMGA1 axis in regulating LIHC progression. RT-qPCR and western blotting were performed to determine the levels of ST8SIA6-AS1, miR-142-3p, and HMGA1 in LIHC. The relationship between ST8SIA6-AS1, miR-142-3p, and HMGA1 was assessed using luciferase assay. The role of the ST8SIA6-AS1-miR-142-3p-HMGA1 axis was evaluated in vitro using LIHC cells. Expression of ST8SIA6-AS1 and HMGA1 was significantly upregulated, whereas that of miR-142-3p was markedly lowered in LIHC specimens and cells. ST8SIA6-AS1 accelerated cell growth, invasion, and migration and suppressed apoptosis in LIHC. Notably, ST8SIA6-AS1 inhibited HMGA1 expression by sponging miR-142-3p in LIHC cells. In conclusion, sponging of miR-142-3p by ST8SIA6-AS1 accelerated the growth of cells while preventing cell apoptosis in LIHC cells, and the inhibitory effect of miR-142-3p was abrogated by elevating HMGA1 expression. The ST8SIA6-AS1-miR-142-3p-HMGA1 axis represents a potential target for the treatment of patients with LIHC.

## Introduction

Liver cancer, which accounts for 8.2% of cancer-related deaths worldwide, is the sixth most common cancer type. Hepatocellular carcinoma (LIHC) accounts for 90% of all liver cancers and is a serious problem worldwide^1,2^. Owing to unsatisfactory curative therapeutic modalities, the 5-year survival rate of LIHC is less than 20%, with high metastatic and recurrence rates^3^. Therefore, exploring the molecular mechanisms of LIHC is necessary to design more effective targeted therapies for patients with LIHC.

Long noncoding RNAs (lncRNAs), with a length of almost 200 nucleotides, play key roles in gene expression regulation through epigenetic modulation, chromatin remodeling, and sponging effect^4^. Notably, lncRNAs that sponge microRNAs (miRNAs) have oncogenic or tumor suppressor functions in cancer occurrence and development^5,6^. LncRNAs can sponge miRNAs in various cancers, including cervical cancer, laryngeal squamous cell carcinoma, and lung cancer, by affecting cancer cell growth and metastasis^7–9^. Studies have reported that lncRNA ST8SIA6-AS1 functions as an oncogene in some cancers^10–12^. Accumulated evidence shows that abnormally increased expression of ST8SIA6-AS1 in LIHC promotes the proliferation, migration, and invasion of cancer cells, resistance to apoptosis, and malignant phenotype of hepatocellular carcinoma^13,14^. Thus, ST8SIA6-AS1 has been shown to be related to LIHC development.

MicroRNAs (miRNAs) are ncRNAs approximately 19–22 nucleotides long that regulate cell growth and differentiation in multiple malignancies, including nasopharyngeal carcinoma, osteosarcoma, and breast cancer^15–17^. Several studies have shown that lncRNAs act as miRNA sponges. LncRNA TUG1 sponges miR-384 and promotes the epithelial-mesenchymal transition (EMT) of nasopharyngeal carcinoma cells by repressing miR-384 expression^15^. In addition, MALAT1 inhibits miR-34a and suppresses osteosarcoma cell growth via the miR‑34a/cyclin D1 axis^16^. miR-142-3p with low expression in hepatocellular carcinoma exerts an antitumor role in hepatocellular carcinoma cells by inhibiting metastasis, aerobic glycolysis, and cell proliferation^18–21^. LncRNAs, such as TUG1, MALAT1, CR594175, and lnc712, can enhance cell growth and EMT of cancer cells in the liver by sponging miR-142-3p^22–25^. However, whether miR-142-3p regulation by ST8SIA6-AS1 affects LIHC progression remains unclear.

The high mobility group AT-hook 1 (HMGA1) gene encodes a chromatin-associated protein that regulates gene transcription and metastatic progression in cancer cells^26,27^. HMGA1 is aberrantly expressed in various cancers and represents a potential target for cancer treatment^28–30^. A study reported HMGA1 as a driver of stem cells and inflammatory pathways of cells, and during lymphoid tumorigenesis, HMGA1 affects cell cycle progression genes^31^. Furthermore, sHMGA1 is highly expressed in hepatocellular carcinoma, correlating with poor prognosis. It also enhances the growth and migration of hepatocellular carcinoma cells, promoting the pathogenesis of hepatocellular carcinoma^32–34^. These results suggest that HMGA1 is of substantial significance in the study of hepatocellular carcinoma. However, few studies have examined the role of the ST8SIA6-AS1/miR-142-3p/HMGA1 axis in LIHC development. This study aimed to evaluate the role of the ST8SIA6-AS1-miR-142-3p-HMGA1 axis in LIHC cells with the goal of identifying a new ideal for LIHC treatment.

## Materials and methods

### Clinical samples, cell culture, and transfection

Thirty-five LIHC specimens and matched paracancerous normal tissues were obtained from patients diagnosed with LIHC at our hospital. All patients provided informed consent, and the ethics committee of the hospital approved our study. Patient characteristics are shown in Table 1. LIHC cell lines (Hep3B, HCCLM3, Huh7, and SNU-182) and normal liver epithelial cells (THLE2) were provided by American Type Culture Collection (ATCC, USA). Hep3B, HCCLM3, and Huh7 cells were cultured in DMEM (Gibco, USA). SNU-182 and THLE2 cells were cultured in medium RPMI-1640 (Gibco). All cells were incubated with 10% FBS at 37 °C and 5% CO_2_. siRNA targeting lncRNA ST8SIA6-AS1 or HMGA1 (si-lnc or si-HMGA1), miR-142-3p mimics/inhibitor (anti-miR), and their negative controls (NC) (RiboBio Co. Ltd., China) were used to treat HCCLM3 and Huh7 cells using Lipofectamine 2000 (Invitrogen, USA). After 48 h, further functional analyses were performed.

**Table 1:** Baseline characteristics of 35 HCC patients.

Categories	Cases (Total n = 35)	Percentage (%)
**Gender**		
Male	14	40.0
Female	21	60.0
**Age (years)**		
≥ 55	18	51.4
< 55	17	48.6
**Tumor size (cm)**		
≥ 5	11	31.4
< 5	24	68.6
**TNM stage**		
I + II	20	57.1
III + IV	15	42.9
**Lymph node metastasis**		
Positive	19	54.3
Negative	16	45.7
**Liver cirrhosis**		
Presence	13	37.1
Absence	22	62.9
**Serum AFP (ng/mL)**		
≤ 20	18	51.4
> 20	17	48.6

### RT-qPCR and nuclear and cytosolic fraction

LncRNAS and mRNA were isolated from tissues and cells using TRIzol reagent (Thermo, USA). Next, cDNA synthesis was performed using a PrimeScript First Strand cDNA Synthesis Kit (Takara, Japan). RT-qPCR of lncRNAs and mRNA was performed using SYBR Green (Thermo Fisher Scientific, USA). For miRNAs, an miRcute miRNA Extraction Kit (China) was used to isolate miRNAs, and cDNA was synthesized using an miRcute miRNA First Strand cDNA Synthesis Kit (Tiangen, China). RT-qPCR of miRNAs was performed using an miRcute enhanced miRNA Fluorescence Quantitative Detection Kit (Tiangen, China).

Nuclear and cytosolic fractions were separated using a PARIS Kit (AM1921, Life Technologies, USA). RT-qPCR was performed to assess the levels of ST8SIA6-AS1 in the nucleus and cytoplasm.

The relative expression of lncRNA and mRNA was normalized to that of GAPDH, and U6 was used to normalize miRNA expression. The obtained data were subjected to 2^−ΔΔCt^ analysis. The sequences of primer are listed in Table 2.

**Table 2:** The sequences of the primers in this study.

Primer	Sequences
miR-142-3p	Forward: 5′-GCCGCGTGTAGTGTTTCCTA-3′
	Reverse: 5′-TATGGTTGTTCTCGTCTCTGTGTC-3′
miR-145-5p	Forward: 5′-GUCCAGUUUUCCCAGGAAUCCCU-3′
	Reverse: 5′-AGGGAUUCCUGGGAAAACUGGAC -3′
miR-338-3p	Forward: 5′-TGCGGTCCAGCATCAGTGAT-3′
	Reverse: 5′- CCAGTGCAGGGT CCGAGGT-3′
miR-5195-3p	Forward: 5′-TAGCAGACTCTTATGATG-3′
	Reverse: 5′-TGGTGGAGTCGTCGTG-3′
ST8SIA6-AS1	Forward: 5′-TCCTGATTCAGTGGCATGGT-3′
	Reverse: 5′-AGGGTTTCTTCGGTCGTCAT-3′
HMGA1	Forward: 5′-GCTGGTAGGGAGTCAGAAGG-3′
	Reverse: 5′-TTGGTTTCCTTCCTGGAGTT-3′
GAPDH	Forward: 5′-CTGGGCTACACTGAGCACC-3′
	Reverse: 5′-AAGTGGTCGTTGAGGGCAATG-3′
U6	Forward: 5′-CAAATTCGTGAAGCGTTCCA-3′
	Reverse: 5′-GTGCAGGGTCCGAGGT-3′

### Cell counting kit-8 (CCK8) assay

Cell viability was determined using a CCK8 assay kit (Cat#: K1018; APExBIO, China). In brief, HCCLM3 and Huh7 cells (5 × 10^3^ cells/well) were cultured in 96-well plates, and the old medium was removed from the wells at 0, 24, 48, 72, and 96 h. Then, 90 µL fresh medium and 10 µL CCK8 buffer was added to all wells for 2 h incubation. Absorbance values were measured at 450 nm using a multimode plate reader.

### EdU assay

Cell proliferation was analyzed using the BeyoClick EdU Cell Proliferation Kit with Alexa Fluor 594 (C0078S; Beyotime, China). Approximately 1 × 10^4^ cells were cultured in 6-well plates. After reaching 80% confluency, the cells were incubated with EdU for 2 h. Next, cell fixing was performed using 4% of paraformaldehyde for 20 min at 25 °C, followed by permeabilizing with 20% of Triton X-100 at 25 °C for 30 min. Subsequently, the cells were washed twice with PBS and mixed with labeled-azide (prepared with Click Reaction buffer) at 25 °C for 30 min without light, followed by nuclear staining with DAPI for 10 min. Finally, stained cells were washed twice and photographed using a confocal microscope (Olympus, Japan).

### Detection of apoptosis using flow cytometry

An Annexin V-FITC detection kit (BD, USA) was used to assess apoptosis in HCCLM3 and Huh7 cells. Transfected HCCLM3 and Huh7 cells at a concentration of 1 × 10^5^ were collected and suspended in binding buffer, and 5 µL FITC and 5 µL PI were added to the suspension and incubated for 30 min in the dark. Next, the cells apoptosis was detected by flow cytometric analysis (BD Biosciences).

### Wound healing assay

Transfected HCCLM3 and Huh7 cells were seeded in 6-well plates at a concentration of 1 × 10^6^ cells/well and cultured to 80% confluence. Next, a circle of cells was cleared using a 10 μL sterile pipette, and the wells were washed twice to remove the non-adherent cells. Images of wound recovery at 0 and 24 h were obtained using a light microscope, and the diameter of circular clearing was recorded.

### Transwell assay

A Transwell chamber perfused with Matrigel (Corning, USA) was used for cell invasion detection. HCCLM3 and Huh7 cells (8 × 10^4^ cells) were cultured in the upper portion of the serum-free medium, while the bottom chamber contained medium supplemented with 10% FBS. After 48 h of incubation, the cells on the membranes were fixed with methanol at 25 °C for 20 min, followed by incubation with 0.1% of crystal violet for 15 min. Finally, images of invaded cells were obtained using a light microscope.

### Luciferase assay

The psiCHECK2 vectors containing wild-type (WT)/mutant (Mut) ST8SIA6-AS1 and WT/Mut HMGA1 3′-UTR were obtained from GeneChem (China). miR-142-3p mimic/mimic-NC and WT/MUT vectors were transfected into Huh7 and HCCLM3 cells using Lipofectamine 2000 (Invitrogen). The luciferase activities of firefly and Renilla were observed using a Dual Luciferase Reporter Gene Assay Kit (Cat#: RG027, Beyotime, China) after 72 h.

### Radioimmunoprecipitation (RIP) assay

Interactions between ST8SIA6-AS1 and the miRNAs were determined using a RIP RNA-Binding Protein Immunoprecipitation Kit (Sigma, USA). After lysing the cells, cell lysates were incubated with miR-142-3p mimic, miR-651-5p mimic, miR-145-5p mimic, miR-338-3p mimic, and miR-5195-3p mimic, and IgG or Ago2 for 4 h. Subsequently, magnetic beads were added to the tube and incubated at 4 °C overnight. Finally, the expression levels of ST8SIA6-AS1 were determined using RT-qPCR.

### RNA pull-down assay

HCCLM3 and Huh7 cells were transfected with biotin-labeled-miR-142-3p or NC (Thermo Fisher Scientific, USA). After 48 h, the streptavidin beads (88817, Thermo, USA) were added to the lysis buffer at 4 °C for overnight incubation. The next day, an RNA purification kit (DP412, Tiangen, China) was used to purify the eluent, and the expression of HMGA1 was determined using RT-qPCR.

### Western blotting analysis

Cell lysates were obtained from transfected HCCLM3 and Huh7 cells using RIPA buffer (20-188, Sigma, USA), and 20 µg of protein was separated by 10% SDS-PAGE and transferred to PVDF membranes. Using 5% non-fat milk for 3 h, the membranes were blocked and then incubated overnight with primary antibodies against HMGA1 (Cat#: ab129153), PCNA (Cat#: ab92552), Bax (Cat#: ab216494), Bcl-2 (Cat#: ab32124) (Abcam (UK)), and GAPDH (Cat#: 5174, CST, USA) diluted 1:1000 at 4 °C. Next day, the membranes were incubated with secondary antibody (Cat#: ab6721, Abcam) diluted 1:10,000 at 25 °C for 2 h. Proteins were visualized using ECL reagents (P0018S; Beyotime, China).

### Xenograft tumor model

C57BL/6 mice were purchased from the Academy of Military Medical Sciences (Beijing, China). All mice used were 6–8 weeks old and weighed 18–22 g. They were housed in a specific pathogen-free animal facility. The care and treatment of mice were performed according to the guidelines for laboratory animal care and approved by the Animal Ethics Committee of our hospital. HCCLM3 cells were injected subcutaneously into the right axilla of mice, with five animals in each group, after stable transfection with NC or ST8SIA6-AS1 shRNA. The length and breadth of tumors were measured weekly. The mice were killed by cervical dislocation 5 weeks after injection, and the tumors were separated and weighed. We then plotted the time-growth curves of the tumors based on the results. This animal experiment was conducted in accordance with the ARRIVE guidelines and was authorized by the Ethics Committee of Sichuan Academy of Medical Sciences, Sichuan Provincial People’s Hospital, School of Medicine, University of Electronic Science and Technology of China.

### Statistical analysis

Data were analyzed using GraphPad Prism version 8.0 software (GraphPad Prism, USA) with a paired Student’s t test for comparison of two groups and one-way analysis of variance for multiple groups. Experimental data are shown as the mean ± standard deviation (SD) of triplicate experiments. Statistical significance was set at P < 0.05.

### Ethical approval and consent to participate

The present study was approved by the Ethical Committee of the Sichuan Provincial People's Hospital (Chengdu, China). The clinical tissue samples processing is with strict compliance of the ethical standards of Declaration of Helsinki. Signed informed consent were taken from all patients.

This animal experiment was conducted in accordance with the ARRIVE guidelines and was authorized by the Ethics Committee of Sichuan Academy of Medical Sciences, Sichuan Provincial People's Hospital, School of Medicine, University of Electronic Science and Technology of China.

## Results

### Genes of interest in LIHC were ST8SIA6-AS1, miR-142-3p, and HMGA1

**Figure 1:**
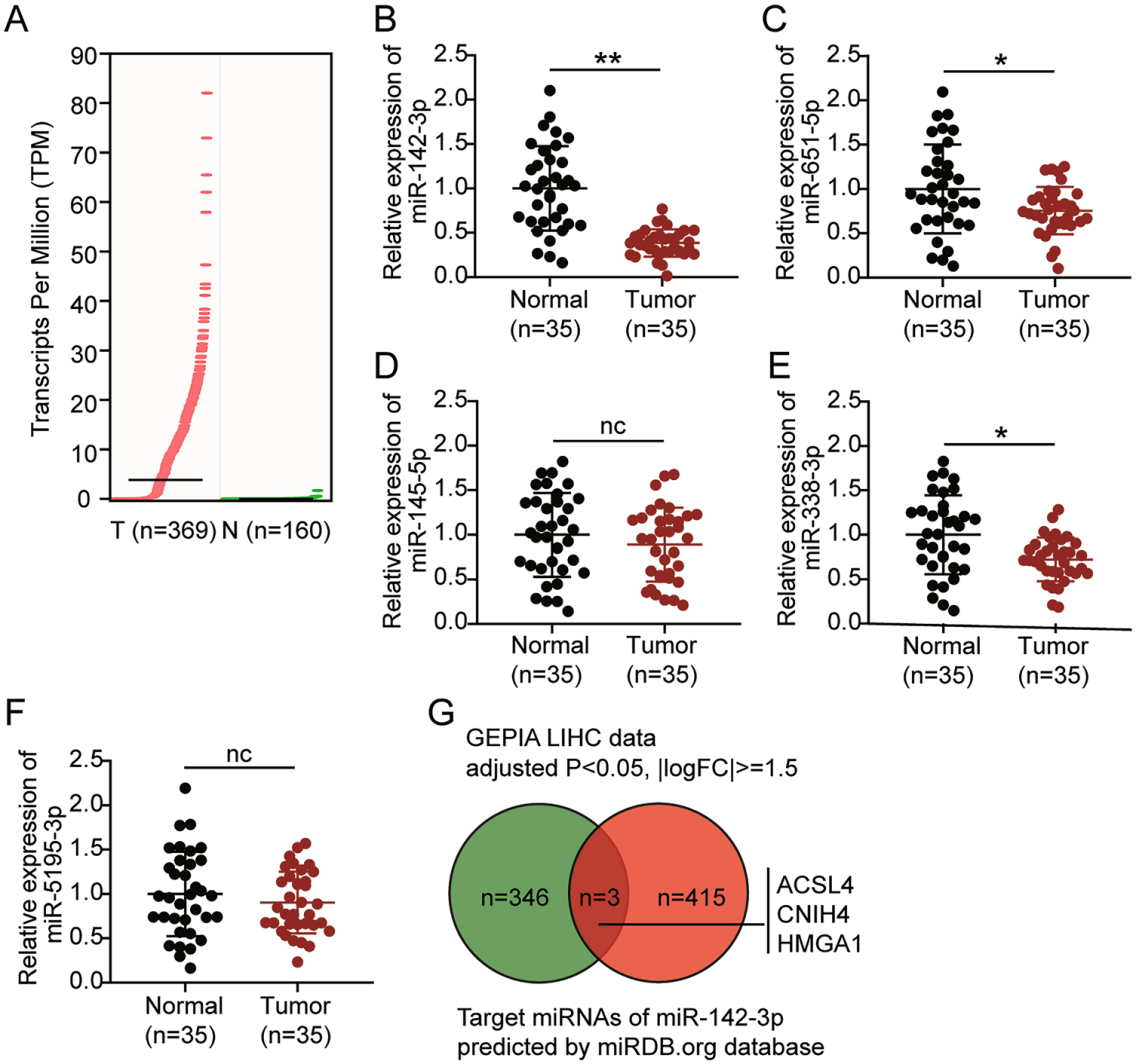
The expression of miR-142-3p and HMGA1 was aberrant in LIHC. (**A**) The expression of ST8SIA6-AS1 in LIHC tissues and normal hepatocellular tissues. Data from GEPIA database. (**B**–**F**) The expression of miR-142-3p (**B**), miR-651-5p (**C**), miR-145-5p (**D**), miR-338-3p (**E**), miR-5195-3p (**F**) in LIHC and normal liver tissues. *P < 0.05, **P < 0.001. nc: not significant. (**G**) The intersection of miRDB-predicted targets of miR-142-3p as well as the DE (differentially expressed) genes from GEPIA LIHC data (logFC ≥ 1.5 and adjusted P < 0.05,). LIHC: hepatocellular carcinoma.

The Gene Expression Profiling Interactive Analysis (GEPIA) database showed that ST8SIA6-AS1 was upregulated in LIHC (logFC = 2.266, Fig. 1A), and several studies have shown that ST8SIA6-AS1 is a driver of LIHC progression^12–14^. Next, by using the StarBase algorithm, five downstream target miRNAs of ST8SIA6-AS1 were predicted. We examined the expression of these five miRNAs in the collected samples and found that miR-142-3p was the most significantly downregulated miRNA in LIHC (Fig. 1B–F). The RIP assay results also confirmed that ST8SIA6-AS1 interacted with miR-142-3p in HCCLM3 and Huh7 cells (Supplementary Fig. 1A–E). Therefore, miR-142-3p was used in further experiments. By intersecting the candidate target genes of miR-142-3p, as predicted by miRDBA, and the significantly differentially expressed genes from the GEPIA LIHC database, we identified three genes common to both datasets: HMGA1, ACSL4, and CNIH4 (Fig. 1G). HMGA1 has been reported to be a potential tumor promoter in LIHC^32,33^; however, its interaction with miRNAs in a ceRNA role remains unclear.

### ST8SIA6-AS1 had high expression in LIHC cells

**Figure 2:**
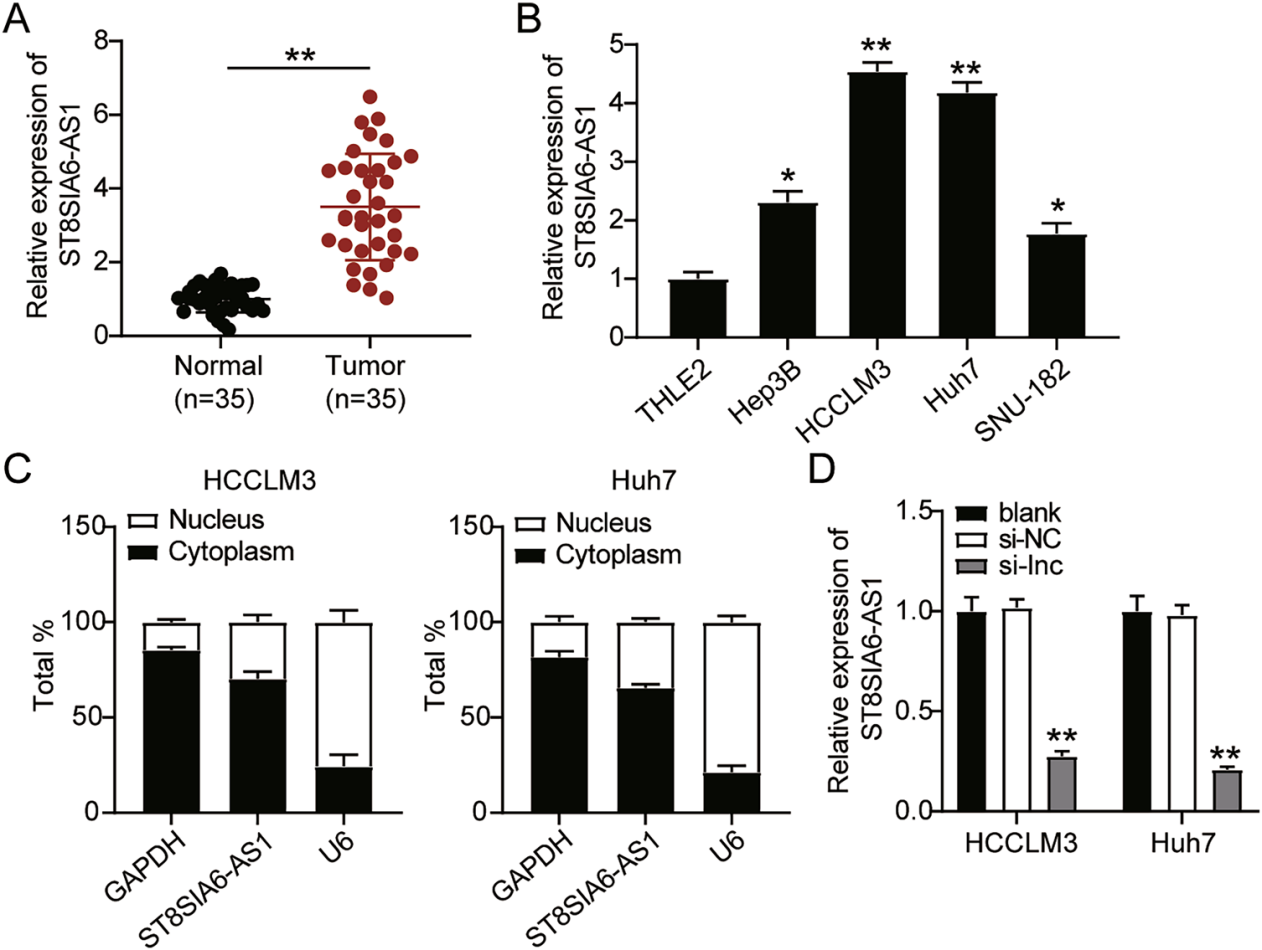
ST8SIA6-AS1 upregulation in LIHC cells. (**A**) ST8SIA6-AS1 upregulated in LIHC tissues. **P < 0.001. (**B**) Upregulation of ST8SIA6-AS1 expression in LIHC cell lines (Hep3B, HCCLM3, Huh7, and SNU-182) comparison with normal liver epithelial cells THLE2. *P < 0.05, **P < 0.001 compared with THLE2. (**C**) RT-qPCR detection of GAPDH, and U6, and ST8SIA6-AS1, in nucleus and cell cytosol. (**D**) ST8SIA6-AS1 expression in HCCLM3 and in Huh7 cells treated with si-ST8SIA6-AS1. *P < 0.05, **P < 0.001 compared with blank. NC: negative control; blank: blank control; si-ST8SIA6-AS1: siRNA-ST8SIA6-AS1.

First, we analyzed ST8SIA6-AS1 expression in LIHC tissues and found that ST8SIA6-AS1 expression was increased in LIHC tissues (Fig. 2A). In this study, the levels of ST8SIA6-AS1 were classified as low or high based on the median value. Statistical analysis revealed that high levels of ST8SIA6-AS1 were positively correlated with serum AFP, lymph node metastasis, and TNM stage; however, no significant correlation was between ST8SIA6-AS1 and other clinical features, such as age, sex, tumor size, and liver cirrhosis (Supplementary Table 1). LIHC cell lines (Hep3B, HCCLM3, Huh7, and SNU-182) also showed markedly elevated expression of ST8SIA6-AS1 compared to normal liver epithelial cells, THLE2 (Fig. 2B). HCCLM3 and Huh7 cells were selected for further studies because they showed the highest ST8SIA6-AS1 levels among the LIHC cell lines analyzed. Additionally, ST8SIA6-AS1 was mainly present in the cytoplasm of HCCLM3 and Huh7 cells (Fig. 2C). We successfully transfected HCCLM3 and Huh7 cells with siRNA-ST8SIA6-AS1 (Fig. 2D).

### ST8SIA6-AS1 enhanced the progression of LIHC

**Figure 3:**
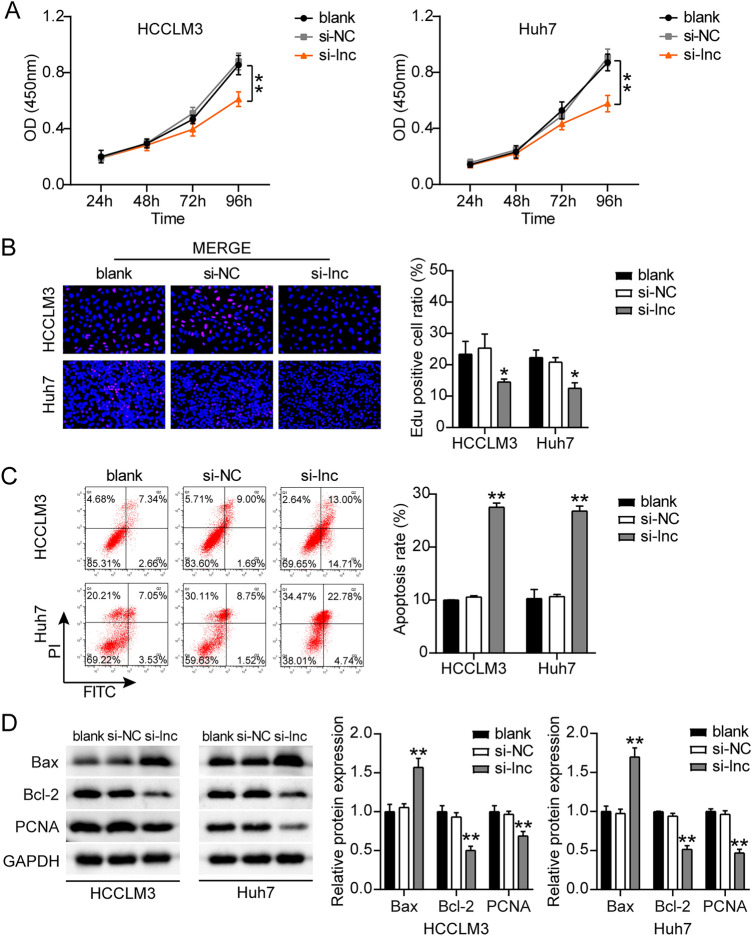
ST8SIA6-AS1 facilitated cell growth of LIHC cells. (**A**) Cell viability determined by CCK8 assay. (**B**) Detection of cell growth by the EdU assay. (**C**) The FITC apoptosis detection kit was used to assess cell apoptosis. (**D**) The expression of Bax, Bcl-2, and PCNA proteins was measured using a western blot test. *P < 0.05, **P < 0.001 compared with blank. NC: negative control; blank: blank control; si-ST8SIA6-AS is siRNA-ST8SIA6-AS1, and the statistical value is P < 0.001** at which we test hypothesis.

**Figure 4:**
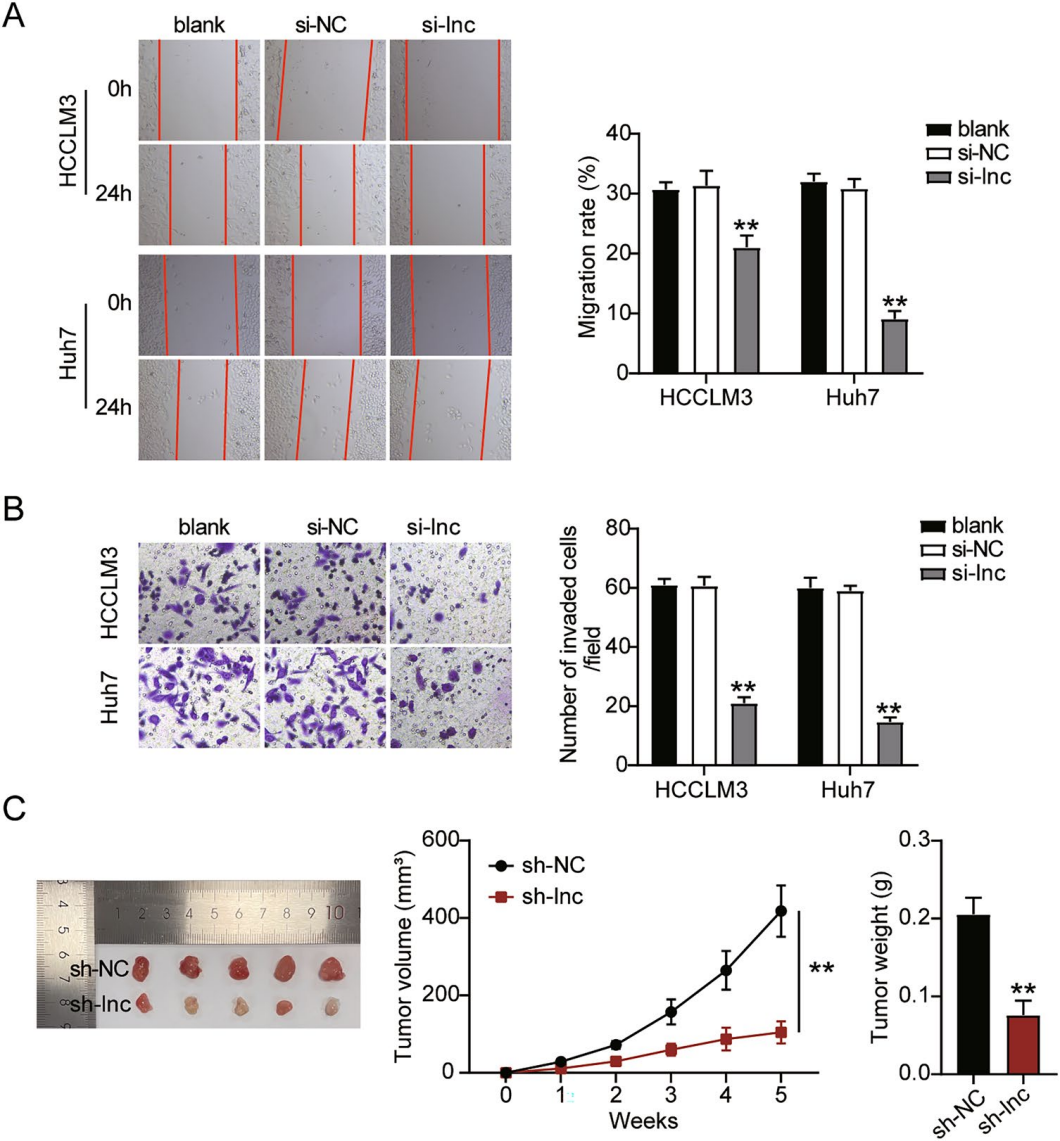
ST8SIA6-AS1 elevated cell migration, invasion and the tumor growth of LIHC cells. (**A**) Cell migration ability detection through wound healing assay. **P < 0.001 compared with blank. NC: negative control; blank: blank control; si-ST8SIA6-AS1: siRNA-ST8SIA6-AS1. (**B**) The transwell assay is used to detect cell invasion. **P < 0.001 compared with blank. NC: negative control; blank: blank control; si-ST8SIA6-AS1: siRNA-ST8SIA6-AS1. (**C**). The tumor volume curve and tumor weight were observed in xenograft Balb/c nude mice model with HCCLM3 cells transfected with sh-ST8SIA6-AS1 in vivo. **P < 0.001 compared with sh-NC. NC: negative control; sh-ST8SIA6-AS1: shRNA-ST8SIA6-AS1.

To determine the role of ST8SIA6-AS1 in LIHC progression, we first assessed the viability, proliferation, and apoptosis of LIHC cells transfected with siRNA-ST8SIA6-AS1. The si-lnc groups showed significantly lower cell viability as compared to non-transfected cell lines (Fig. 3A). The EdU assay showed that cell proliferation in cells treated with si-lnc was lower than that in the control groups in both cell lines (Fig. 3B). Furthermore, the silencing of ST8SIA6-AS1 resulted in a 2.5-fold increase in apoptosis in both cell lines (Fig. 3C). Bax protein expression was consistently increased in cells treated with si-lnc, according to western blot analysis. In contrast, the levels of Bcl-2 and PCNA protein in cells treated with si-lnc were reduced in the HCCLM3 and Huh7 cell lines, suggesting a negative role for si-lnc in proliferation and a positive role in apoptosis (Fig. 3D). The cell migration assay revealed that cell migration was inhibited when HCCLM3 and Huh7 cells were transfected with si-lnc (Fig. 4A). For cell invasion, the si-lnc group showed an approximately 60% decrease in invasion in the HCCLM3 and Huh7 cell lines (Fig. 4B). Additionally, we constructed a xenograft nude mouse model using HCCLM3 cells to confirm the effect of silencing HCCLM3 in vivo. The results of the tumor growth curve showed that ST8SIA6-AS1 knockdown markedly suppressed tumor growth, with a tumor inhibition rate of 65% at 5 weeks after cell inoculation. Similarly, tumor weight was also decreased (Fig. 4C). Thus, the downregulation of ST8SIA6-AS1 hindered LIHC progression.

### ST8SIA6-AS1 sponged miR-142-3p in LIHC cells

**Figure 5:**
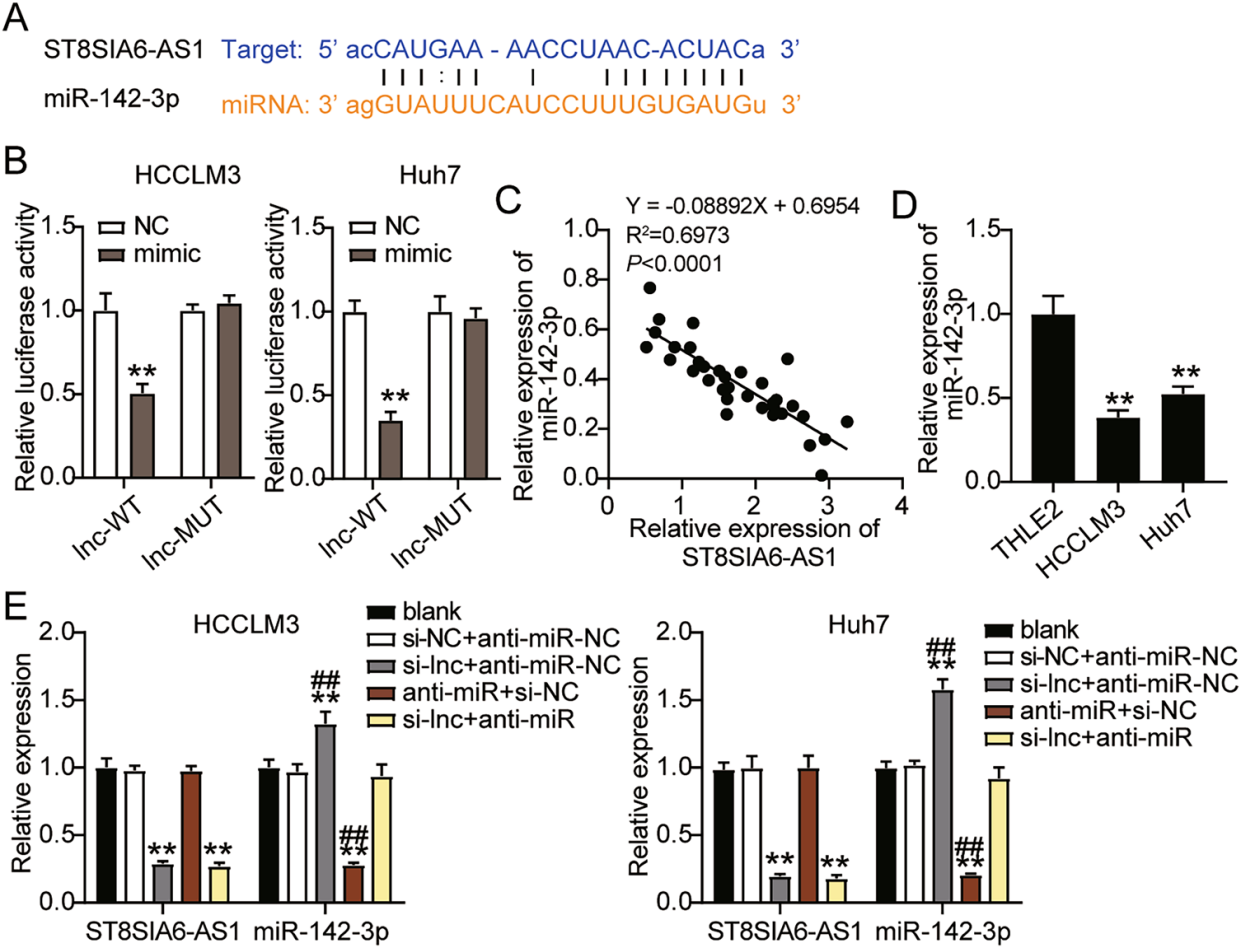
ST8SIA6-AS1 sponged miR-142-3p in LIHC cells. (**A**) StarBase analysis prediction for binding sites between miR-142-3p and ST8SIA6-AS1. (**B**) Performance of Dual luciferase assessment in HCCLM3 and Huh7 cells. **P < 0.001 compared with NC. (**C**) The expression of miR-142-3p and ST8SIA6 AS1 are negatively correlated. (**D**) In LIHC cells (HCCLM3 and Huh7 cells), the expression of miR-142-3p was downregulate compared to normal cells (THLE2). **P < 0.001 compared with THLE2. (**E**) In HCCLM3 and Huh7 cells, miR-142-3p and ST8SIA6-AS1 expression was measured by RT-qPCR. ^##^P < 0.001 compared with si-lnc + anti-miR; **P < 0.001 compared with blank. WT: wild-type; Si-lnc: SiRNA-ST8SIA6-AS1, MUT: Mutant; anti-miR: miR-142-3p inhibitor, NC: negative control, and blank: blank control, Si-lnc + anti-miR (SiRNA-ST8SIA6-AS1 + miR-142-3p inhibitor).

Binding site sequences of ST8SIA6-AS1 on miR-142-3p were obtained using StarBase (Fig. 5A). The luciferase assay revealed that the luciferase activity of cells co-transfected with miR-142-3p mimics and WT vector was markedly downregulated by nearly 50% compared with that in the NC group, whereas cells transfected with psiCHECK2 ST8SIA6-AS1-Mut showed no difference in either the HCCLM3 or Huh7 cell lines (Fig. 5B). miR-142-3p expression and ST8SIA6-AS1 in LIHC specimens showed a negative correlation (Fig. 5C). Statistical analysis revealed that high levels of miR-142-3p were negatively correlated with serum AFP, lymph node metastasis, and TNM stage; however, no significant correlation was observed between miR-142-3p and other clinical features, such as age, sex, tumor size, and liver cirrhosis (Supplementary Table 1). Furthermore, miR-142-3p expression in HCCLM3 and Huh7 cells was lower than that in THLE2 cells (Fig. 5D). After transfection of si-ST8SIA6-AS1 and the anti-miR into HCCLM3 and Huh7 cells in the si-ST8SIA6-AS1 group, miR-142-3p levels were increased 1.5-fold, but that of ST8SIA6-AS1 were decreased by 60%. The anti-miR treatment presented a 70% reduction in miR-142-3p levels, whereas ST8SIA6-AS1 levels remained the same as in blank control cells. In addition, the si-lnc + anti-miR group showed a 70% decrease in ST8SIA6-AS1 levels, whereas miR-142-3p levels remained the same as in the blank control group (Fig. 5E).

### ST8SIA6-AS1 mediated the malignancy of LIHC cells by sponging miR-142-3p

**Figure 6:**
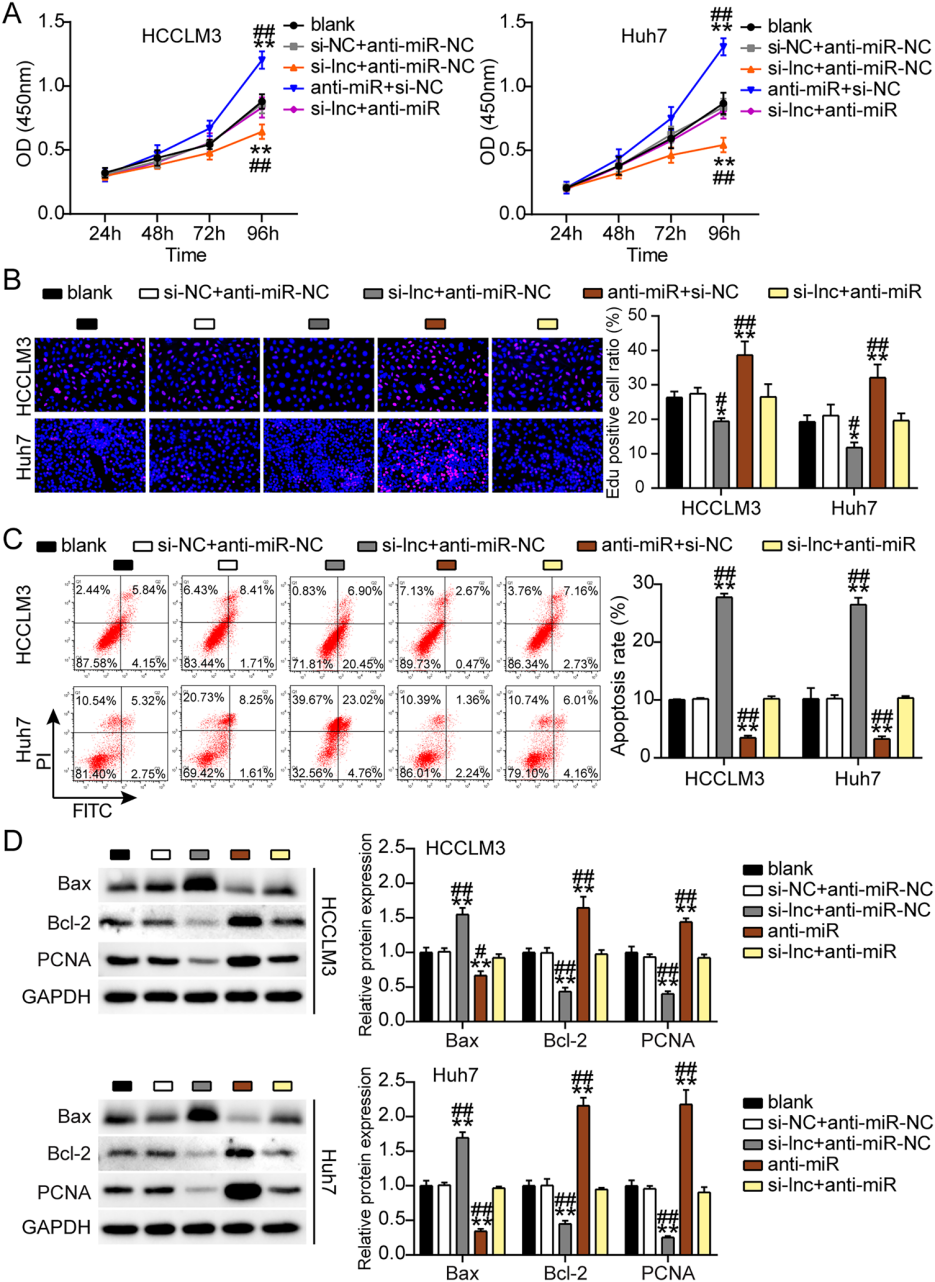
ST8SIA6-AS1 sponging miR-142-3p facilitated the development of LIHC cells. (**A**) Cell viability determination with CCK8 assay. (**B**) Proliferation of cell was determined by EdU assay. (**C**) Apoptosis of cell determination by FITC apoptosis detection kit. (**D**) Bax, Bcl-2 and PCNA protein expression were detected using western bloting assay. ^#^P < 0.05, ^##^P < 0.001 compared with si-lnc + anti-miR; *P < 0.05, **P < 0.001 compared with blank. NC: negative control; blank: blank control; anti-miR: miR-142-3p inhibitor; Si-lnc: SiRNA-ST8SIA6-AS1; Si-lnc + anti-miR: SiRNA-ST8SIA6-AS1 + miR-142-3p inhibitor.

**Figure 7:**
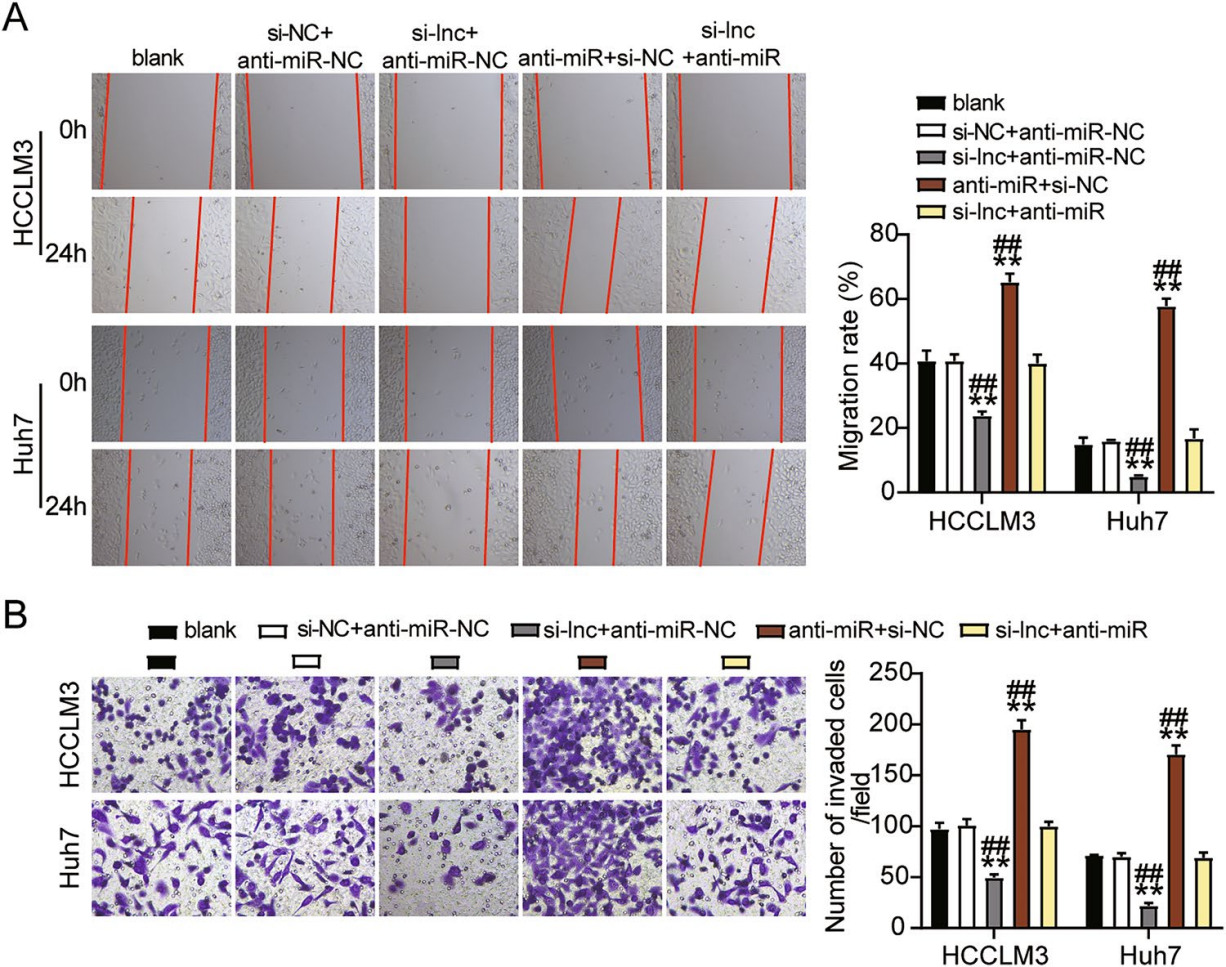
ST8SIA6-AS1 sponging miR-142-3p enhanced the cell migration and invasion of LIHC cells. (**A**) An assay for wound healing used to determine cell migration ability. (**B**) Cell invasion was detection using transwell assay. ^##^P < 0.001 compared with si-lnc + anti-miR; **P < 0.001 compared with blank. NC: negative control; blank: blank control; anti-miR: miR-142-3p inhibitor; Si-lnc: SiRNA-ST8SIA6-AS1; Si-lnc + anti-miR: SiRNA-ST8SIA6-AS1 + miR-142-3p inhibitor.

Next, we found that treatment with the anti-miR promoted cell viability, whereas si-lnc abrogated this effect in the HCCLM3 and Huh7 cells (Fig. 6A). In addition, the anti-miR enhanced cell proliferation, whereas this effect was limited by co-transfection with si-lnc (Fig. 6B). Conversely, the anti-miR group showed over 50% inhibition of apoptosis, but si-lnc transfection inhibited this effect (Fig. 6C). Furthermore, compared with the blank control groups, miR-142-3p downregulation inhibited the protein level of Bax and increased Bcl-2 and PCNA protein expression; however, these effects were partially reversed by si-lnc (Fig. 6D). Furthermore, the anti-miR groups showed markedly increased cell migration (1.4-fold higher in HCCLM3 cells and threefold higher in Huh7 cells, respectively), whereas this effect was abrogated in the si-lnc group in both cell types (Fig. 7A). In addition, the anti-miR groups showed almost a twofold and threefold increase in cell invasion in HCCLM-3 and Huh7 cells, respectively, whereas si-lnc abrogated this effect in both cell lines (Fig. 7B).

### miR-142-3p targeted HMGA1 and inhibited HMGA1 expression in LIHC cells

**Figure 8:**
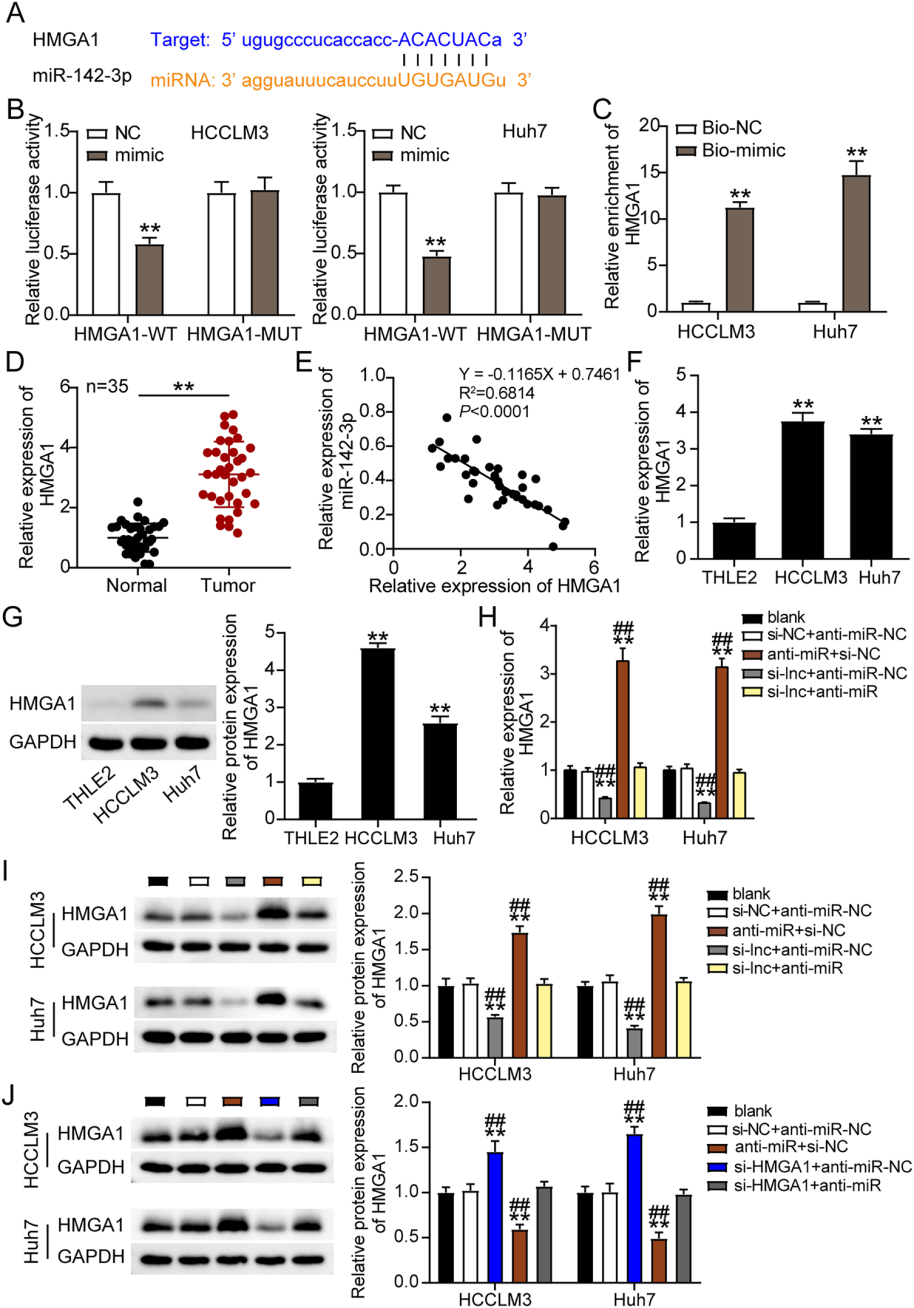
MiR-142-3p repressed the level of HMGA1 in LIHC cells. (**A**) StarBase prediction of binding sequences between HMGA1 and miR-142-3p. (**B**) Dual luciferase assay was measured in HCCLM3 and Huh7 cells. **P < 0.001 comparison with NC. (**C**) The enrichment of miR-142-3p, ST8SIA6-AS1 and HMGA1 was determined by RT-qPCR. **P < 0.001 comparison with Bio-NC. (**D**) RT-qPCR used for determination of HMGA1 expression in LIHC tissues. **P < 0.001. (**E**) In LIHC tissues, there is a negative connection between miR-142-3p and HMGA1. (**F**) HMGA1 expression was measured using RT-qPCR in LIHC cells (HCCLM3 and Huh7 cells) and normal cells (THLE2). **P < 0.001 comparison with THLE2. (**G**) HMGA1 expression was measured using western blot in LIHC cells (HCCLM3 and Huh7 cells) and normal cells (THLE2). **P < 0.001 comparison with THLE2. (**H**) HMGA1 mRNA expression measurement in transfected Huh7 and HCCLM3 cells. (**I**) Expression of the HMGA1 protein in transfected Huh7 cells and HCCLM3 was estimated. **P < 0.001 comparison with blank; ^##^P < 0.001 comparison with si-lnc + anti-miR. NC: negative control; Si-lnc: SiRNA-ST8SIA6-AS1; anti-miR: miR-142-3p inhibitor; Si-lnc + anti-miR: SiRNA-ST8SIA6-AS1 + miR-142-3p inhibitor. (**J**) Measurement of HMGA1 expression in HCCLM3 and Huh7 cells was detected by western blot. ^##^P < 0.001 compared with si-HMGA1 + anti-miR; **P < 0.001 compared with blank. NC: negative control; blank: blank control, MUT: Mutant; WT: wild-type; Si-HMGA1 + anti-miR: SiRNA-HMGA1 + miR-142-3p inhibitor, and anti-miR: miR-142-3p inhibitor; Si-HMGA1: SiRNA-HMGA1.

The sequences of the HMGA1 and miR-142-3p binding sites are shown in Fig. 8A. In contrast to the psiCHECK2 HMGA1 3′UTR Mut plasmid-treated cells, cells co-treated with the WT vector and mimics showed an approximately 50% decrease in luciferase activity (Fig. 8B). In LIHC cells, an RNA pull-down test revealed an association between miR-142-3p and HMGA1 (Fig. 8C). Furthermore, HMGA1 expression increased threefold in LIHC tissues (Fig. 8D), and miR-142-3p expression and HMGA1 in LIHC samples showed a negative correlation (Fig. 8E). In addition, statistical analysis revealed that high levels of HMGA1 were positively correlated with serum AFP, lymph node metastasis, and TNM stage; however, no significant correlation was between HMGA1 and other clinical features, such as age, sex, tumor size, and liver cirrhosis (Supplementary Table 1). The mRNA and protein level of HMGA1 in HCCLM3 and Huh7 cells were significantly enhanced (Fig. 8F,G). Next, we examined the effects of ST8SIA6-AS1 and miR-142-3p on the expression of HMGA1. The mRNA and protein levels of HMGA1 in HCCLM3 and Huh7 cells decreased after ST8SIA6-AS1 knockdown and increased when miR-142-3p was downregulated (Fig. 8H,I). Next, we transfected si-HMGA1 and anti-miR into HCCLM3 cells and Huh7 cells. The si-HMGA1 group showed a decrease in HMGA1 protein levels, and the anti-miR group showed an increase in HMGA1 protein levels in HCCLM3 and Huh7 cells. The si-HMGA1 + anti-miR group, by contrast, expressed the same level of HMGA1 as the blank control cells (Fig. 8J).

### miR-142-3p affected LIHC cell malignancy by inhibiting HMGA1

**Figure 9:**
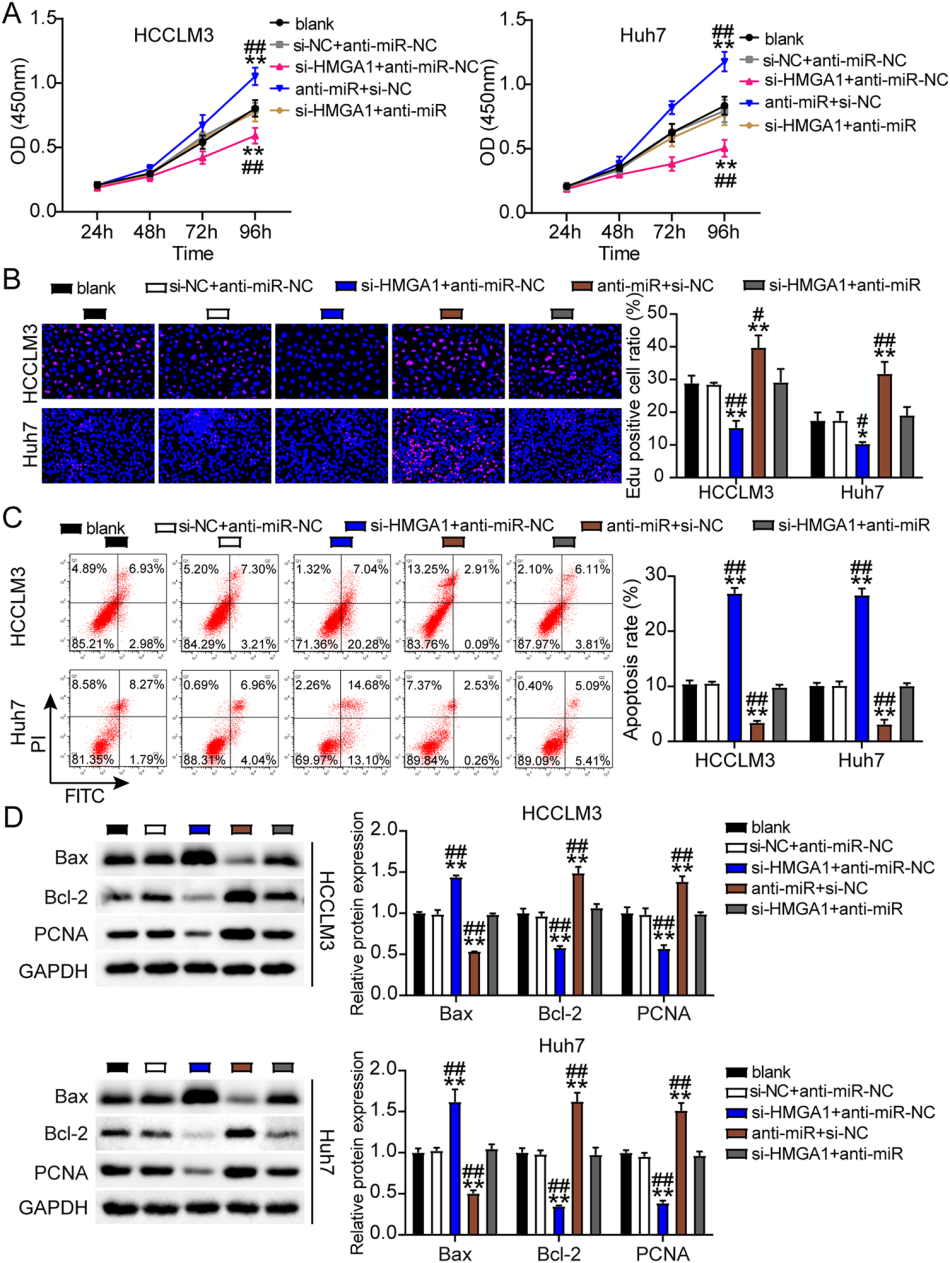
MiR-142-3p attenuated cell proliferation and elevated cell apoptosis by repressing HMGA1. (**A**) Cell viability determination using CCK8 assay. (**B**) Cell proliferation was determined by EdU assay. (**C**) Cell apoptosis was determined by FITC apoptosis detection kit. (**D**) Bax, Bcl-2 and PCNA protein expression were determined in HCCLM3 and Huh7 cells. ^#^P < 0.05, ^##^P < 0.001 compared with si-HMGA1 + anti-miR; *P < 0.05, **P < 0.001 compared with blank. anti-miR: miR-142-3p inhibitor; Si-HMGA1 + anti-miR: SiRNA-HMGA1 + miR-142-3p inhibitor. NC: negative control; Si-HMGA1: SiRNA-HMGA1.

**Figure 10:**
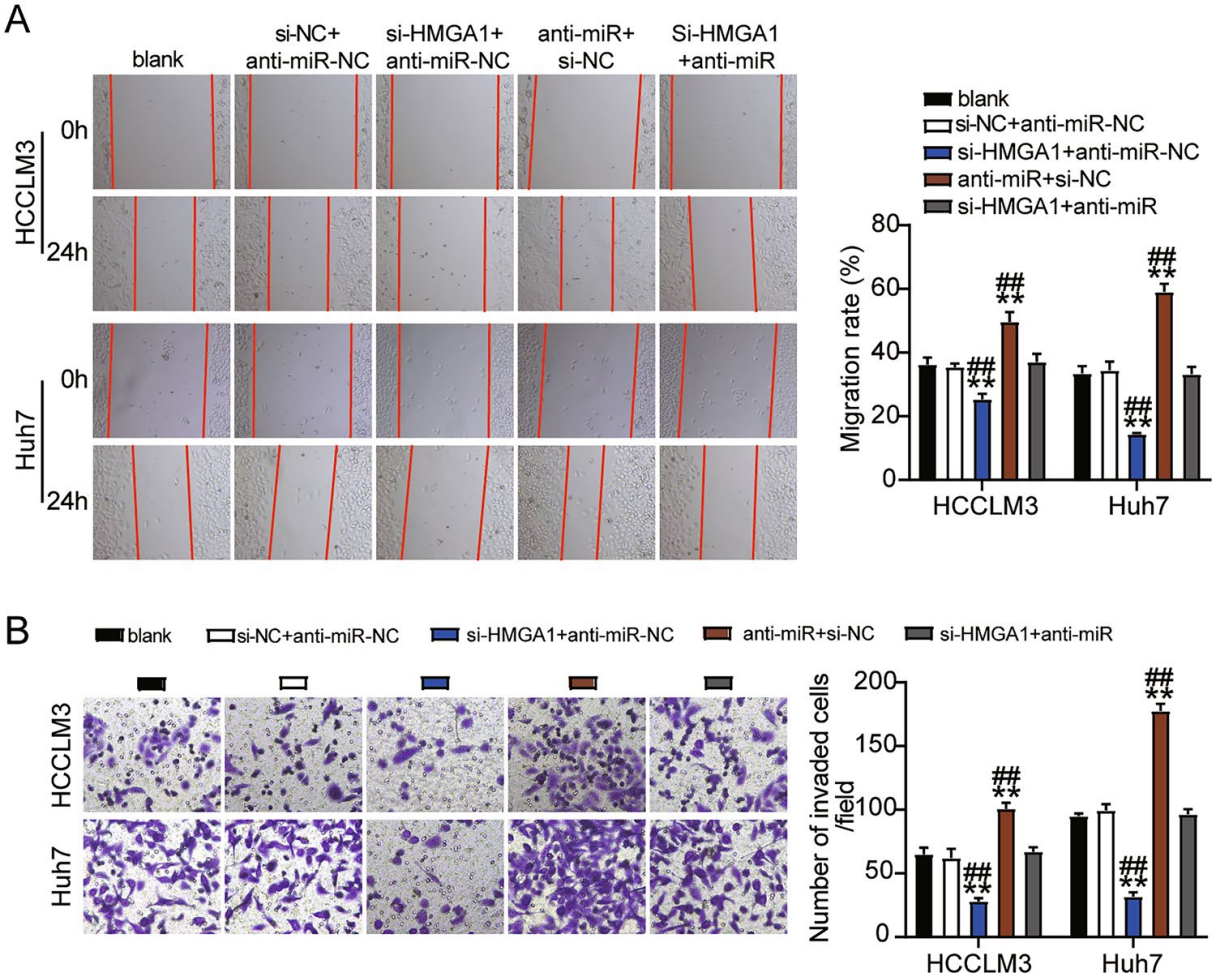
MiR-142-3p suppressed the migration and invasion in LIHC by repressing HMGA1. (**A**) Cell migration ability detection by wound healing assay. (**B**) the cell invasion detection using transwell assay. ^##^P < 0.001 compared with si-HMGA1 + anti-miR; **P < 0.001 compared with blank. anti-miR: miR-142-3p inhibitor; Si-HMGA1 + anti-miR: SiRNA-HMGA1 + miR-142-3p inhibitor, blank: blank control, NC: negative control; Si-HMGA: SiRNA-HMGA1.

The si-HMGA1 group showed decreased cell viability, whereas si-HMGA1 + anti-miR treatment abrogated this effect in HCCLM3 and Huh7 cells (Fig. 9A). Similar to cell viability, cell proliferation was inhibited in the si-HMGA1 group, whereas the si-HMGA1 + anti-miR treatment abrogated this effect in Huh7 and HCCLM3 cells (Fig. 9B). In addition, in HCCLM3 and Huh7 cells, the si-HMGA1 groups showed a 2.5-fold increase in apoptosis, respectively, whereas si-HMGA1 + anti-miR treatment abrogated this effect (Fig. 9C). In addition, in HCCLM3 and Huh7 cells, knockdown of HMGA1 promoted Bax protein expression and inhibited the protein levels of Bcl-2 and PCNA, whereas co-transfection with si-HMGA1 and miR-142-3p inhibitor reversed this effect (Fig. 9D). Finally, cell migration was significantly downregulated in si-HMGA1 transfected HCCLM3 and Huh7 cells by 25% and 50%, respectively, whereas si-HMGA1 + anti-miR treatment abrogated this effect (Fig. 10A). Furthermore, the si-HMGA1 groups showed marked suppression of cell invasion by 50% and 60% in the HCCLM3 and Huh7 cell lines, respectively, whereas si-HMGA1 + anti-miR treatment abrogated this effect (Fig. 10B).

## Discussion

This study showed the upregulation of ST8SIA6-AS1 and HMGA1 and the downregulation of miR-142-3p in LIHC tissues and cell lines. Sponging of miR-142-3p by ST8SIA6-AS1 increased cell growth and inhibited apoptosis in LIHC cells. Furthermore, the suppressive role of miR-142-3p was inhibited by raising the expression of HMGA1.

Studies have analyzed the biological effects of lncRNA-mediated miRNA sponging, which plays multiple roles in tumor occurrence and progression^8,35,36^. Evidence revealed that SNHG4 promoted cell growth and EMT by repressing miR-204-5p in gastric cancer^35^. Liu et al. revealed that lncRNA RASSF8-AS1 attenuated cell growth by inhibiting miR-664b-3p and promoting TLE1 expression in laryngeal squamous cell carcinoma^8^. Xiao et al. showed that lncRNA MALAT1 enhanced oral squamous cell carcinoma cell growth by decreasing miR-101 and upregulating EZH2^36^. LncRNAs also participate in the initiation and development of HCC^37–39^. Lnc-ATG9B-4 prevents cell growth by upregulating CDK5 in hepatocellular carcinoma cells^37^. Moreover, lncRNA HAGLROS accelerated cell growth and autophagy but reduced cell apoptosis by downregulating miR-5095 and enhancing ATG12 expression in hepatocellular carcinoma^38^. ST8SIA6-AS1 sponged two miRNAs, miR-5195-3p and miR-4656, to elevate HOXB6 expression and promote HDAC11 expression, respectively, which promoted cell proliferation but hampered apoptosis^13,14^. Similarly, in our study, the expression of ST8SIA6-AS1 was increased in LIHC tissues and cells, and the silencing of ST8SIA6-AS1 inhibited cell growth while enhancing cell apoptosis in HCCLM3 and Huh7 cells. Moreover, ST8SIA6-AS1 abrogated the effects of miR-142-3p on LIHC cells.

The role of miR-142-3p in hepatocellular carcinoma has been demonstrated^18,19,21^. For instance, miR-142-3p repressed aerobic glycolysis and cell growth by repressing LDHA in hepatocellular carcinoma^19^. Furthermore, miR-142-3p attenuated metastasis by inhibiting HMGB1 expression in hepatocellular carcinoma cells^18^. Notably, various lncRNAs have been shown to inhibit miR-142-3p expression and carcinogenesis^23,25,40^. For instance, MALAT1 upregulated SMAD5 expression by reducing miR-142-3p expression in hepatocellular cancer, which facilitates cell growth and EMT^23^. Cui et al. reported that lncRNA Lnc712 accelerated cell growth by repressing miR-142-3p and promoting Bach-1 in hepatocellular carcinoma^25^. Consistent with these results, our study’s results showed that miR-142-3p expression was markedly repressed in LIHC tissues and negatively correlated with ST8SIA6-AS1 and HMGA1 levels in LIHC tissues. Furthermore, downregulation of miR-142-3p facilitated cell growth and reduced cell apoptosis in LIHC. Thus, sponging of miR-142-3p by ST8SIA6-AS1 accelerates LIHC progression.

Previous studies have highlighted the role of HMGA1 in different cancers^29,30^. Chen et al. demonstrated that HMGA1 was downregulated by miR-26 in lung cancer cells. miR-26 inhibited HMGA1 expression and further suppressed TNF-alpha/NF-kappaB signaling^29^. Additionally, HMGA1 upregulation promoted colorectal cancer cell growth by boosting Wnt/β-catenin signaling^30^. HMGA1 overexpression was associated with tumorigenesis in hepatocellular carcinoma^33,34,41^. Liu et al. demonstrated that the upregulation of HMGA1 promoted hepatocellular carcinoma cell growth and that the ILK/Akt/GSK3beta signaling pathway was involved in this progression^33^. Andreozzi et al. identified that high expression of HMGA1 increased cell growth in hepatocellular carcinoma^41^. Additionally, Teng et al. found that HMGA1 transcriptional activity was activated by KIFC1, promoting cell growth in vitro and in vivo^34^. Similarly, we proved that silencing HMGA1 suppressed the growth and enhanced apoptosis of LIHC cells. Moreover, miR-142-3p suppressed LIHC progression by inhibiting HMGA1 expression.

TNF-α initiates a signaling cascade to activate the transcription factor NF-κB, forming the basis for various physiological and pathological processes^42^. Sustained activation of NF-κB has been linked to several aspects of oncogenesis, such as cancer cell proliferation promotion, the inhibition of apoptosis in drug resistance, and an increase in tumor angiogenesis and metastasis^43^. miR-142-3p was shown to inhibit NF-κB signaling in periodontitis^44^ and regulate the TNF-α pathway to affect chronic rhinosinusitis with nasal polyposis^45^. In addition, Chen et al. found that HMGA1 promoted the development of lung cancer cells by activating the TNF-α/NF-κB signaling pathway^29^. These results suggest that the TNF-α/NF-κB signaling pathway is affected by miR-142-3p/HMGA1. However, the relationship between ST8SIA6-AS1 and TNF-α/NF-κB signaling remains unclear. Therefore, in further research, we plan to determine whether the ST8SIA6-AS1/miR-142-3p/HMGA1 axis affects the proliferation, apoptosis, and invasion of LIHC cells by regulating the TNF-α/NF-κB pathway.

## Conclusion

Our findings reveal that ST8SIA6-AS1 accelerates cell growth while reducing cell apoptosis via the miR-142-3p/HMGA1 in LIHC. Thus, they indicate a potential effective strategy based on targeting the ST8SIA6-AS1/miR-142-3p/HMGA1 axis for the treatment of patients with LIHC.

## Supplementary Information

Supplementary Legends.

Supplementary Figure 1.

Supplementary Table 1.

Supplementary Information 4.

Supplementary Information 5.

Supplementary Information 6.

## Data Availability

The datasets used during this study are available on request to the corresponding author <laichunyou2010@163.com>.
