# Supplementary material for: ST8SIA6-AS1 contributes to hepatocellular carcinoma progression by targeting miR-142-3p/HMGA1 axis
Source: Sci Rep. 2023 Jan 12;13:650. doi: 10.1038/s41598-022-26643-8 (PMC9837176; doi:10.1038/s41598-022-26643-8)
Supplement: Supplementary file 1 — Supplementary Legends. [file 41598_2022_26643_MOESM1_ESM.docx]

**Supplementary Figure 1. The enrichment of ST8SIA6-AS1 on miRNA.**

The enrichment of ST8SIA6-AS1 by treating with miR-142-3p mimic (A) miR-651-5p mimic (B) miR-145-5p mimic (C) miR-338-3p mimic (D) miR-5195-3p mimic (E) in HCCLM3 and in Huh7 cells through RIP assay. ^*^P< 0.05, ^**^P< 0.001.
