# Supplementary figures and images for: ST8SIA6-AS1 contributes to hepatocellular carcinoma progression by targeting miR-142-3p/HMGA1 axis
Source: Sci Rep. 2023 Jan 12;13:650. doi: 10.1038/s41598-022-26643-8 (PMC9837176; doi:10.1038/s41598-022-26643-8)

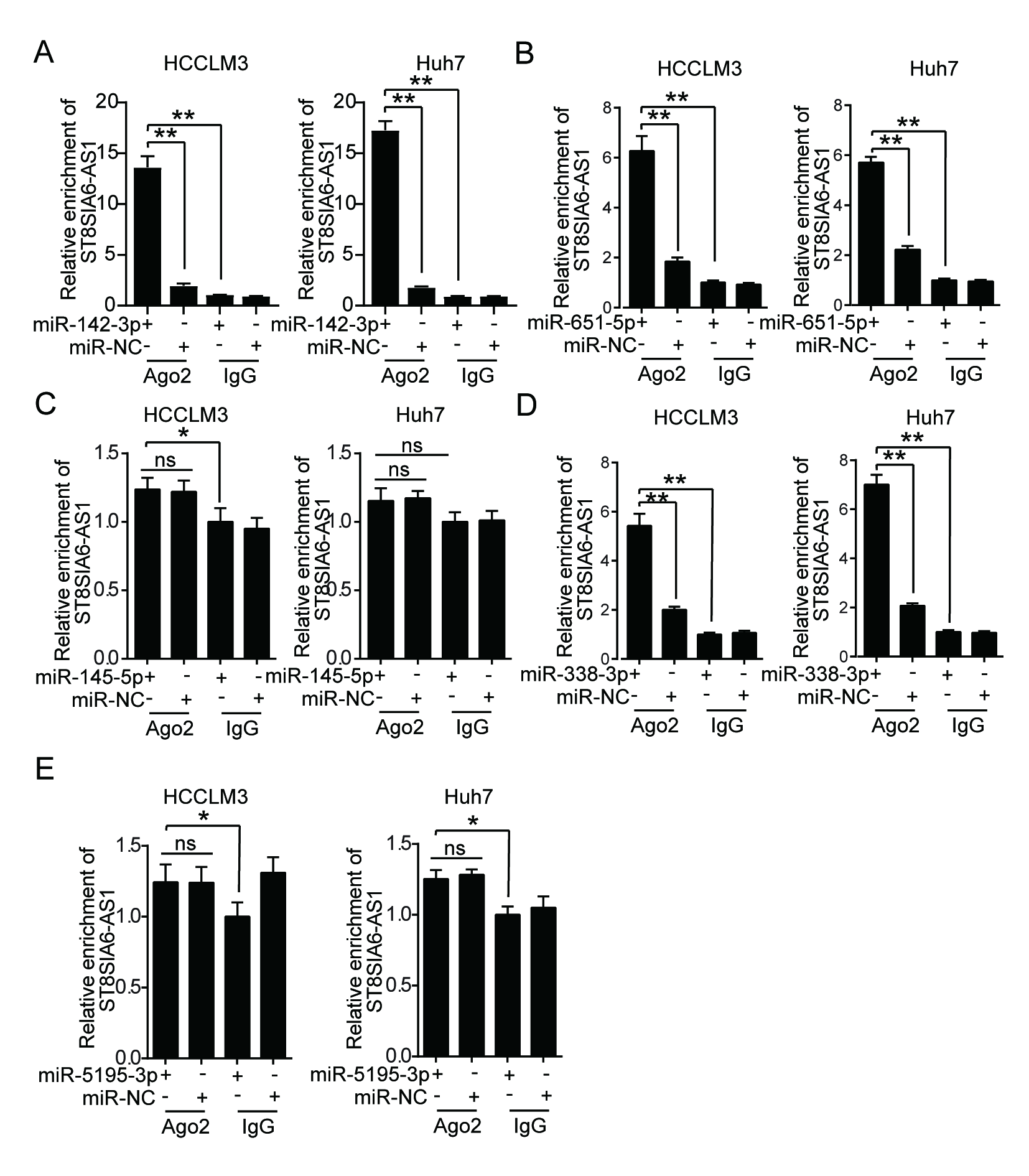

Supplement: Supplementary file 2 — Supplementary Figure 1. [file 41598_2022_26643_MOESM2_ESM.tif]
