# Supplementary material for: ST8SIA6-AS1 contributes to hepatocellular carcinoma progression by targeting miR-142-3p/HMGA1 axis
Source: Sci Rep. 2023 Jan 12;13:650. doi: 10.1038/s41598-022-26643-8 (PMC9837176; doi:10.1038/s41598-022-26643-8)
Supplement: Supplementary file 3 — Supplementary Table 1. [file 41598_2022_26643_MOESM3_ESM.docx]

Supplementary Table 1. Clinical parameters of patients with hepatocellular carcinoma in this study.

| Characteristics | N | ST8SIA6-AS1 expression | | P | miR-142-3p expression | | P | HMGA1 expression | | P |
| --- | --- | --- | --- | --- | --- | --- | --- | --- | --- | --- |
|  |  | High (n=18) | Low (n=17) |  | High (n=18) | Low (n=17) |  | High (n=18) | Low (n=17) |  |
| Gender |  |  |  | 0.305 |  |  | 0.500 |  |  | 0.176 |
| Male | 14 | 9 | 5 |  | 6 | 8 |  | 5 | 9 |  |
| Female | 21 | 9 | 12 |  | 12 | 9 |  | 13 | 8 |  |
| Age (years) |  |  |  | 0.740 |  |  | 0.370 |  |  | 0.181 |
| ≥55 | 18 | 10 | 8 |  | 7 | 11 |  | 7 | 11 |  |
| <55 | 17 | 8 | 9 |  | 11 | 6 |  | 11 | 6 |  |
| Tumor size (cm) |  |  |  | 0.146 |  |  | 0.181 |  |  | 0.075 |
| ≥5 | 11 | 8 | 3 |  | 4 | 7 |  | 3 | 8 |  |
| <5 | 24 | 10 | 14 |  | 14 | 10 |  | 15 | 9 |  |
| TNM stage |  |  |  | 0.041 |  |  | 0.289 |  |  | <0.001 |
| I+II | 20 | 7 | 13 |  | 15 | 5 |  | 17 | 3 |  |
| III+IV | 15 | 11 | 4 |  | 3 | 12 |  | 1 | 14 |  |
| Lymph node metastasis |  |  |  | 0.044 |  |  | 0.002 |  |  | 0.001 |
| Positive | 19 | 13 | 6 | 19 | 5 | 14 |  | 15 | 4 |  |
| Negative | 16 | 5 | 11 |  | 13 | 3 |  | 3 | 13 |  |
| Liver cirrhosis |  |  |  | 0.489 |  |  | 0.086 |  |  | 0.489 |
| Presence | 13 | 8 | 5 |  | 4 | 9 |  | 8 | 5 |  |
| Absence | 22 | 10 | 12 |  | 14 | 8 |  | 10 | 12 |  |
| Serum AFP (ng/mL) |  |  |  | 0.044 |  |  | 0.007 |  |  | 0.044 |
| ≤20 | 18 | 6 | 12 |  | 13 | 4 |  | 6 | 12 |  |
| >20 | 17 | 12 | 5 |  | 5 | 13 |  | 12 | 5 |  |

The correlation was analyzed by Fisher's exact test.
