# Supplementary material for: ST8SIA6-AS1 contributes to hepatocellular carcinoma progression by targeting miR-142-3p/HMGA1 axis
Source: Sci Rep. 2023 Jan 12;13:650. doi: 10.1038/s41598-022-26643-8 (PMC9837176; doi:10.1038/s41598-022-26643-8)
Supplement: Supplementary file 5 — Supplementary Information 5. [file 41598_2022_26643_MOESM5_ESM.pdf]

Figure 3C

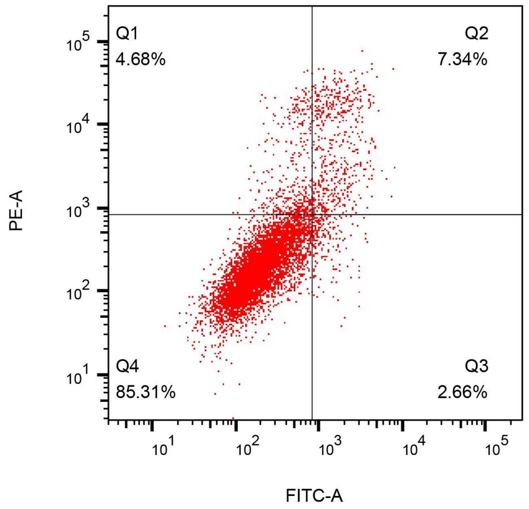

HCCLM3 Blank

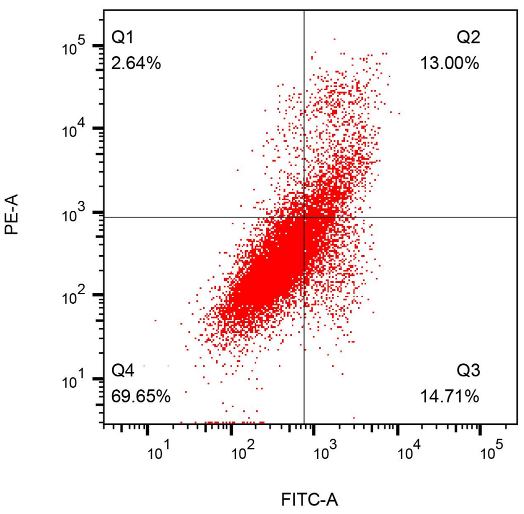

HCCLM3 si-lnc

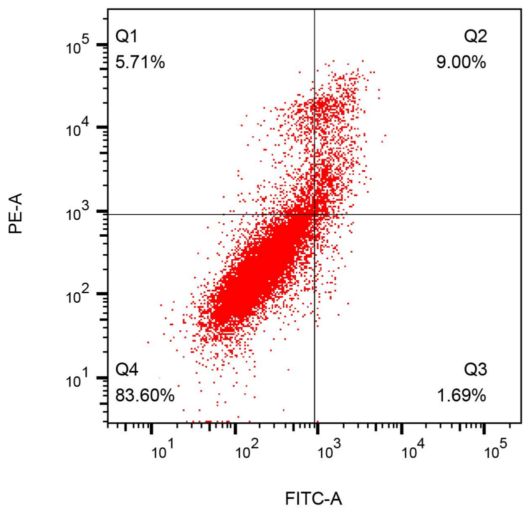

HCCLM3 si-NC

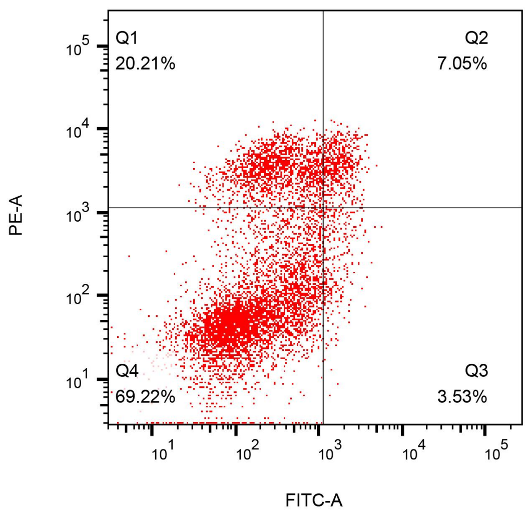

Huh7 blank

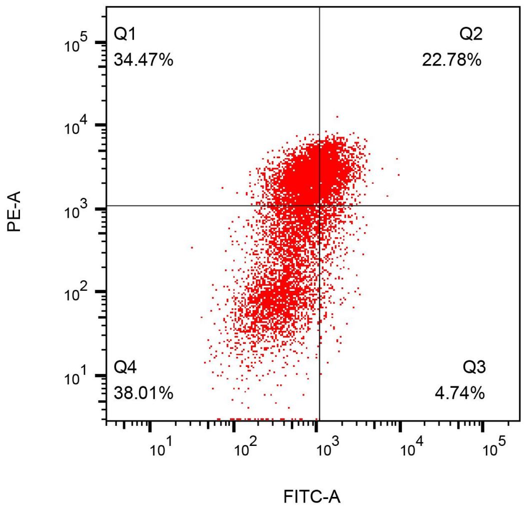

Huh7 si-lnc

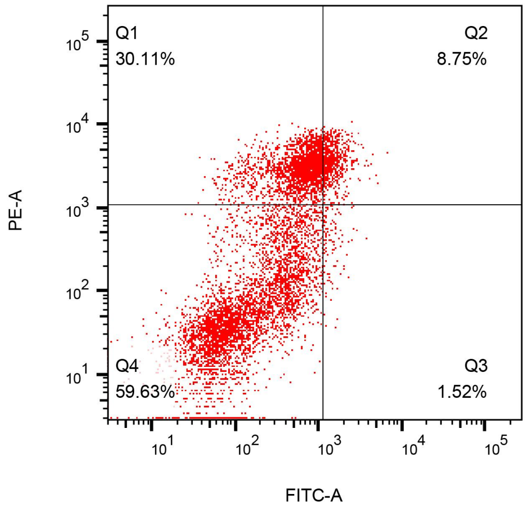

Huh7 si-NC

Figure 6C

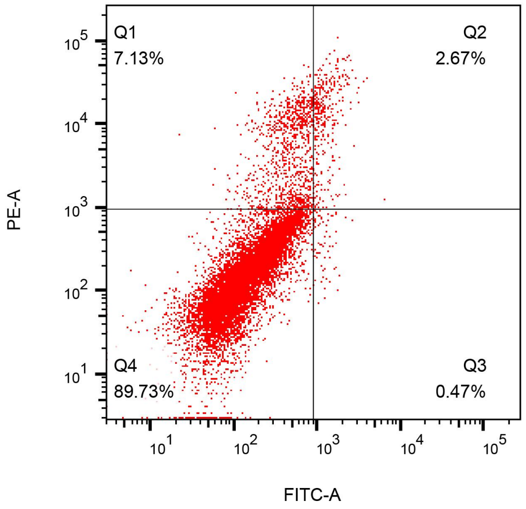

HCCLM3 anti-miR

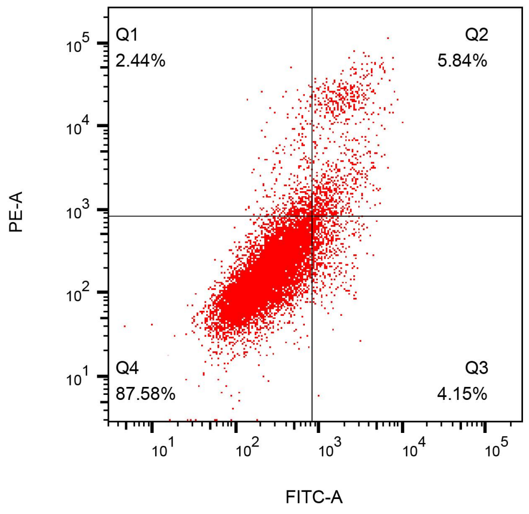

HCCLM3 blank

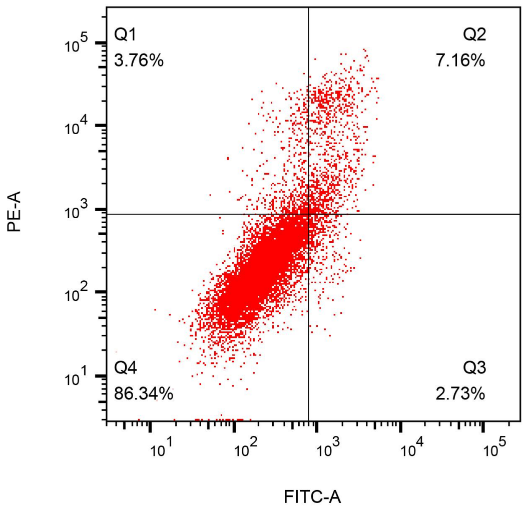

HCCLM3 si-lnc+anti-miR

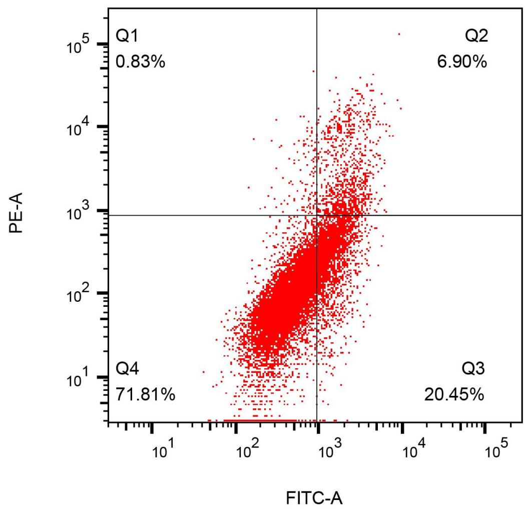

HCCLM3 si-lnc+anti-miR-NC

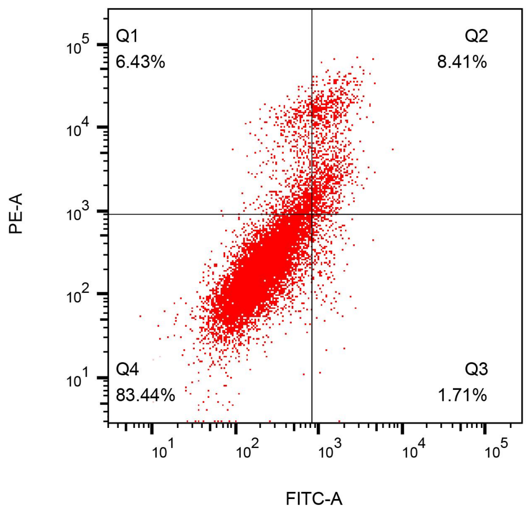

HCCLM3 si-NC

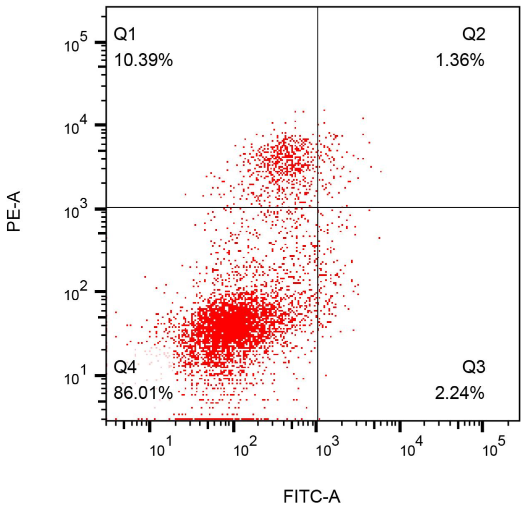

Huh7 anti-miR

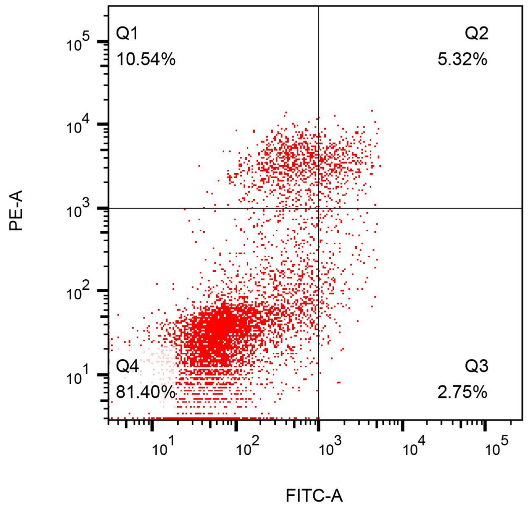

Huh7 blank

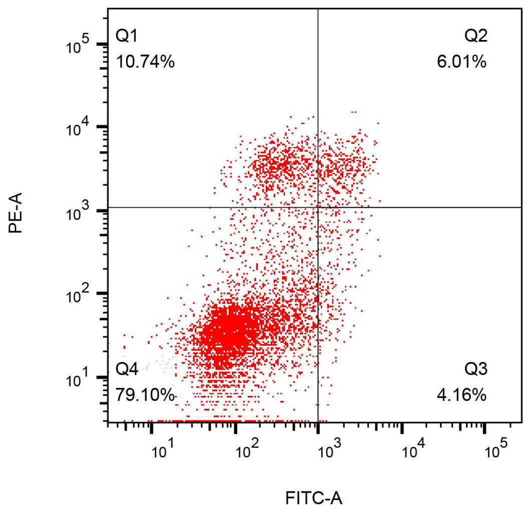

Huh7 si-lnc+anti-miR

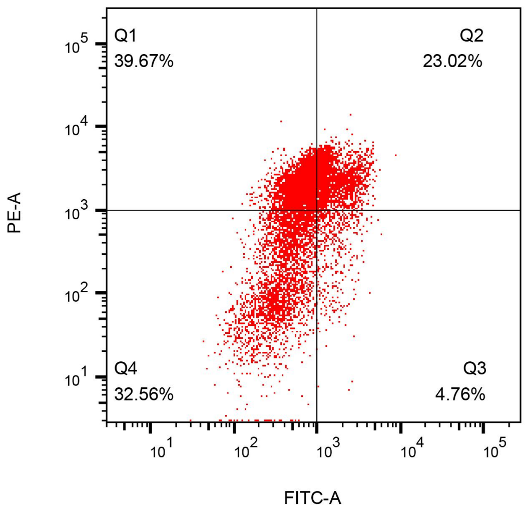

Huh7 si-lnc+anti-miR-NC

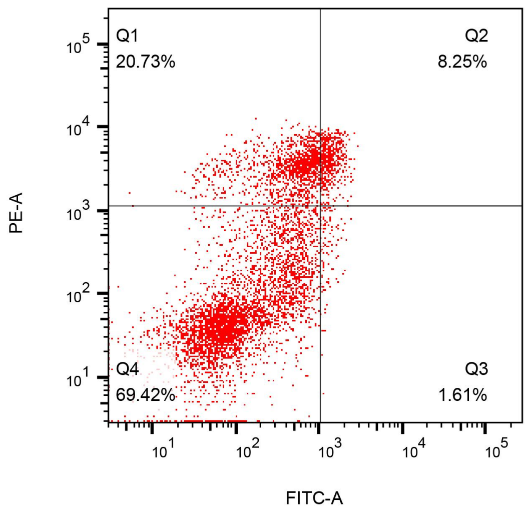

Huh7 si-NC+anti-miR-NC

Figure 9C

Repeat 1

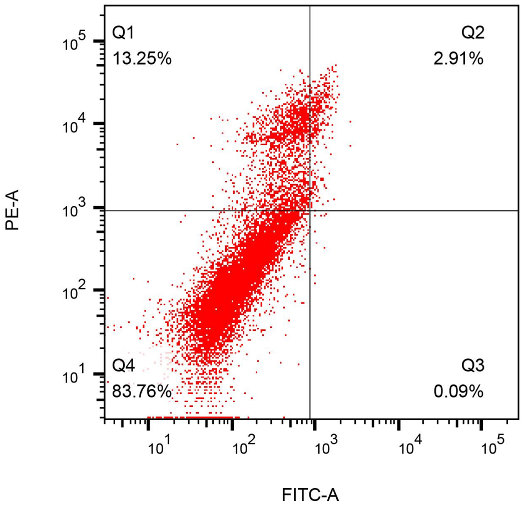

HCCLM3 anti-miR

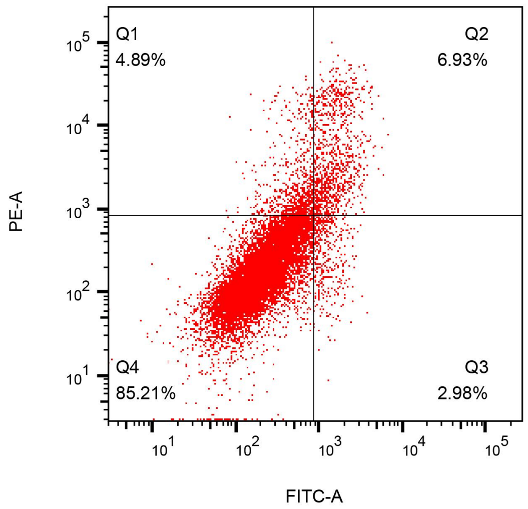

HCCLM3 blank

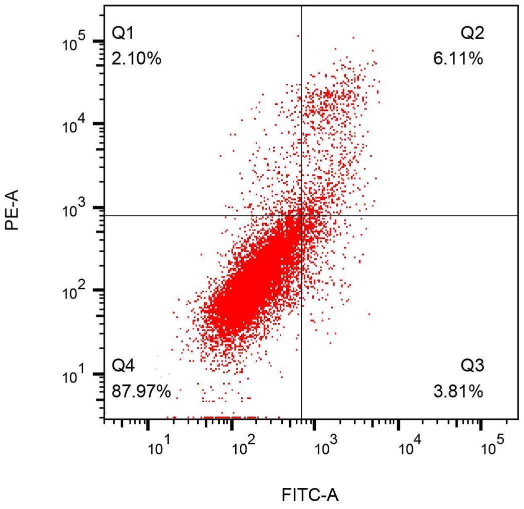

HCCLM3 si-HMGA1+anti-miR

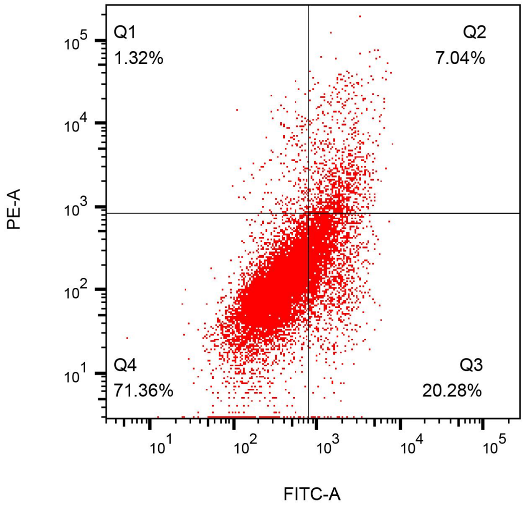

HCCLM3 si-HMGA1+anti-miR-NC

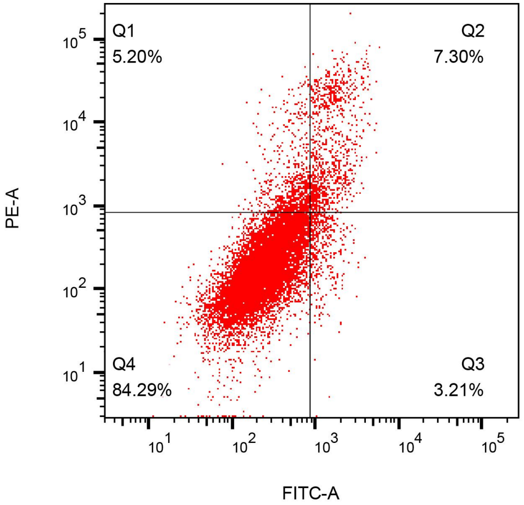

HCCLM3 si-NC+anti-miR-NC

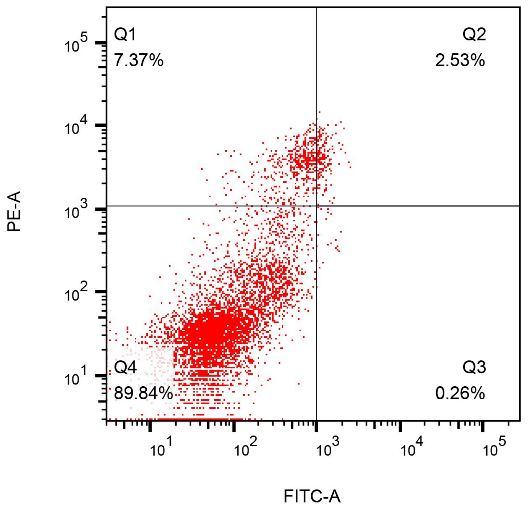

Huh7 anti-miR

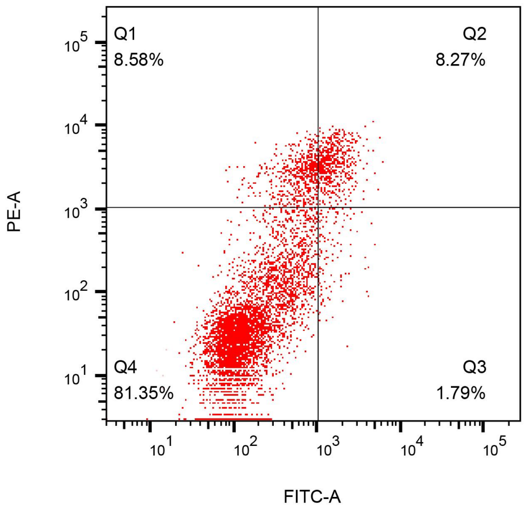

Huh7 blank

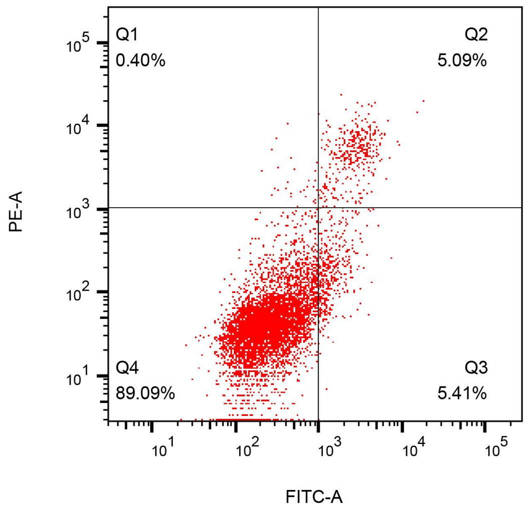

Huh7 si-HMGA1+anti-miR

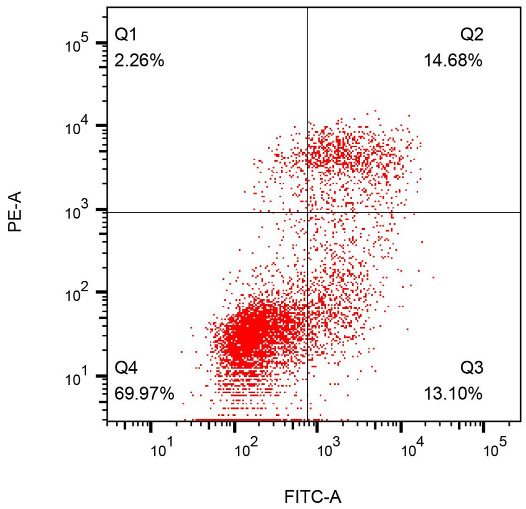

Huh7 si-HMGA1+anti-miR-NC

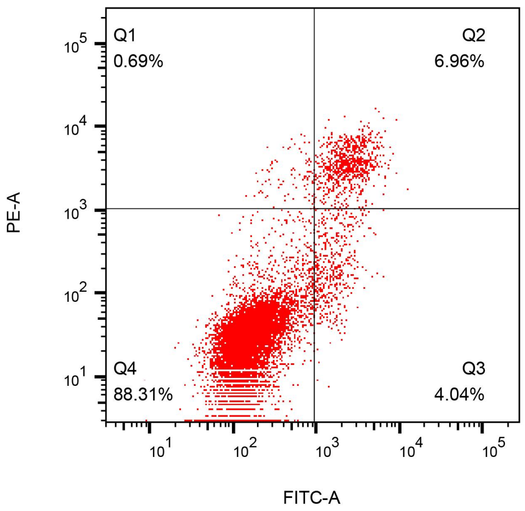

Huh7 si-NC+anti-miR-NC
