# Supplementary material for: ST8SIA6-AS1 contributes to hepatocellular carcinoma progression by targeting miR-142-3p/HMGA1 axis
Source: Sci Rep. 2023 Jan 12;13:650. doi: 10.1038/s41598-022-26643-8 (PMC9837176; doi:10.1038/s41598-022-26643-8)
Supplement: Supplementary file 6 — Supplementary Information 6. [file 41598_2022_26643_MOESM6_ESM.pdf]

Figure 3D

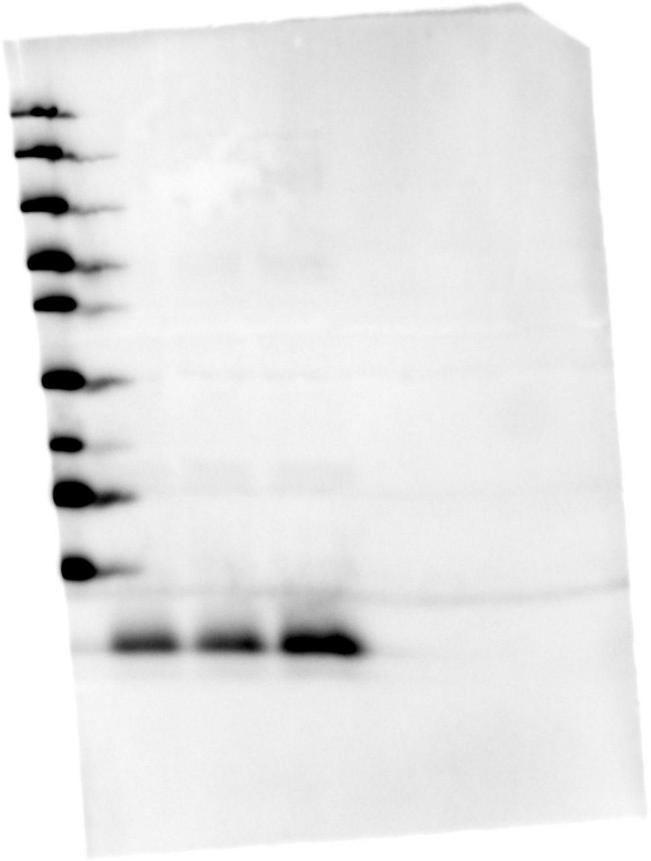

HCCLM3 Bax

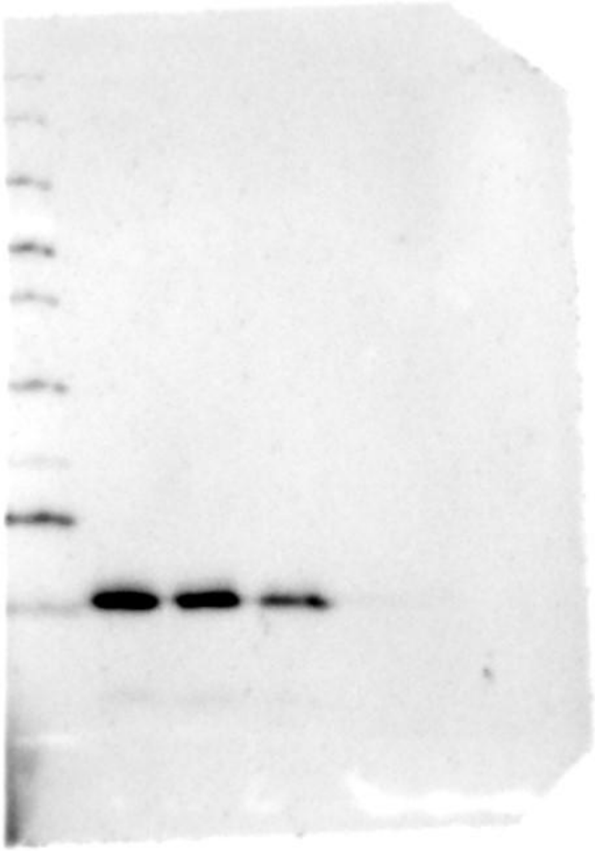

HCCLM3 Bcl-2

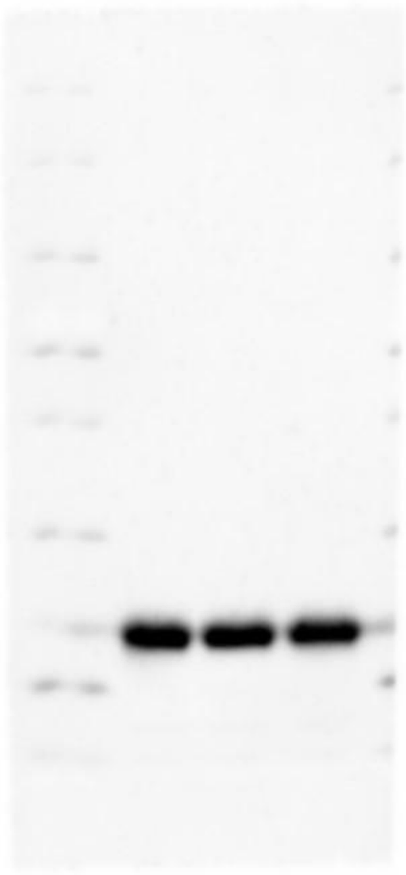

HCCLM3 GAPDH

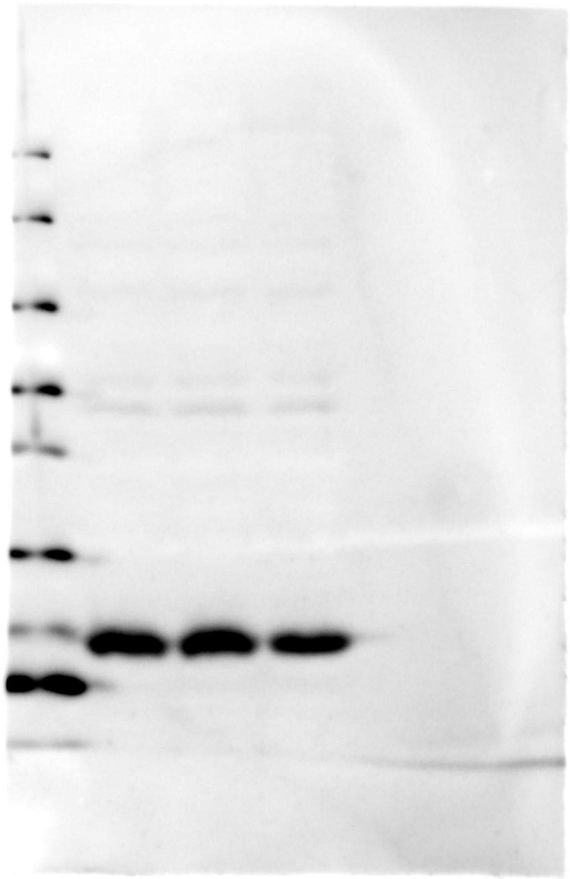

HCCLM3 PCNA

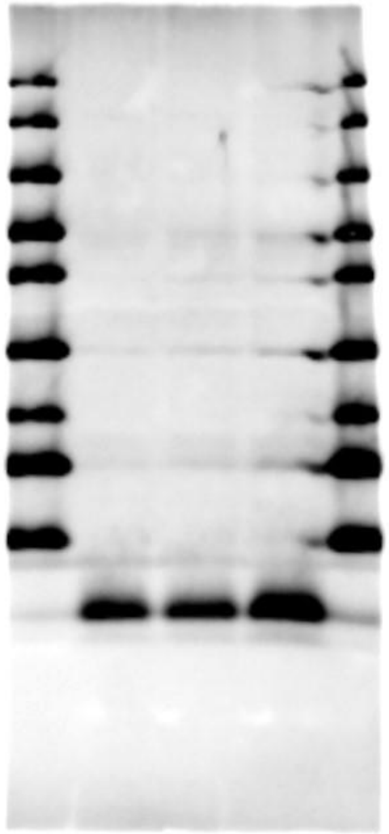

Huh7 Bax

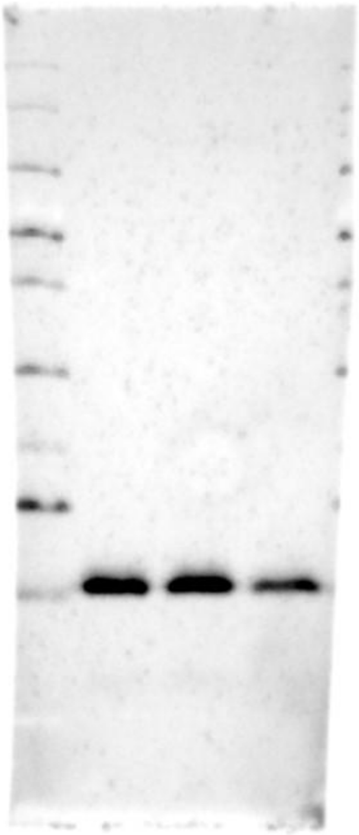

Huh7 Bcl-2

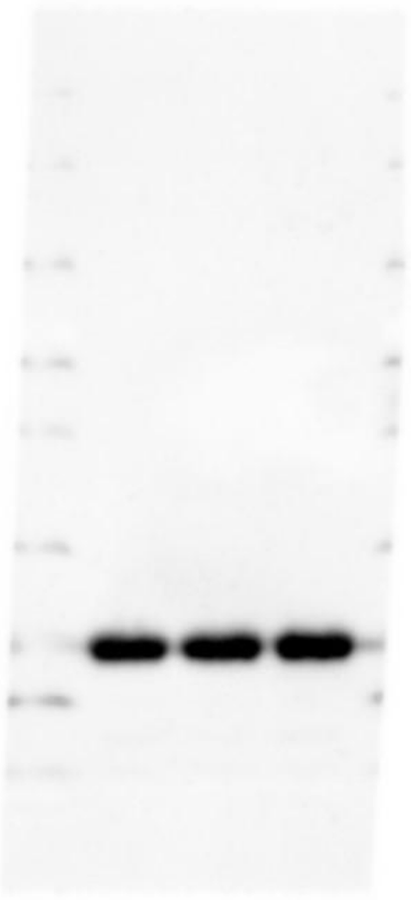

Huh7 GAPDH

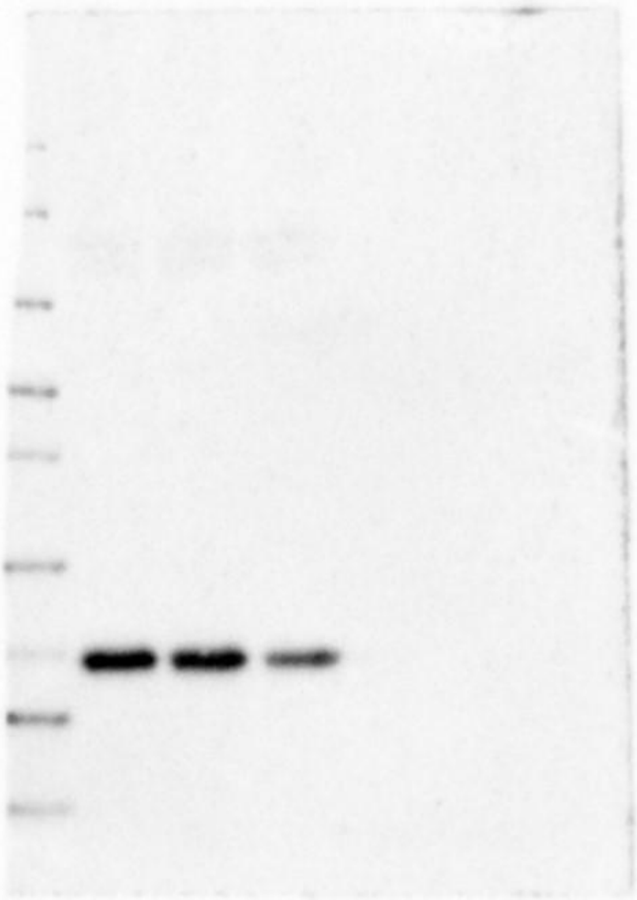

Huh7 PCNA

Figure 6D

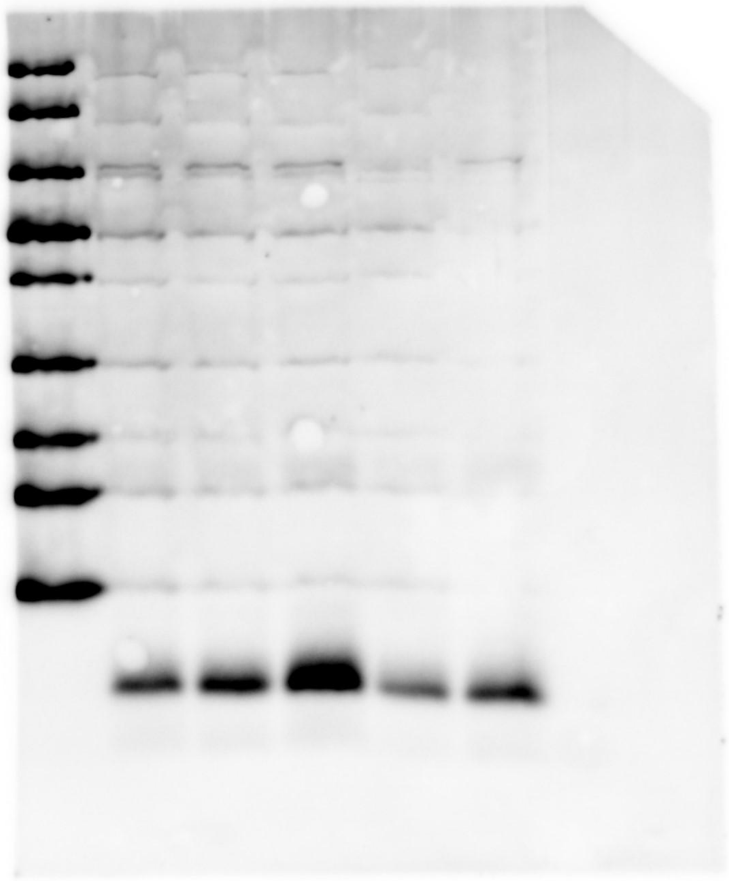

HCCLM3 Bax

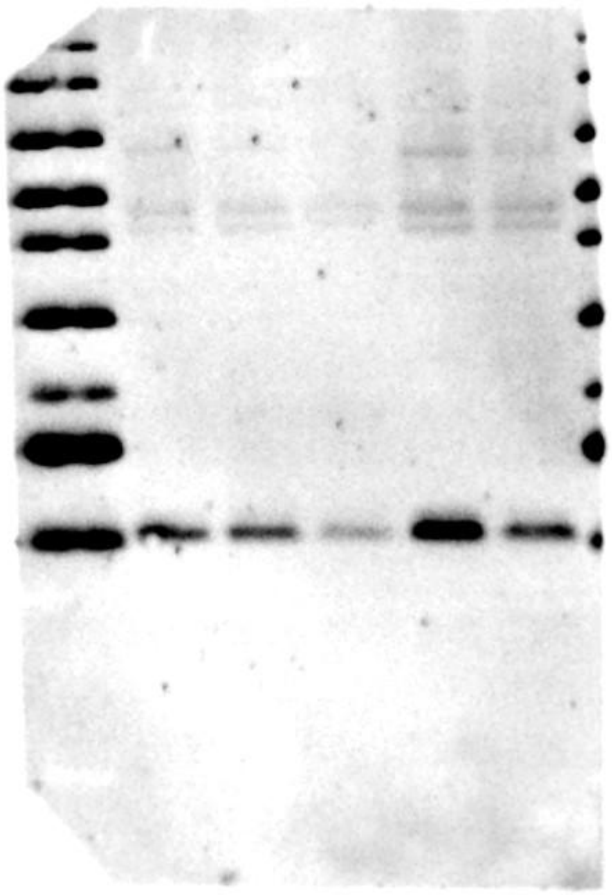

HCCLM3 Bcl-2

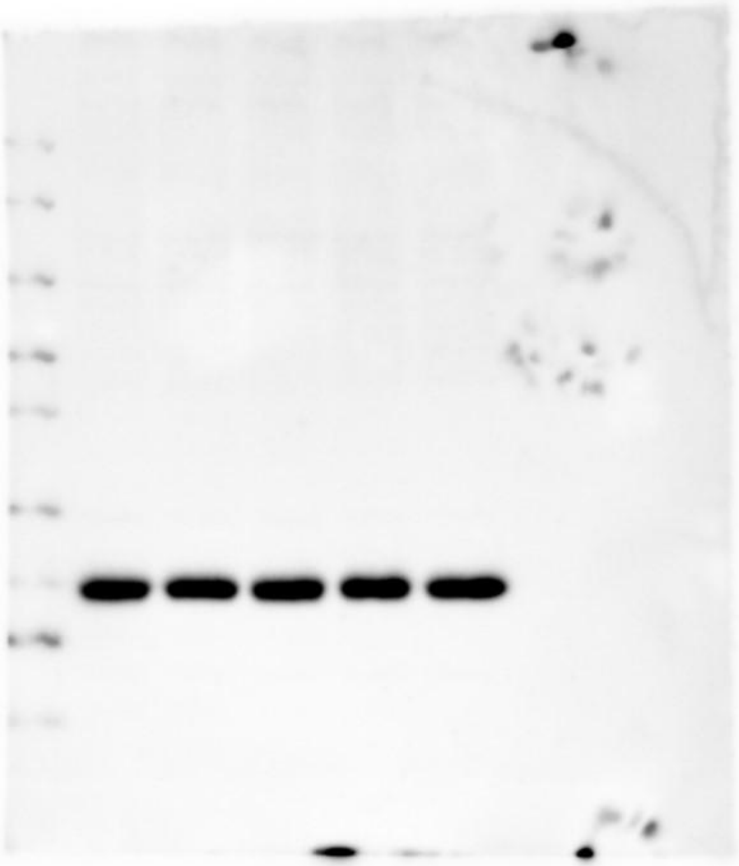

HCCLM3 GAPDH

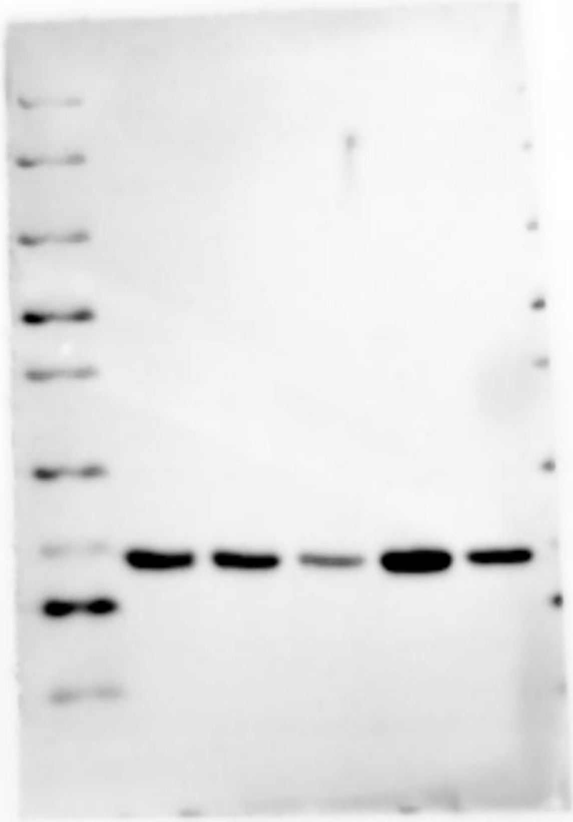

HCCLM3 PCNA

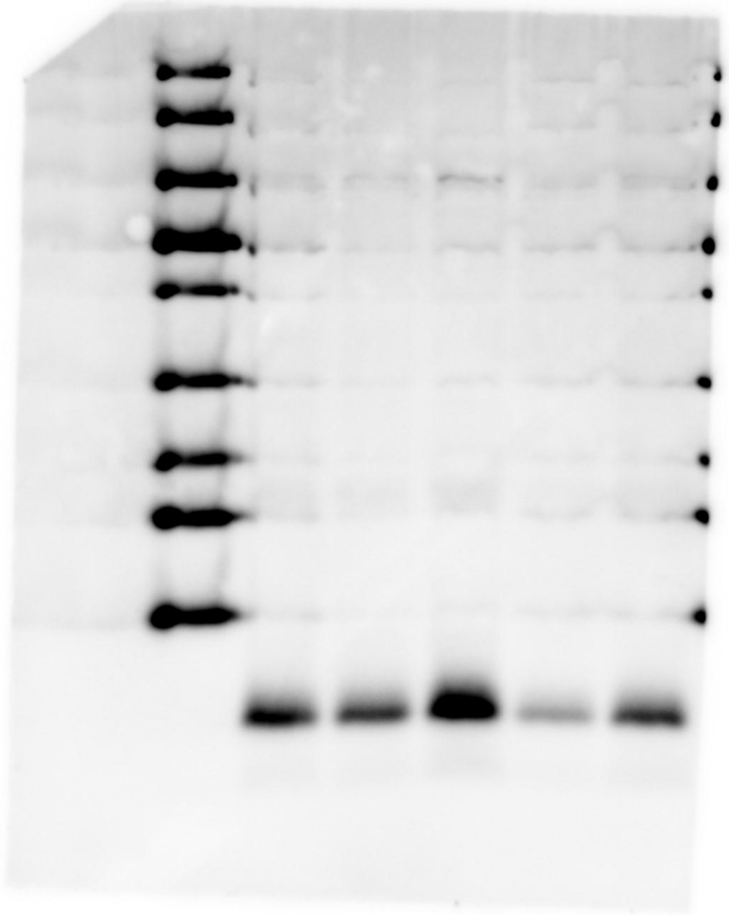

Huh7 Bax

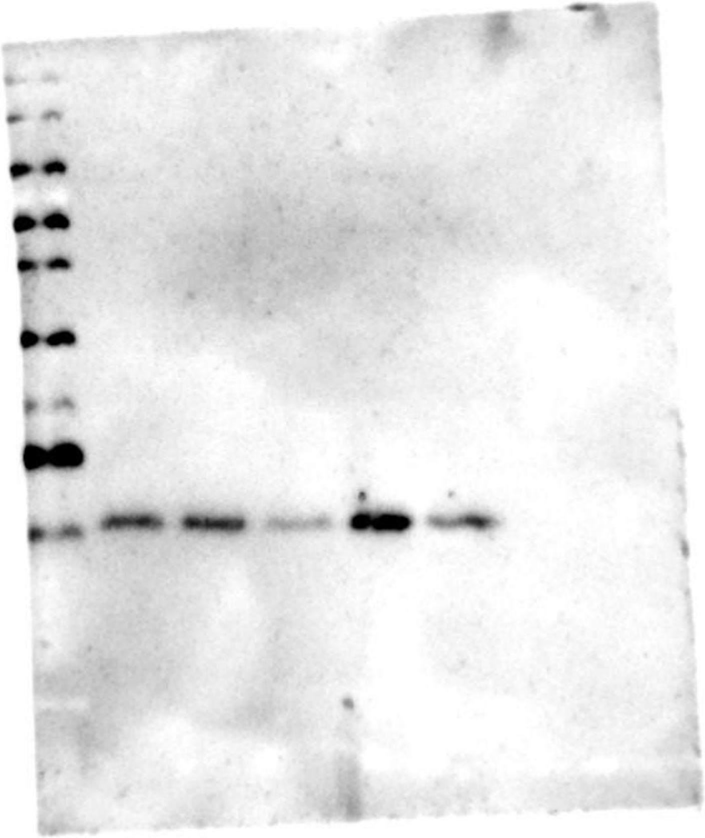

Huh7 Bcl-2

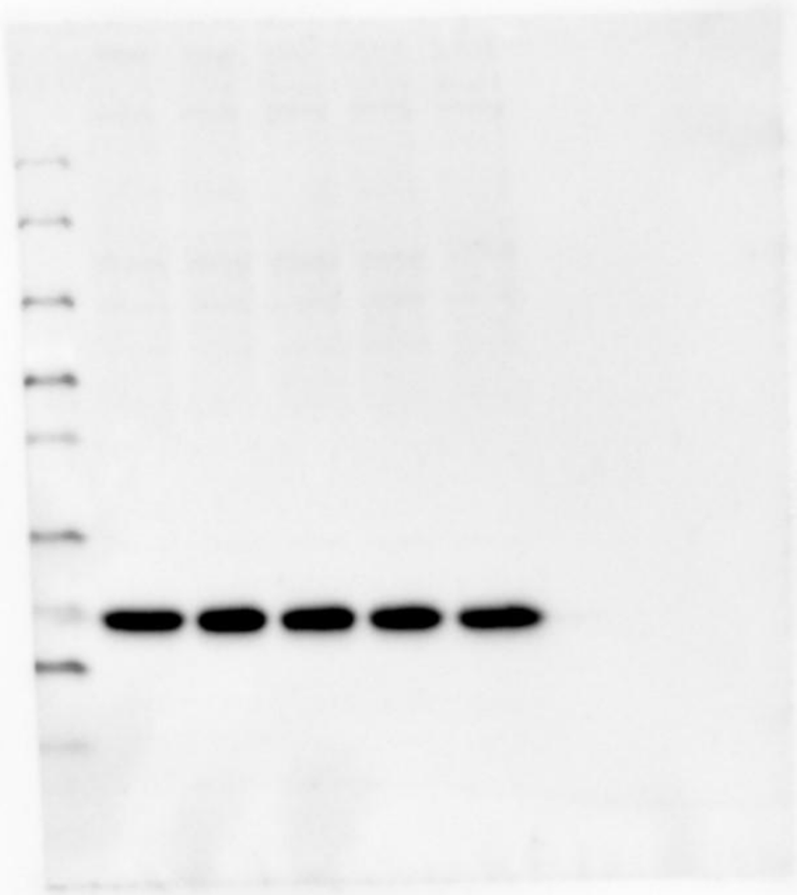

Huh7 GAPDH

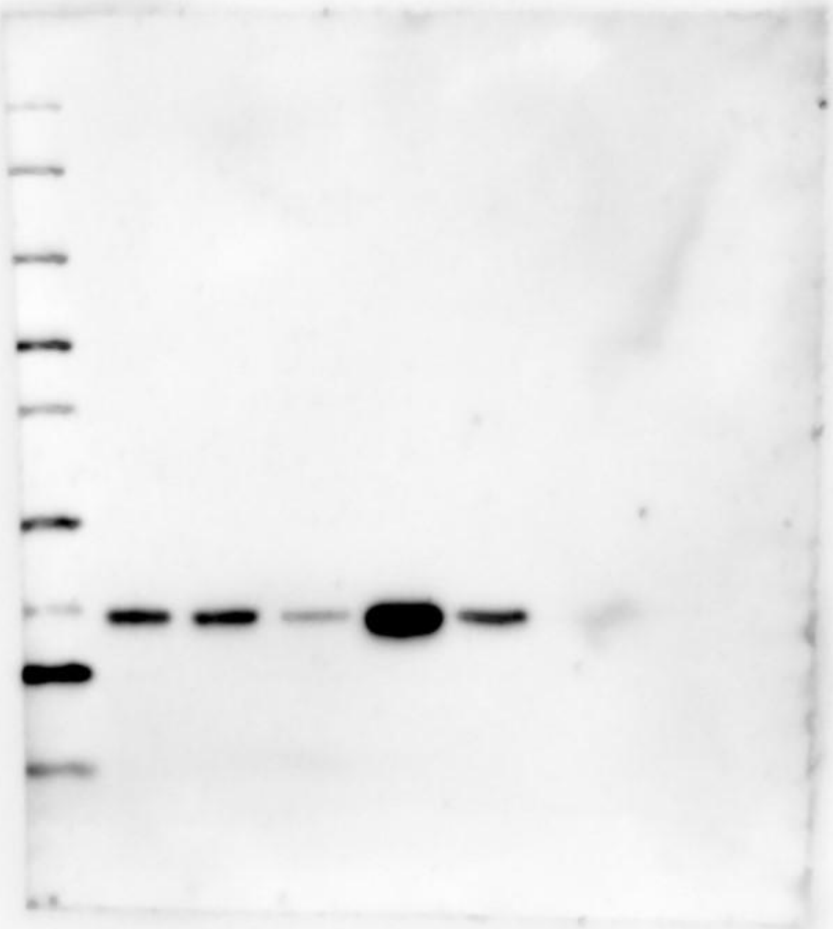

Huh7 PCNA

Figure 8G

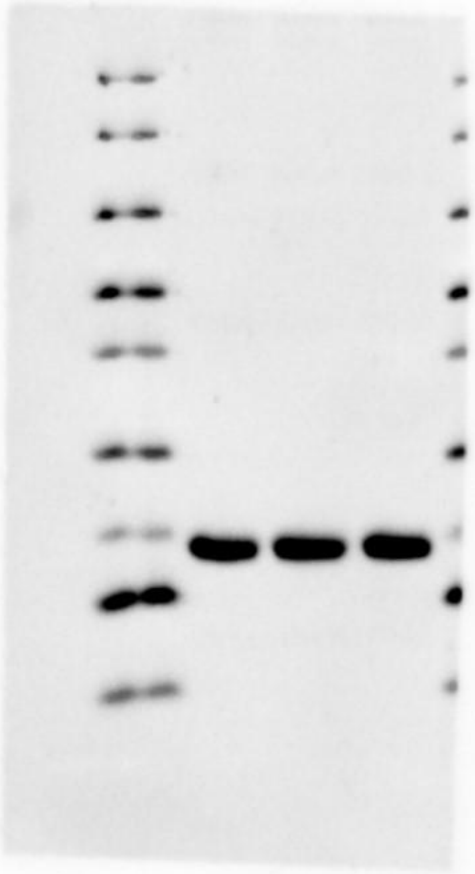

GAPDH

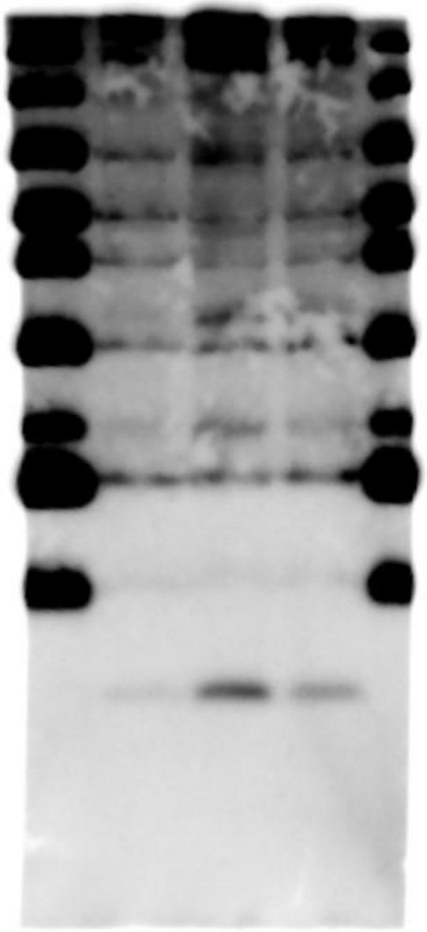

HMGA1

Figure 8I

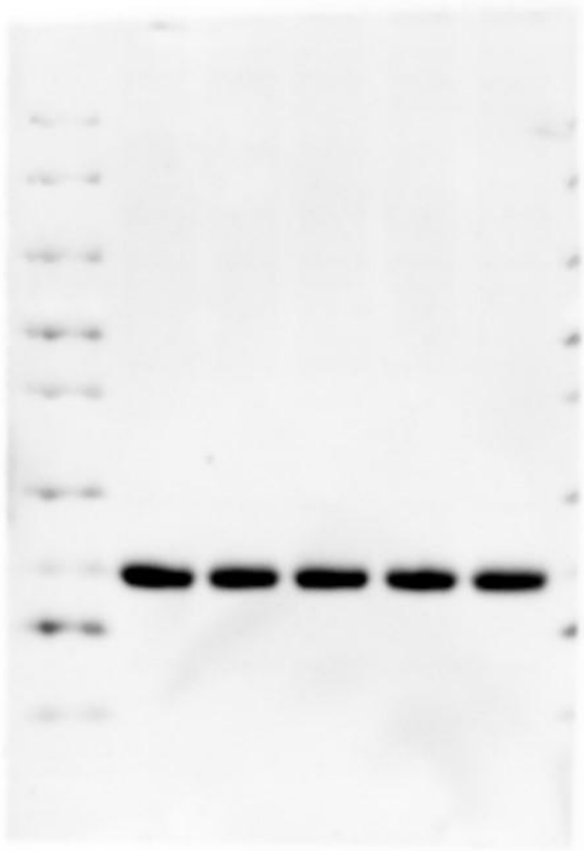

HCCLM3 GAPDH

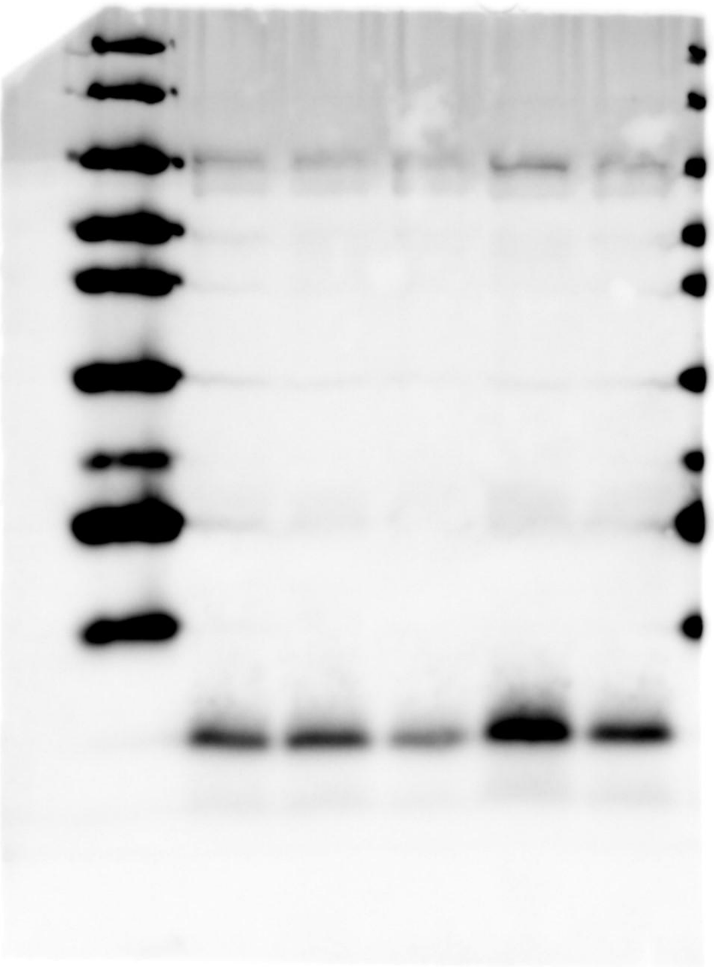

HCCLM3 HMGA1

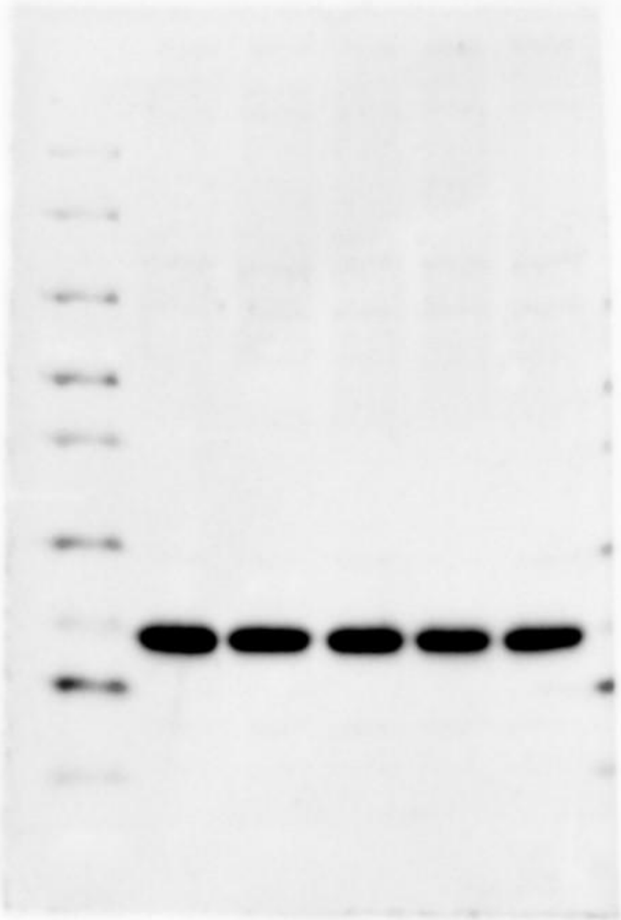

Huh7 GAPDH

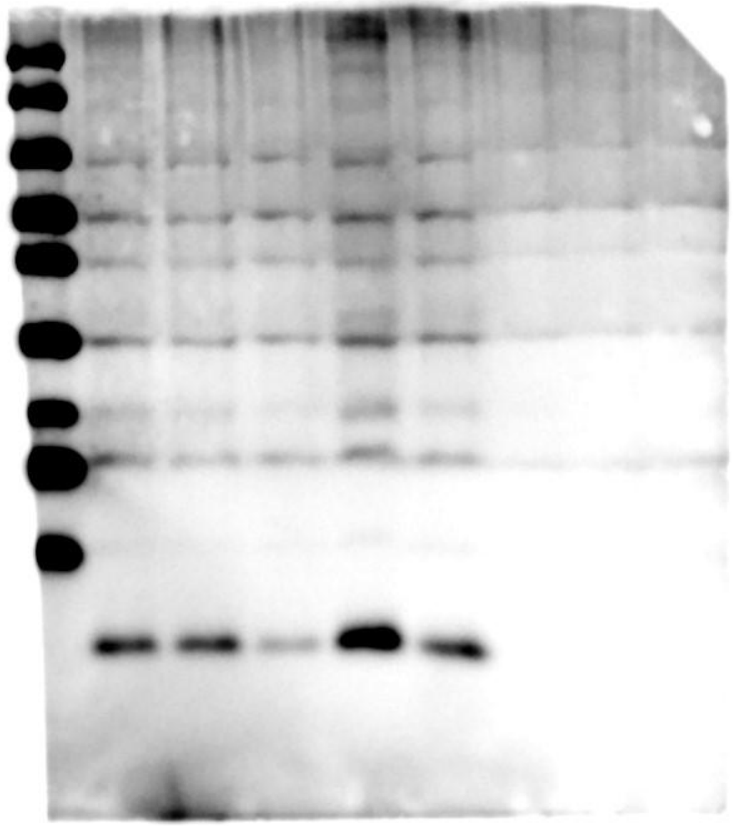

Huh7 HMGA1

Figure 8J

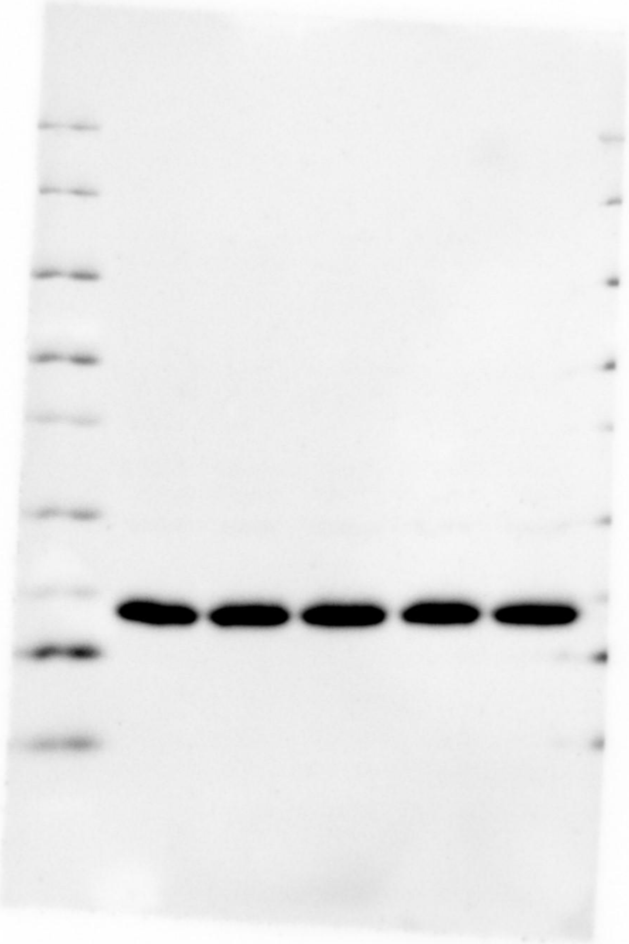

HCCLM3 GAPDH

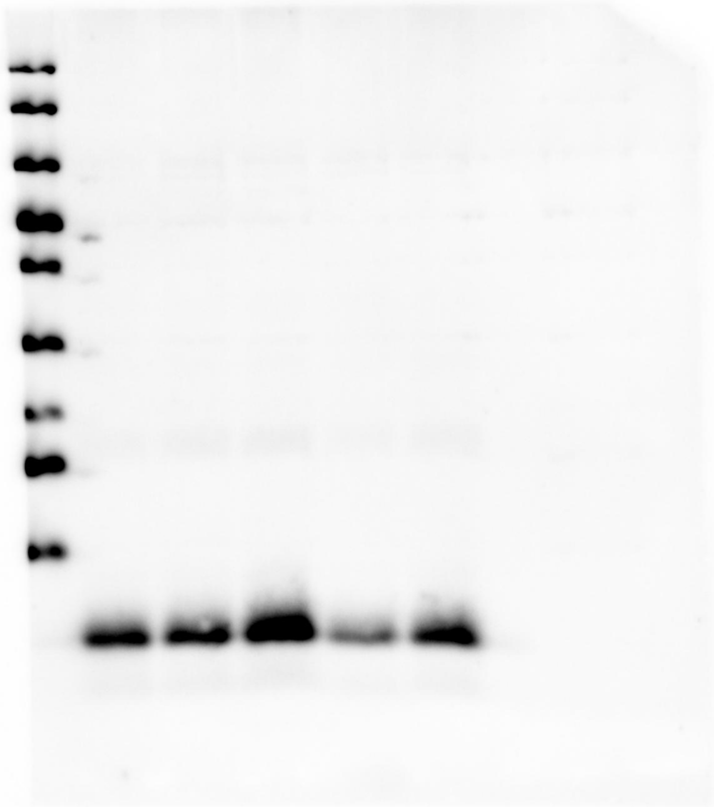

HCCLM3 HMGA1

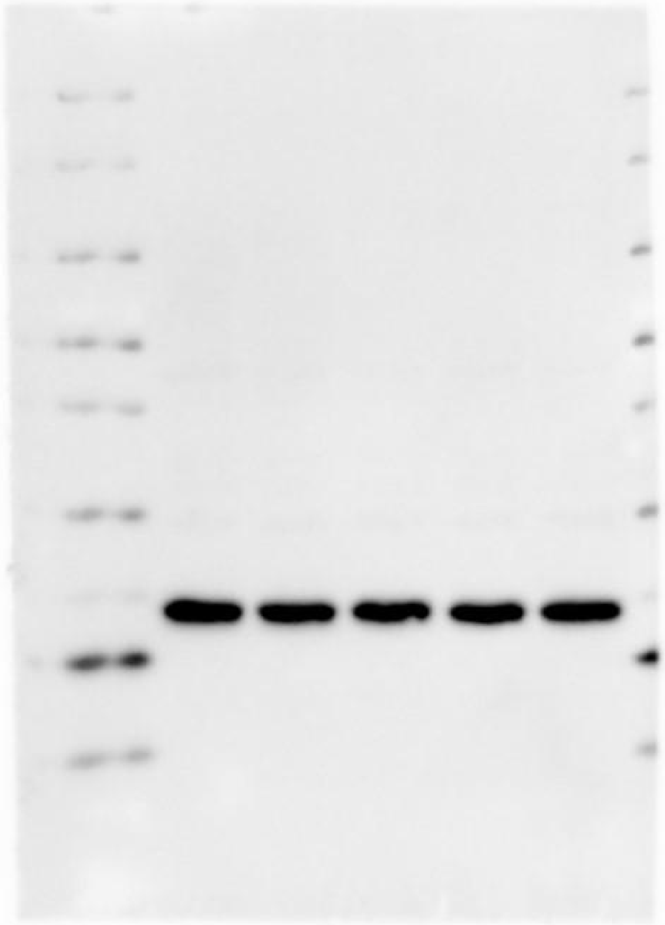

Huh7 GAPDH

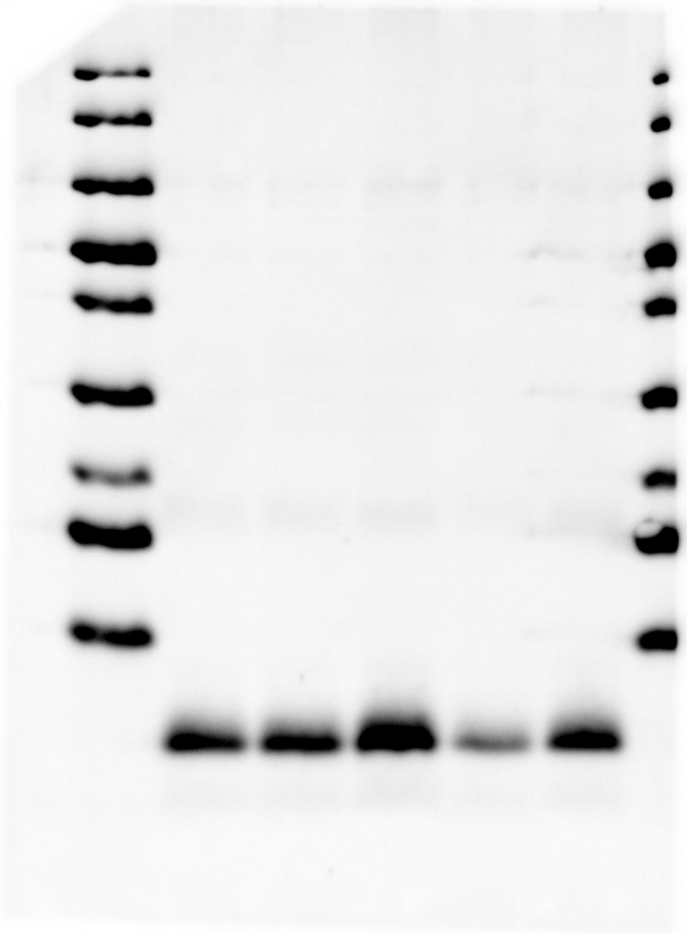

Huh7 HMGA1

Figure 9D

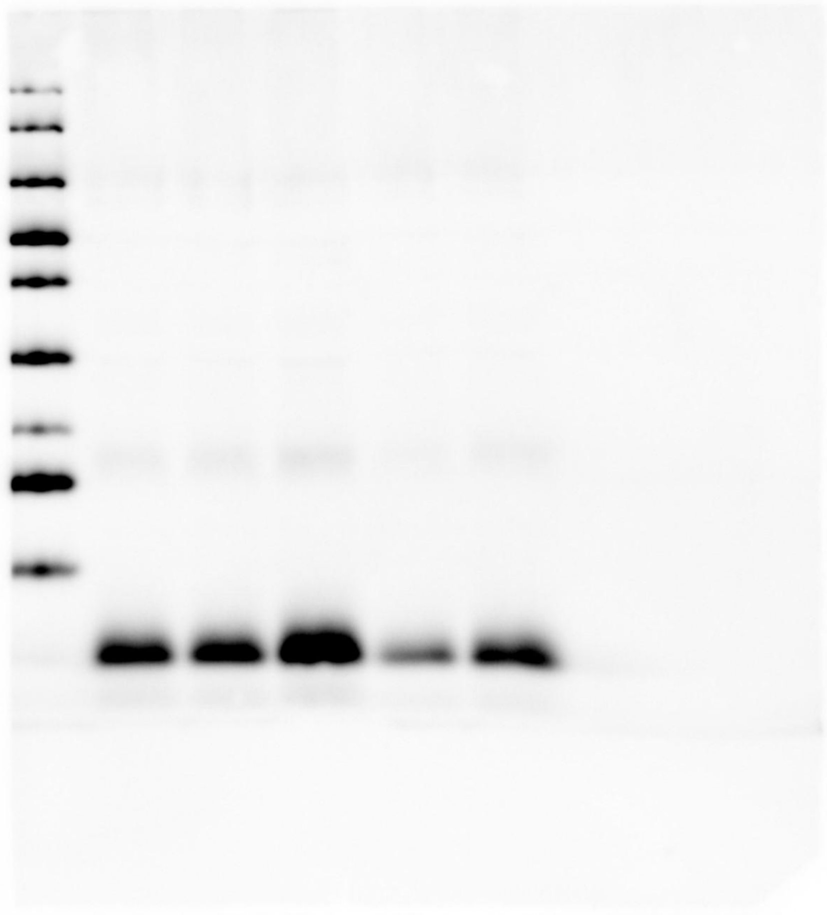

HCCLM3 Bax

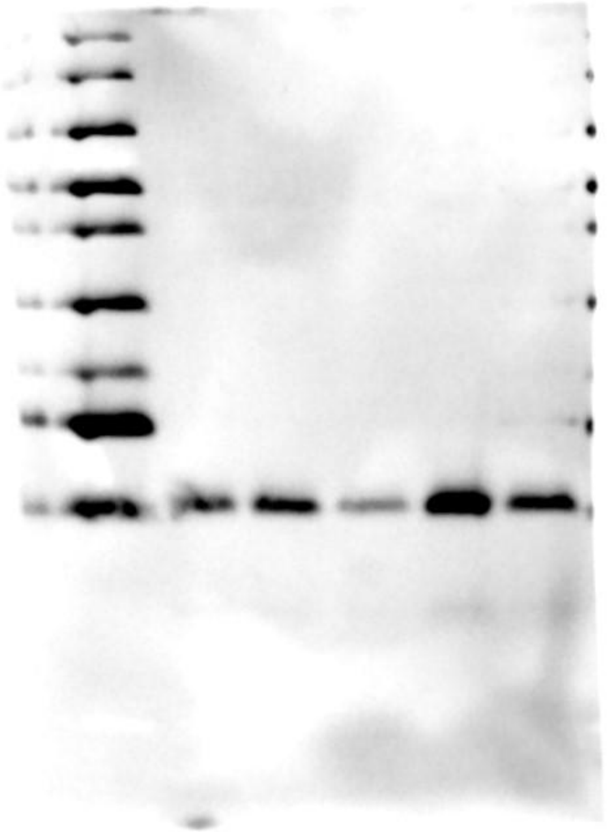

HCCLM3 Bcl-2

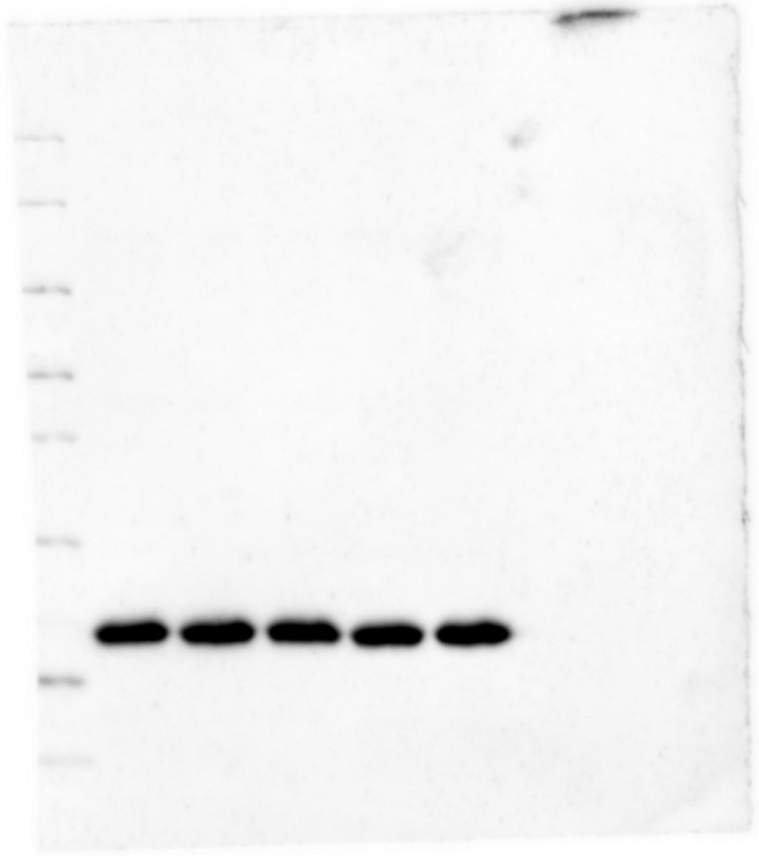

HCCLM3 GAPDH

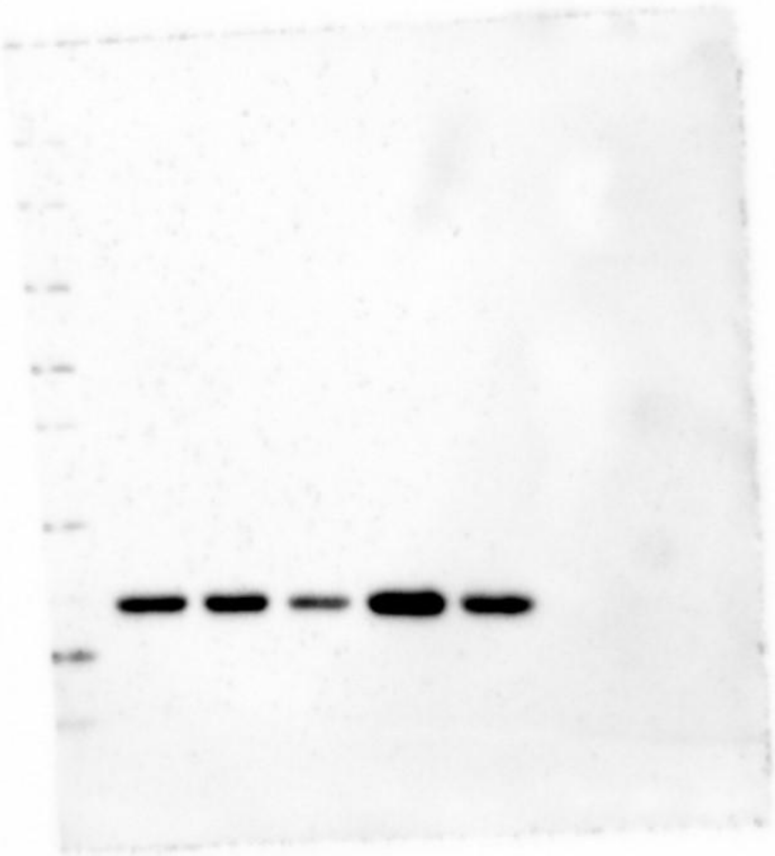

HCCLM3 PCNA

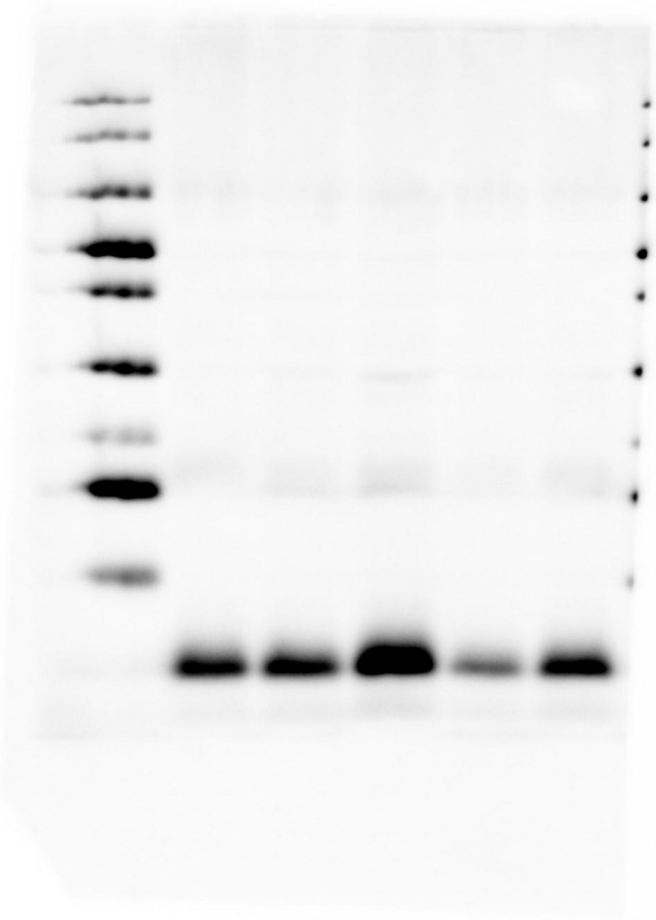

Huh7 Bax

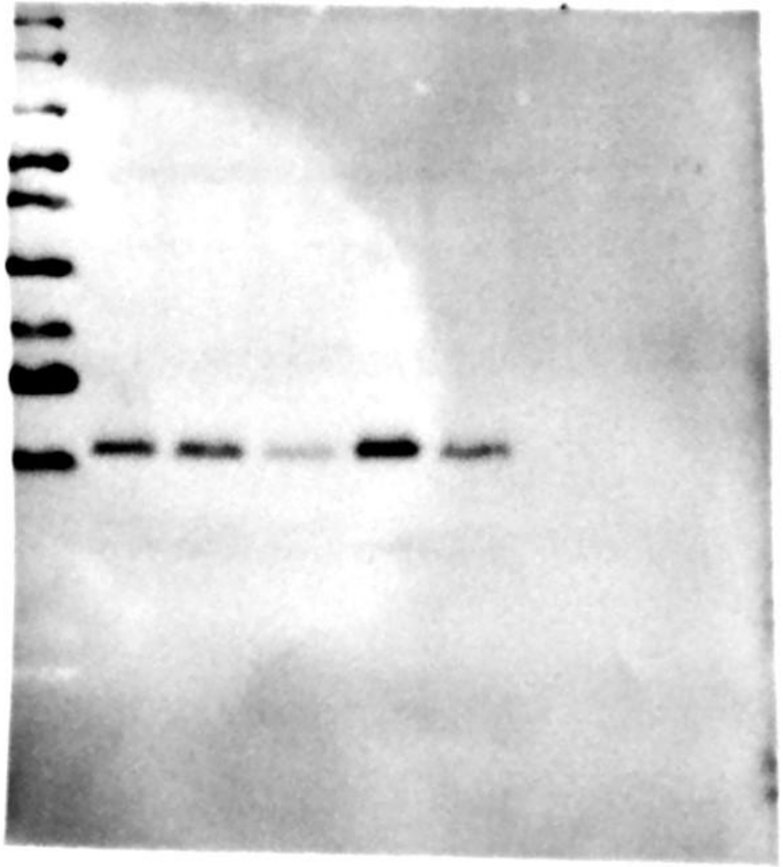

Huh7 Bcl-2

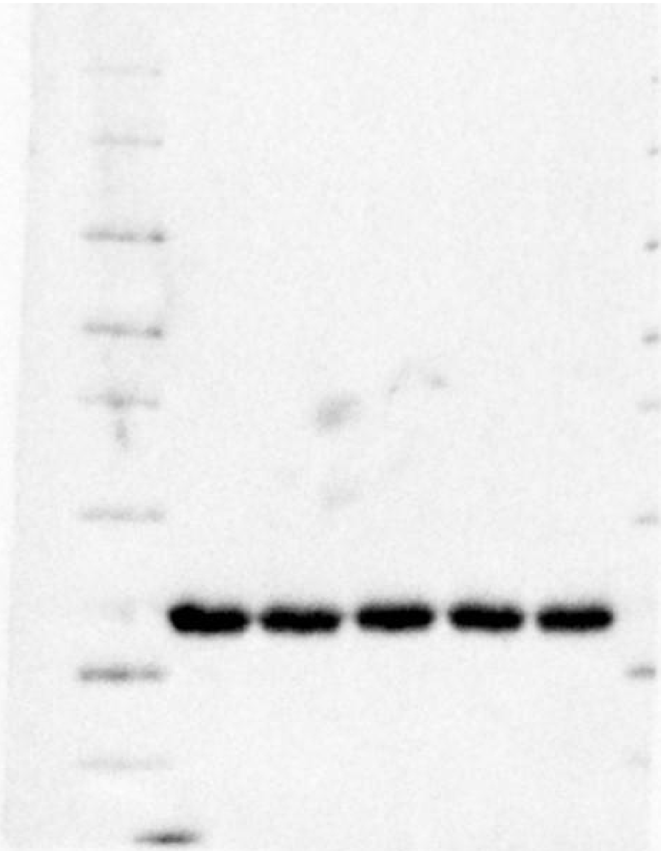

Huh7 GAPDH

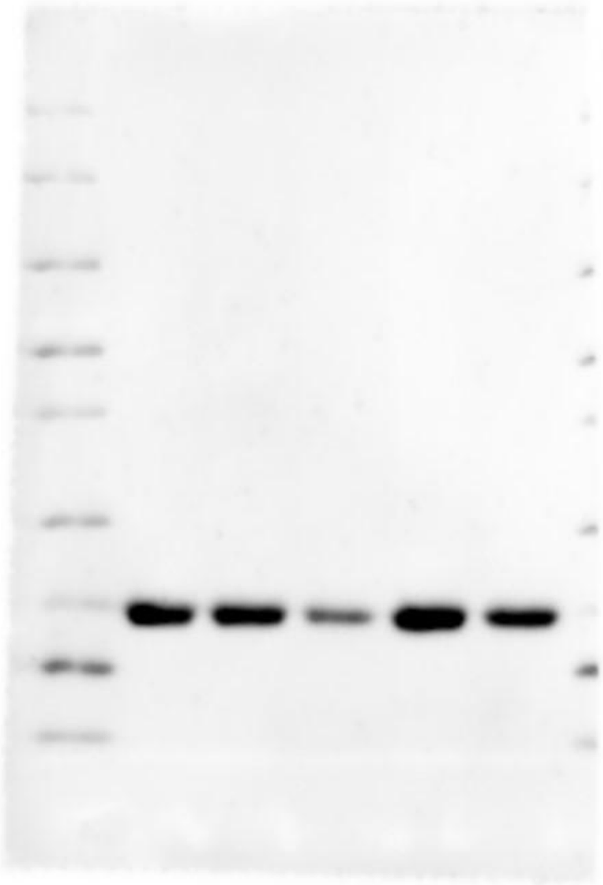

Huh7 PCNA
