# Supplementary material for: ST8SIA6-AS1 contributes to hepatocellular carcinoma progression by targeting miR-142-3p/HMGA1 axis
Source: Sci Rep. 2023 Jan 12;13:650. doi: 10.1038/s41598-022-26643-8 (PMC9837176; doi:10.1038/s41598-022-26643-8)
Supplement: Supplementary file 4 — Supplementary Information 4. [file 41598_2022_26643_MOESM4_ESM.pdf]

Figure 3B

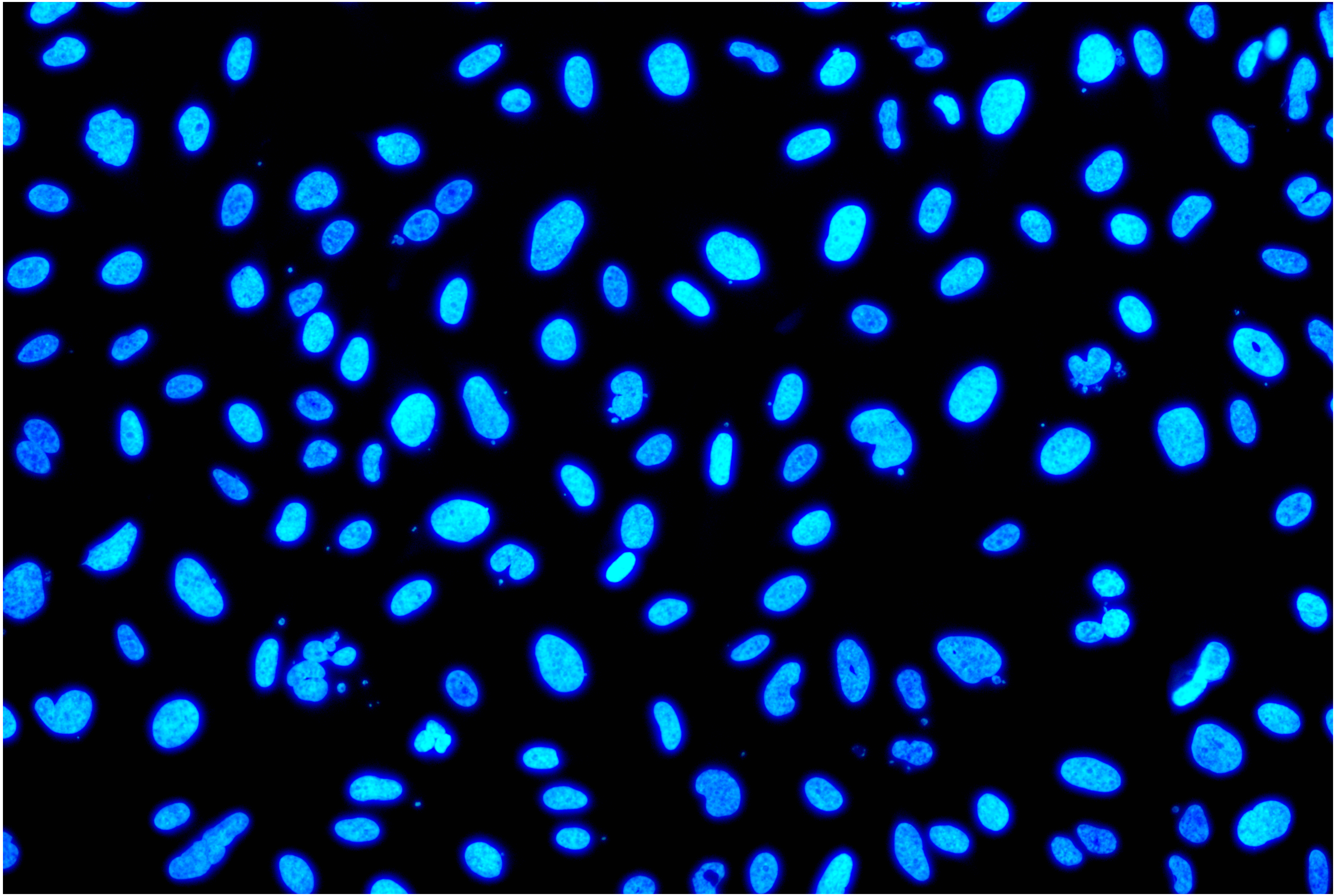

HCCLM3 blank DAPI

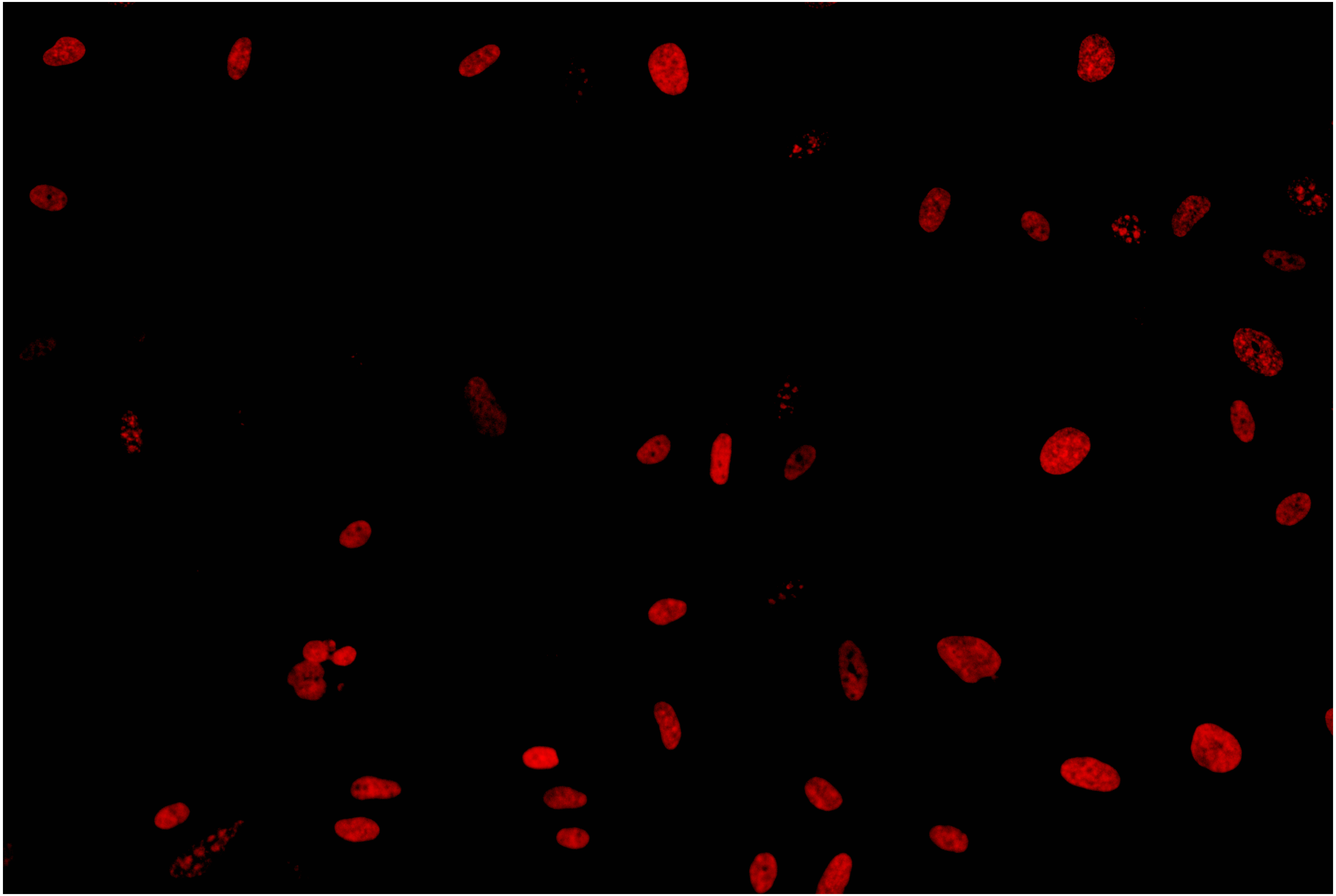

HCCLM3 blank EDU

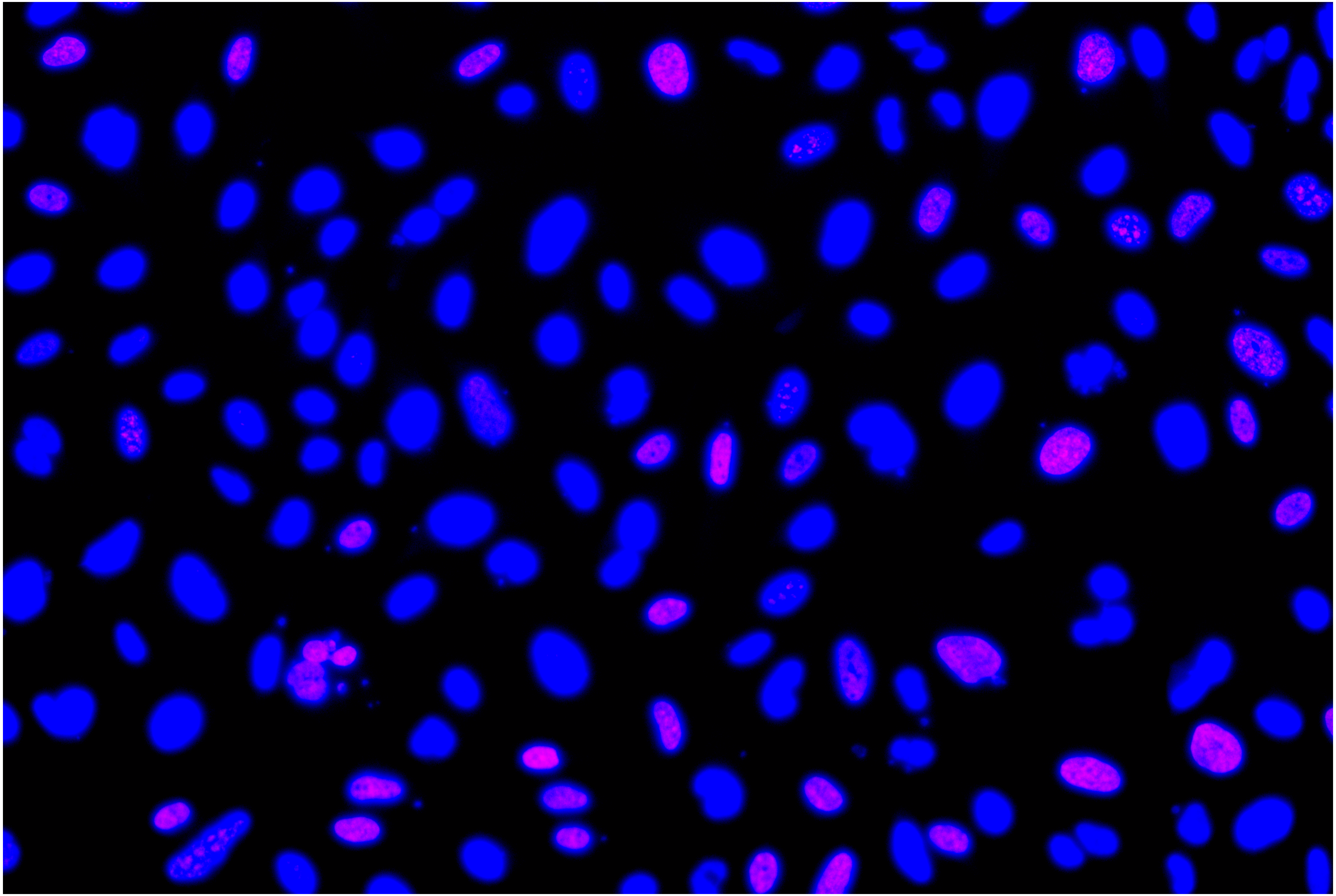

HCCLM3 blank Merge

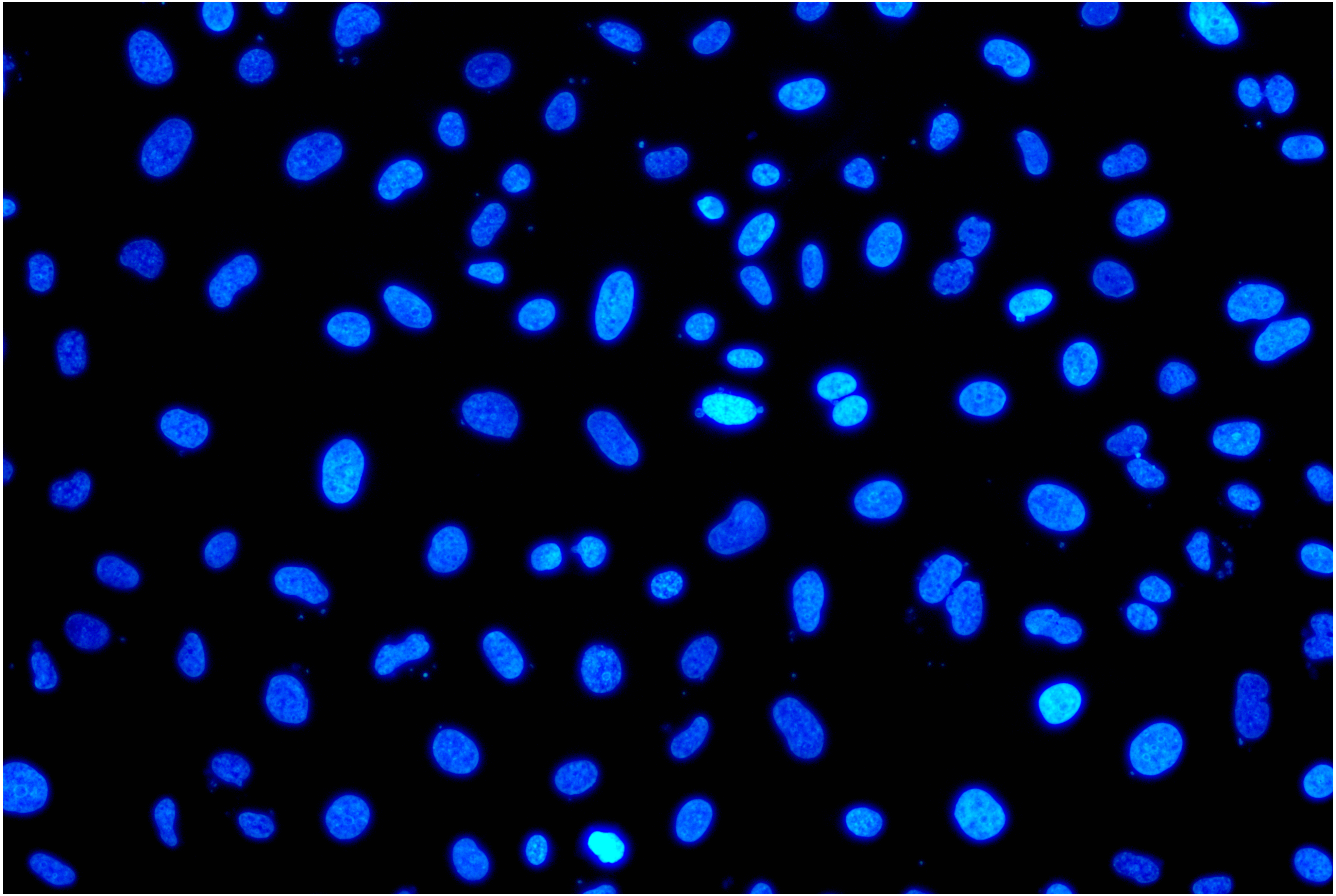

HCCLM3 Si-lnc DAPI

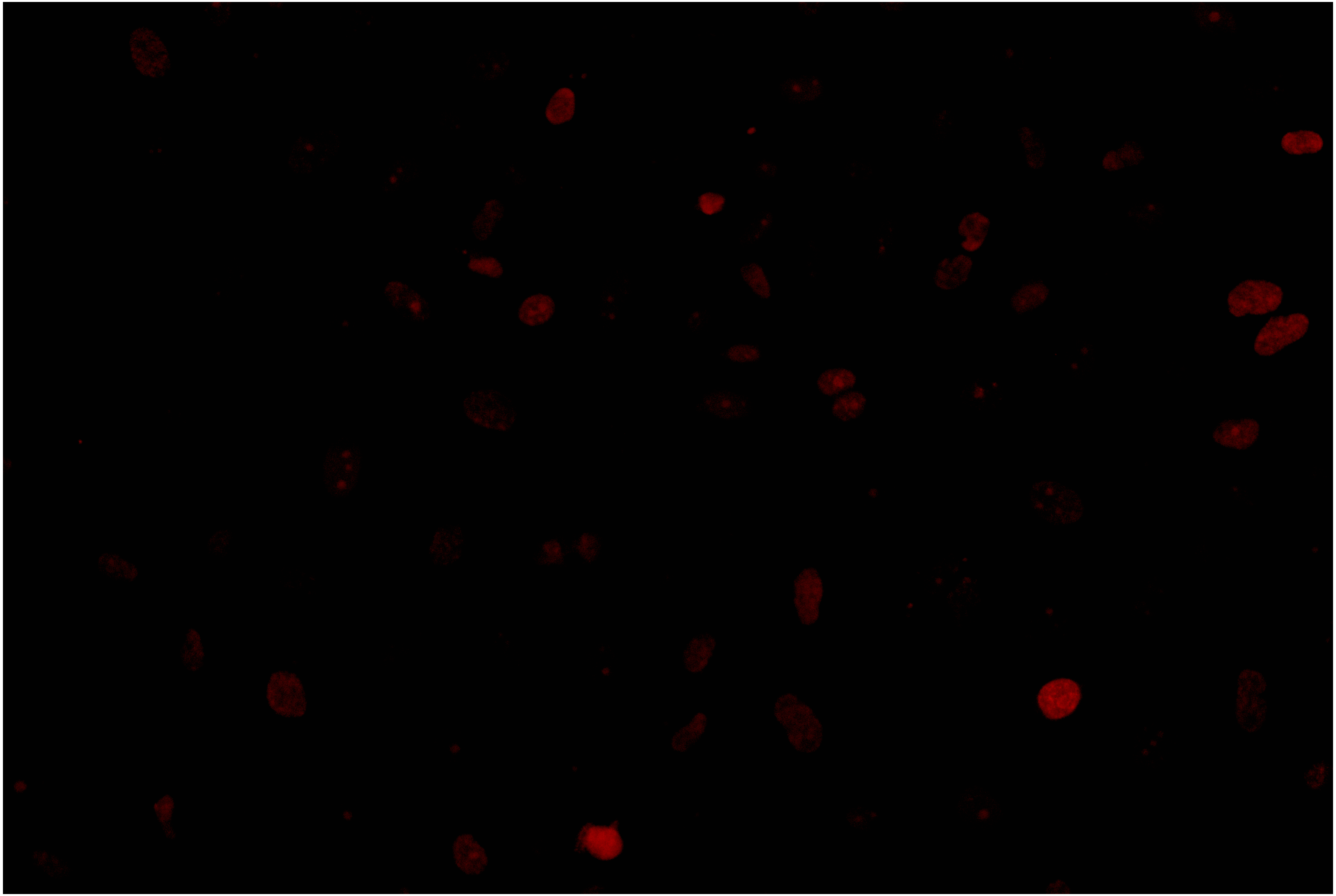

HCCLM3 Si-lnc EDU

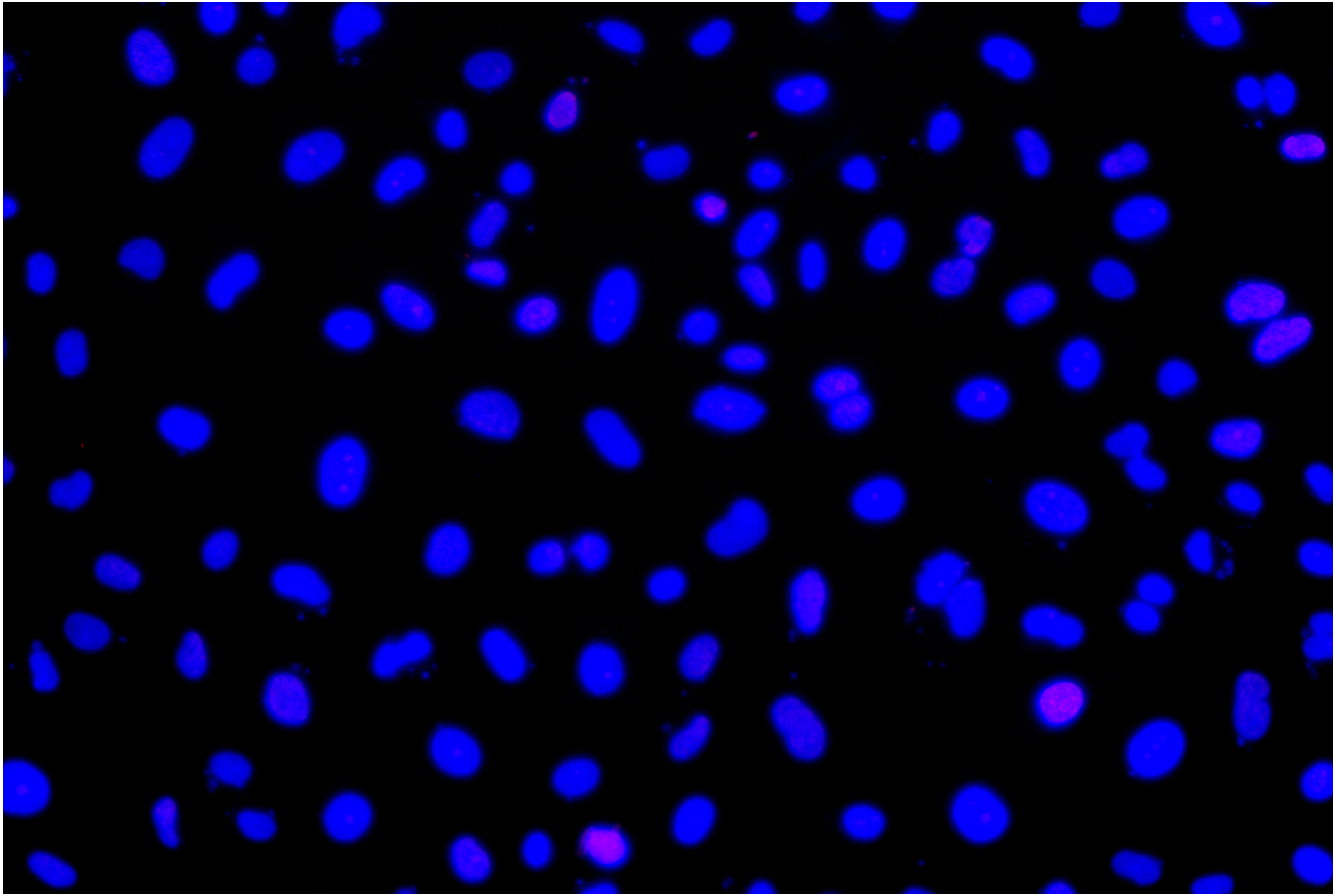

HCCLM3 Si-lnc Merge

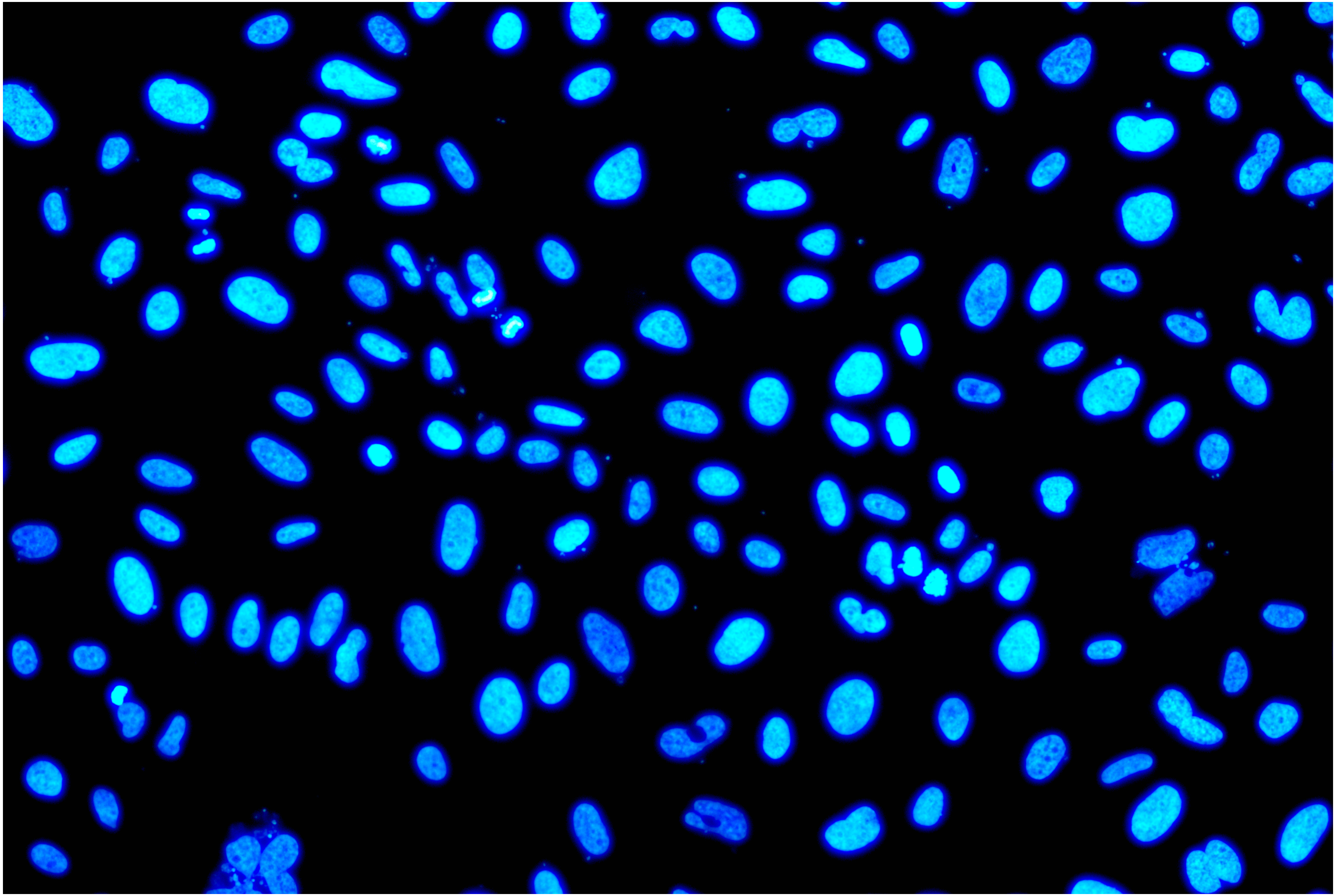

HCCLM3 si-NC DAPI

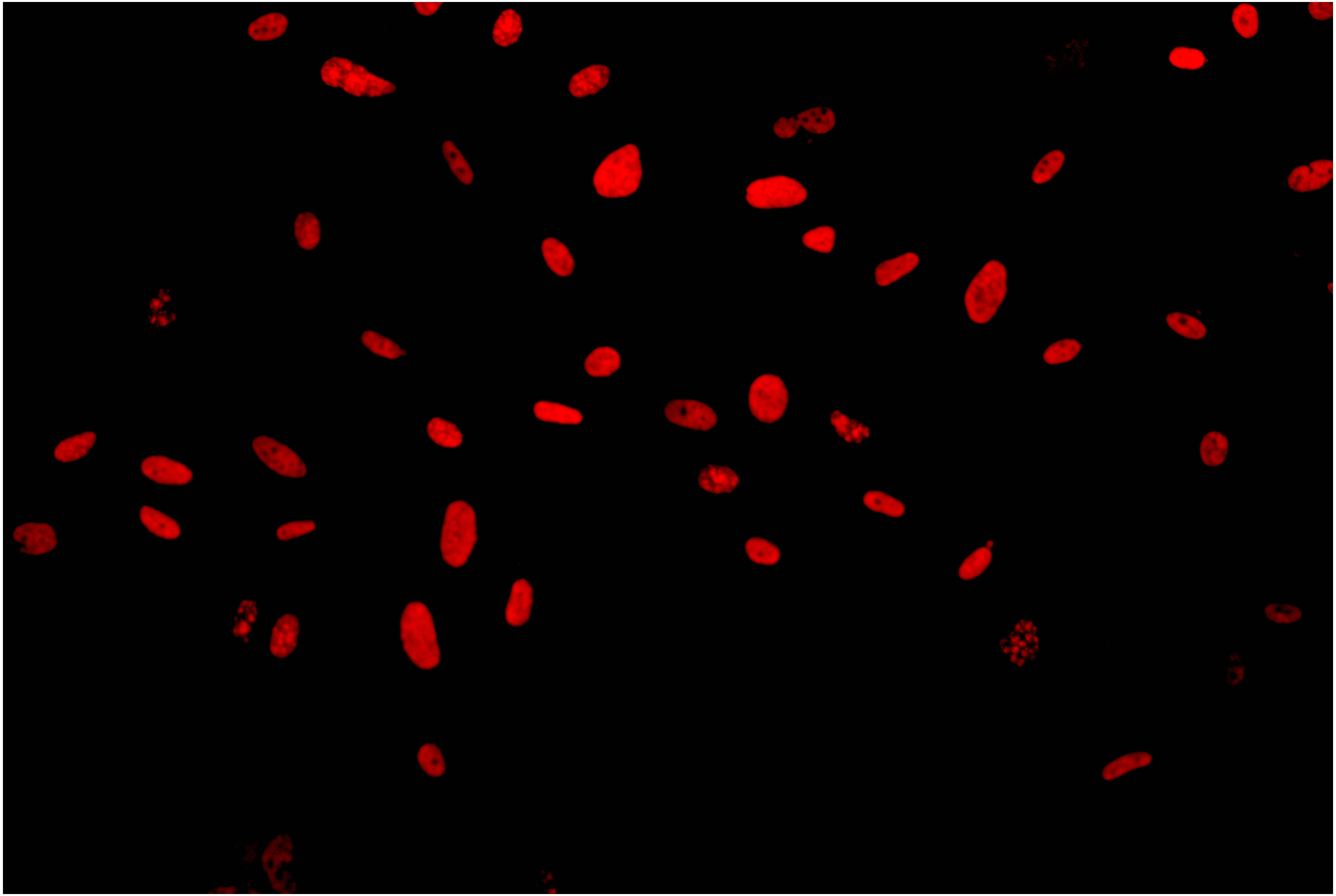

HCCLM3 si-NC EDU

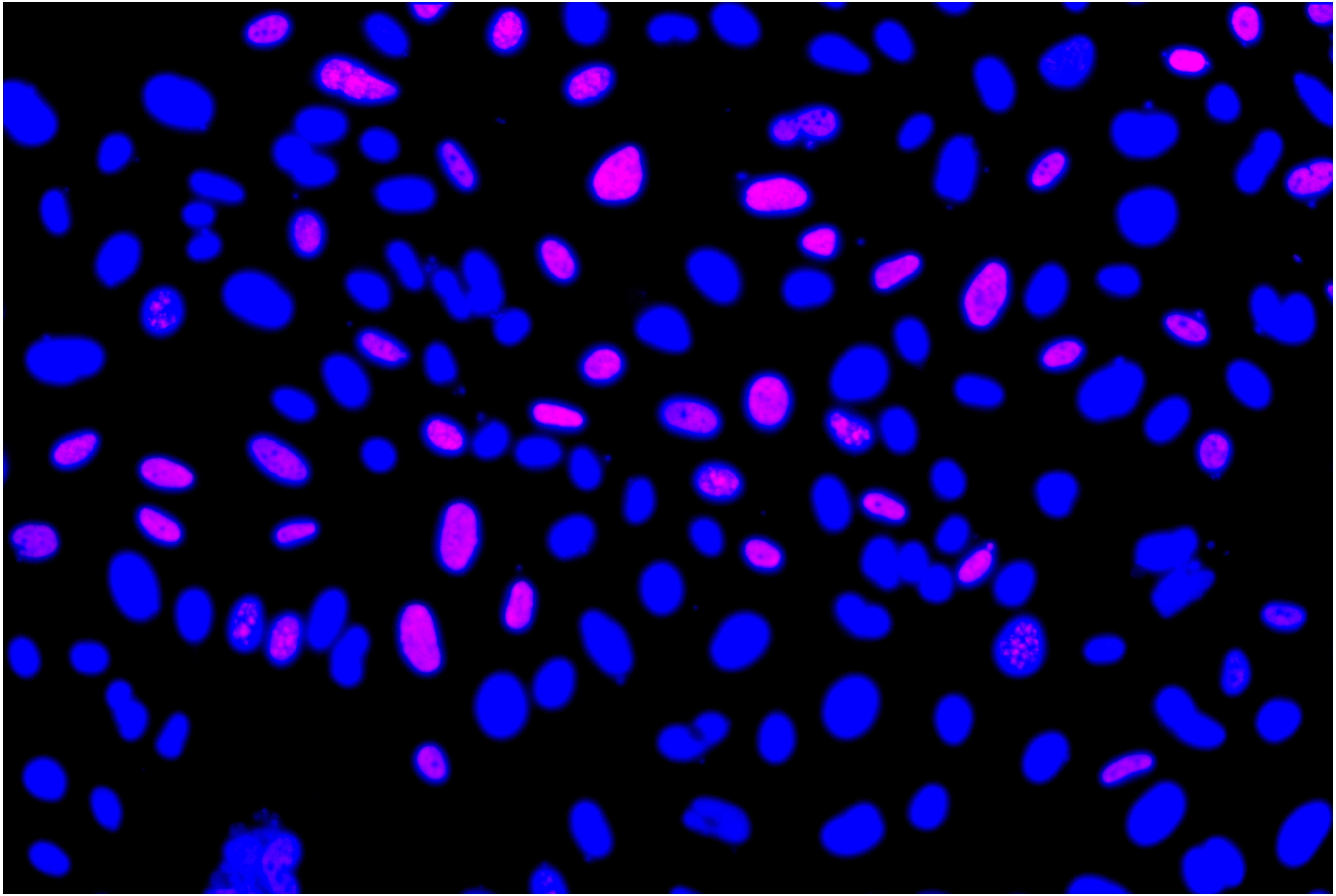

HCCLM3 si-NC Merge

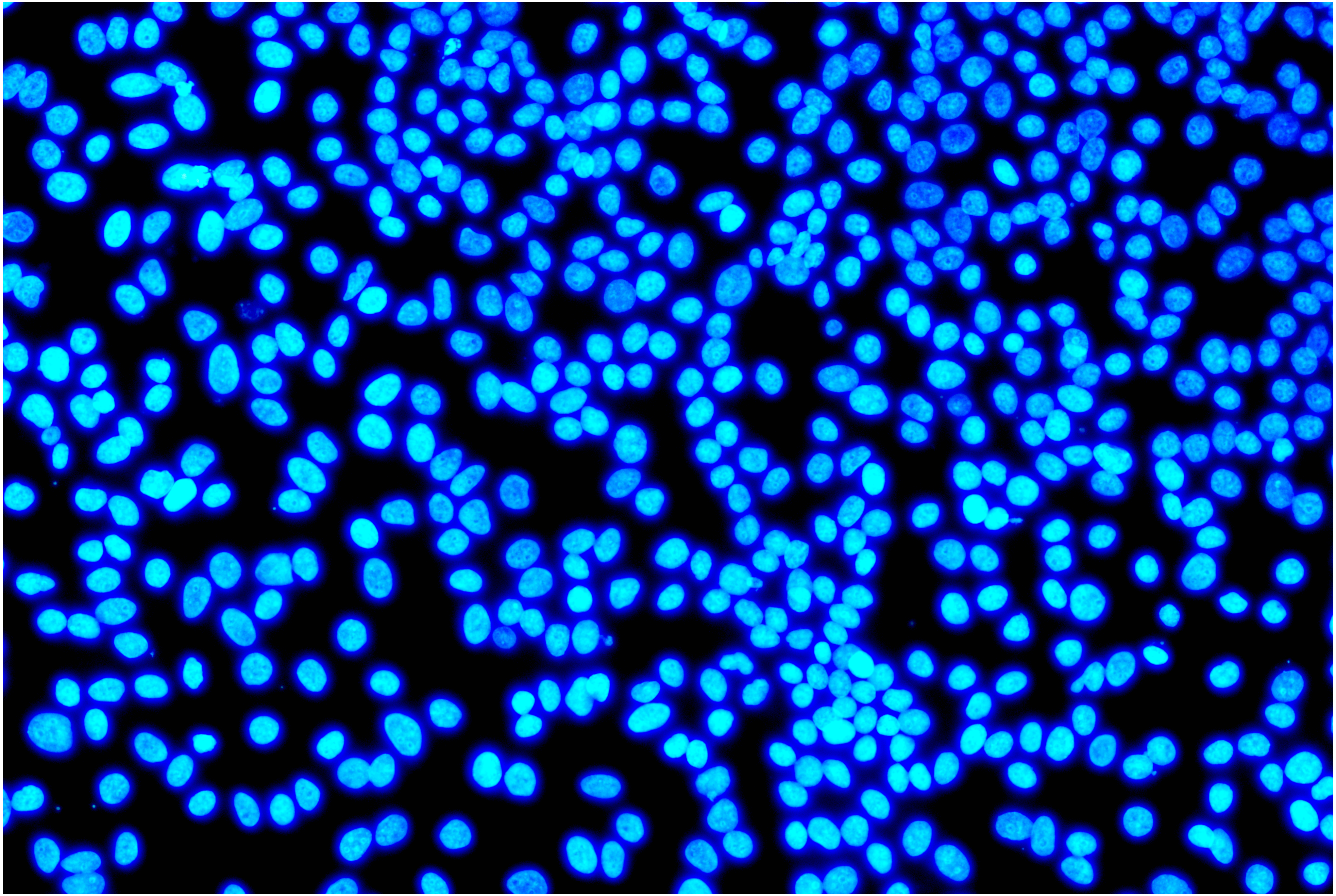

Huh7 blank DAPI

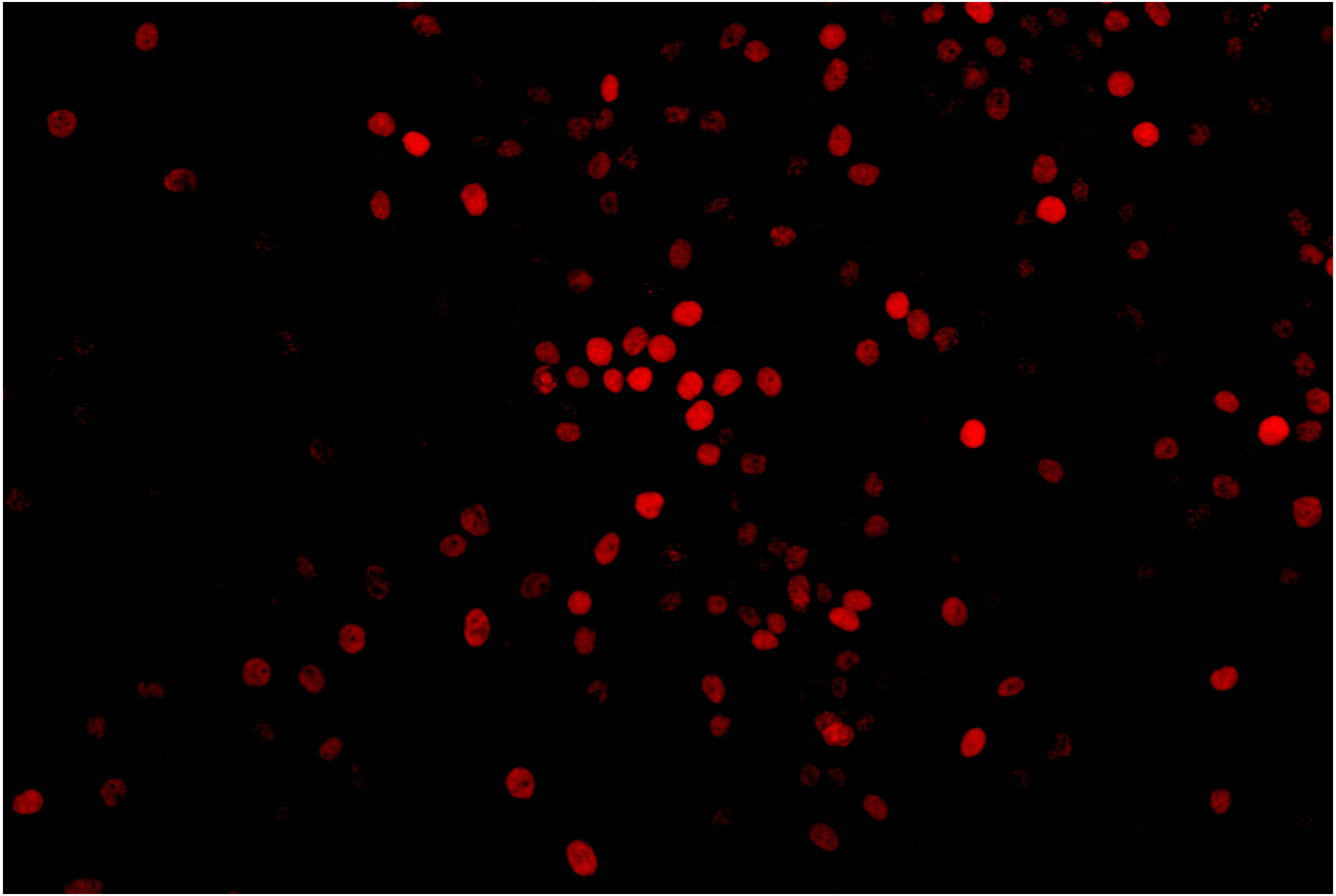

Huh7 blank EDU

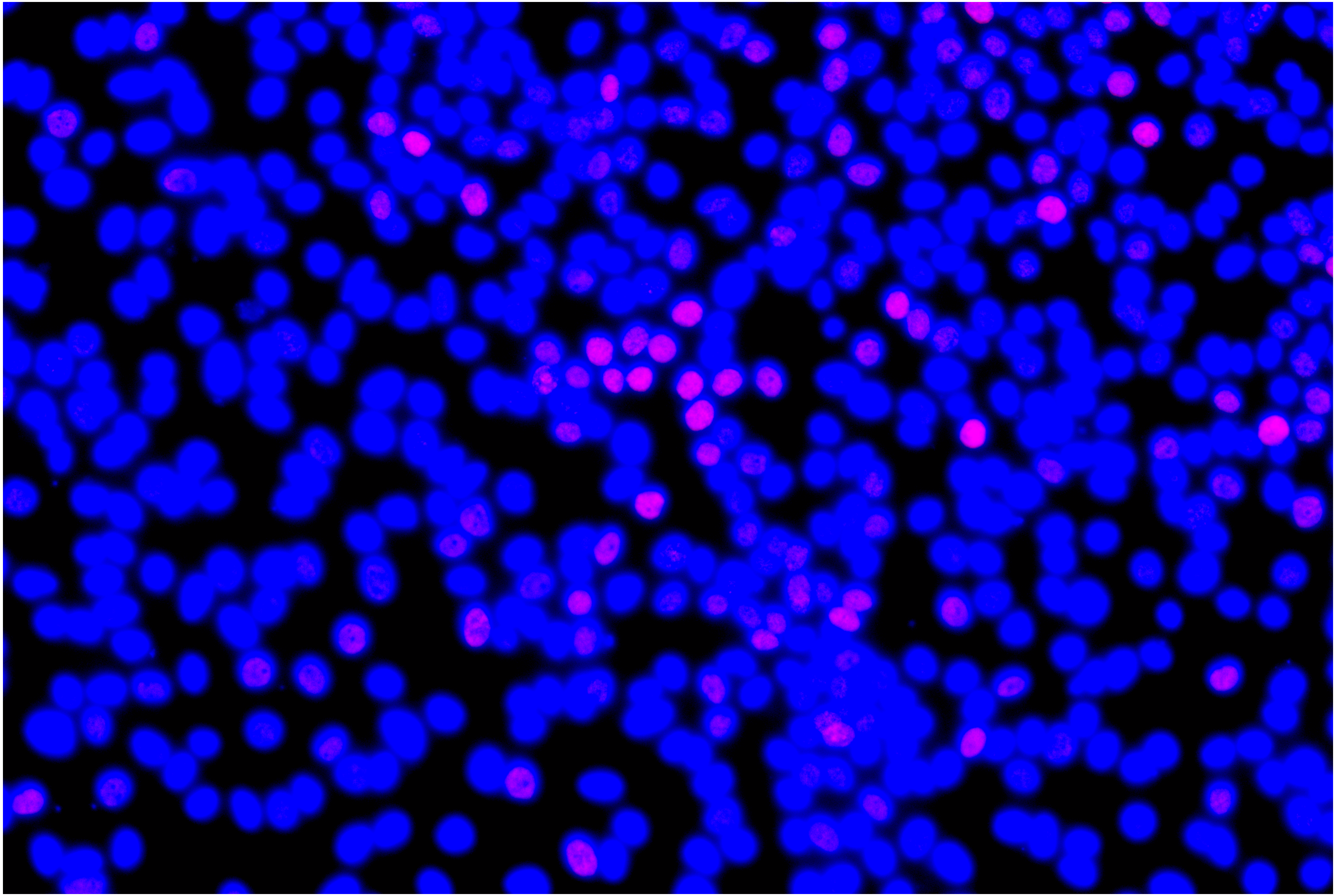

Huh7 blank Merge

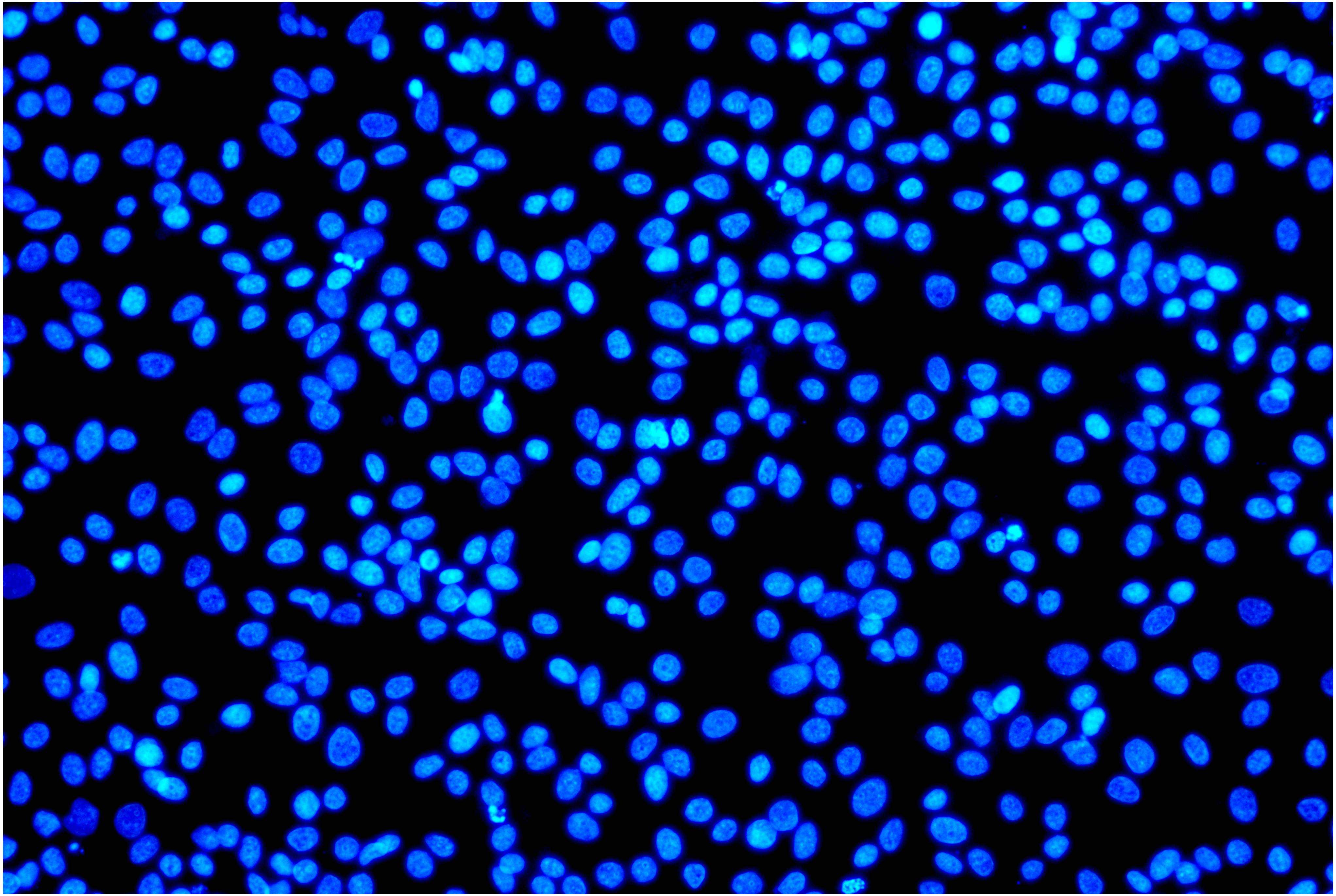

Huh7 Si-lnc DAPI

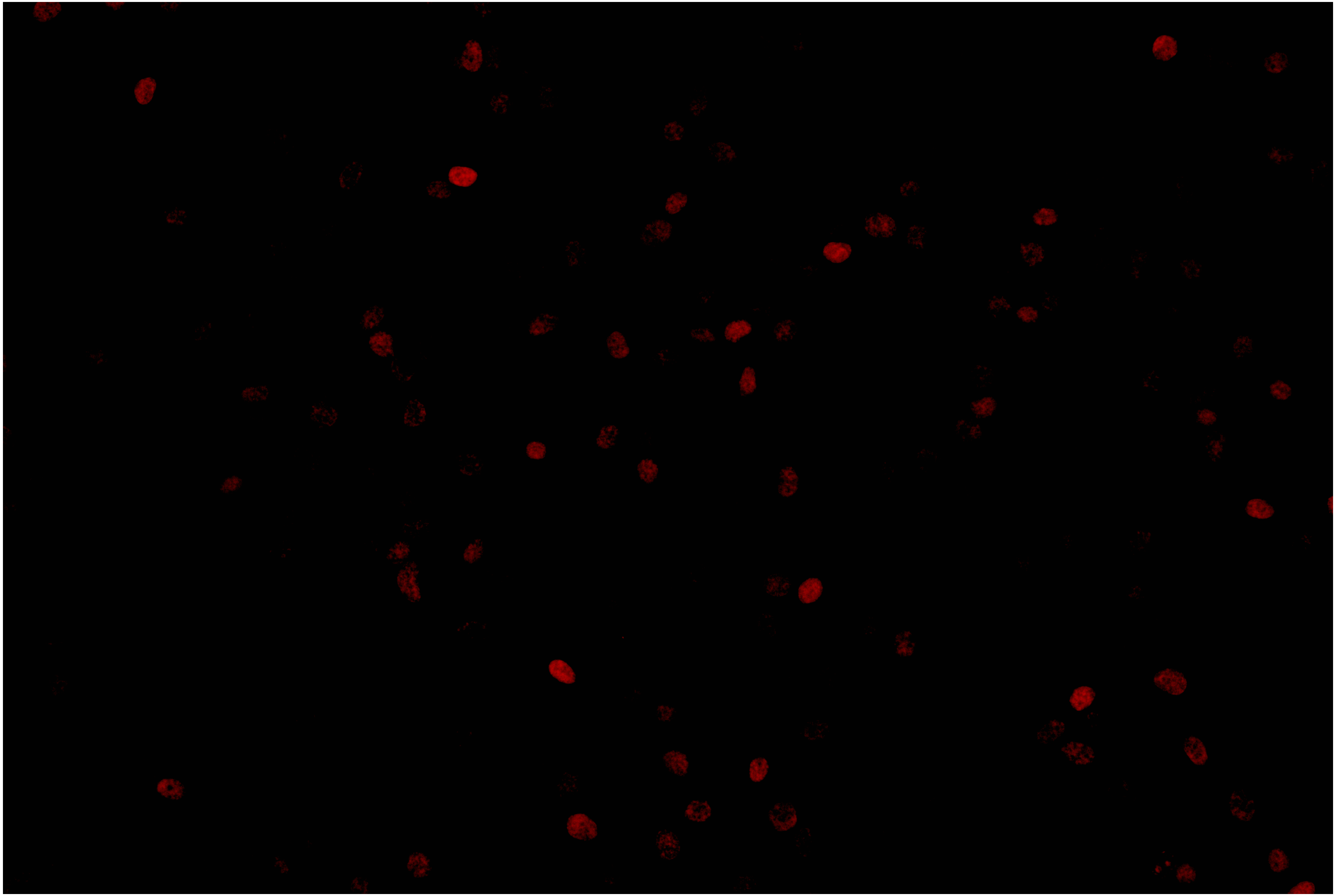

Huh7 Si-lnc EDU

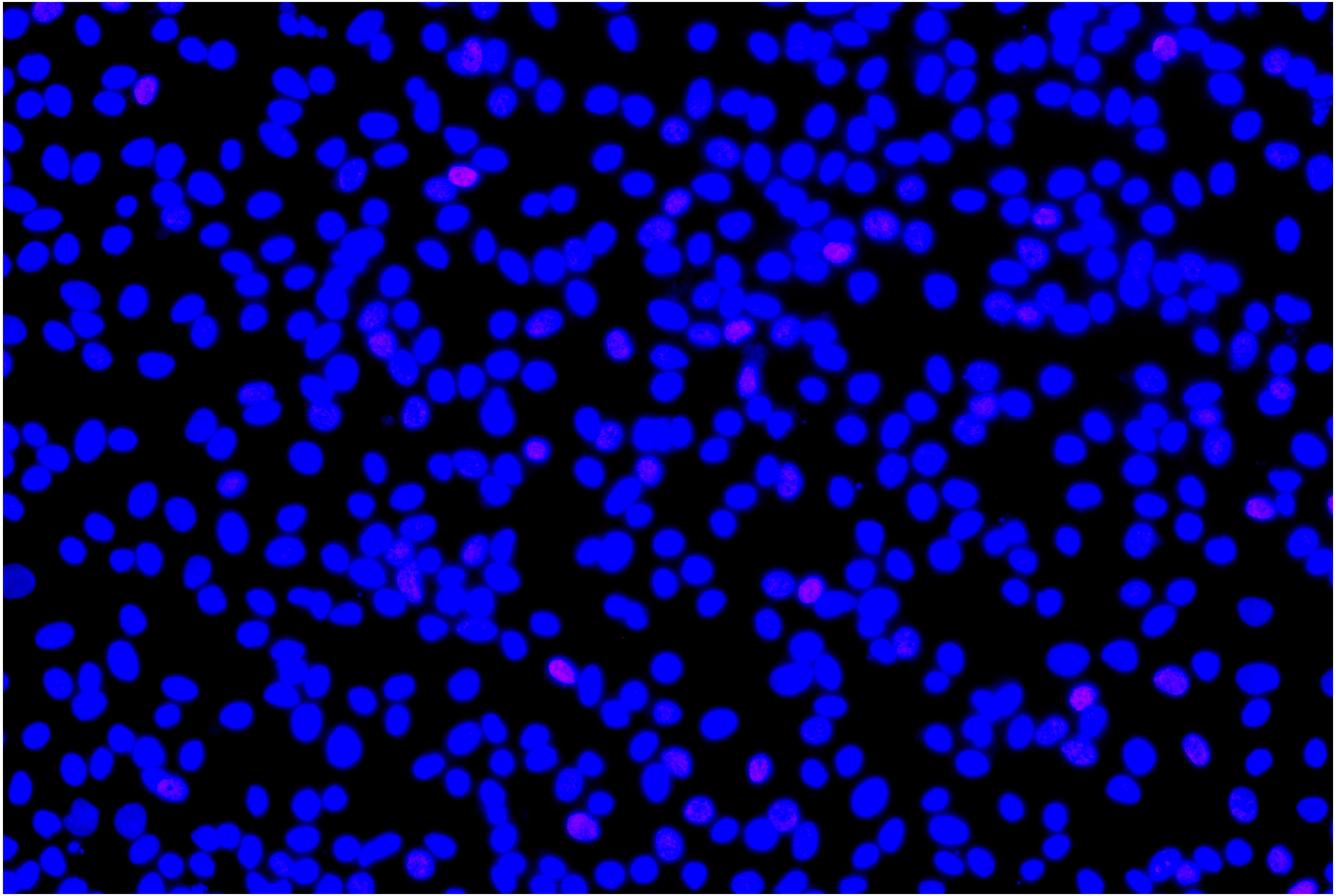

Huh7 Si-lnc Merge

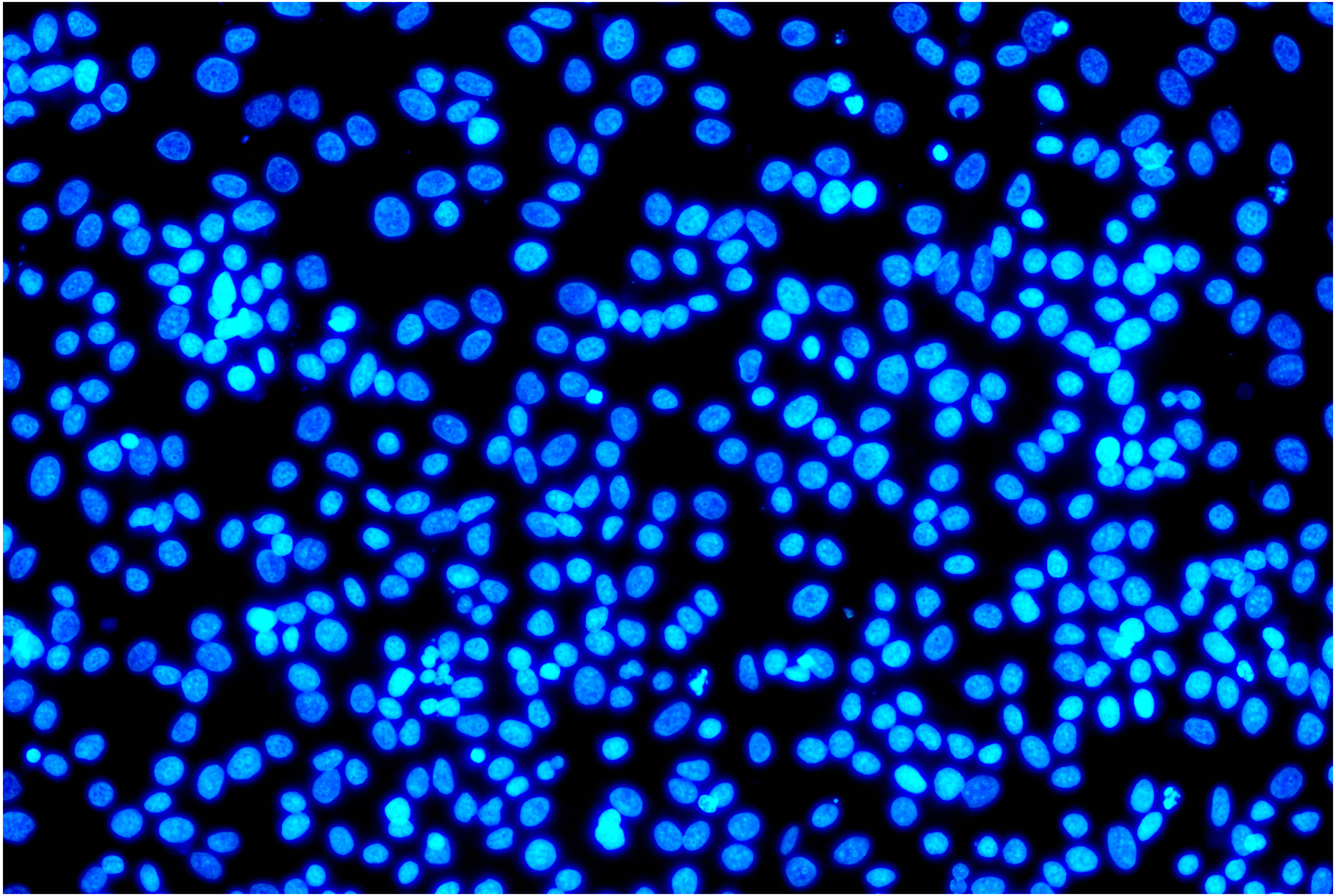

Huh7 si-NC DAPI

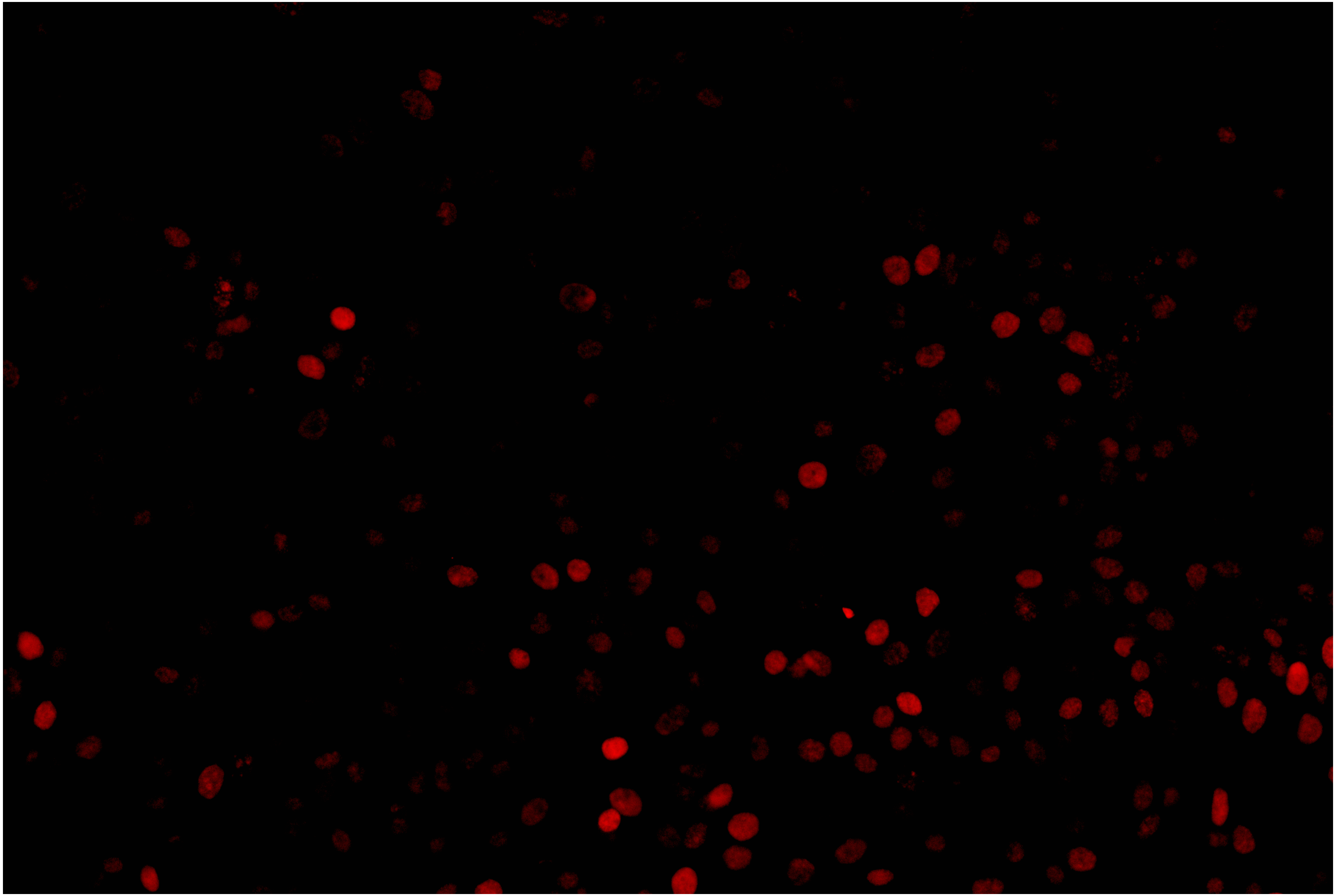

Huh7 si-NC EDU

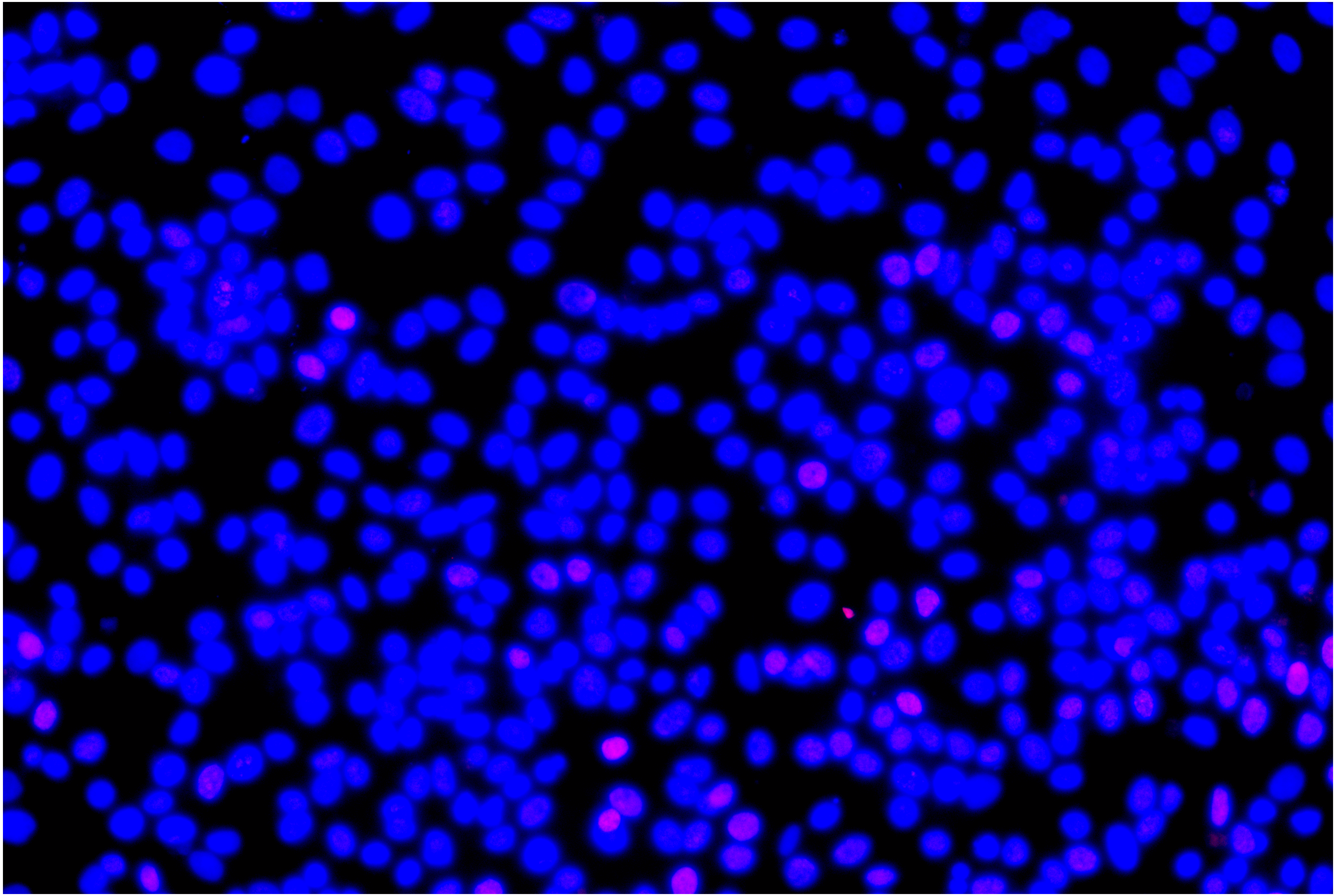

Huh7 si-NC Merge

Figure 6B

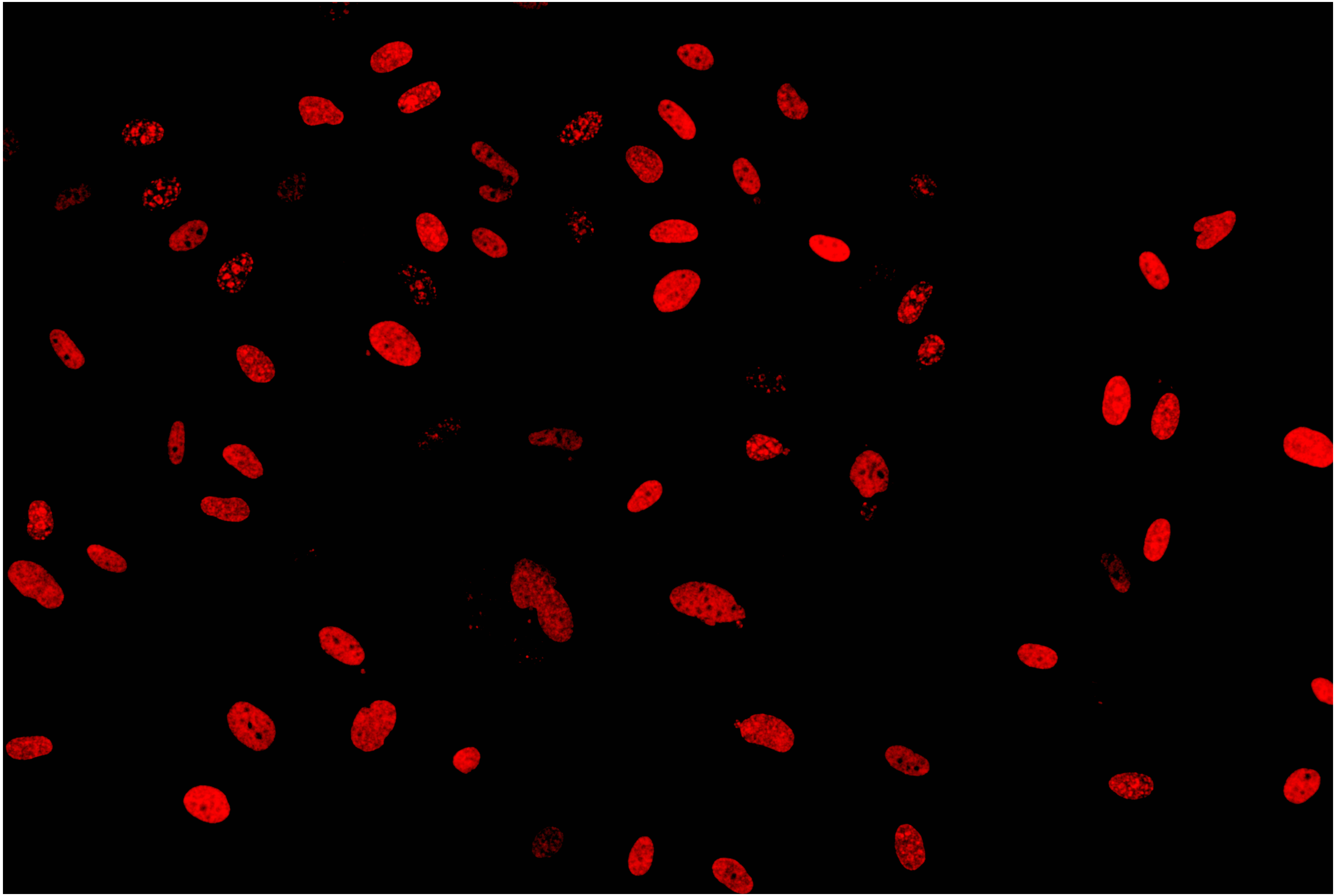

HCCLM3 anti-miR+si-NC EDU

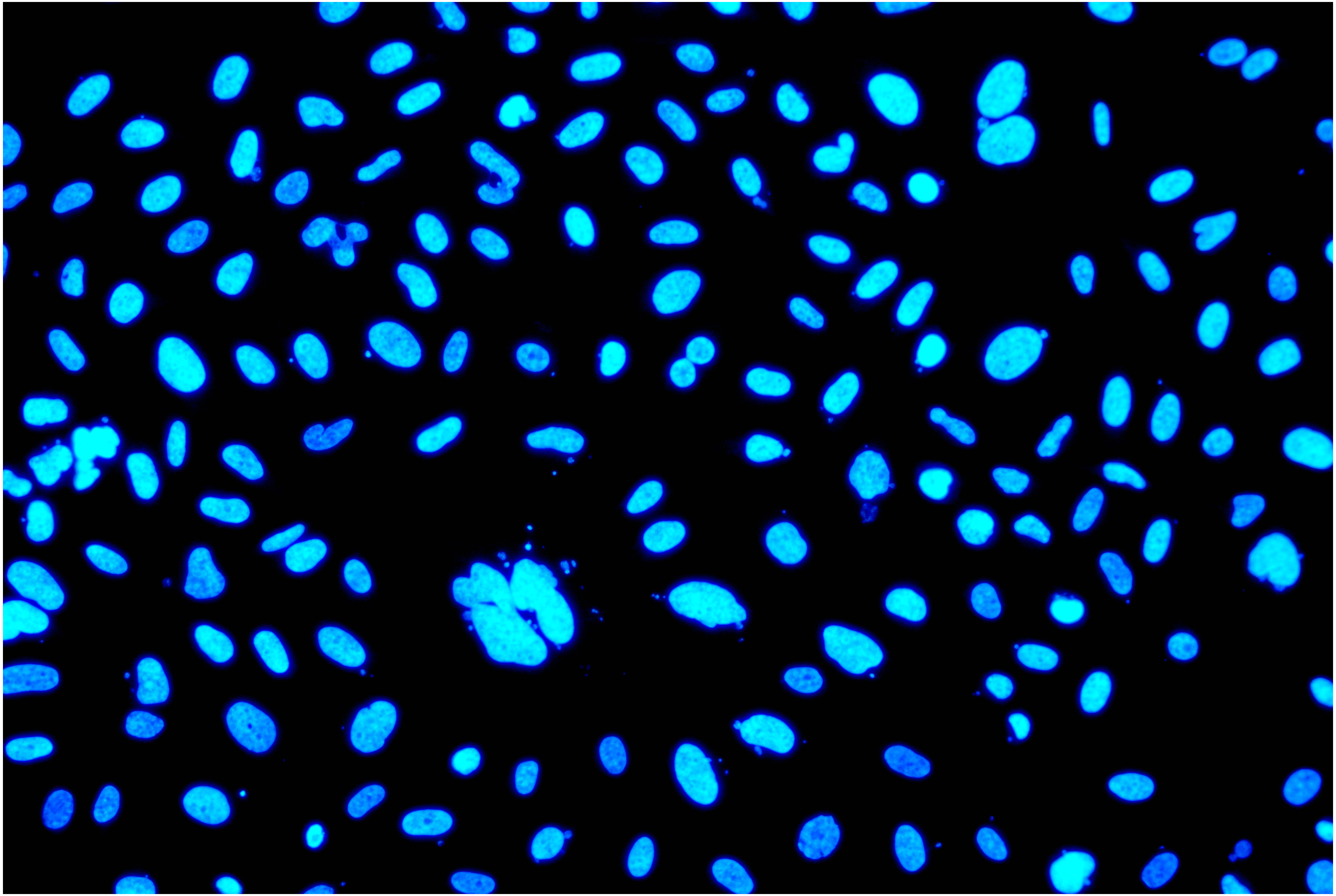

HCCLM3 anti-miR+si-NC DAPI

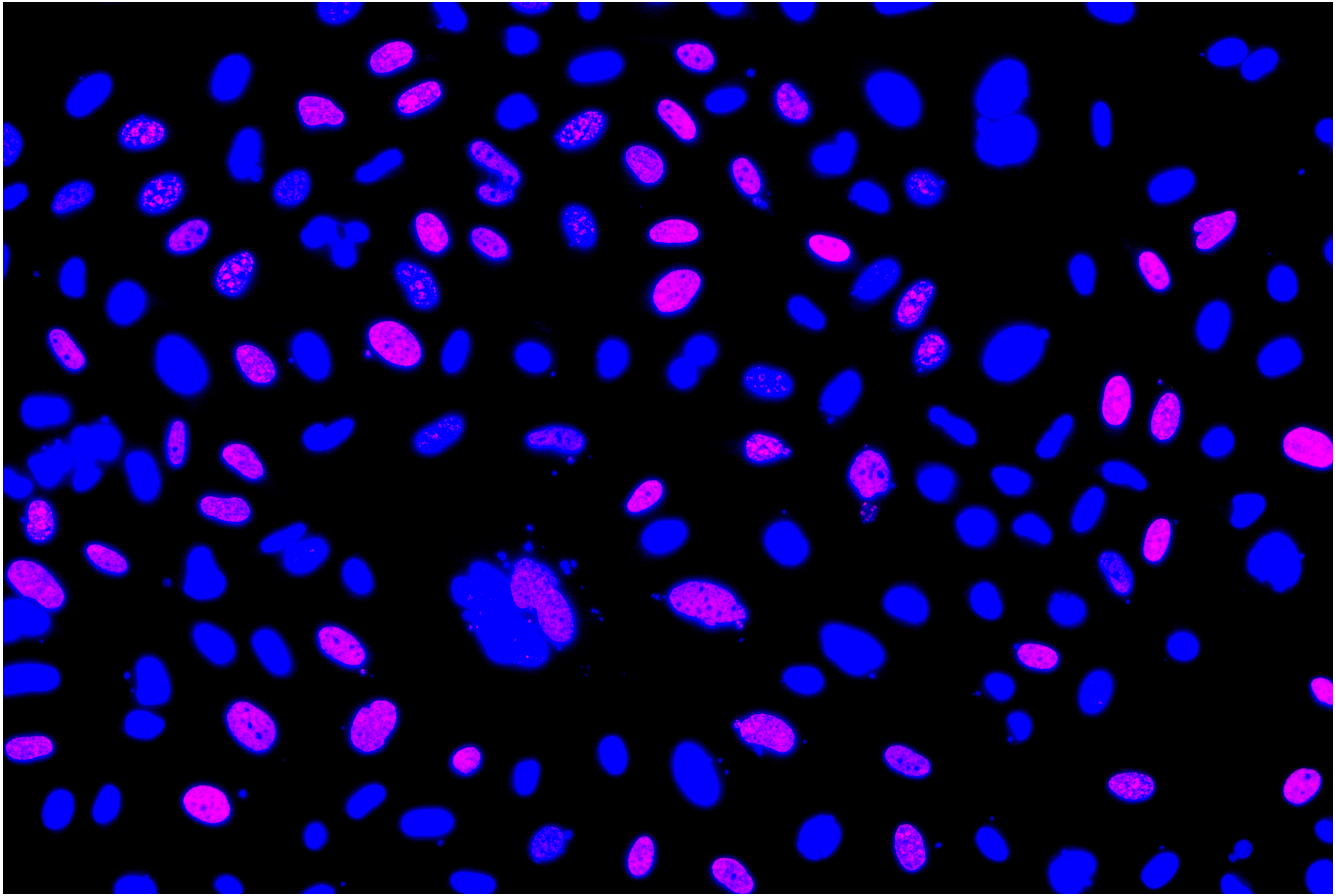

HCCLM3 anti-miR+si-NC Merge

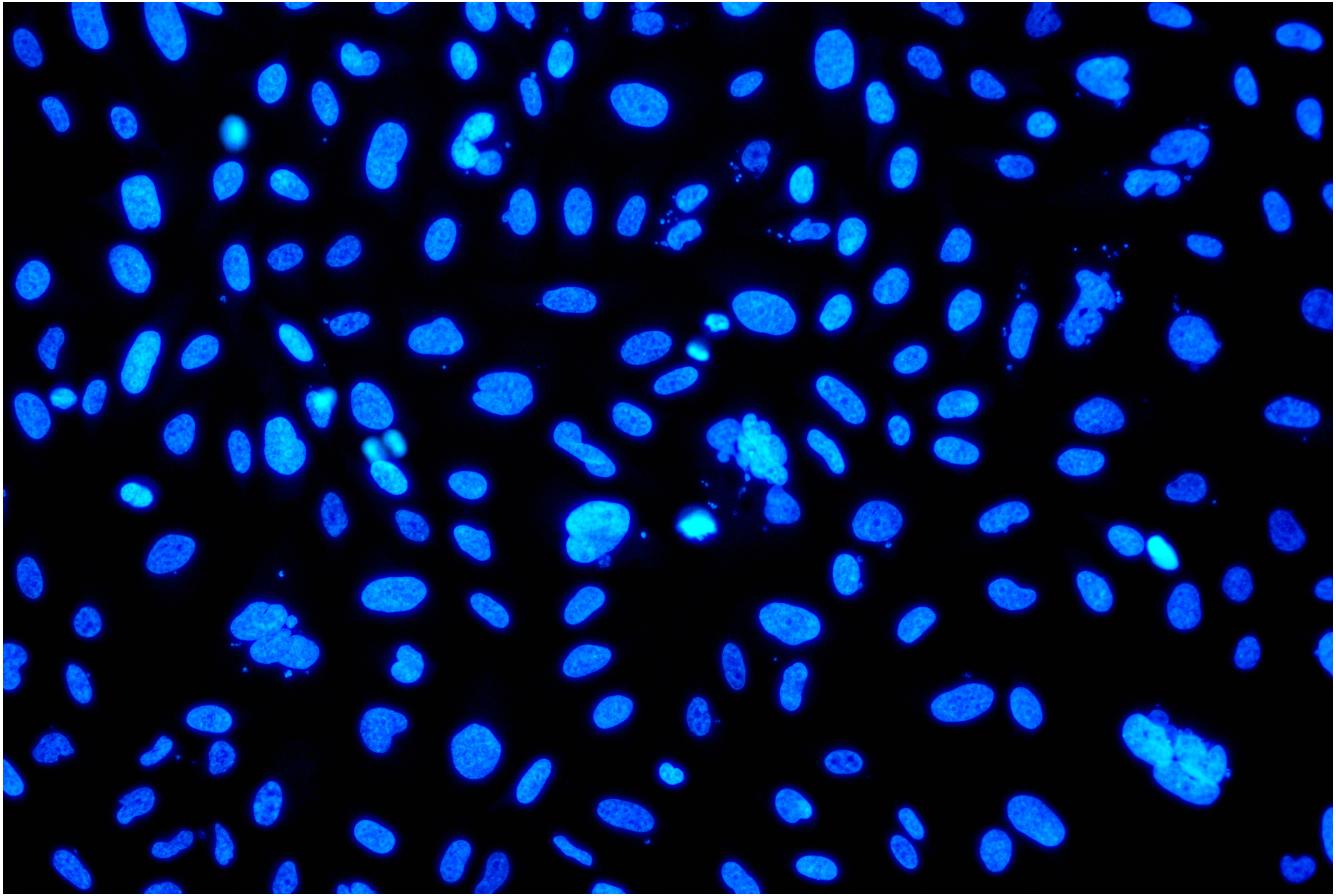

HCCLM3 blank DAPI

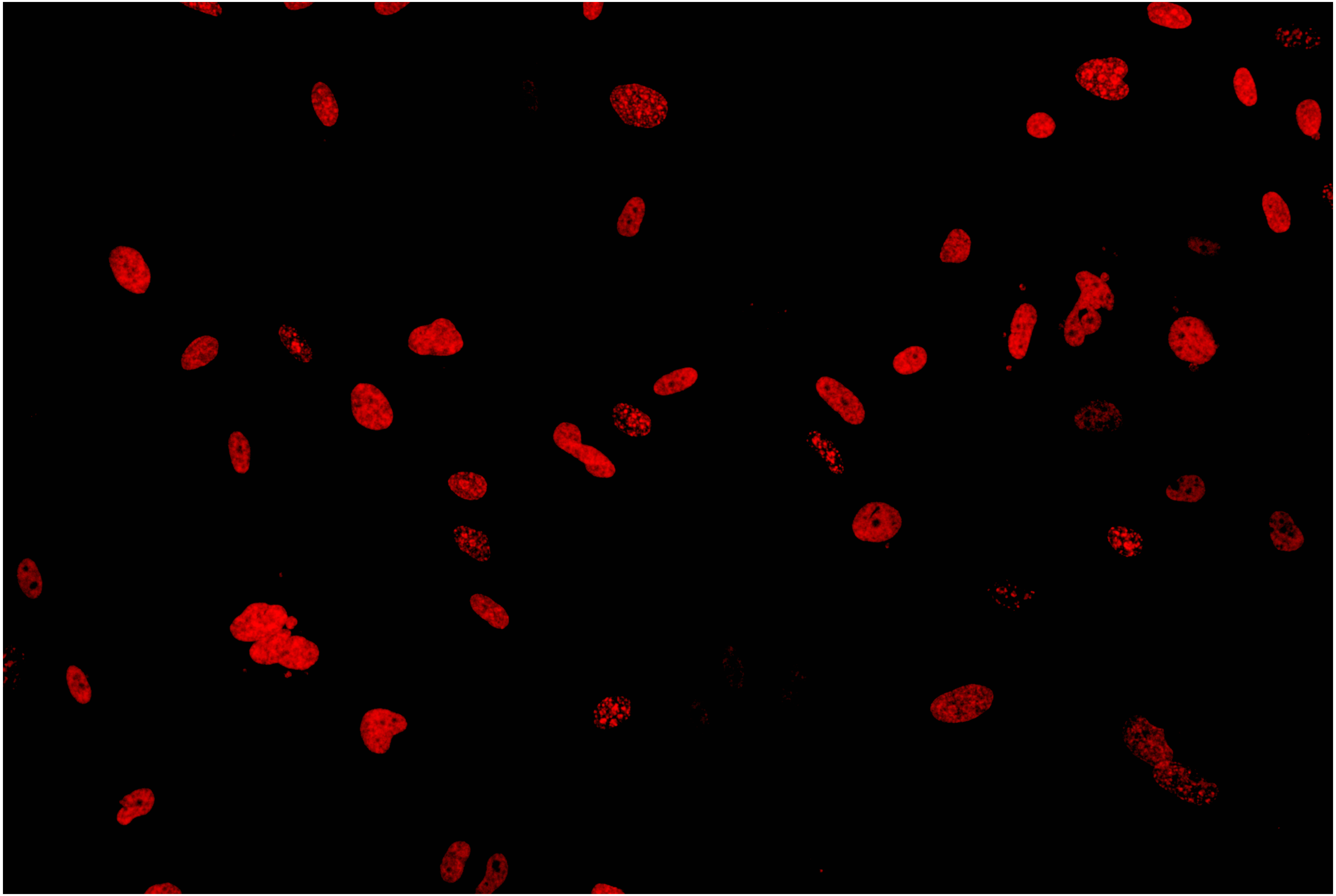

HCCLM3 blank EDU

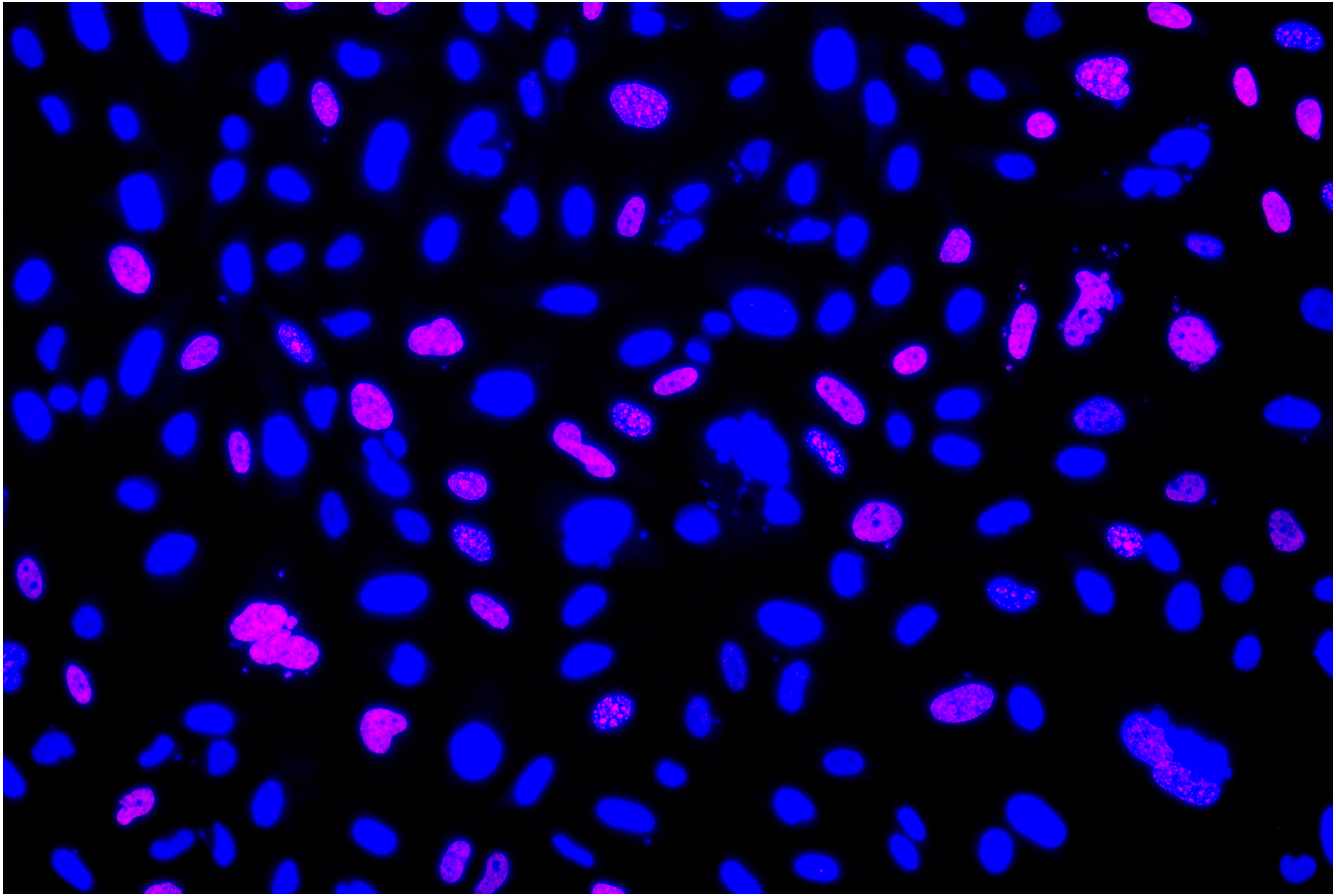

HCCLM3 blank Merge

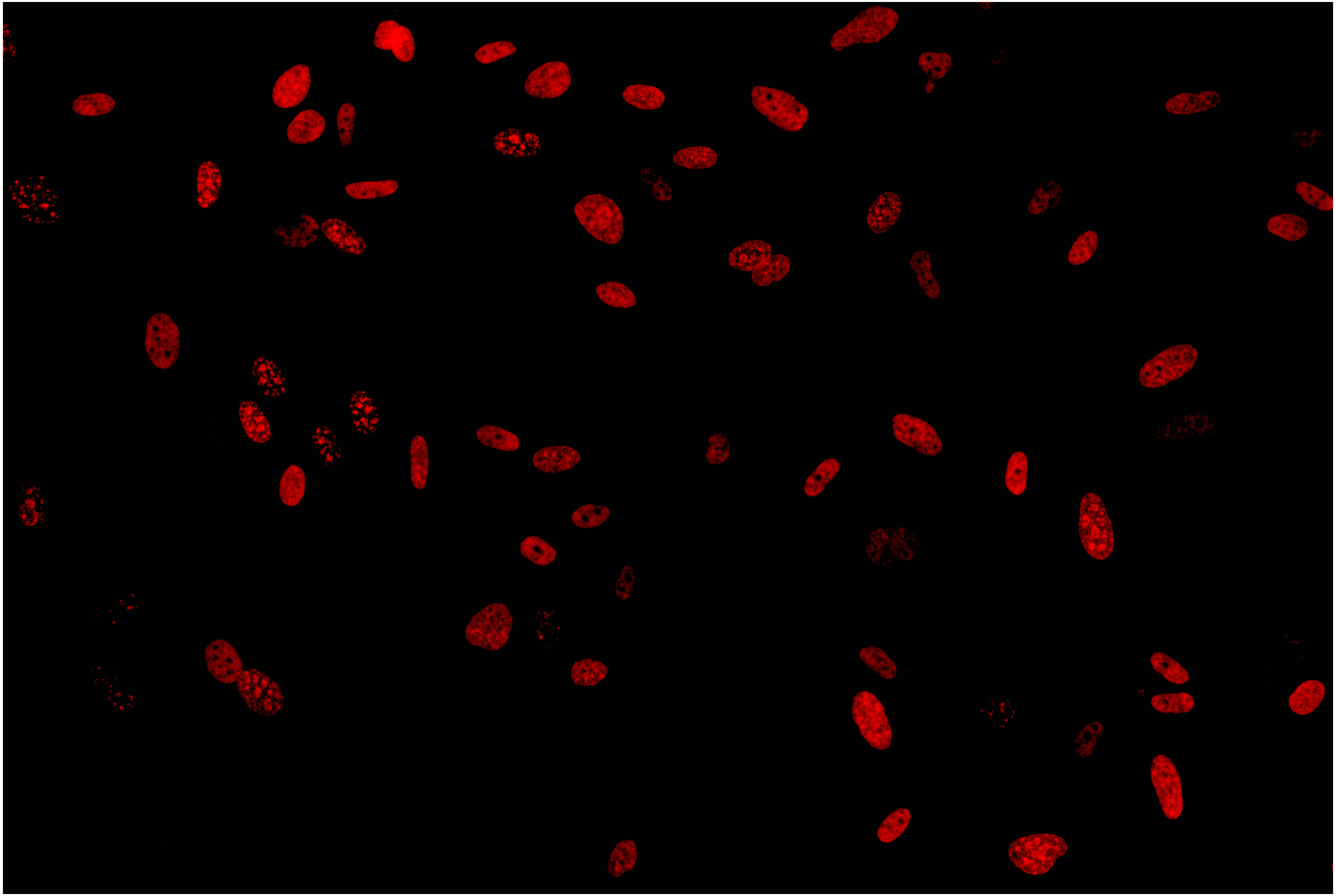

HCCLM3 Si-lnc+anti-miR EDU

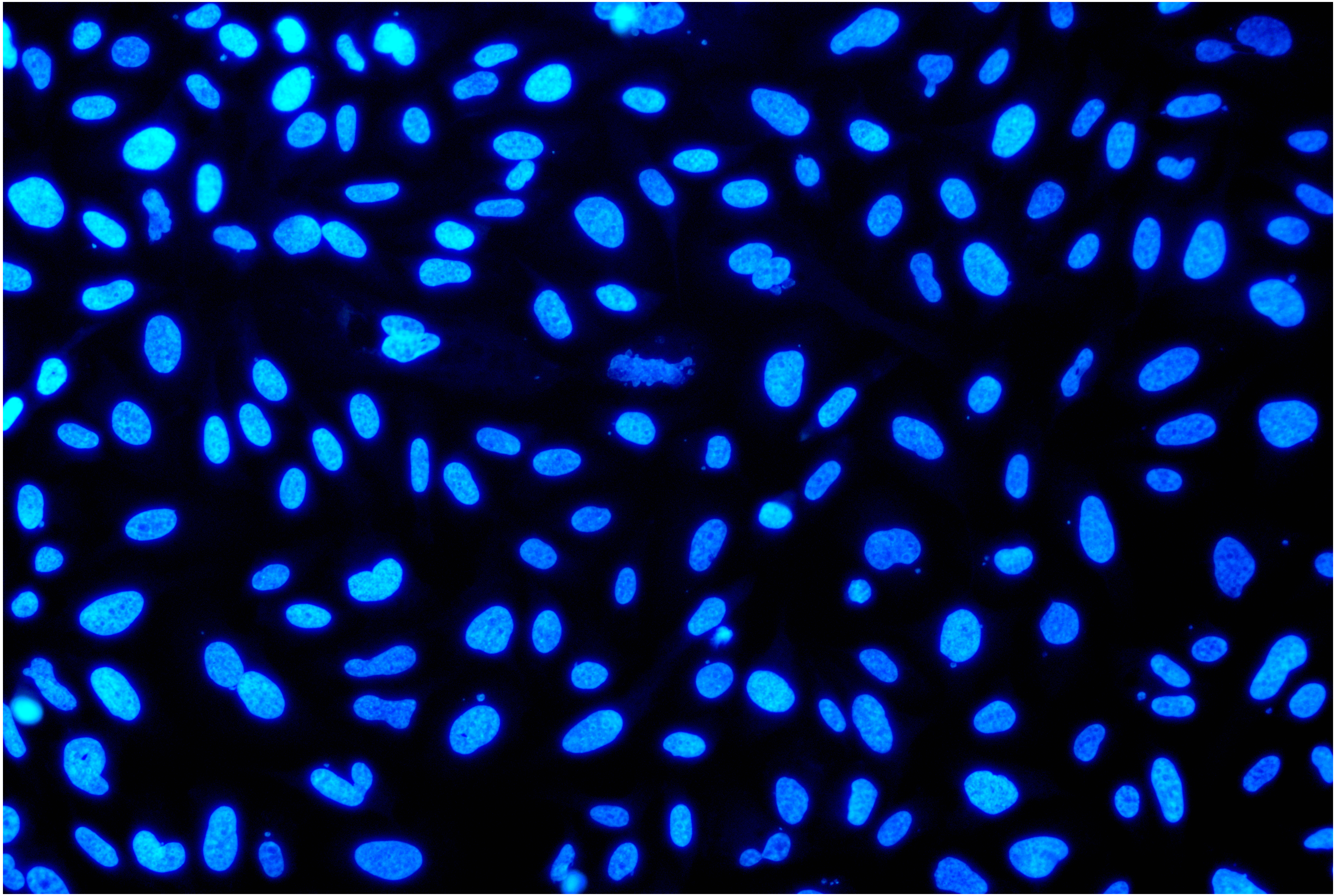

HCCLM3 Si-lnc+anti-miR DAPI

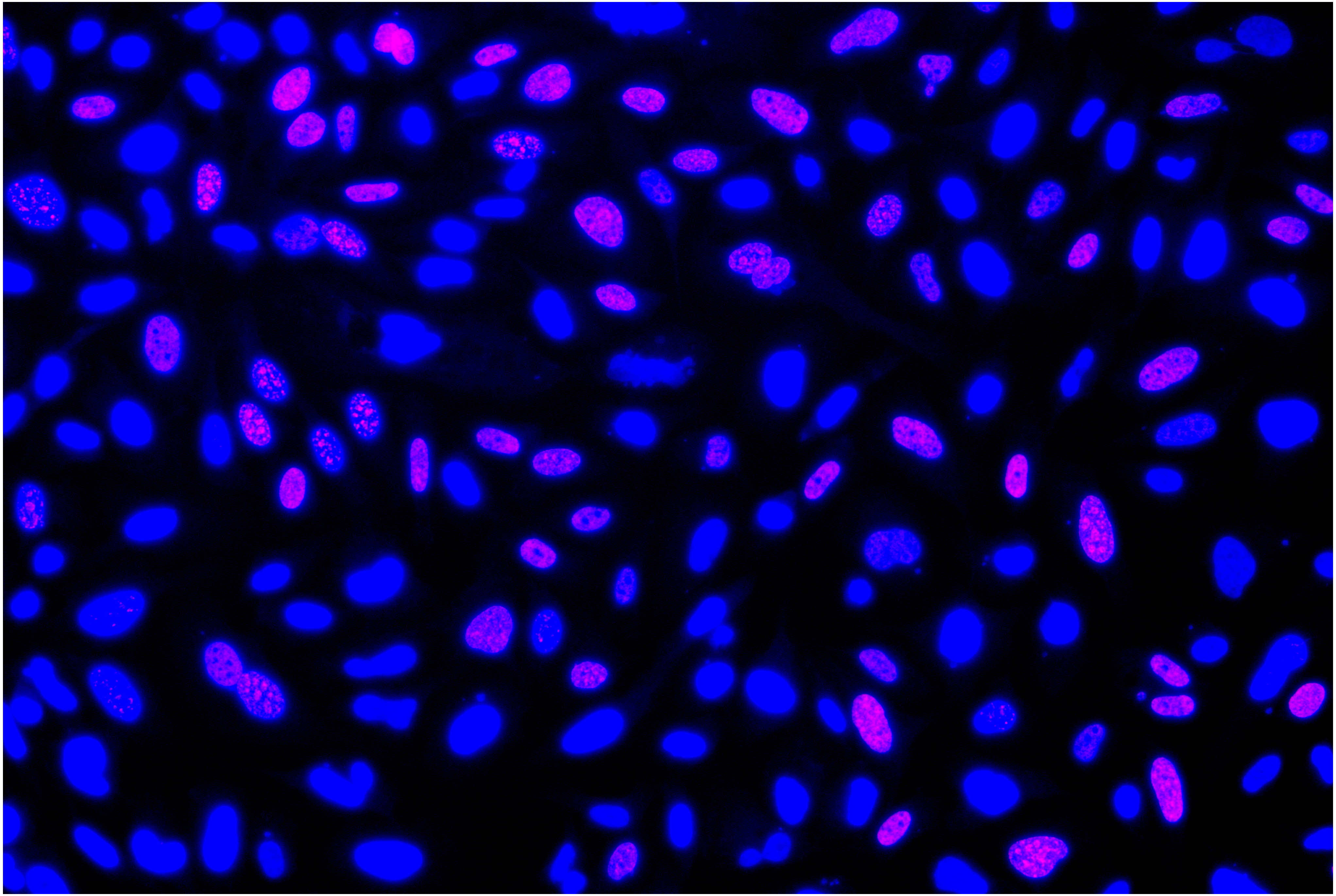

HCCLM3 Si-lnc+anti-miR Merge

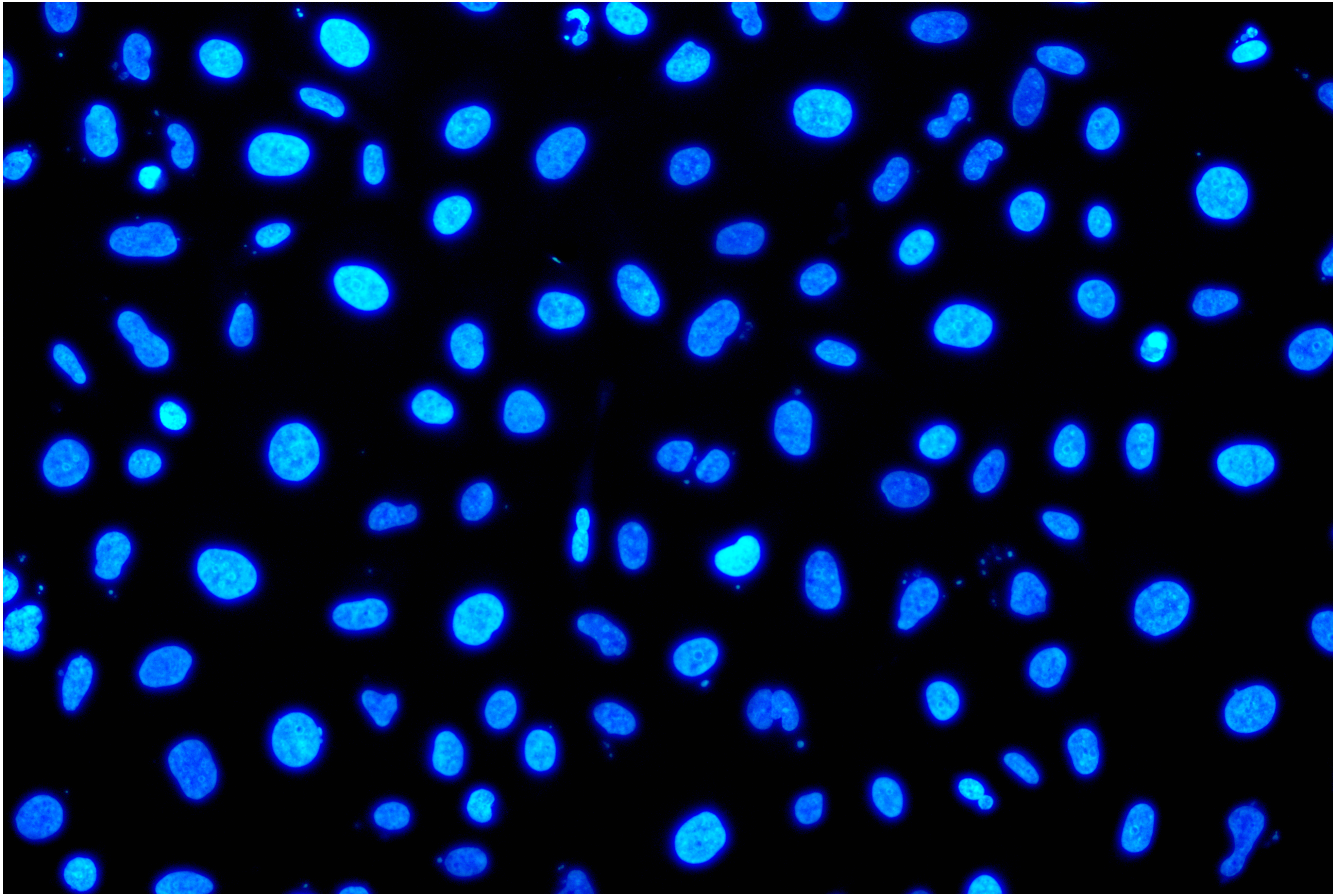

HCCLM3 Si-lnc+anti-miR-NC DAPI

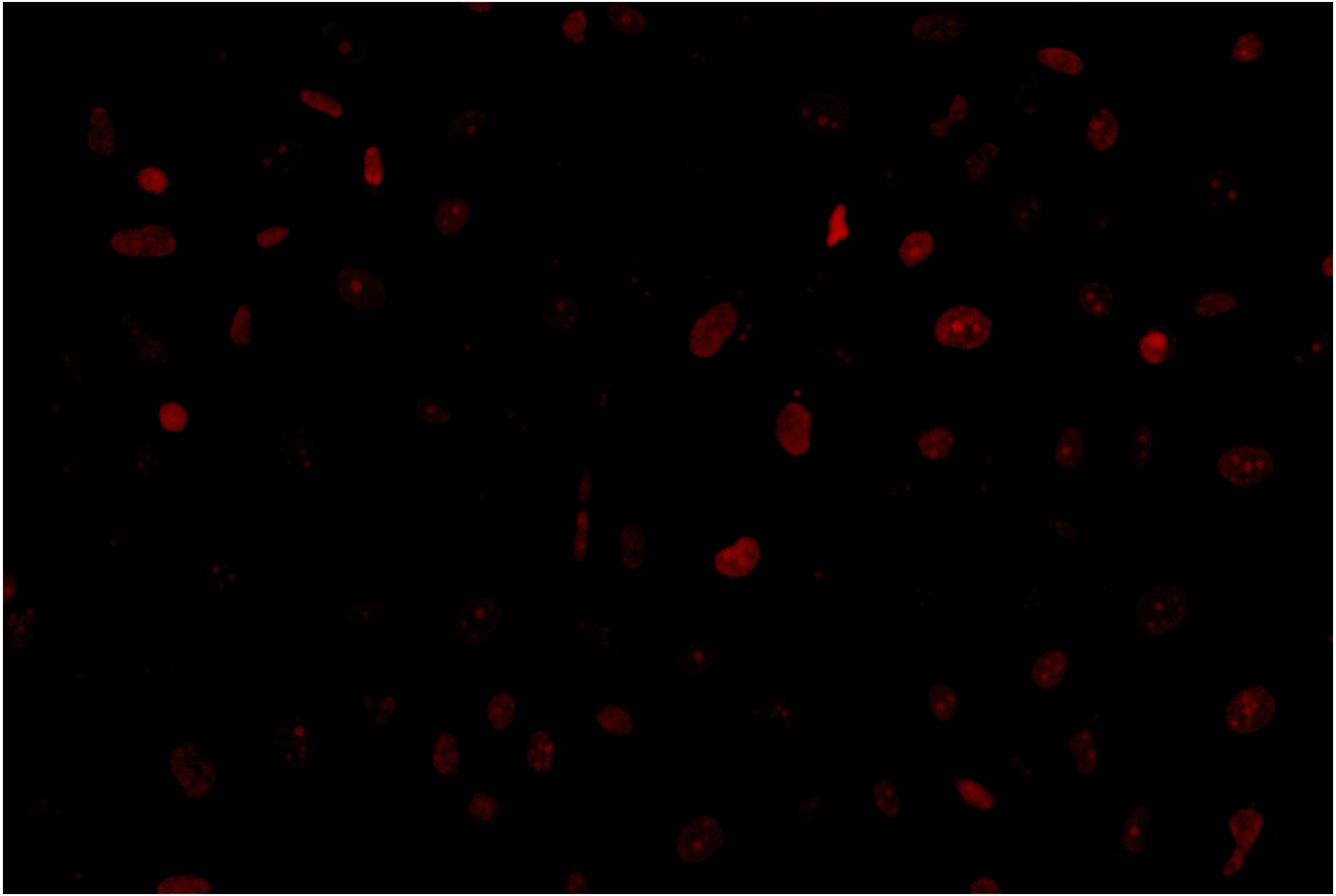

HCCLM3 Si-lnc+anti-miR-NC EDU

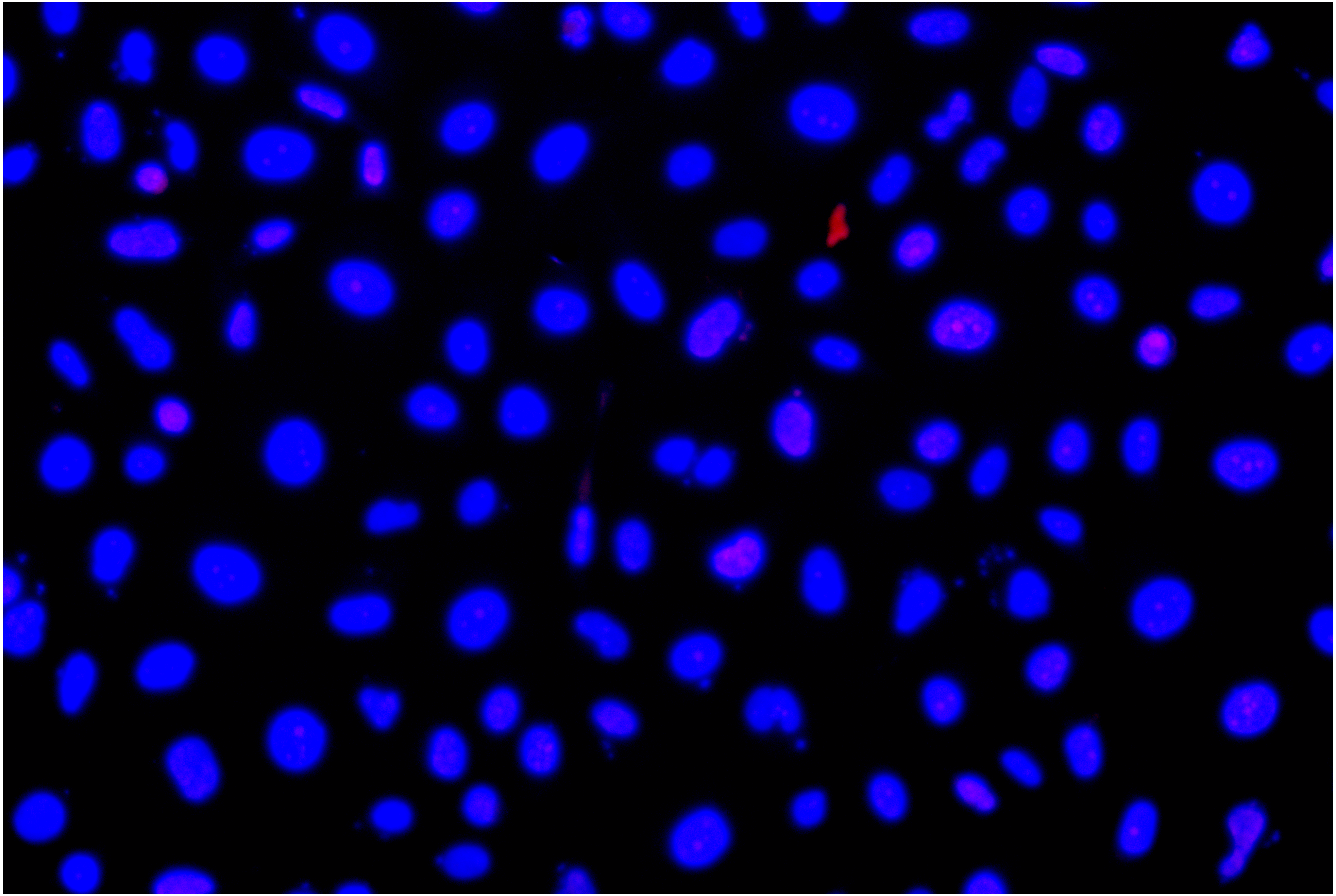

HCCLM3 Si-lnc+anti-miR-NC Merge

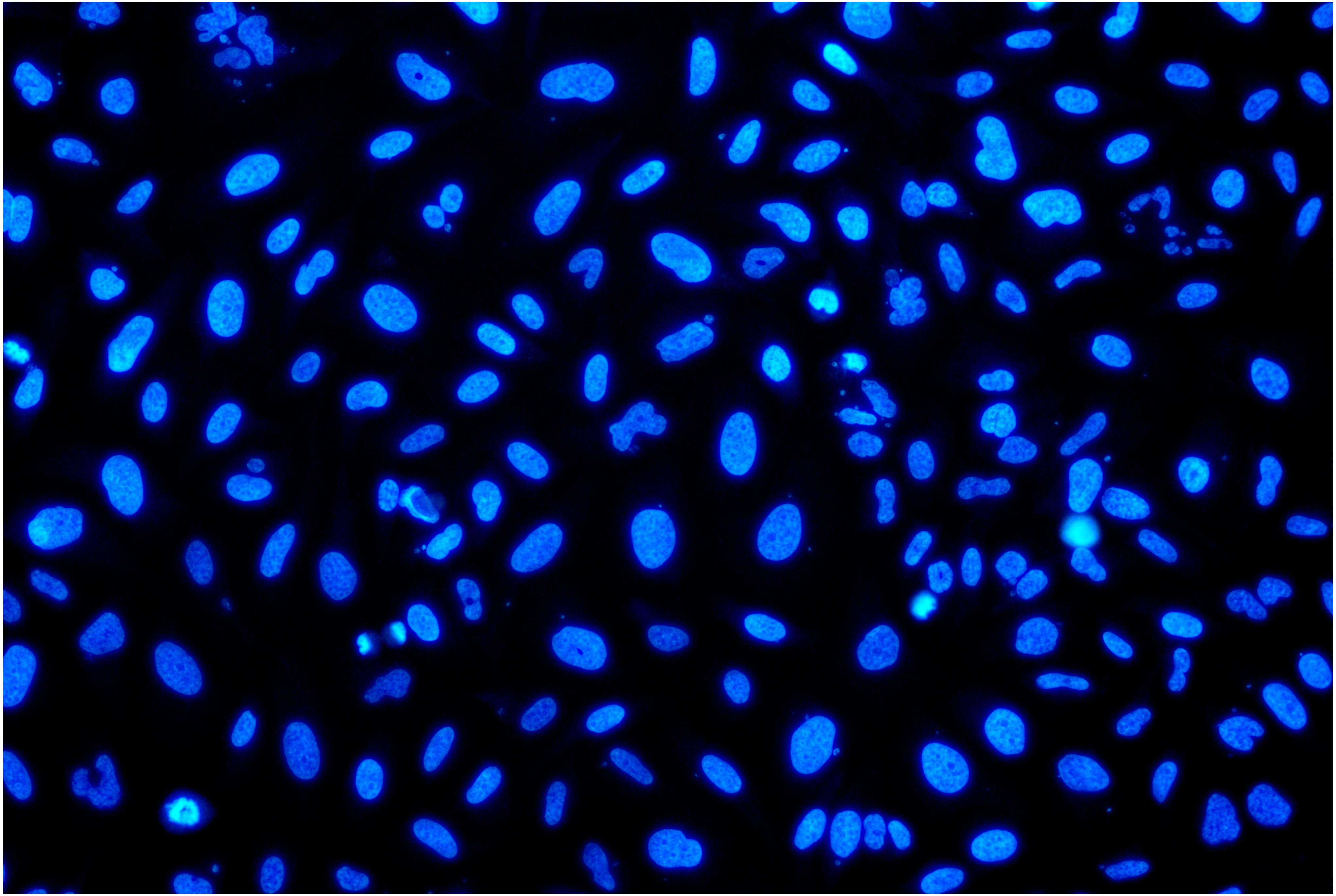

HCCLM3 Si-NC+anti-miR-NC DAPI

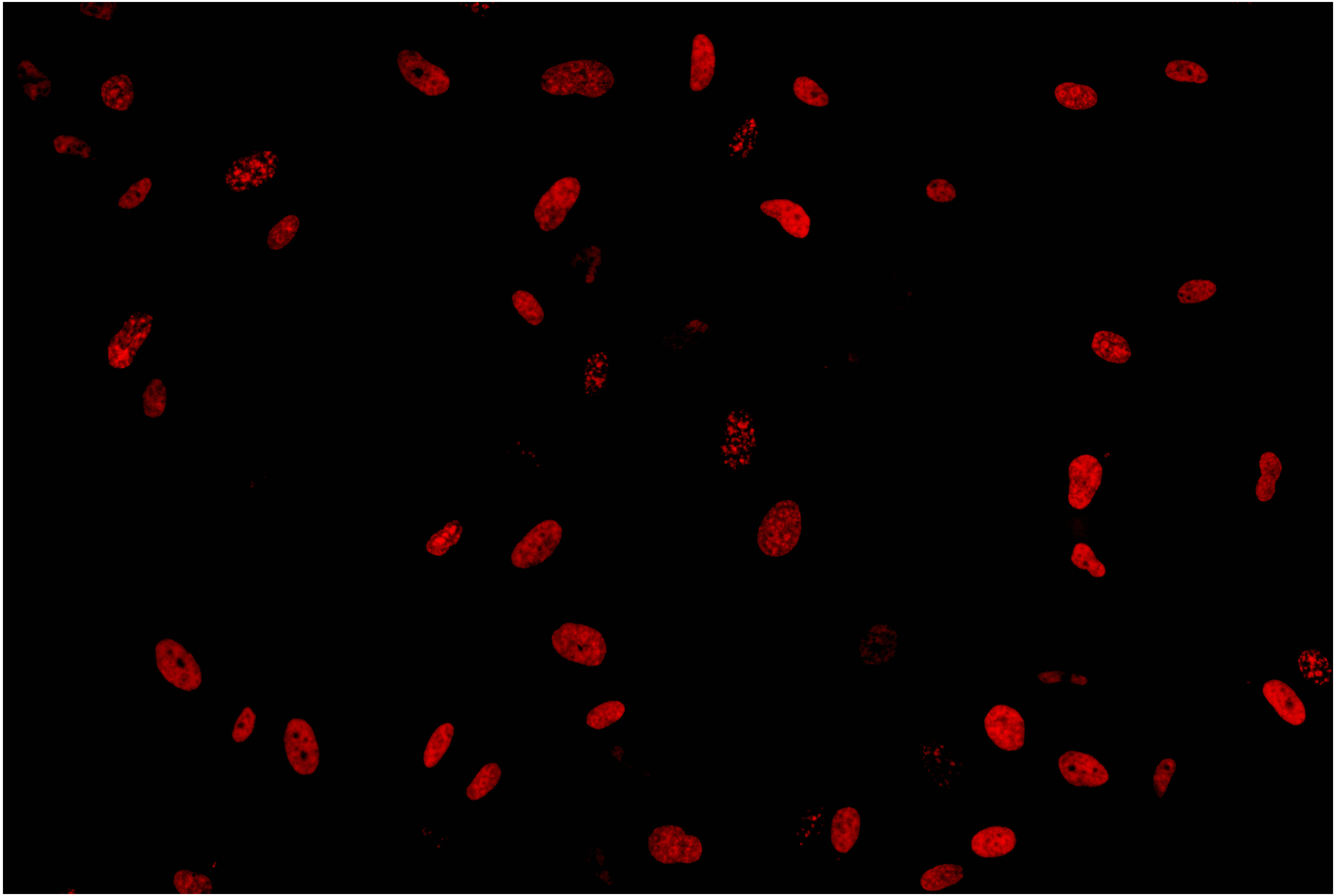

HCCLM3 Si-NC+anti-miR-NC EDU

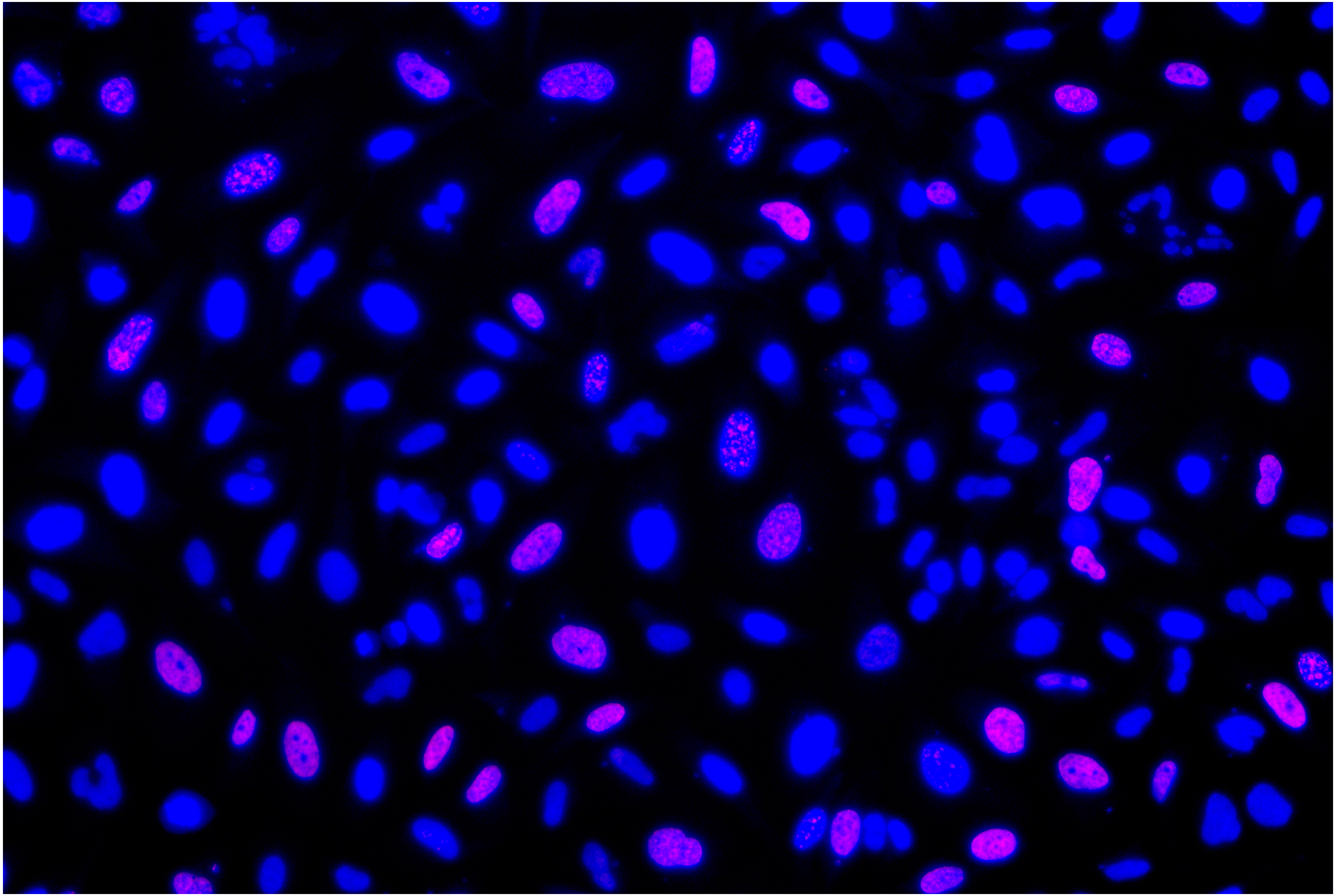

HCCLM3 Si-NC+anti-miR-NC Merge

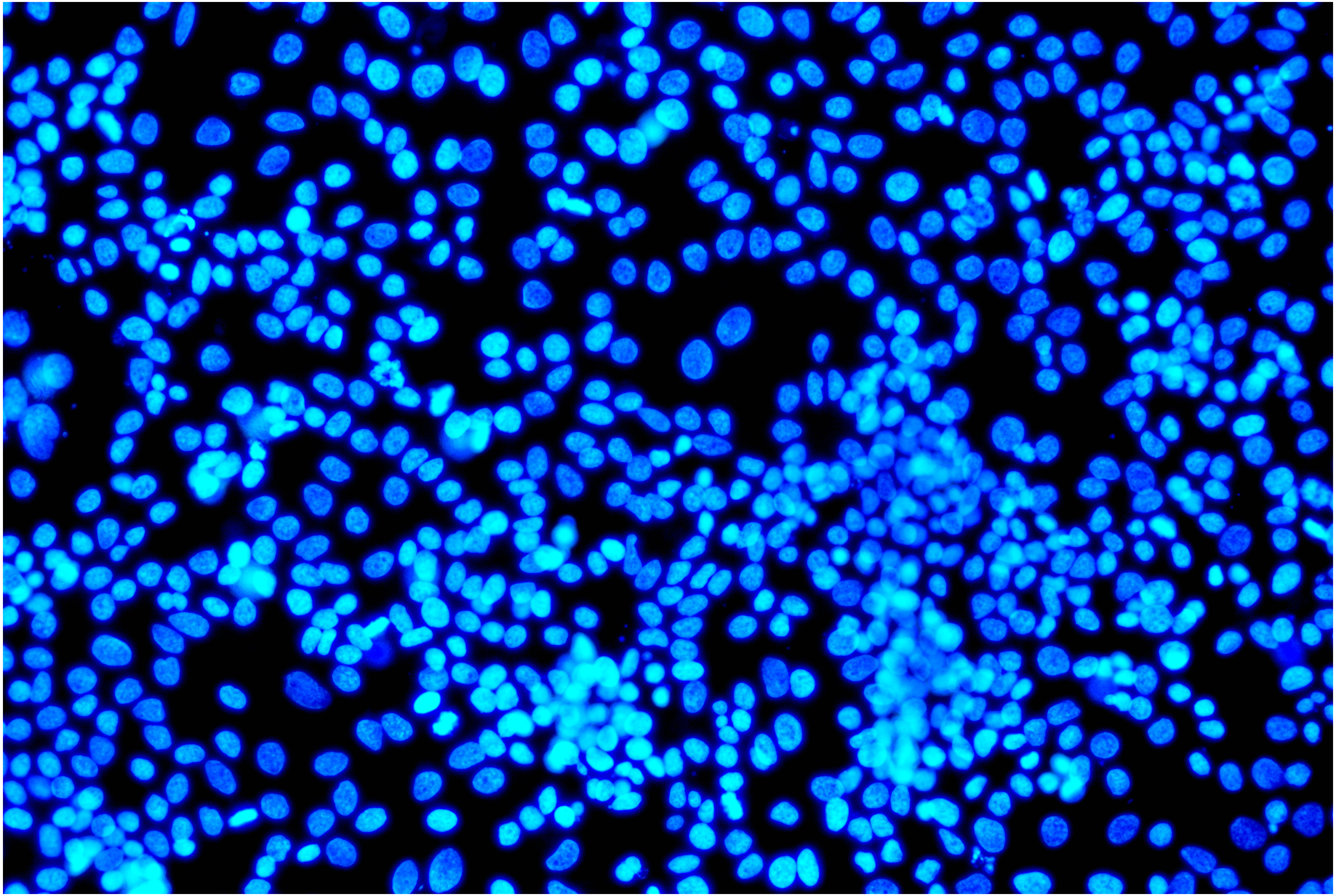

Huh7 anti-miR+si-NC DAPI

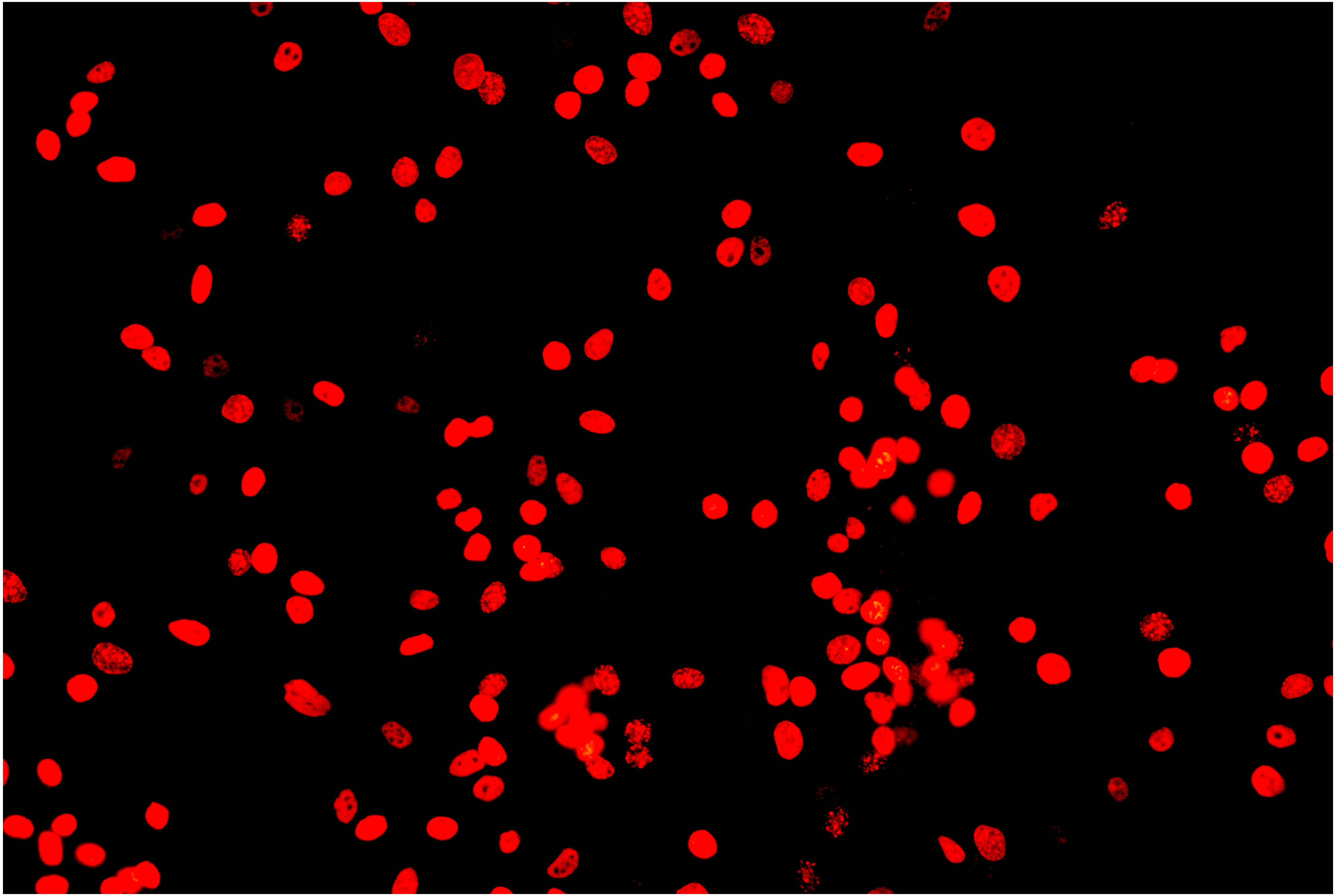

Huh7 anti-miR+si-NC EDU

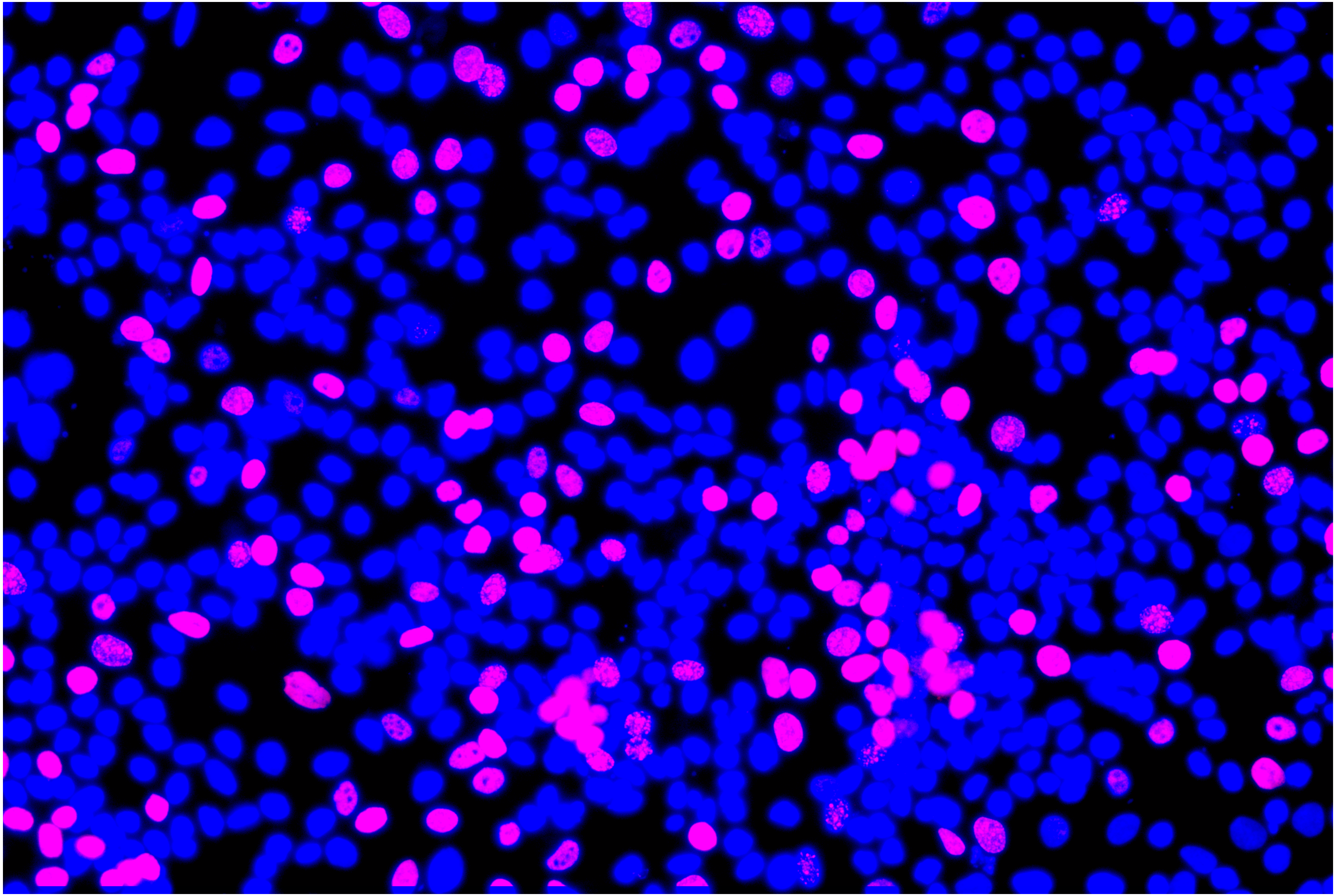

Huh7 anti-miR+si-NC Merge

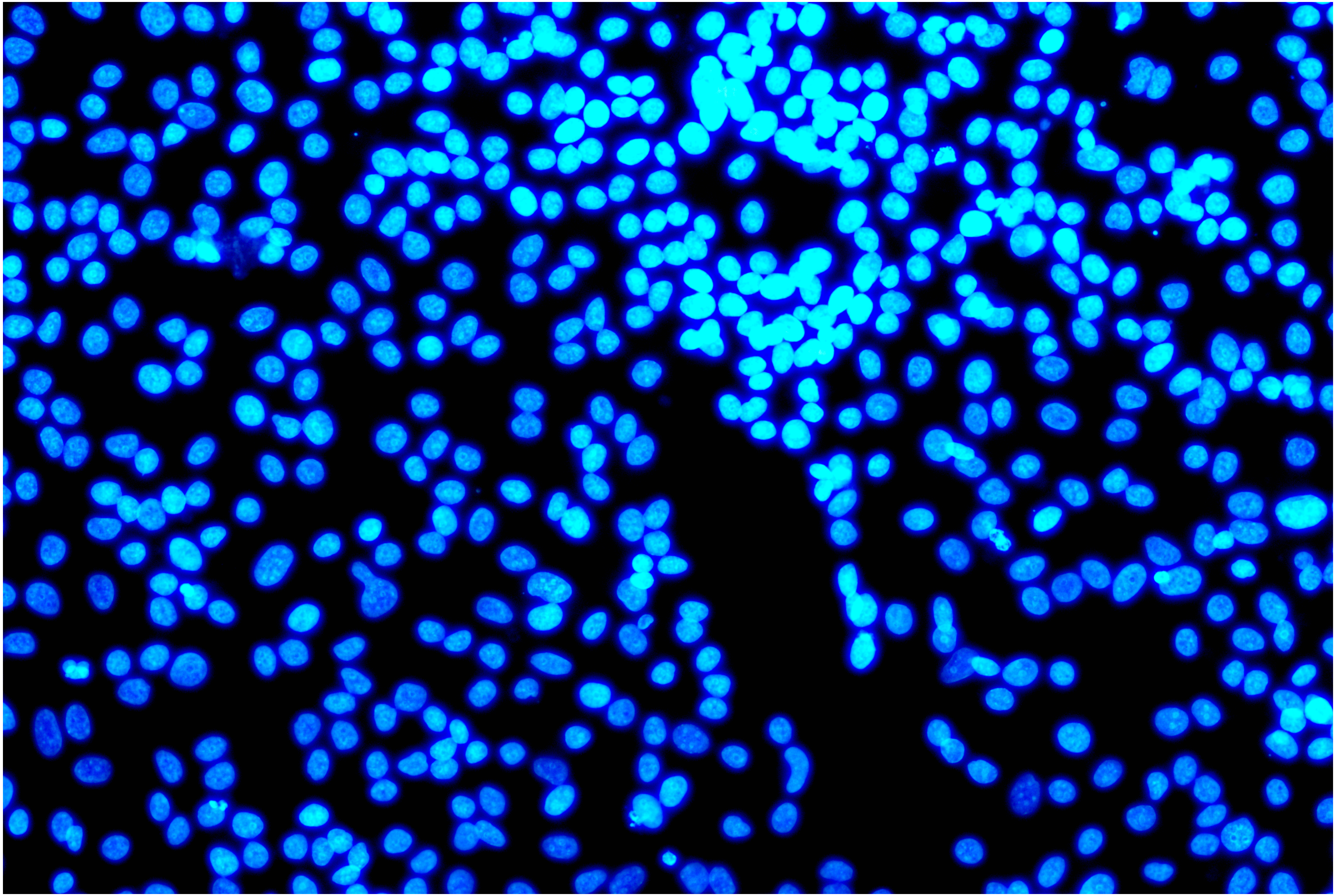

Huh7 blank DAPI

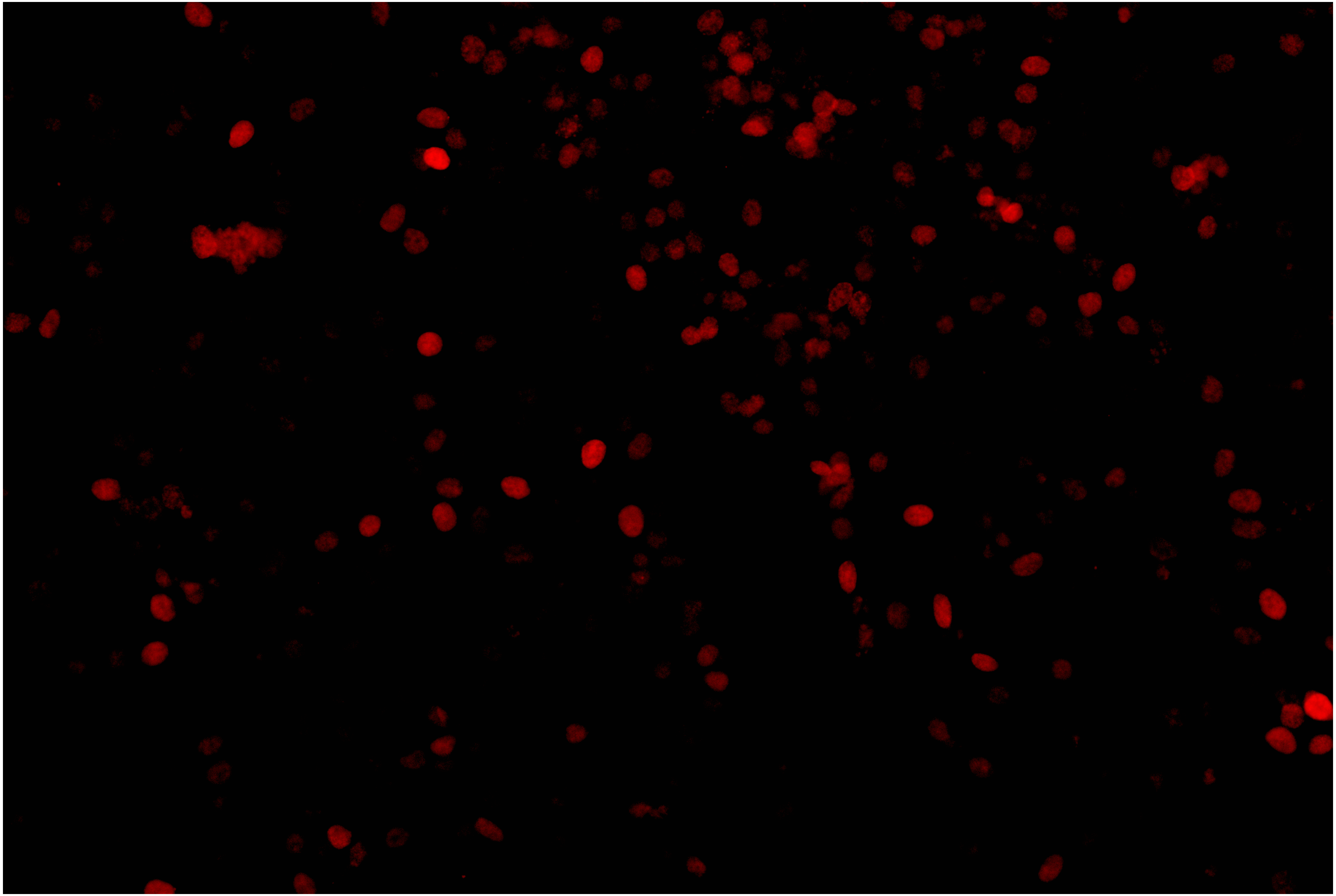

Huh7 blank EDU

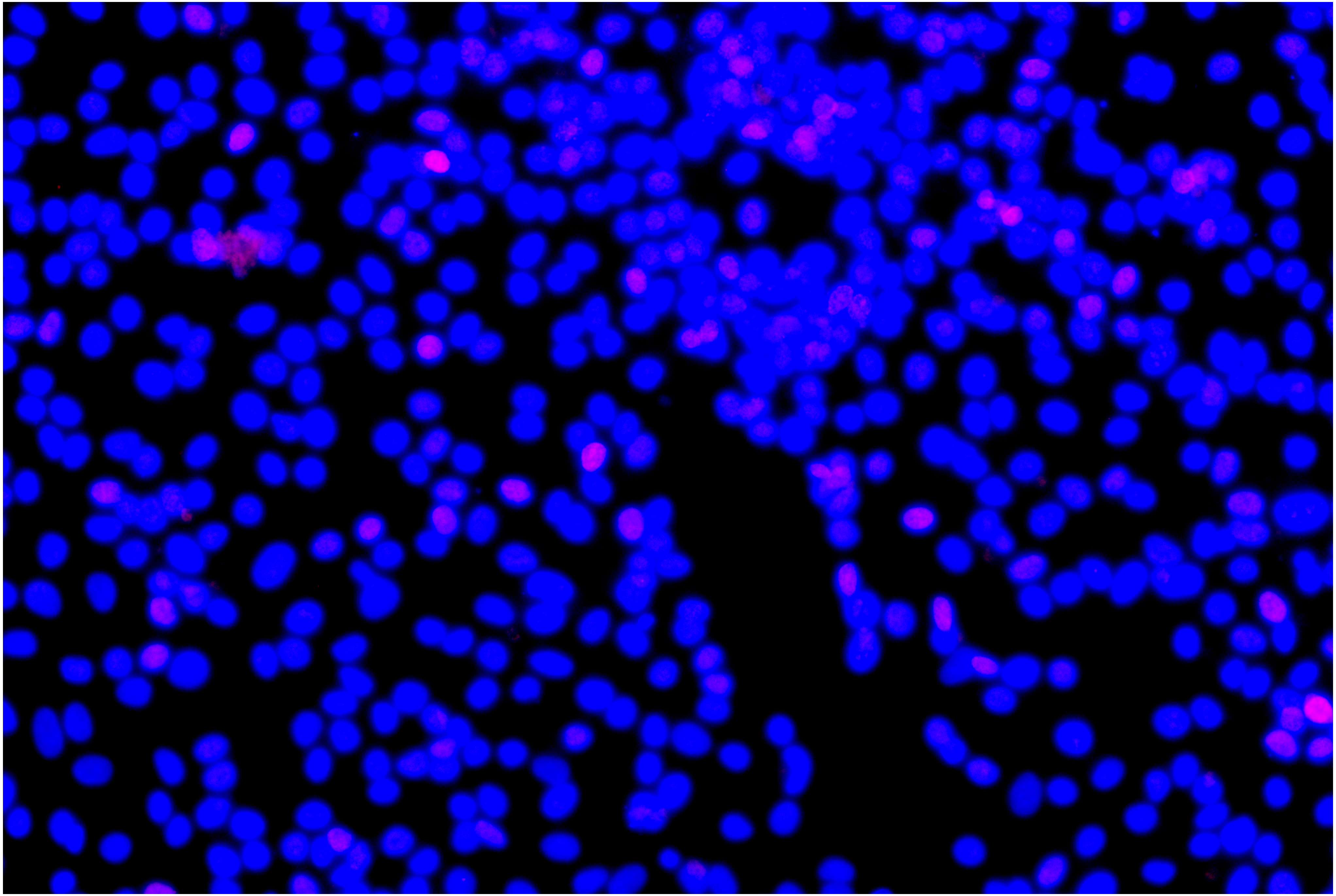

Huh7 blank Merge

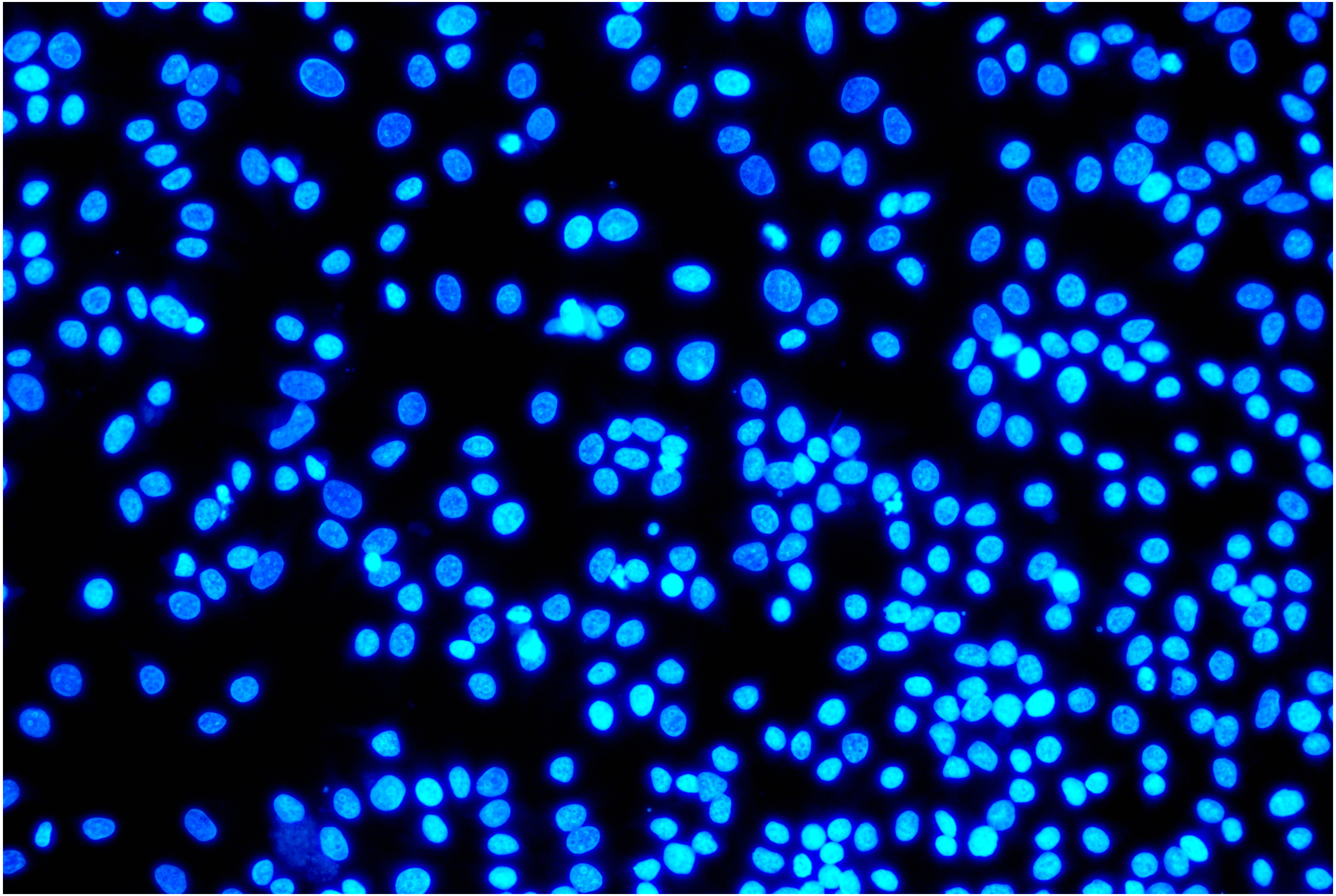

Huh7 Si-lnc+anti-miR DAPI

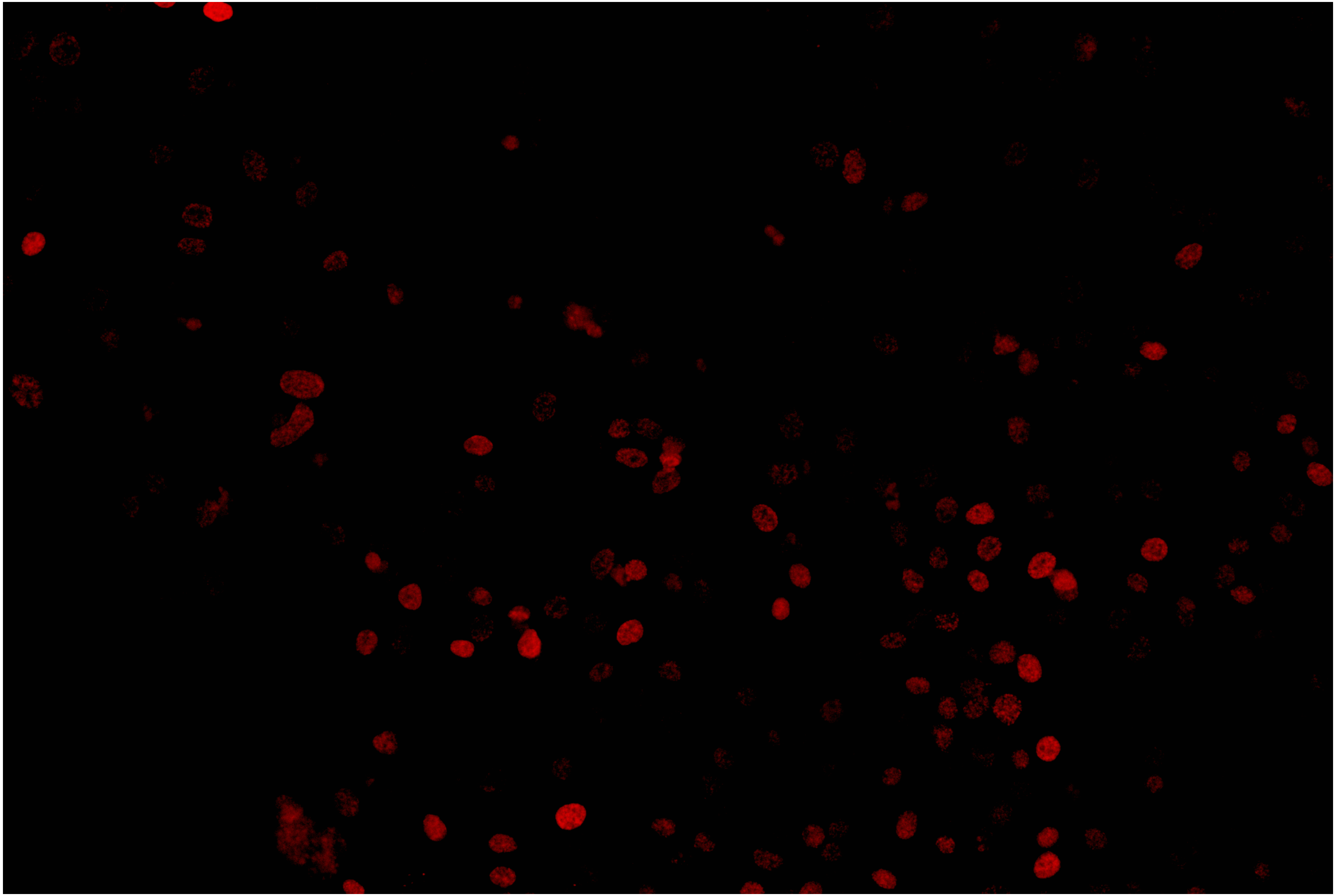

Huh7 Si-lnc+anti-miR EDU

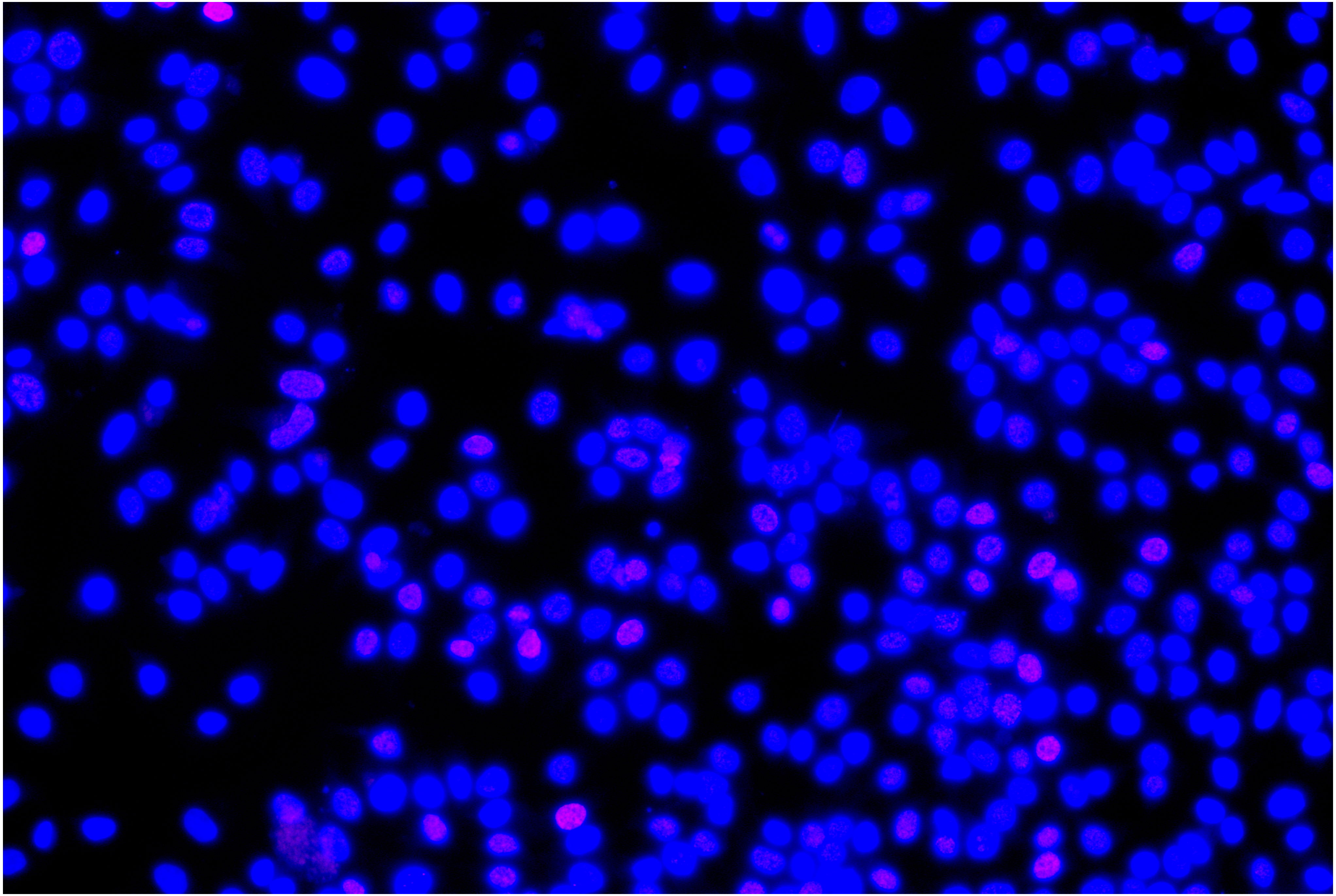

Huh7 Si-lnc+anti-miR Merge

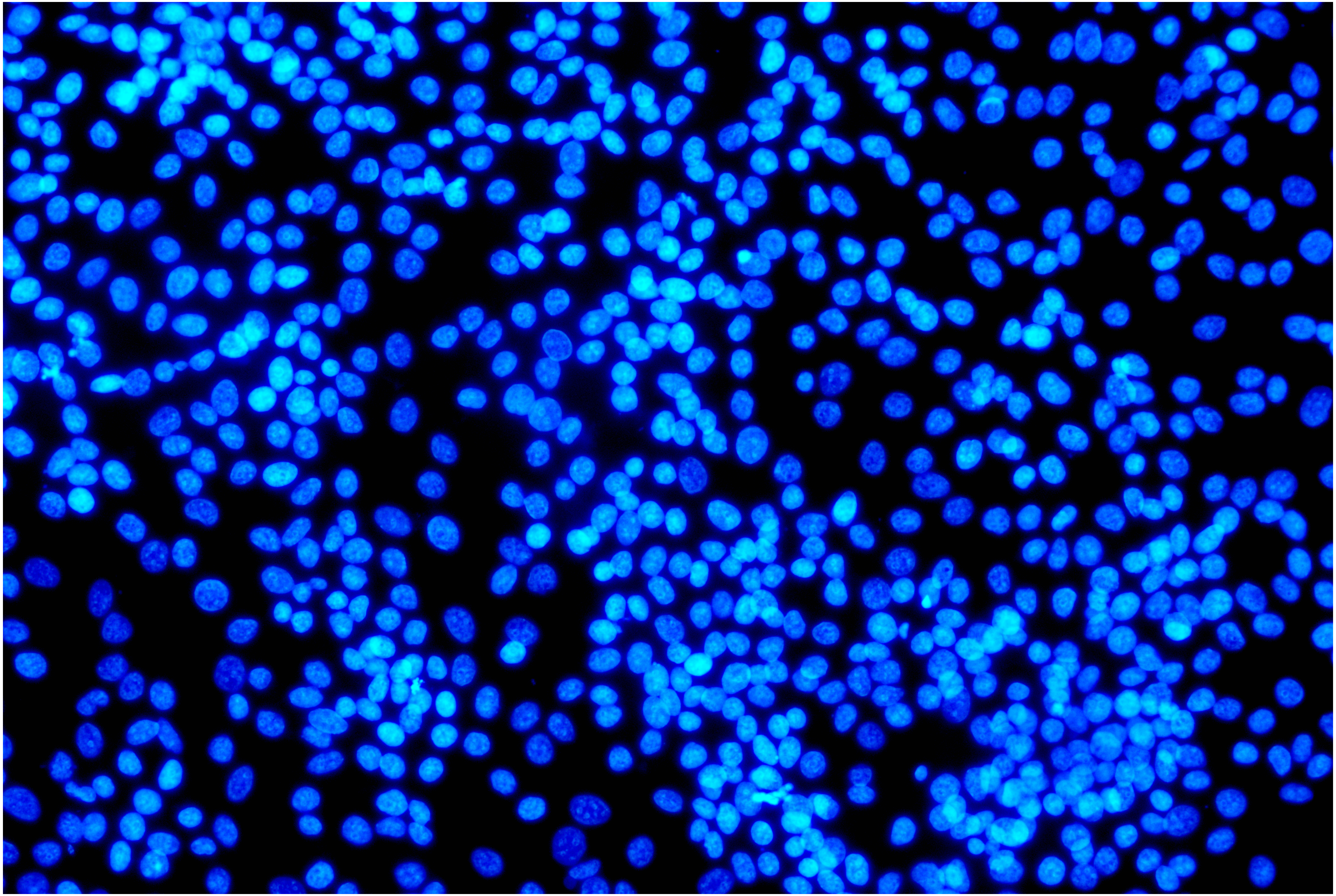

Huh7 Si-lnc+anti-miR-NC DAPI

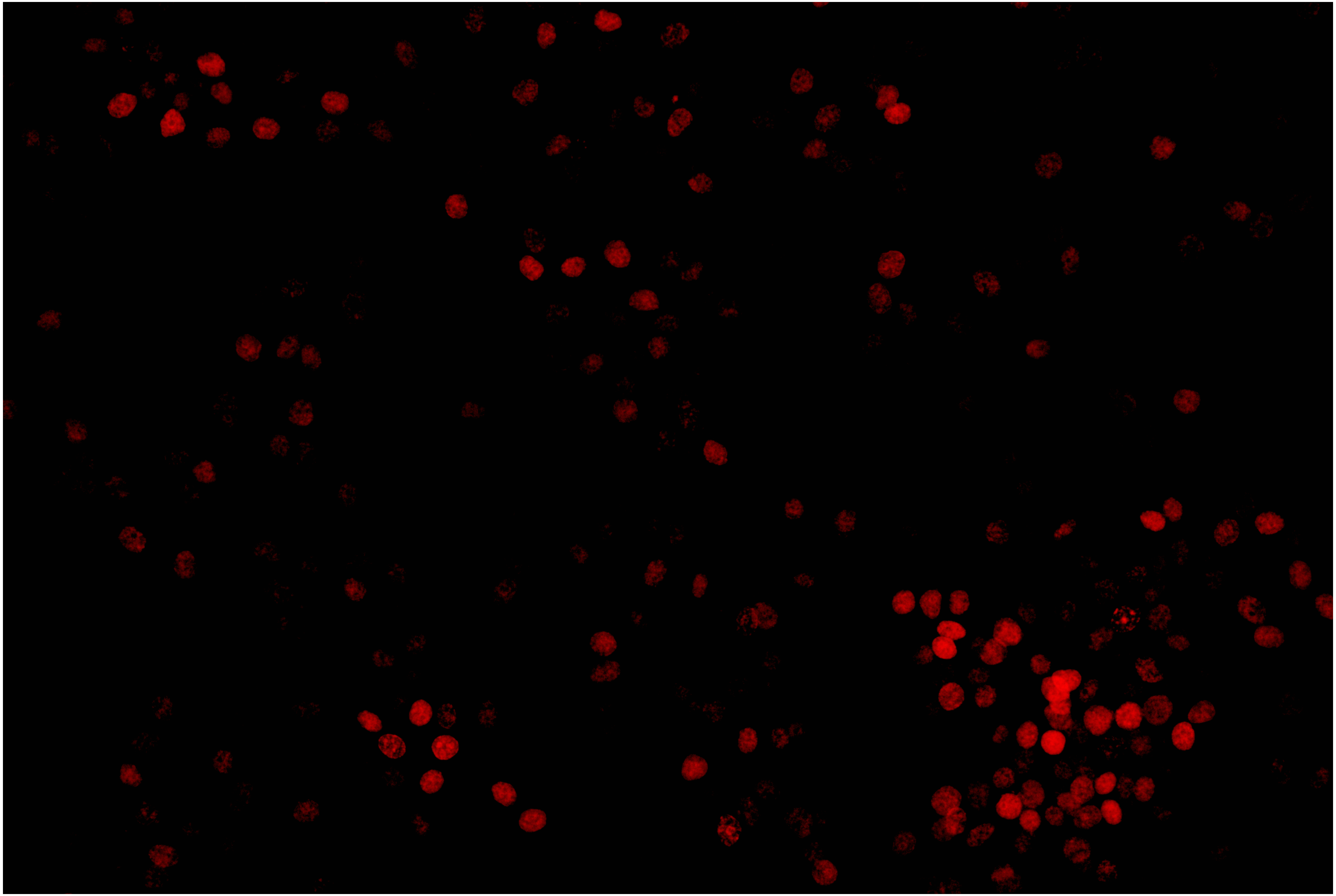

Huh7 Si-lnc+anti-miR-NC EDU

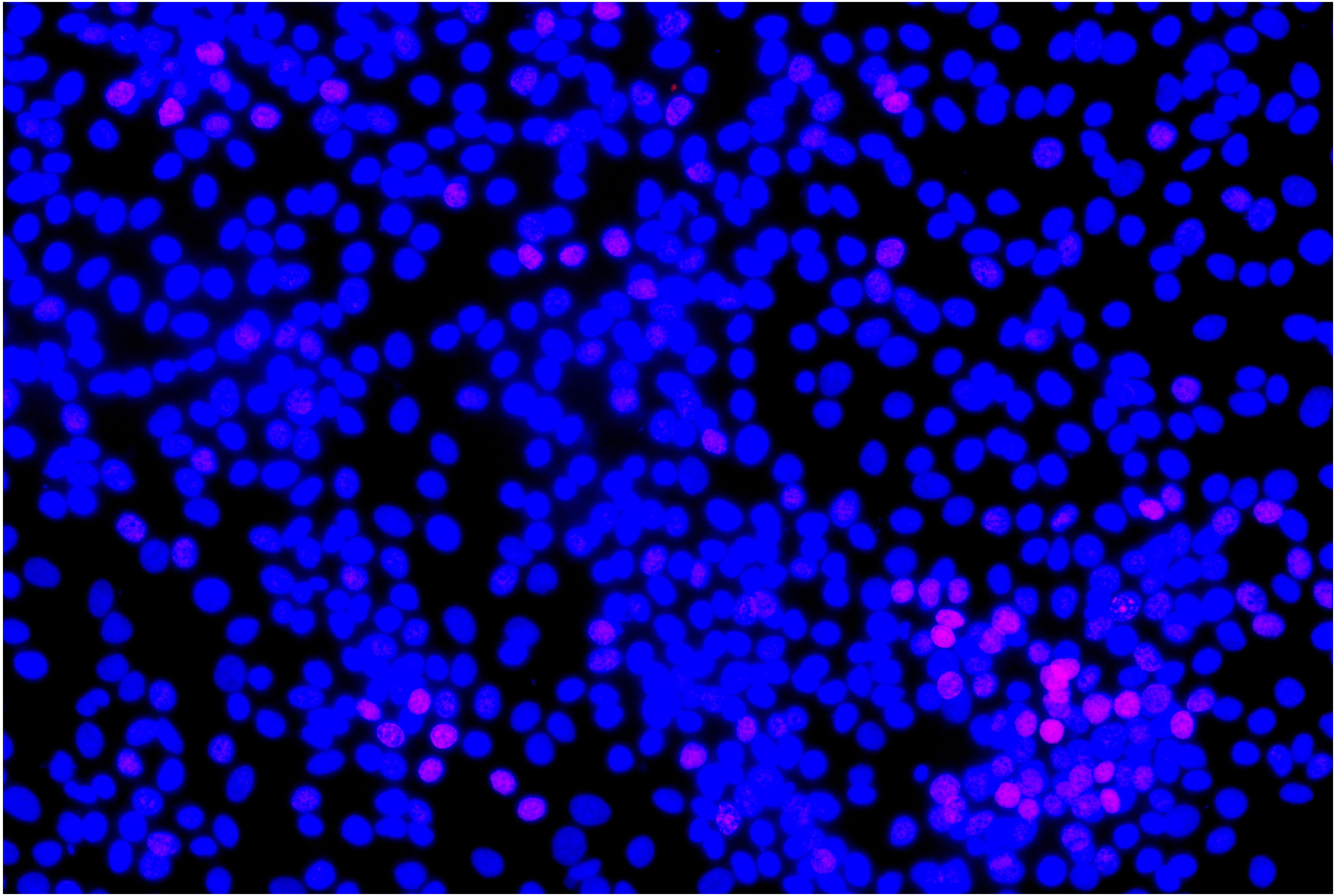

Huh7 Si-lnc+anti-miR-NC Merge

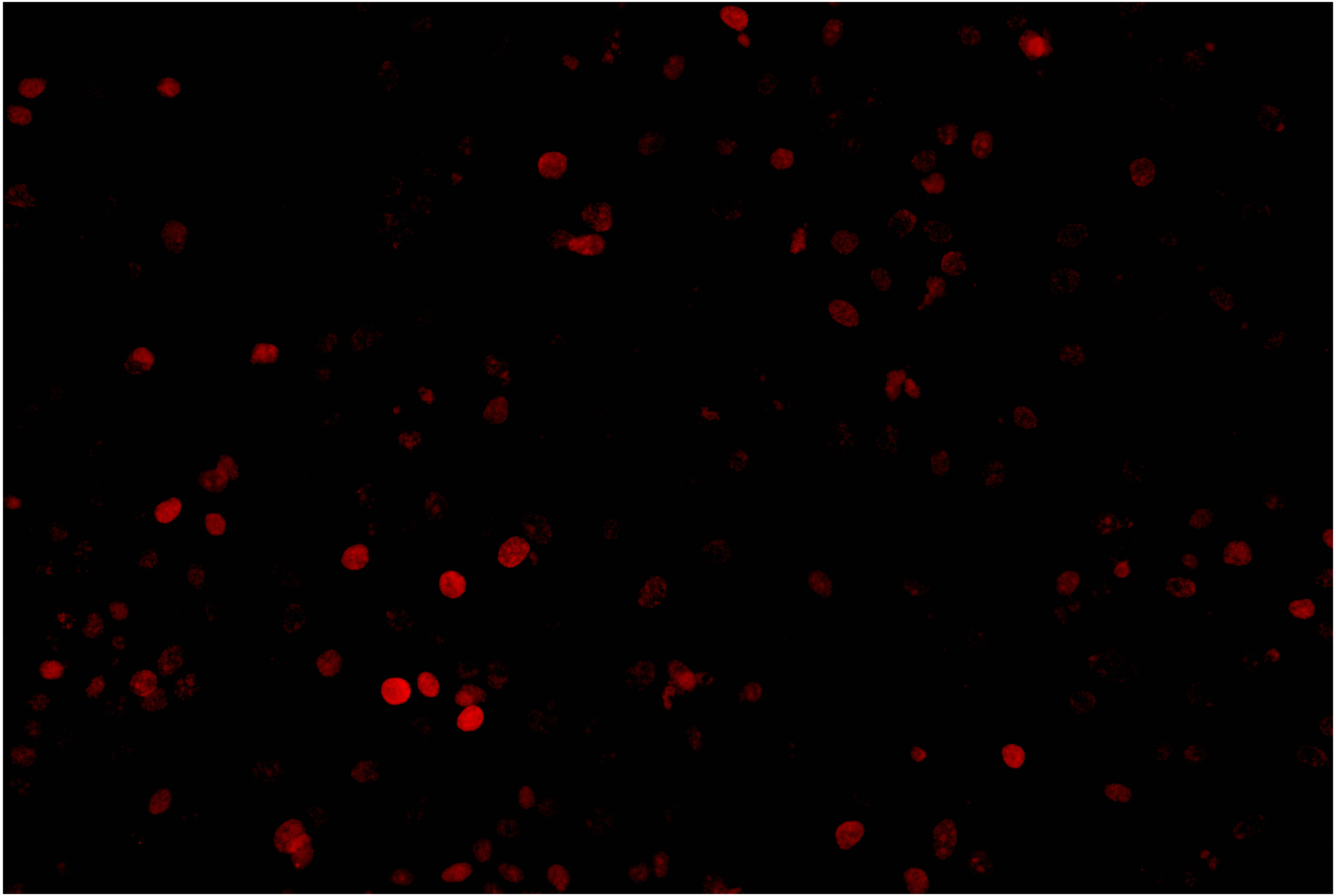

Huh7 Si-NC+anti-miR-NC EDU

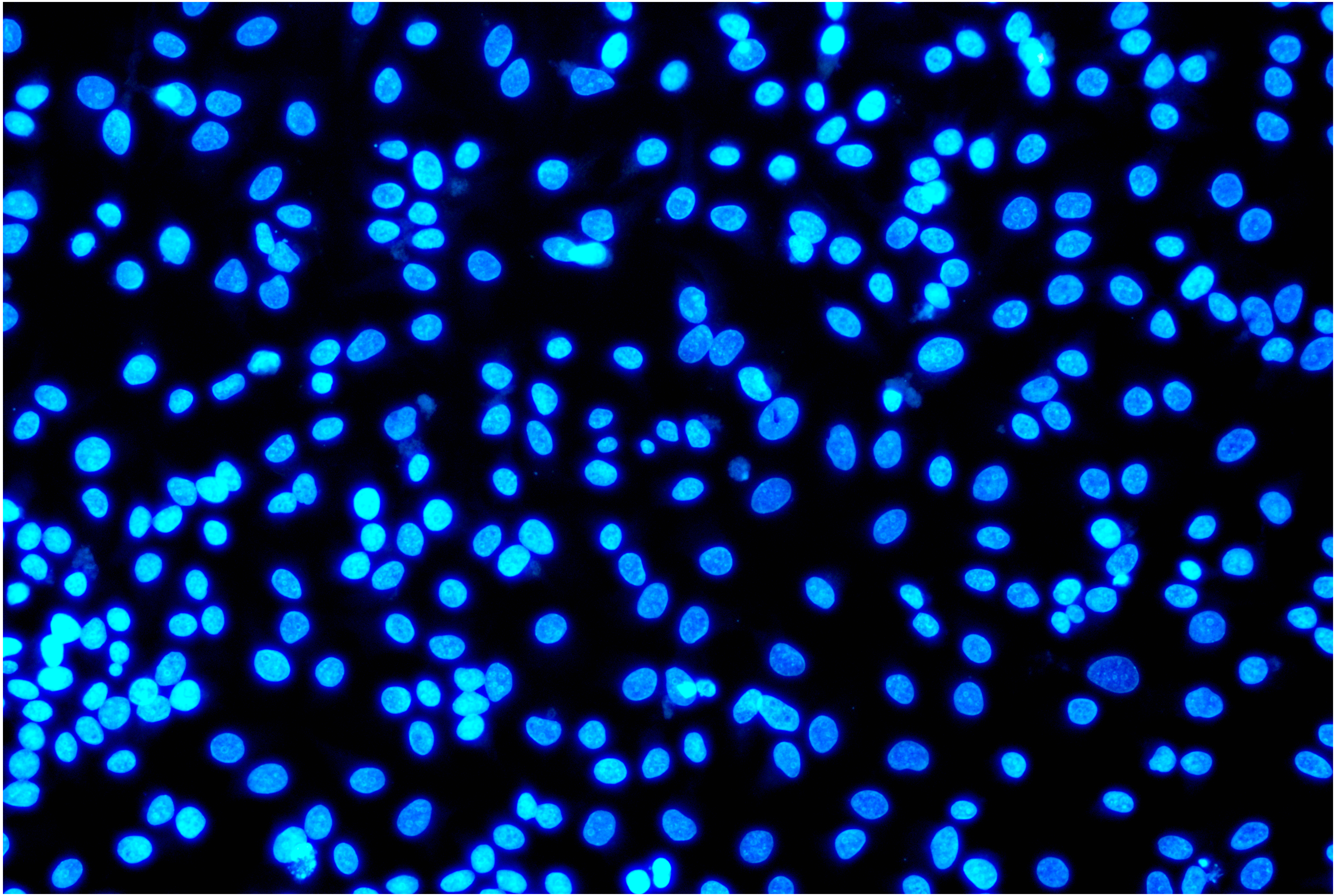

Huh7 Si-NC+anti-miR-NC DAPI

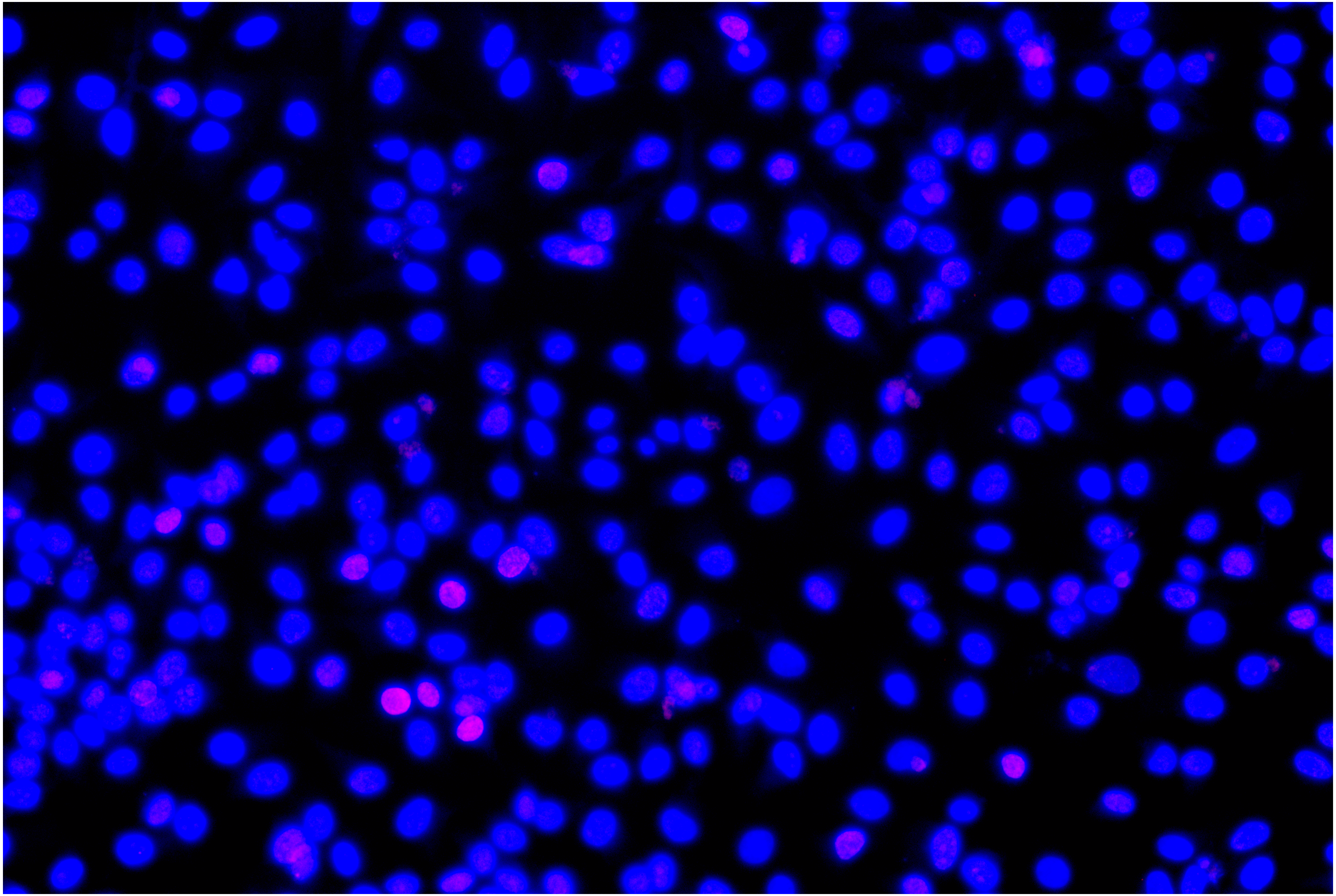

Huh7 Si-NC+anti-miR-NC Merge

Figure 9B

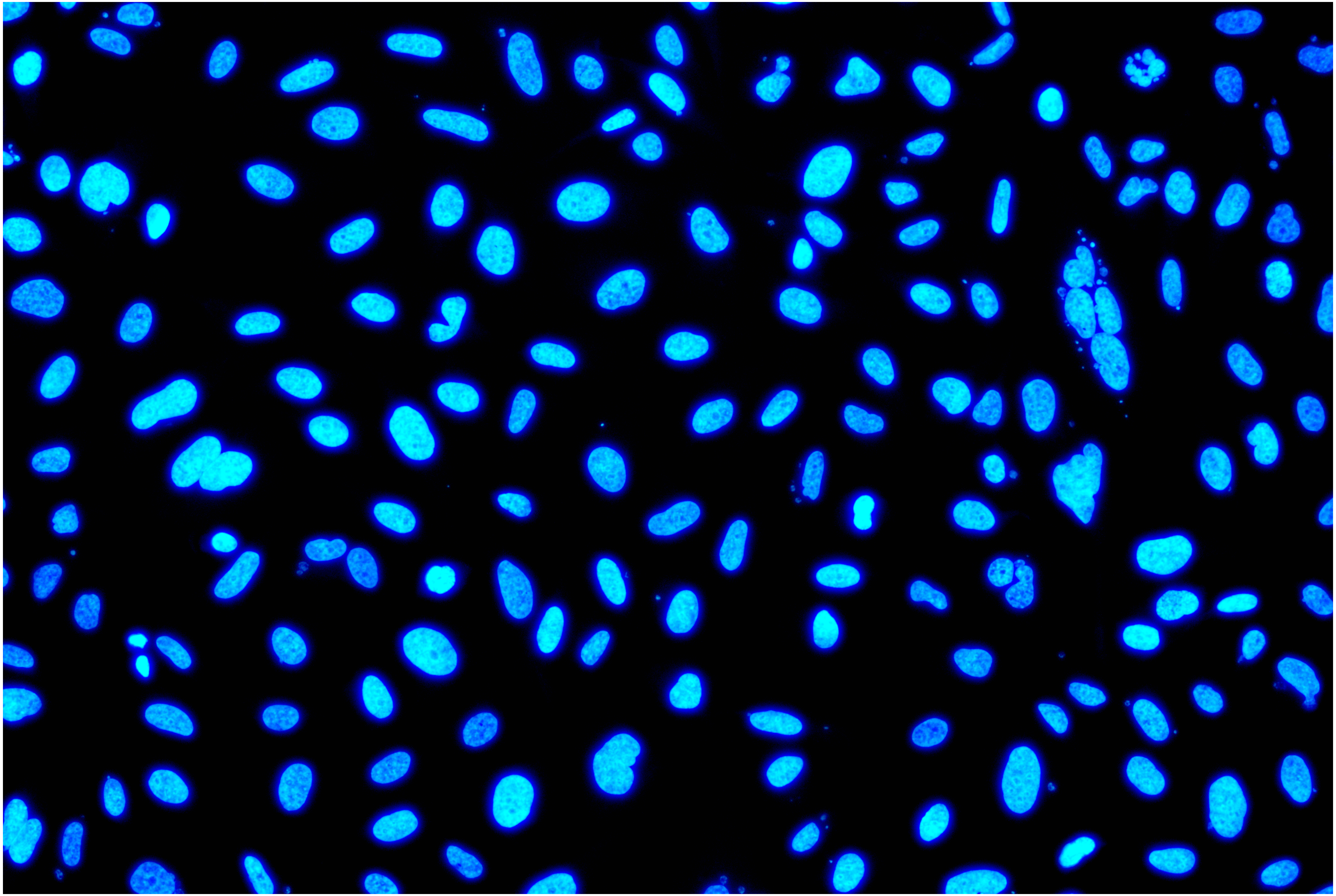

HCCLM3 anti-miR+si-NC DAPI

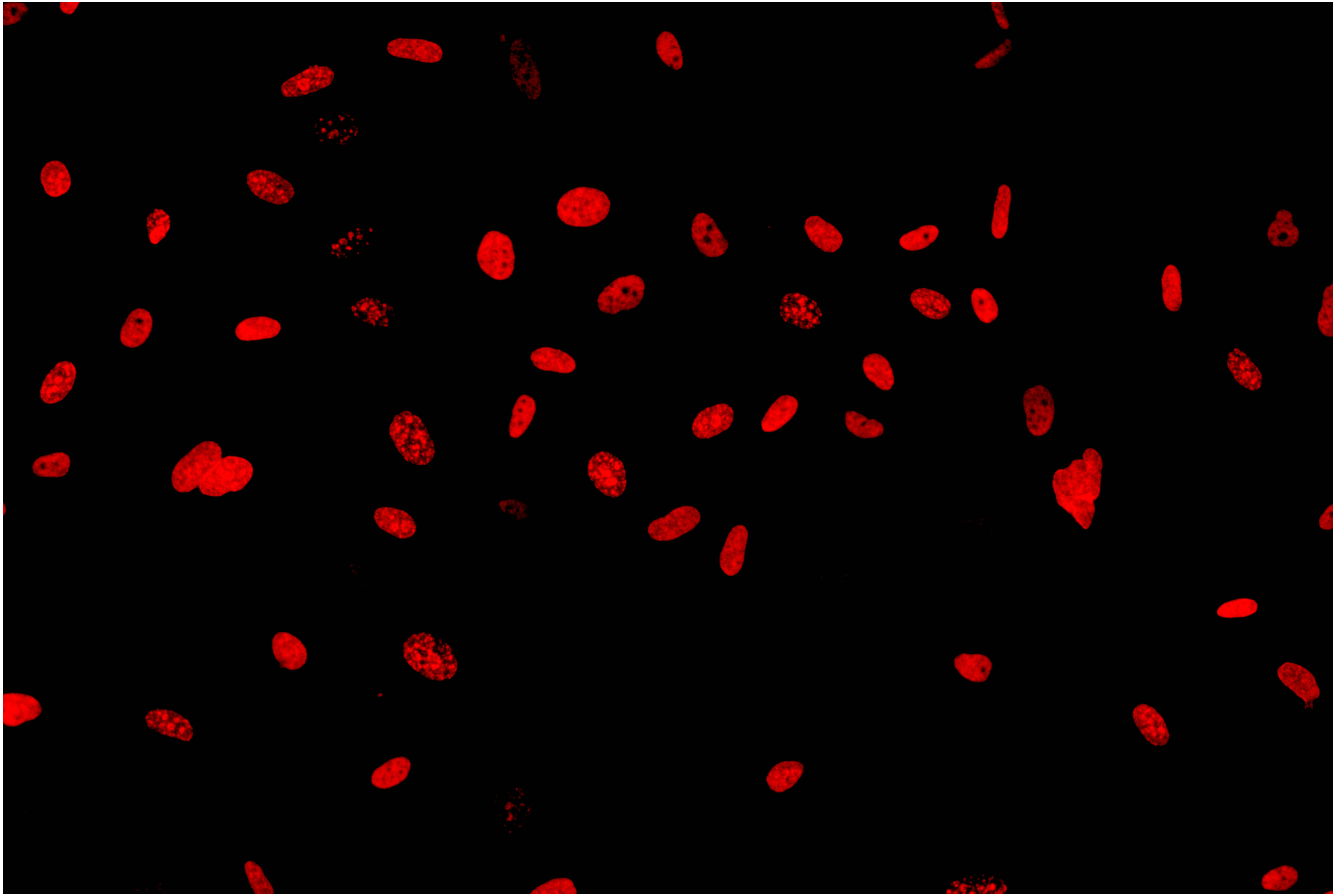

HCCLM3 anti-miR+si-NC EDU

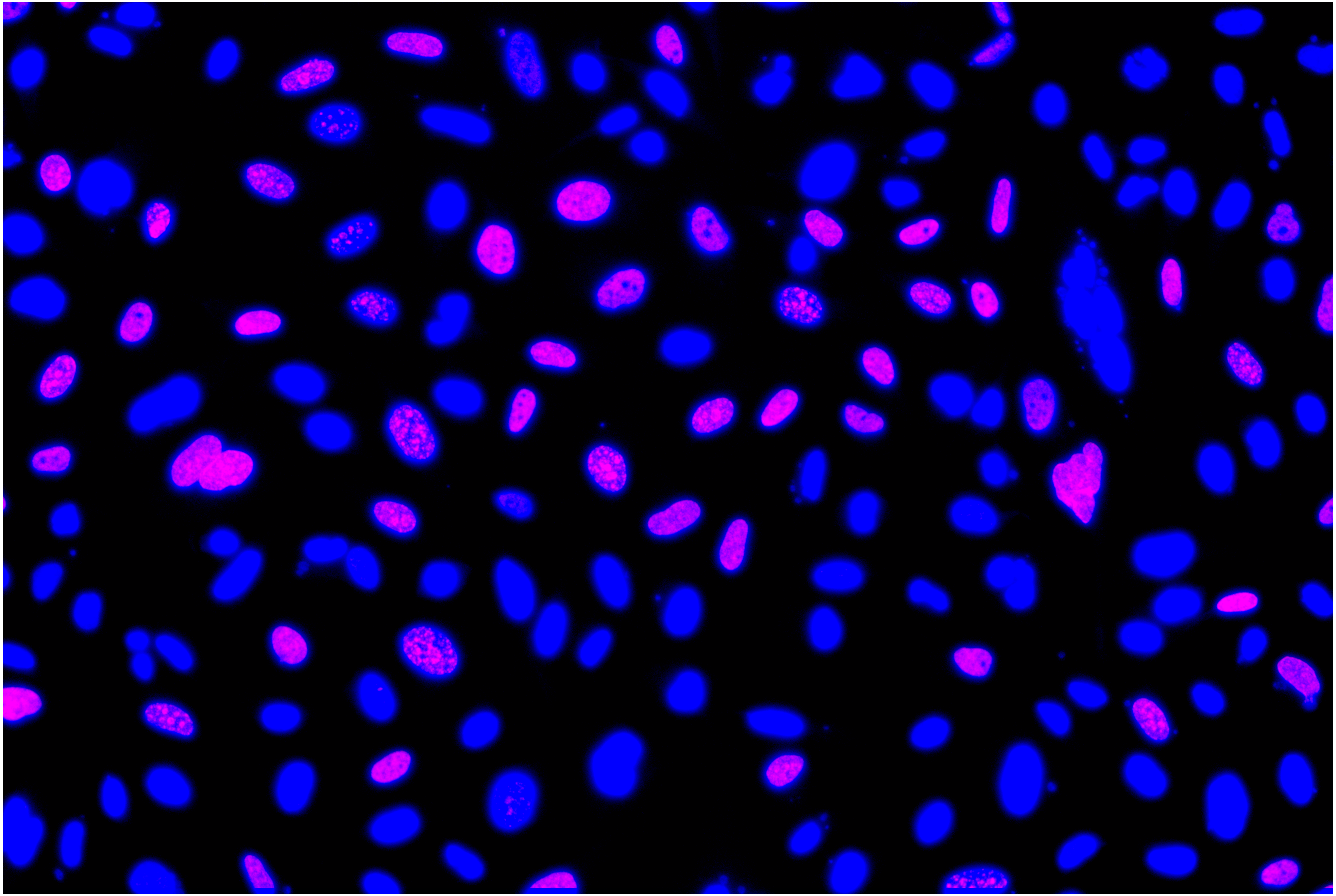

HCCLM3 anti-miR+si-NC Merge

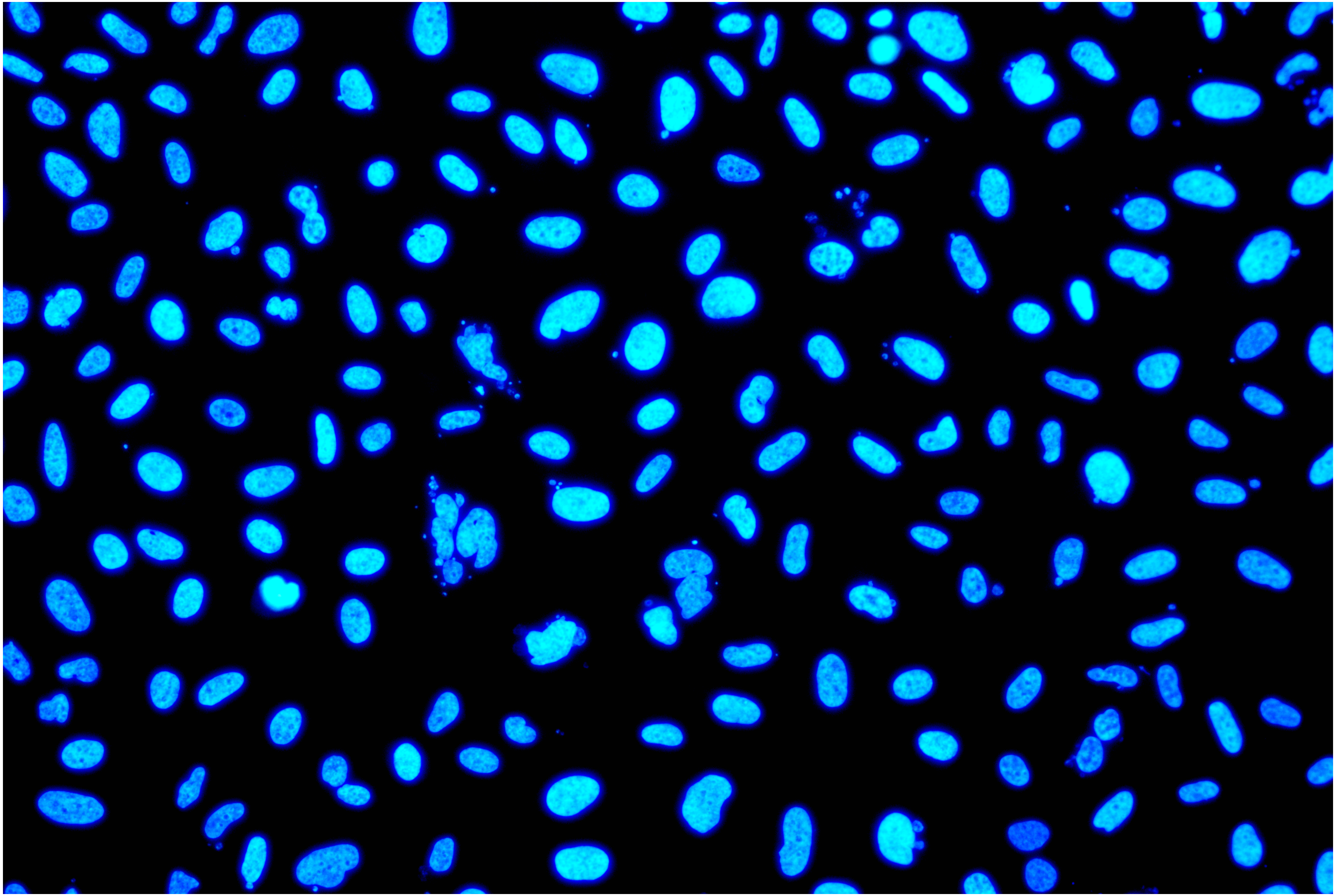

HCCLM3 blank DAPI

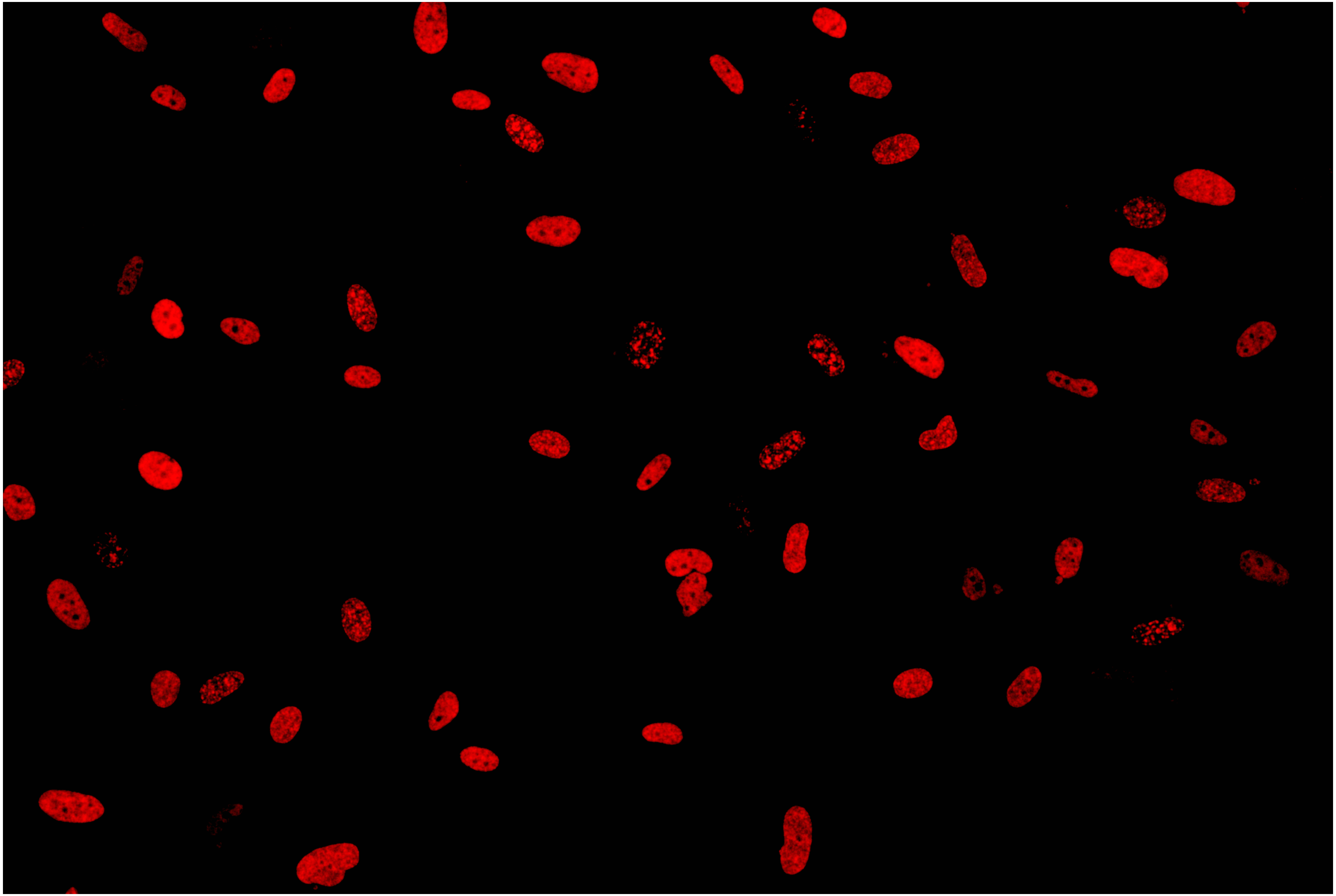

HCCLM3 blank EDU

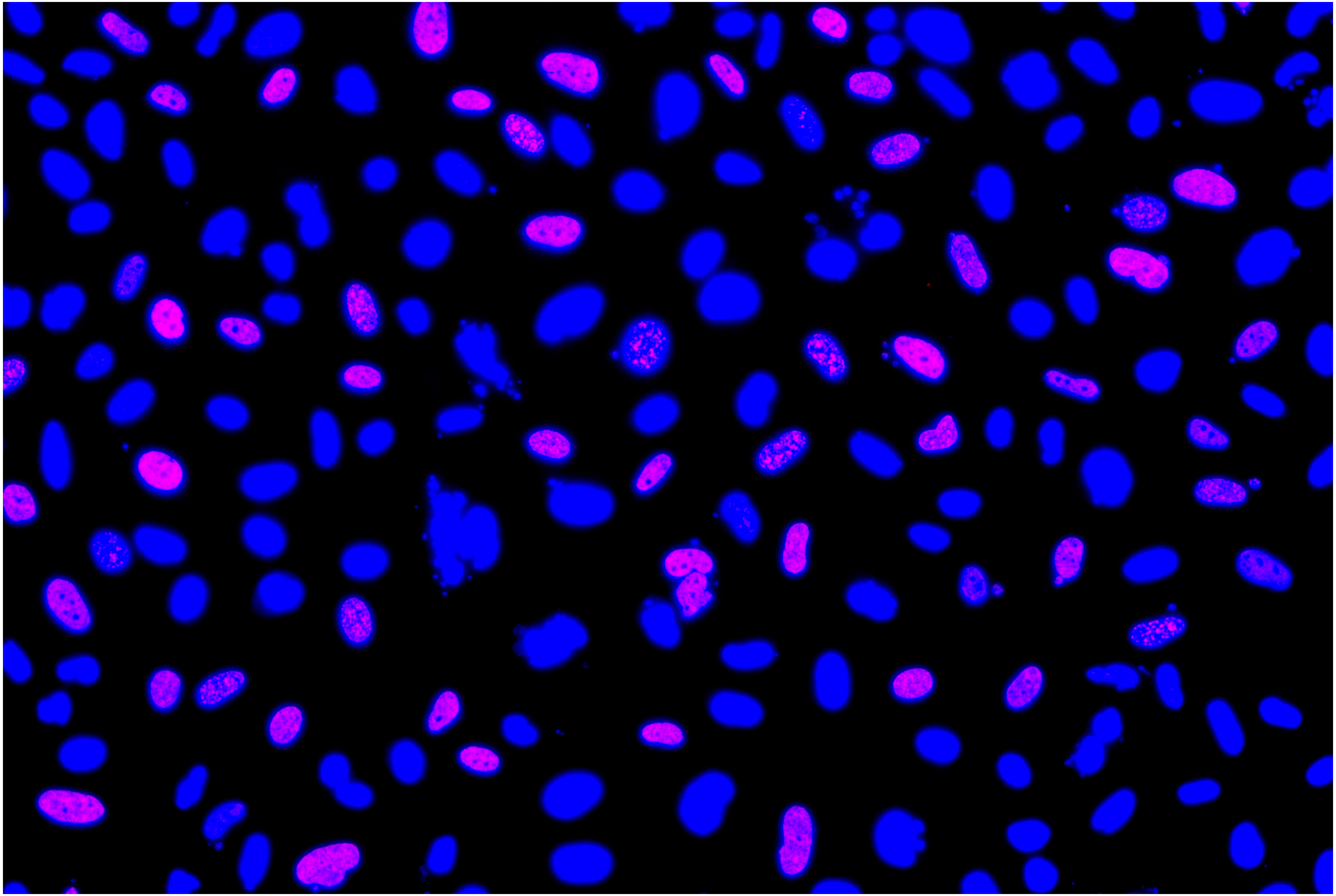

HCCLM3 blank Merge

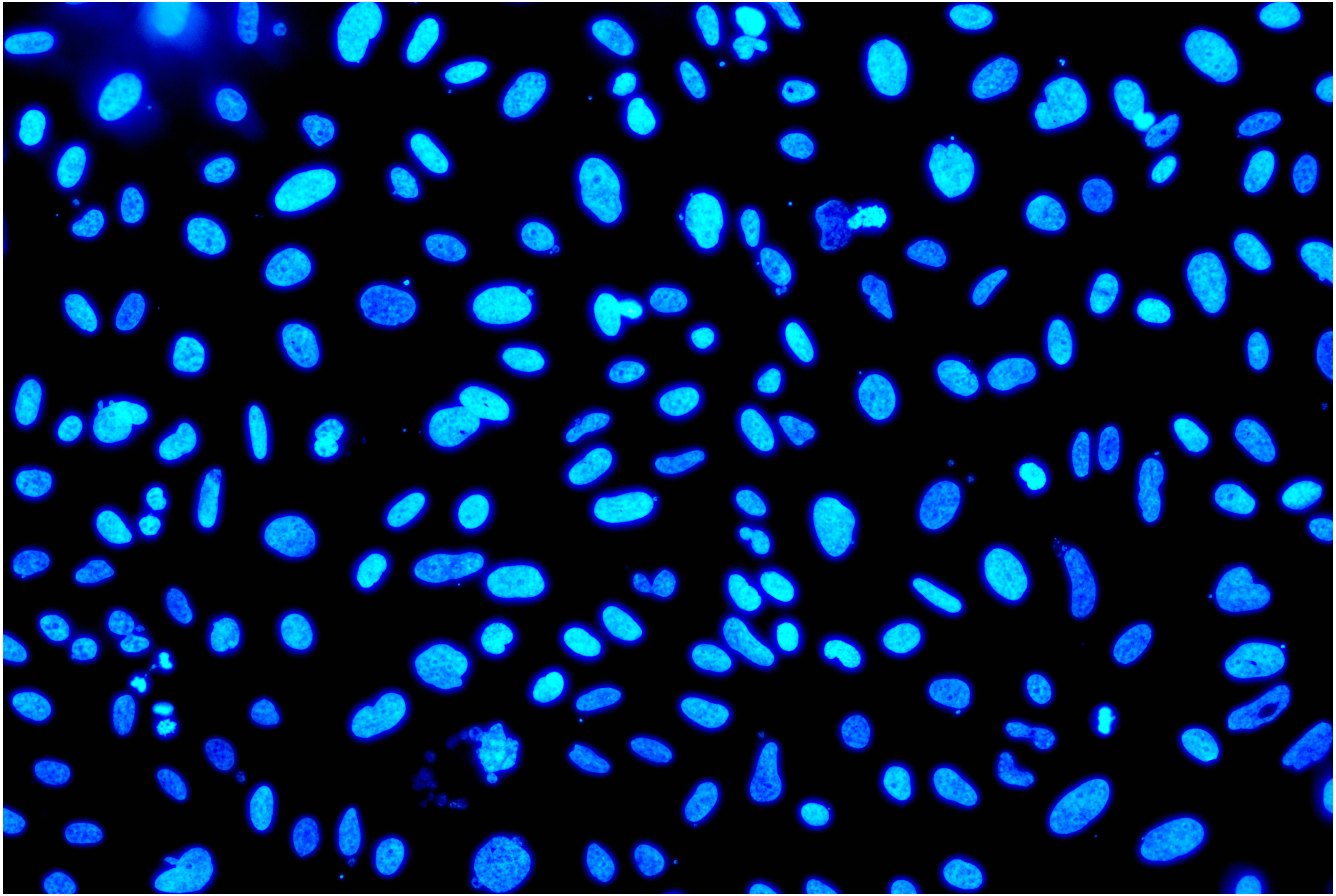

HCCLM3 Si-HMGA1+anti-miR DAPI

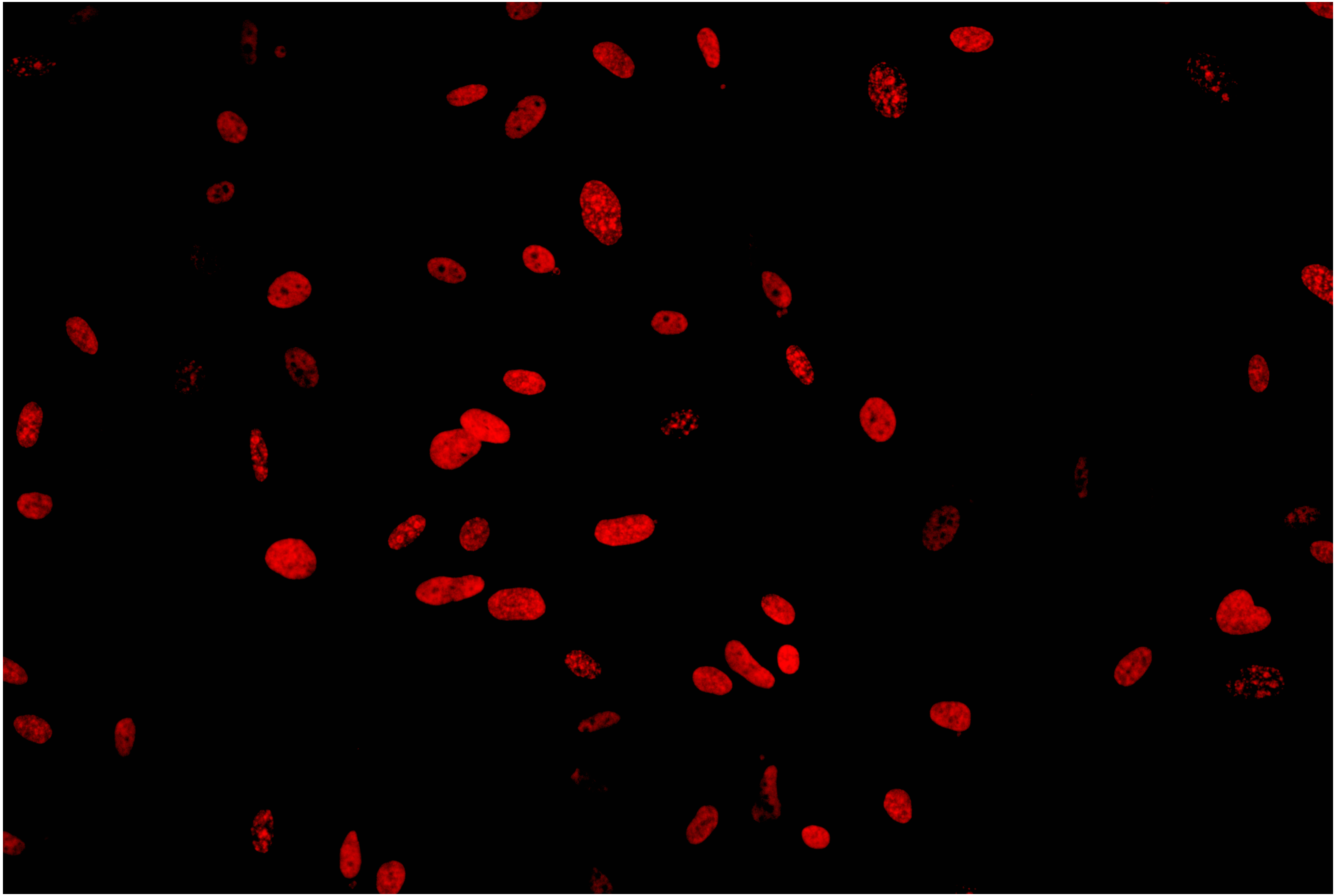

HCCLM3 Si-HMGA1+anti-miR EDU

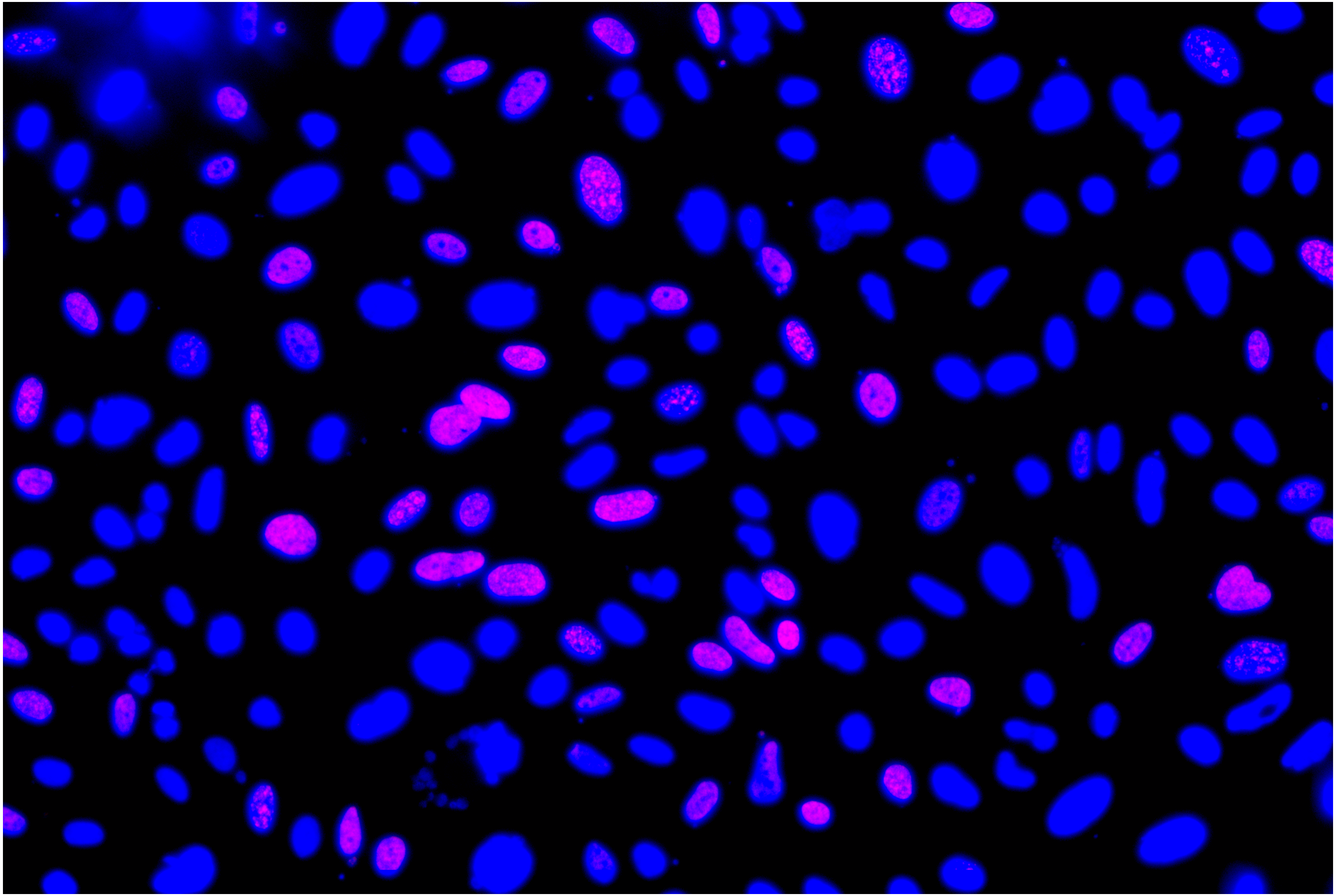

HCCLM3 Si-HMGA1+anti-miR Merge

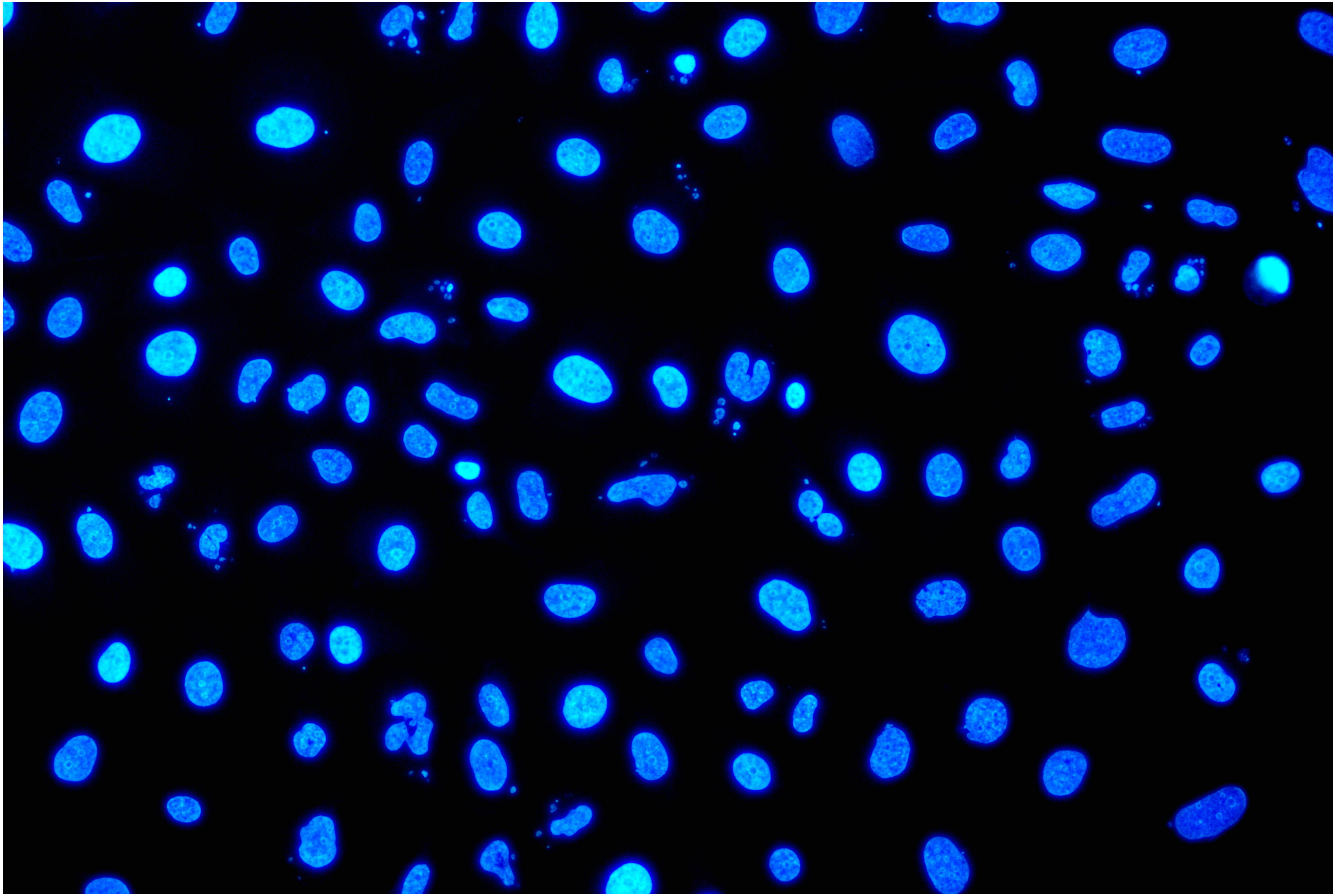

HCCLM3 Si-HMGA1+anti-miR-NC DAPI

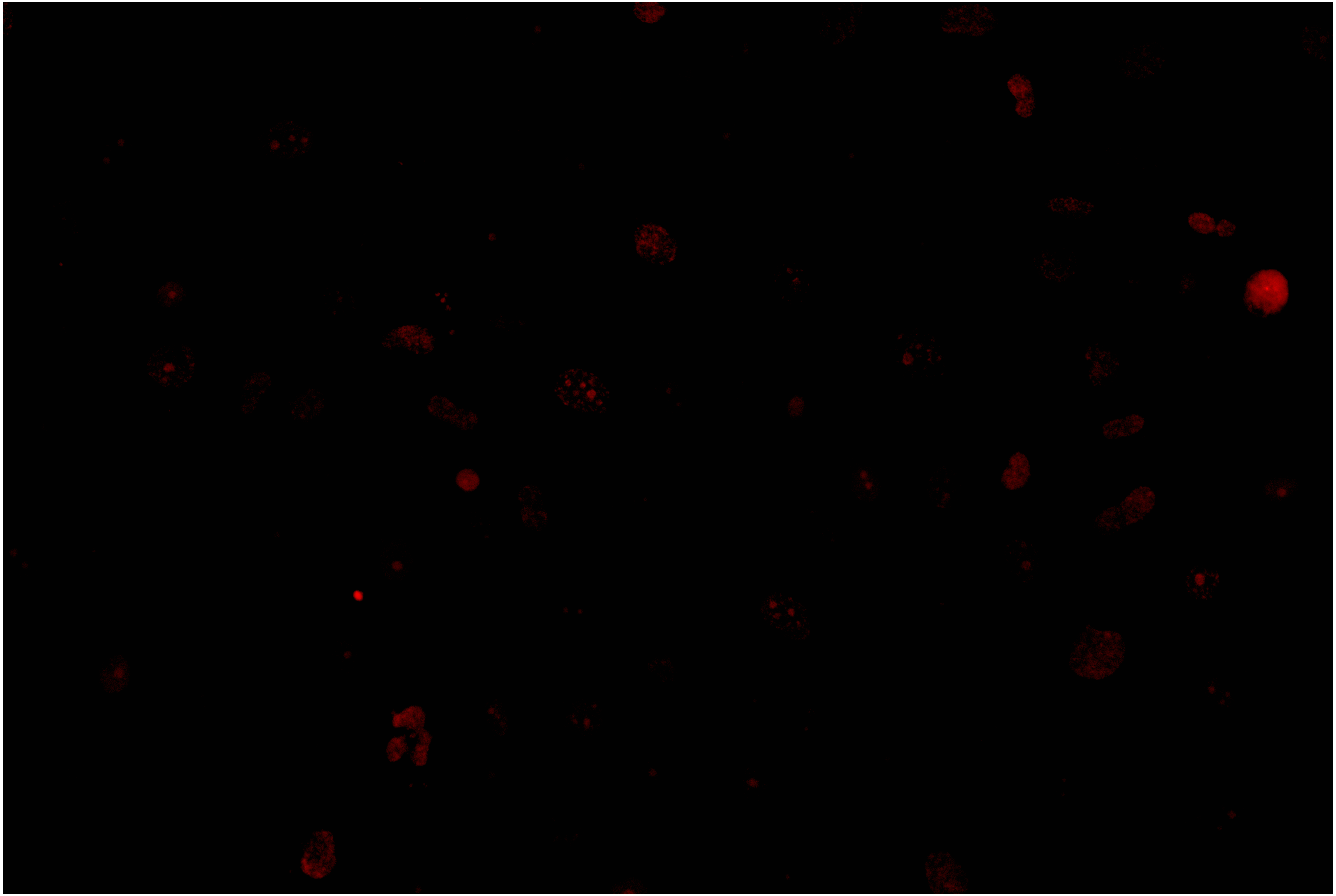

HCCLM3 Si-HMGA1+anti-miR-NC EDU

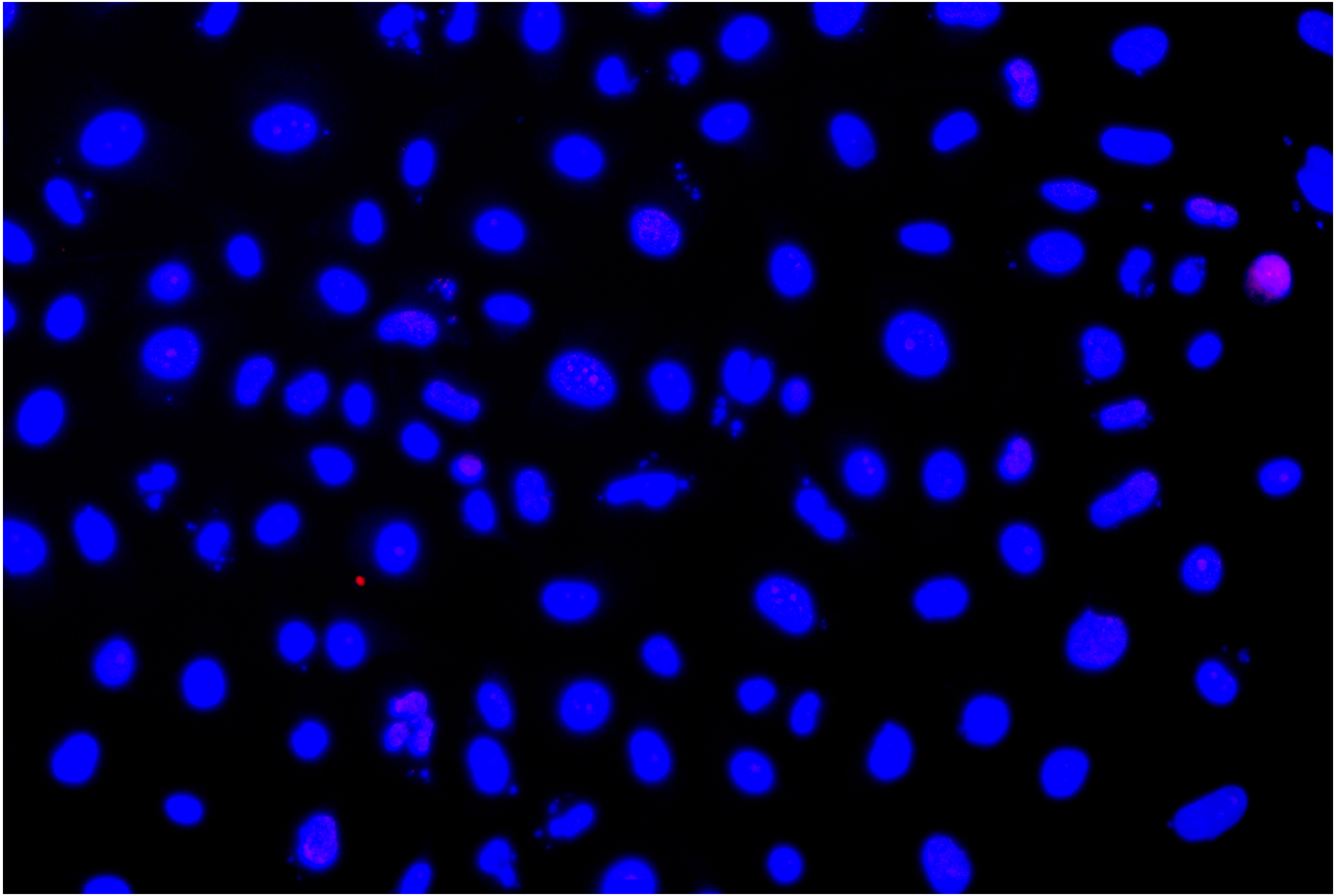

HCCLM3 Si-HMGA1+anti-miR-NC Merge

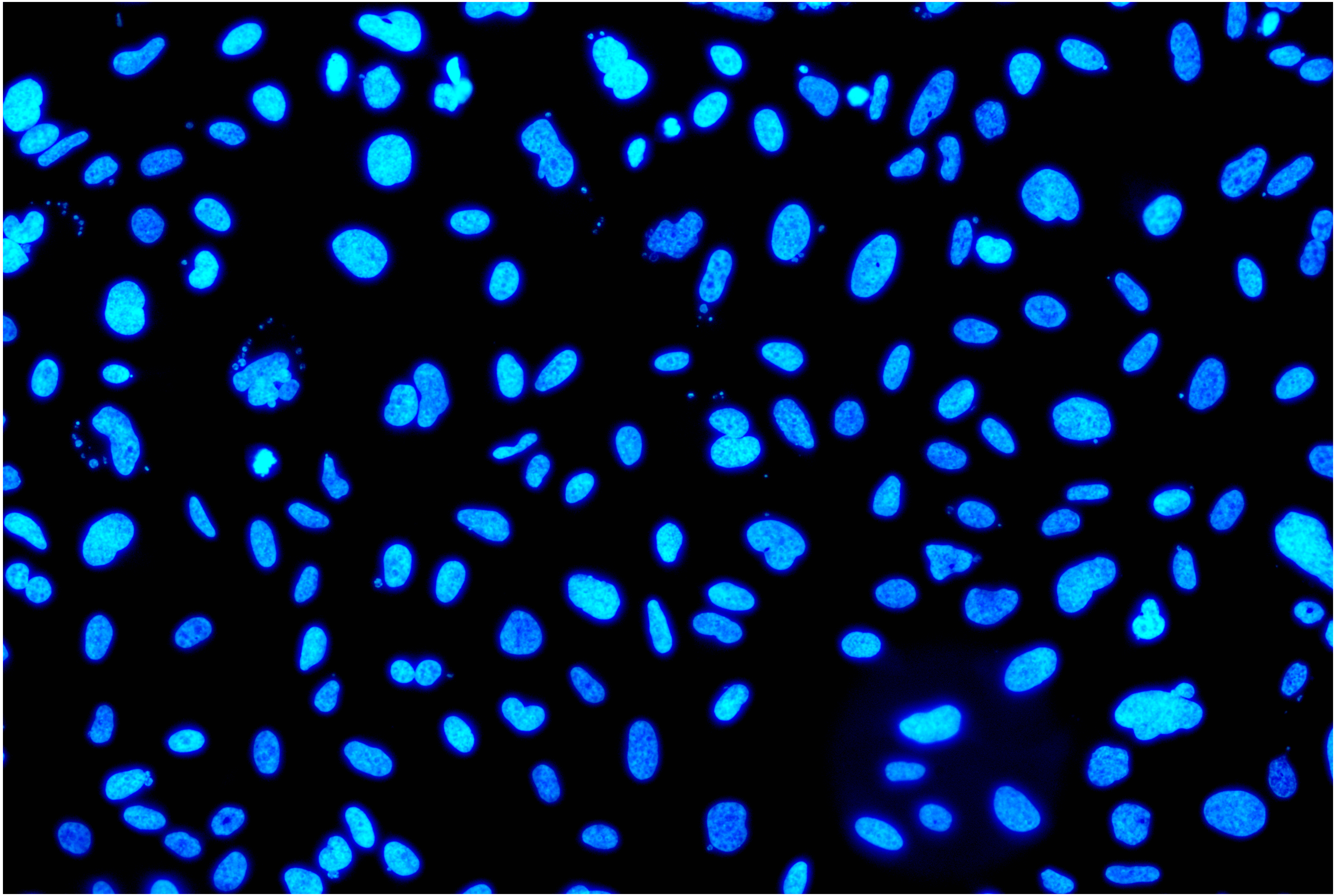

HCCLM3 Si-NC+anti-miR-NC DAPI

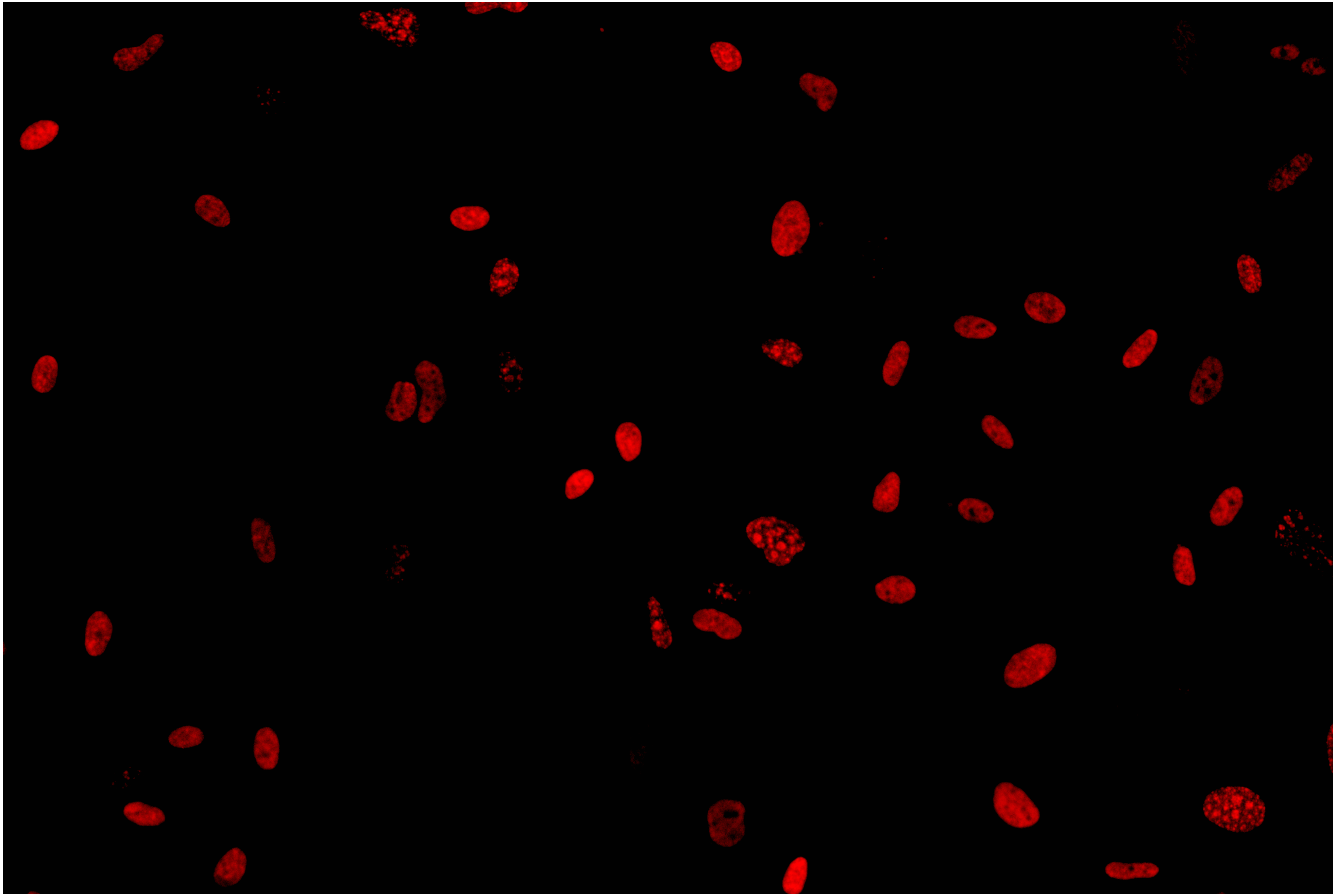

HCCLM3 Si-NC+anti-miR-NC EDU

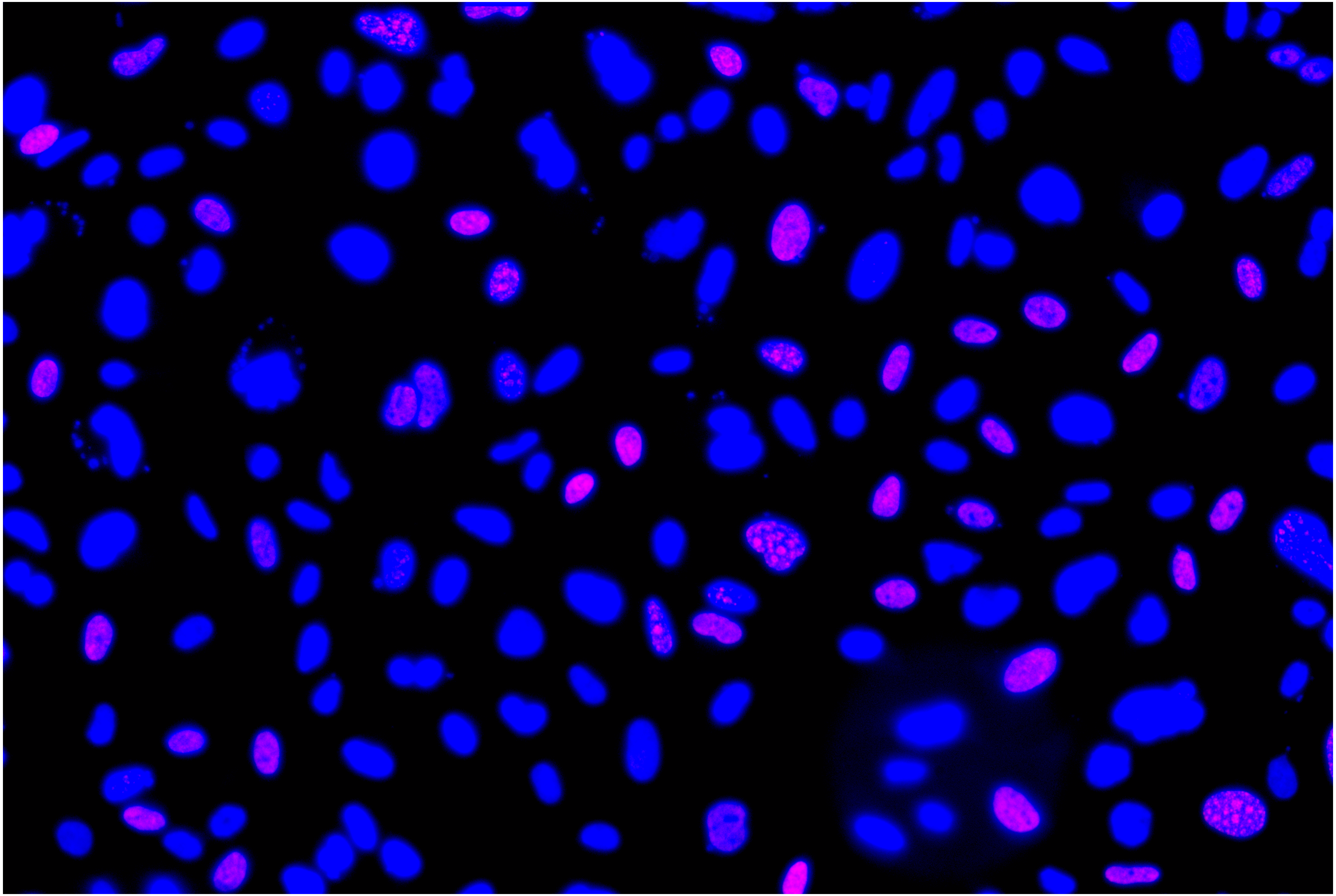

HCCLM3 Si-NC+anti-miR-NC Merge

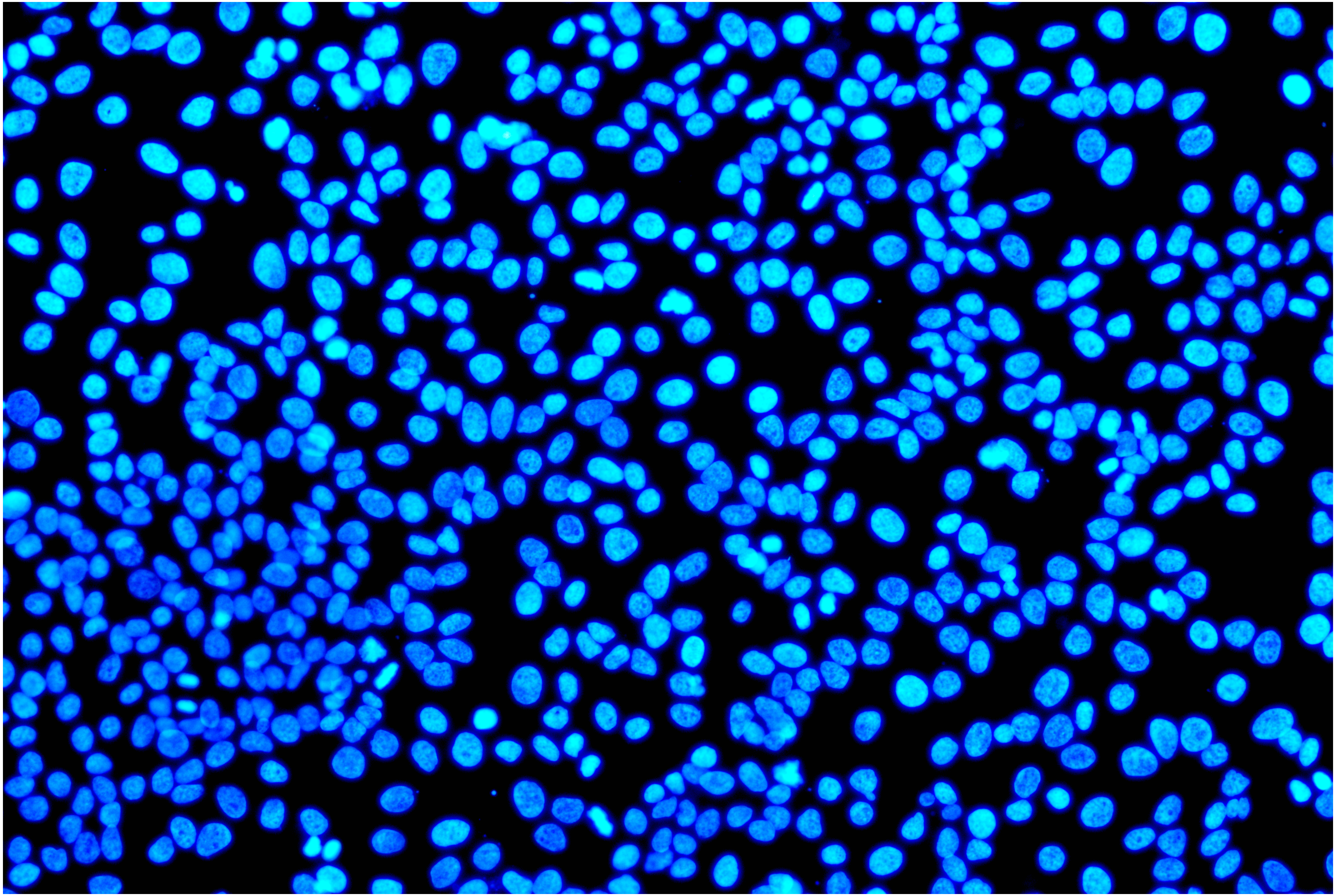

Huh7 anti-miR+si-NC DAPI

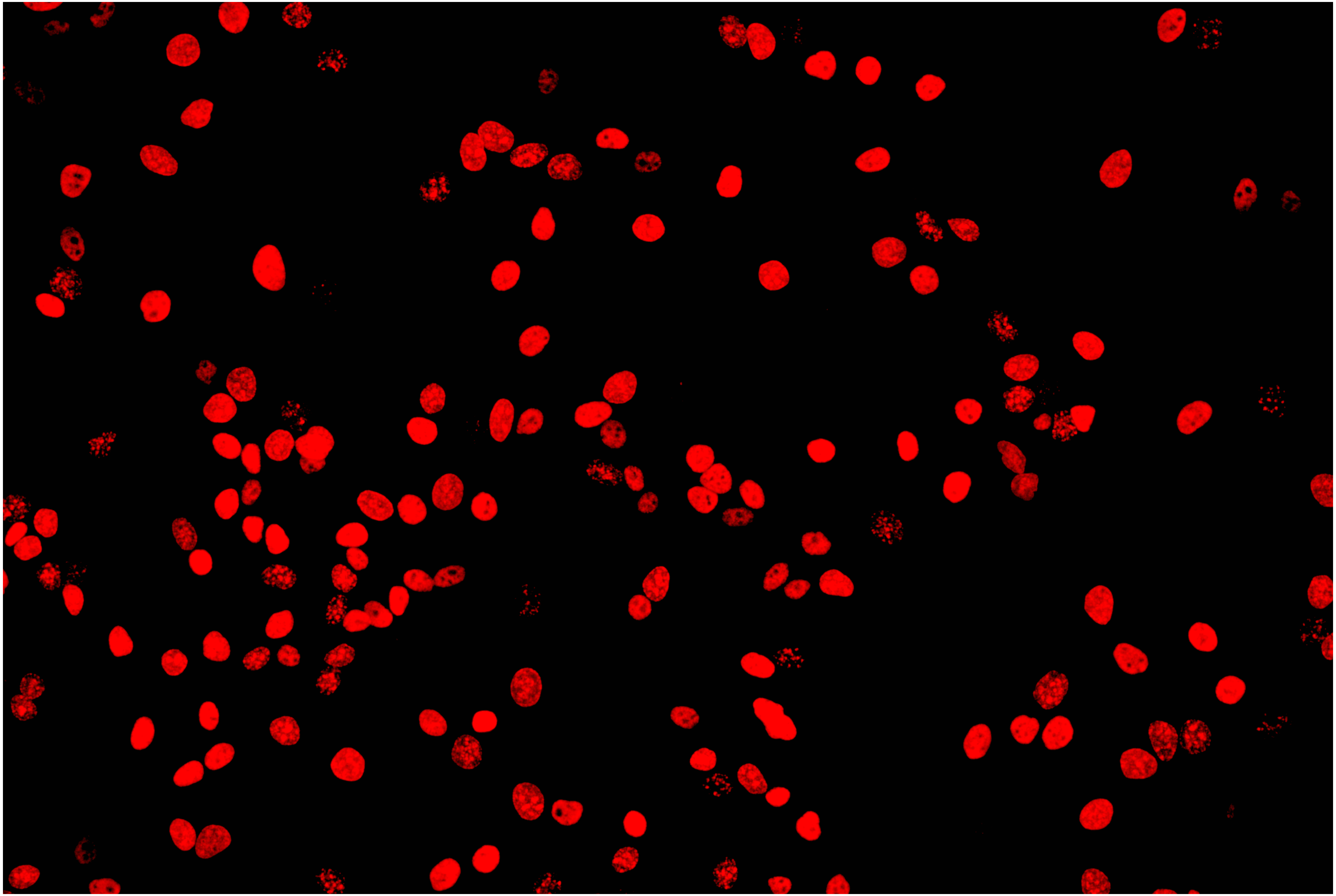

Huh7 anti-miR+si-NC EDU

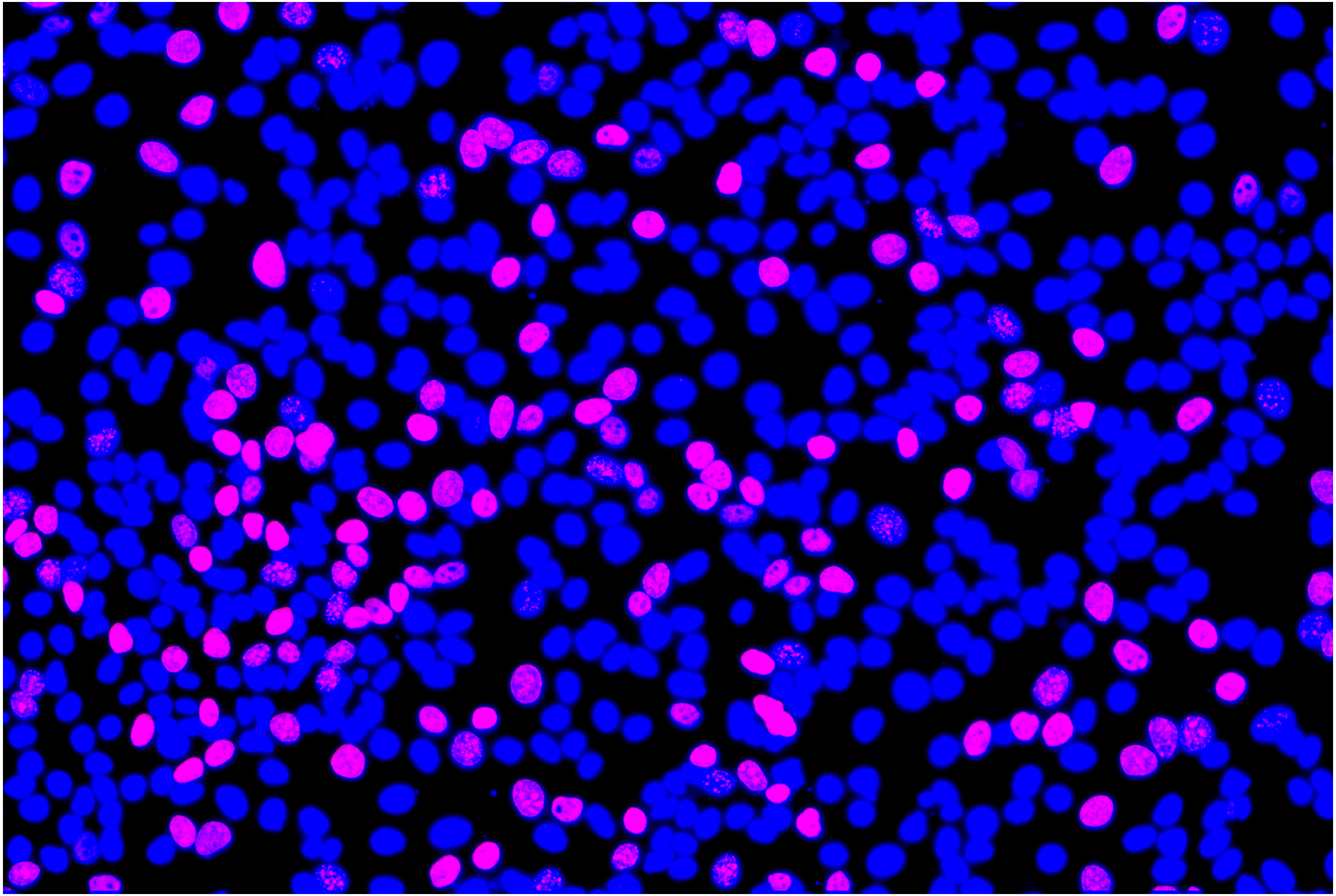

Huh7 anti-miR+si-NC Merge

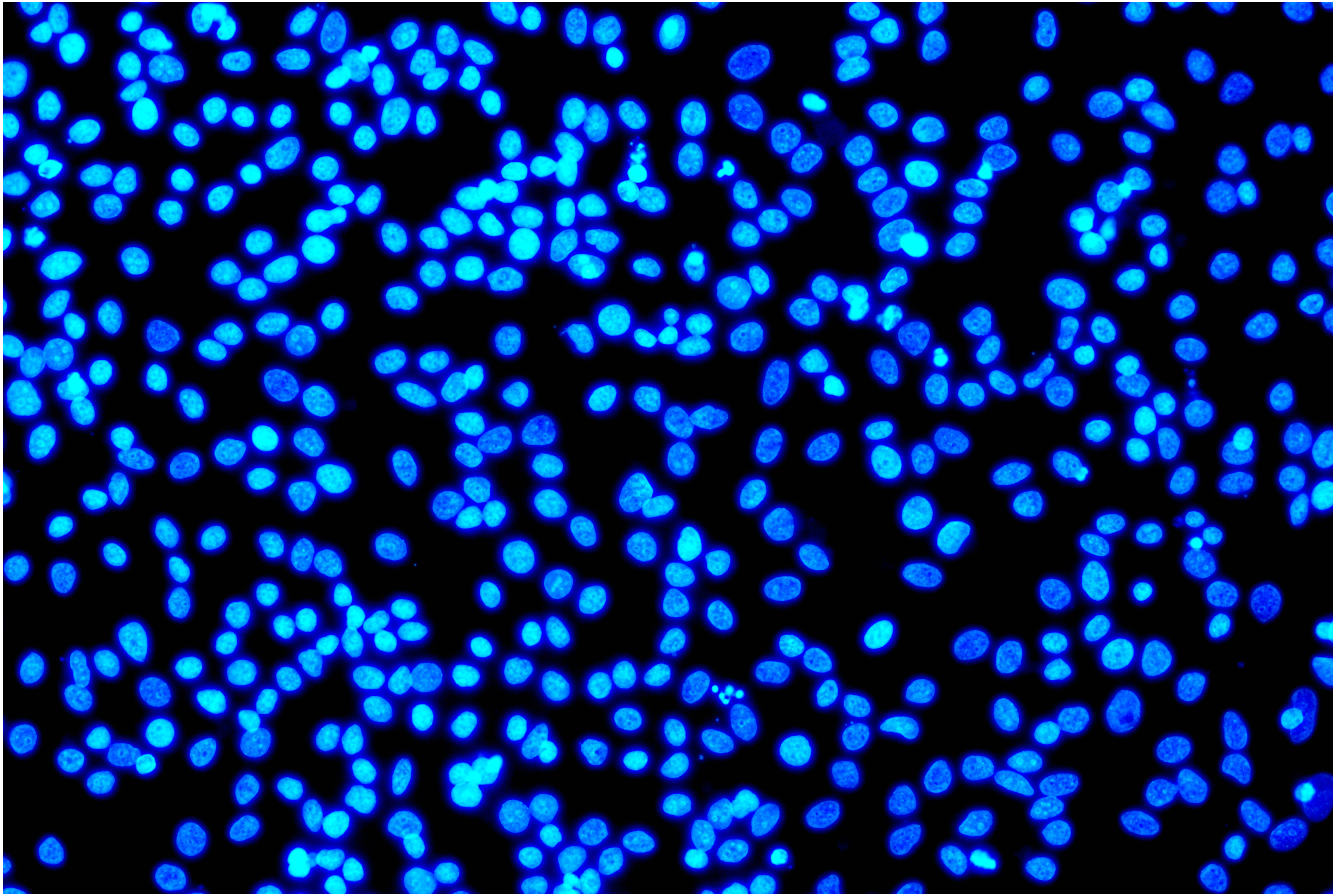

Huh7 blank DAPI

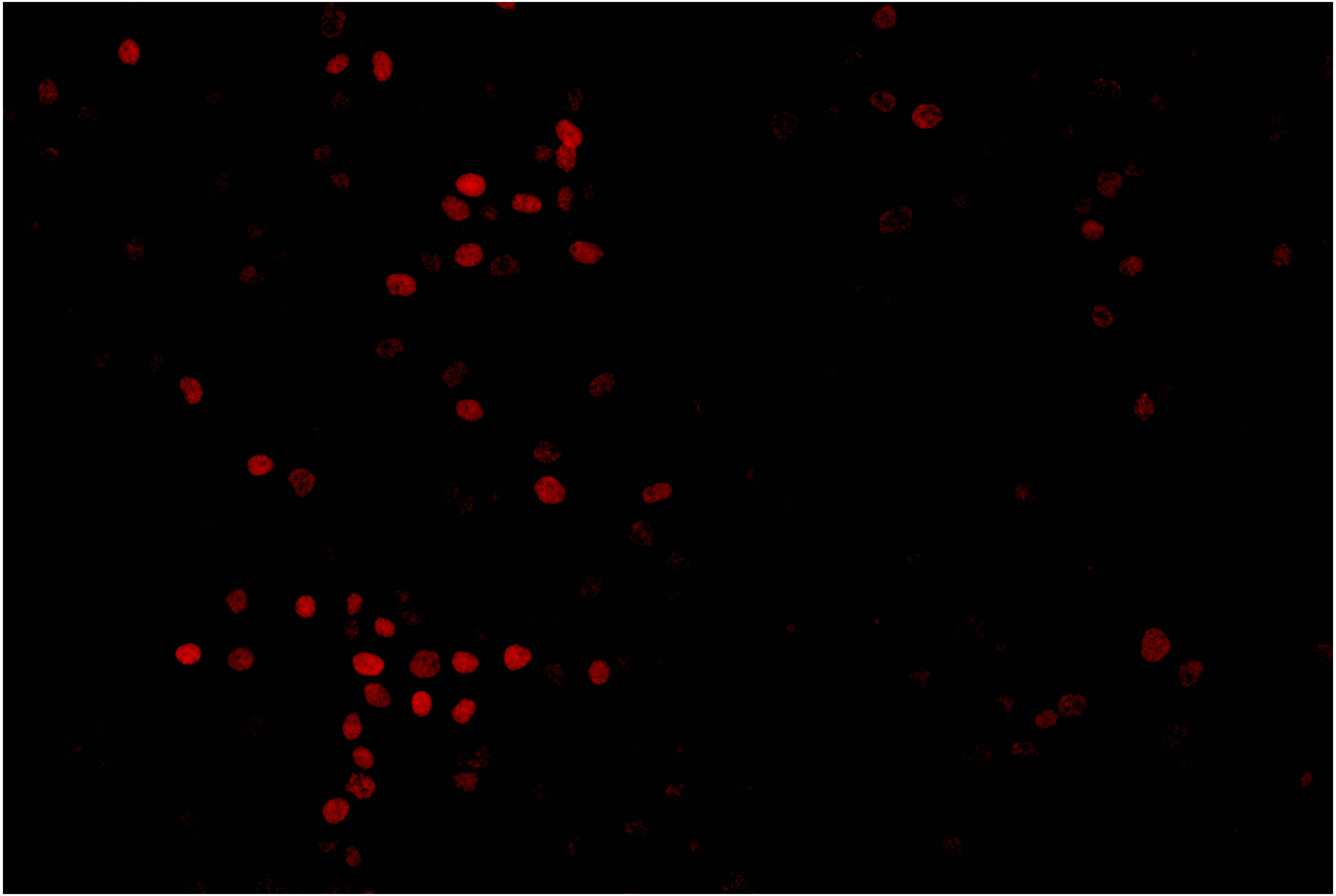

Huh7 blank EDU

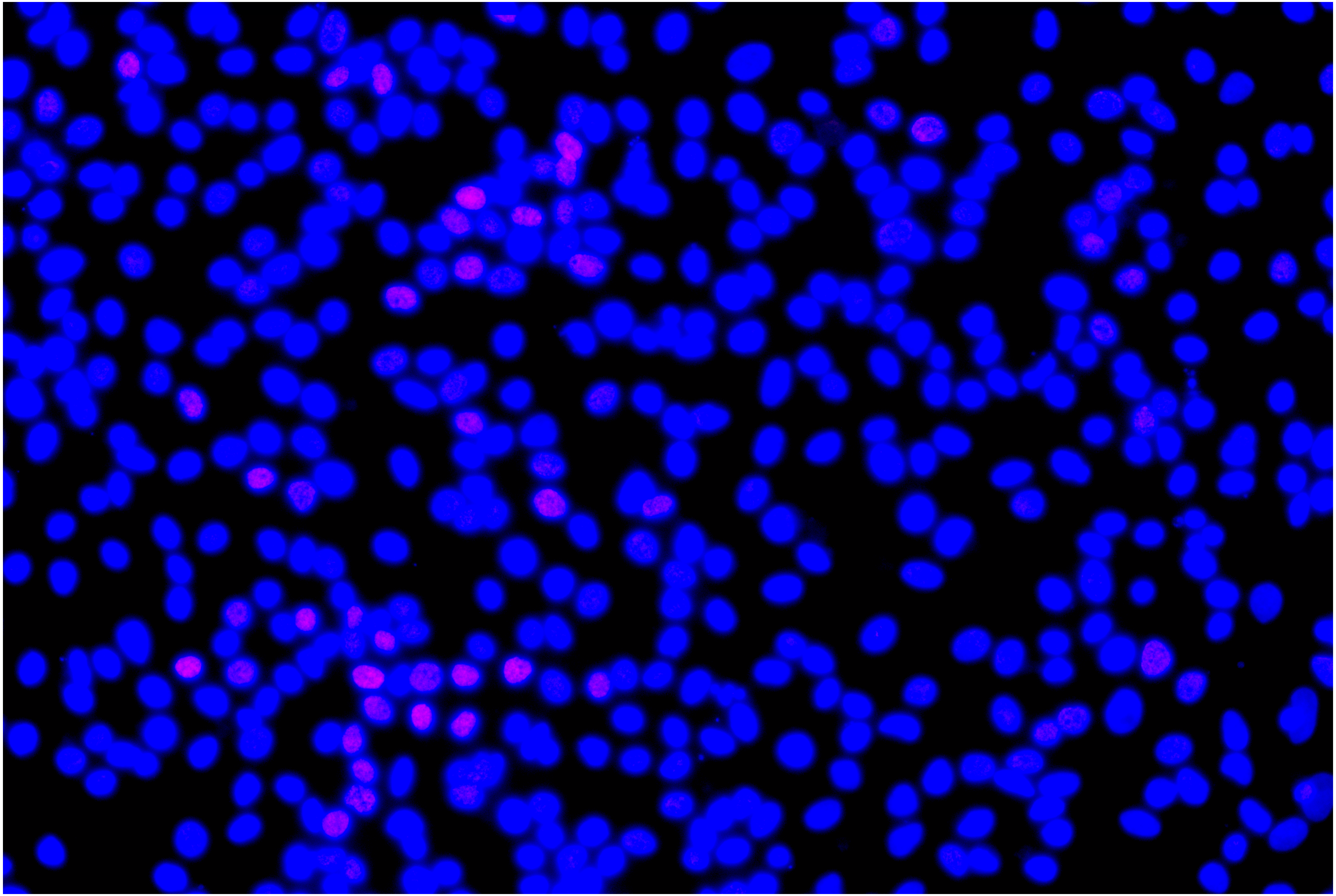

Huh7 blank Merge

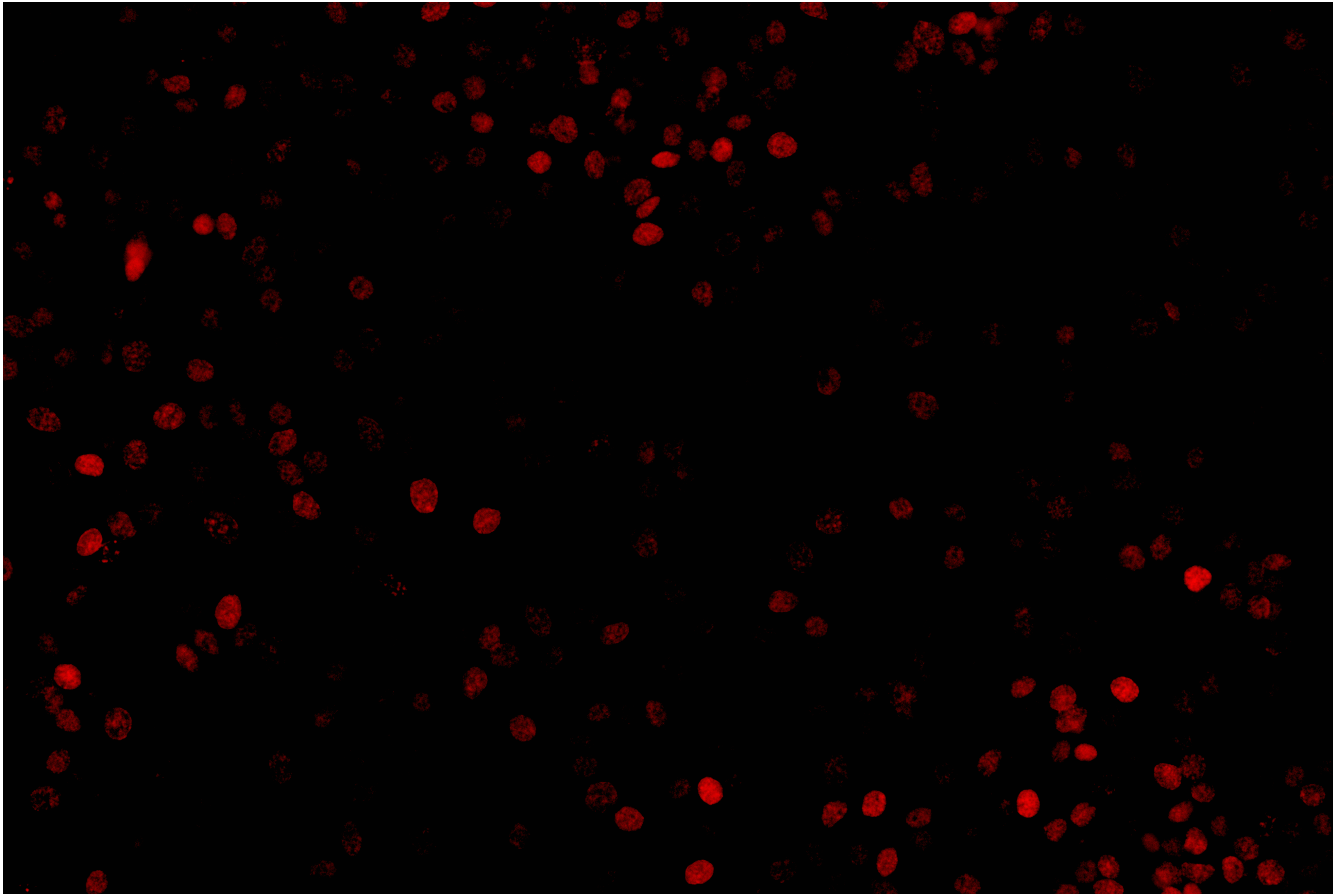

Huh7 Si-HMGA1++anti-miR EDU

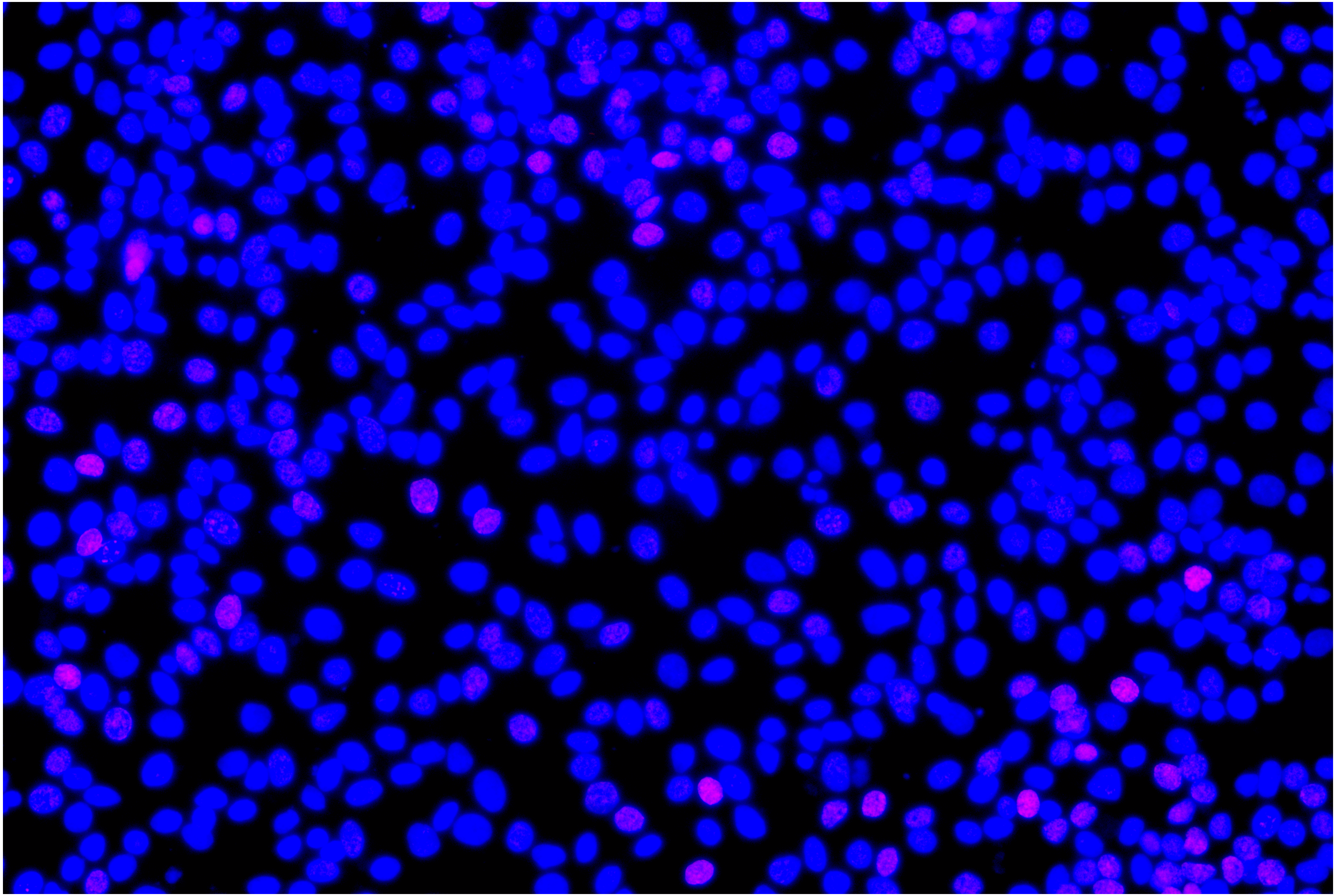

Huh7 Si-HMGA1++anti-miR Merge

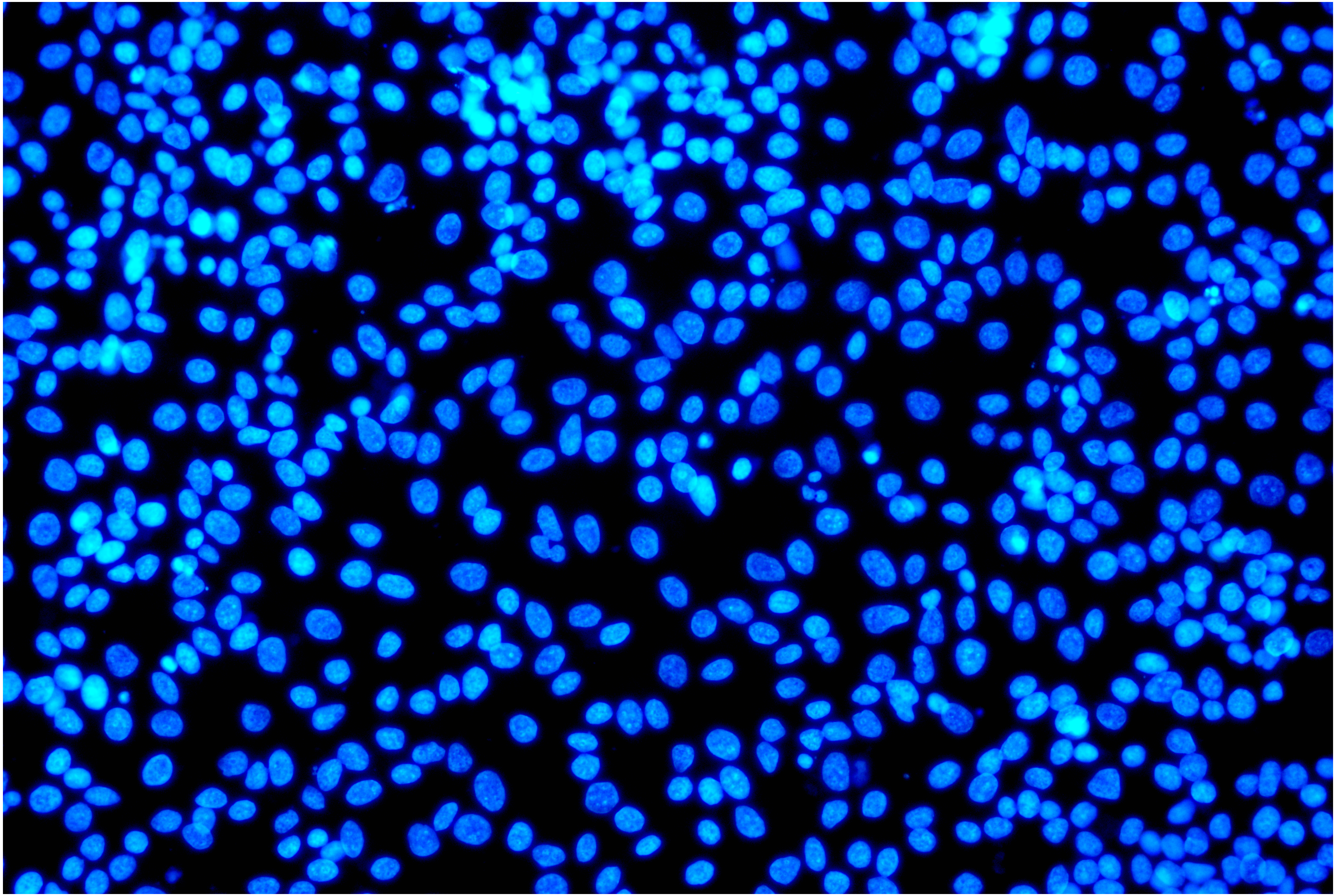

Huh7 Si-HMGA1+anti-miR DAPI

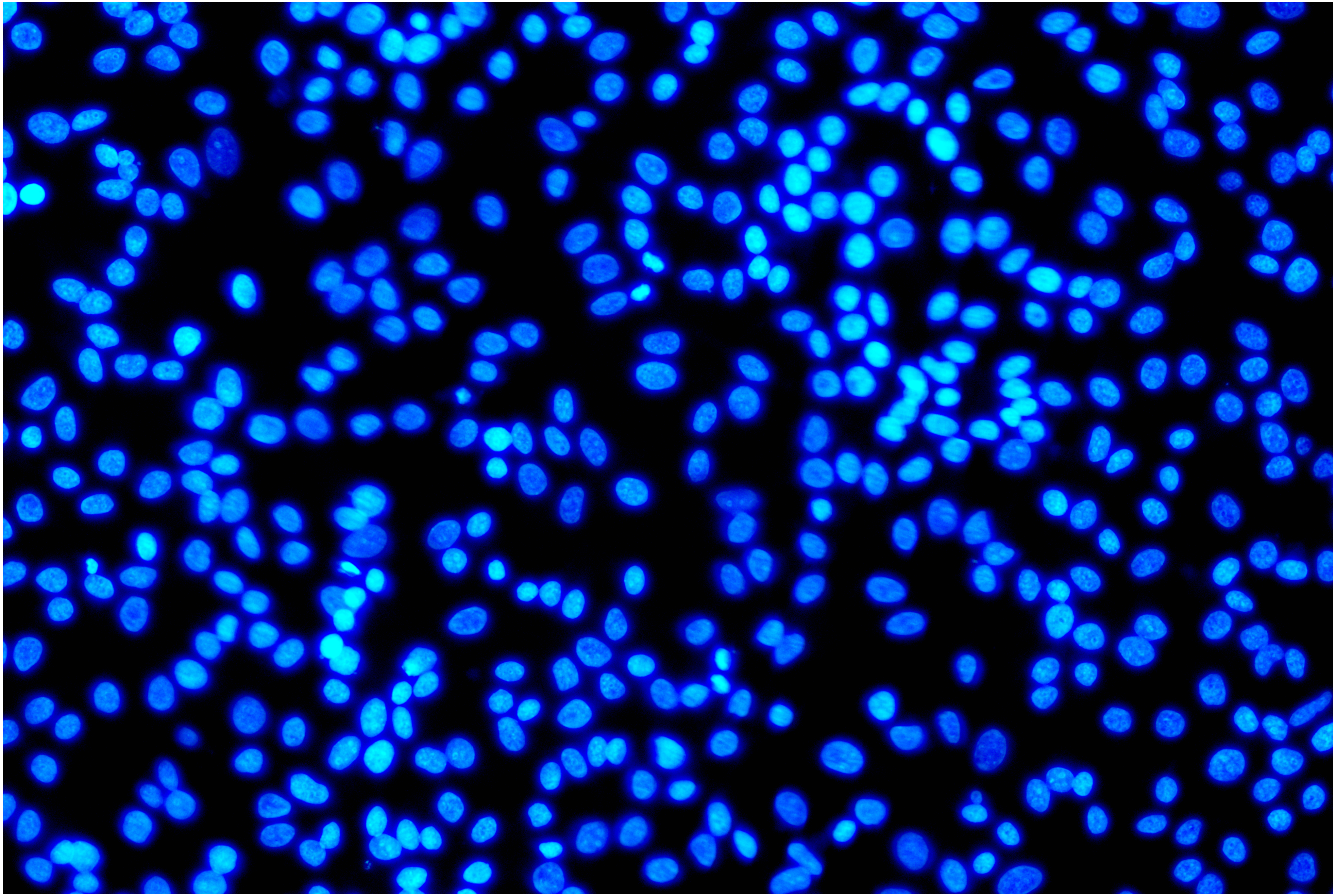

Huh7 Si-HMGA1+anti-miR-NC DAPI

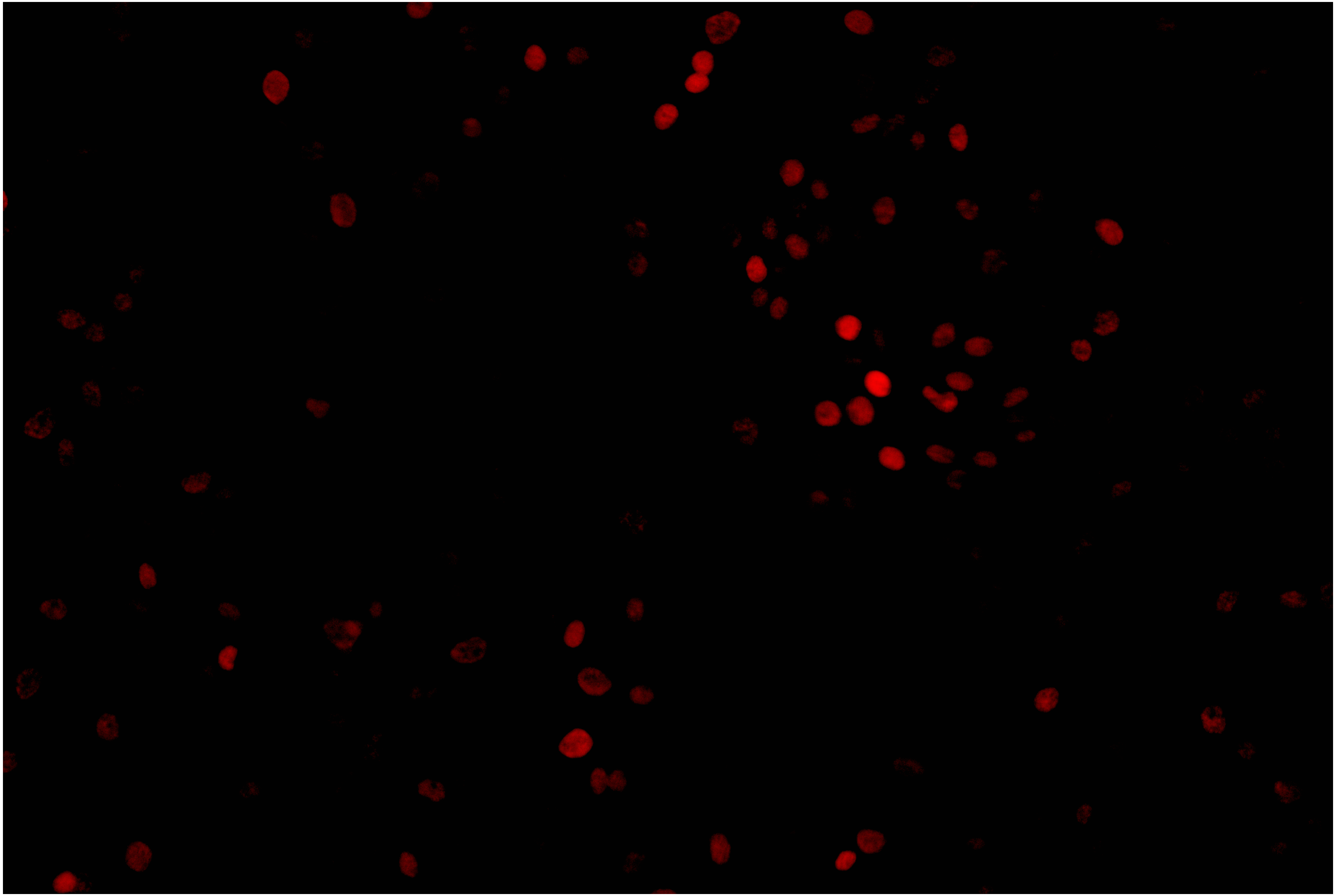

Huh7 Si-HMGA1+anti-miR-NC EDU

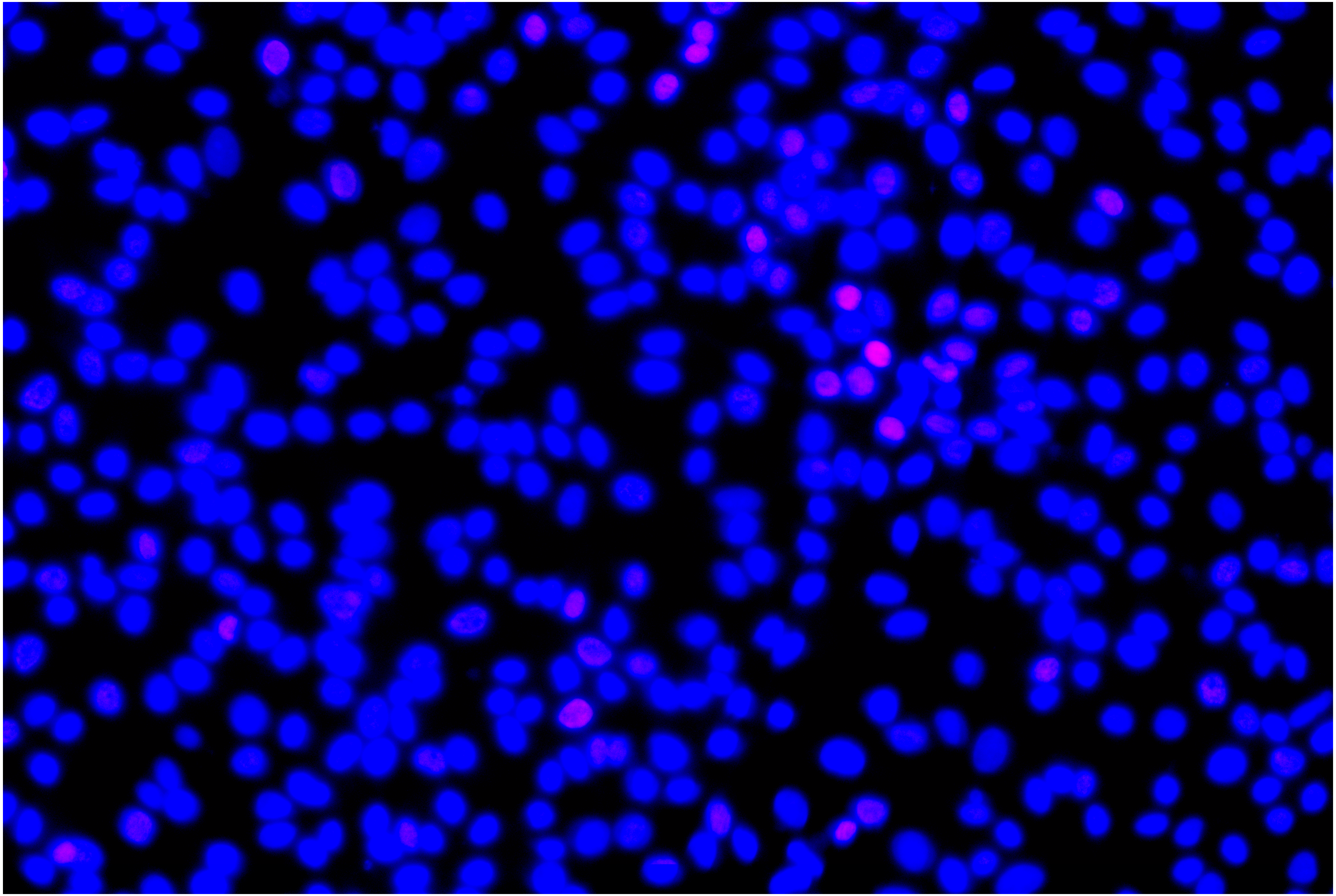

Huh7 Si-HMGA1+anti-miR-NC Merge

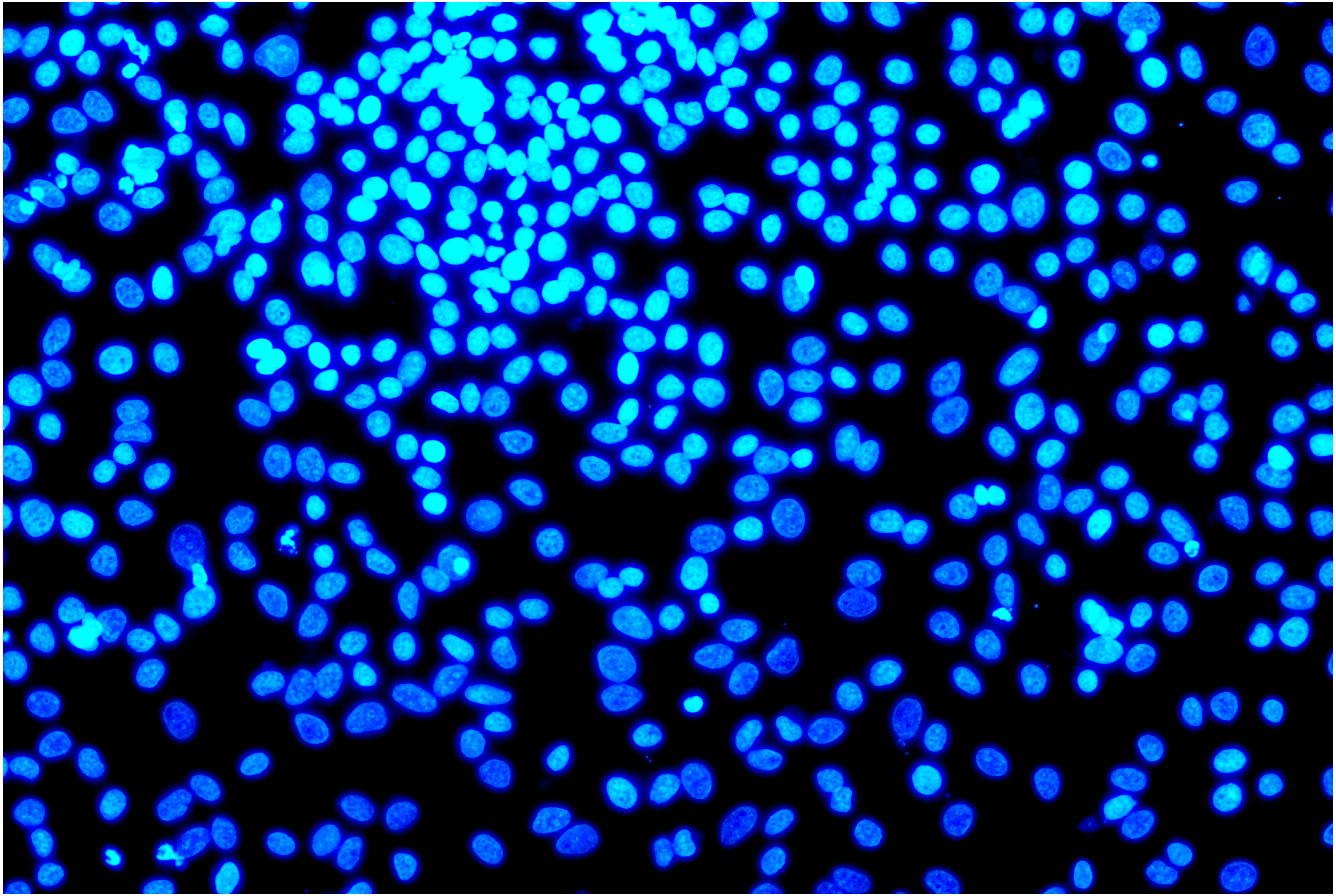

Huh7 Si-NC+anti-miR-NC DAPI

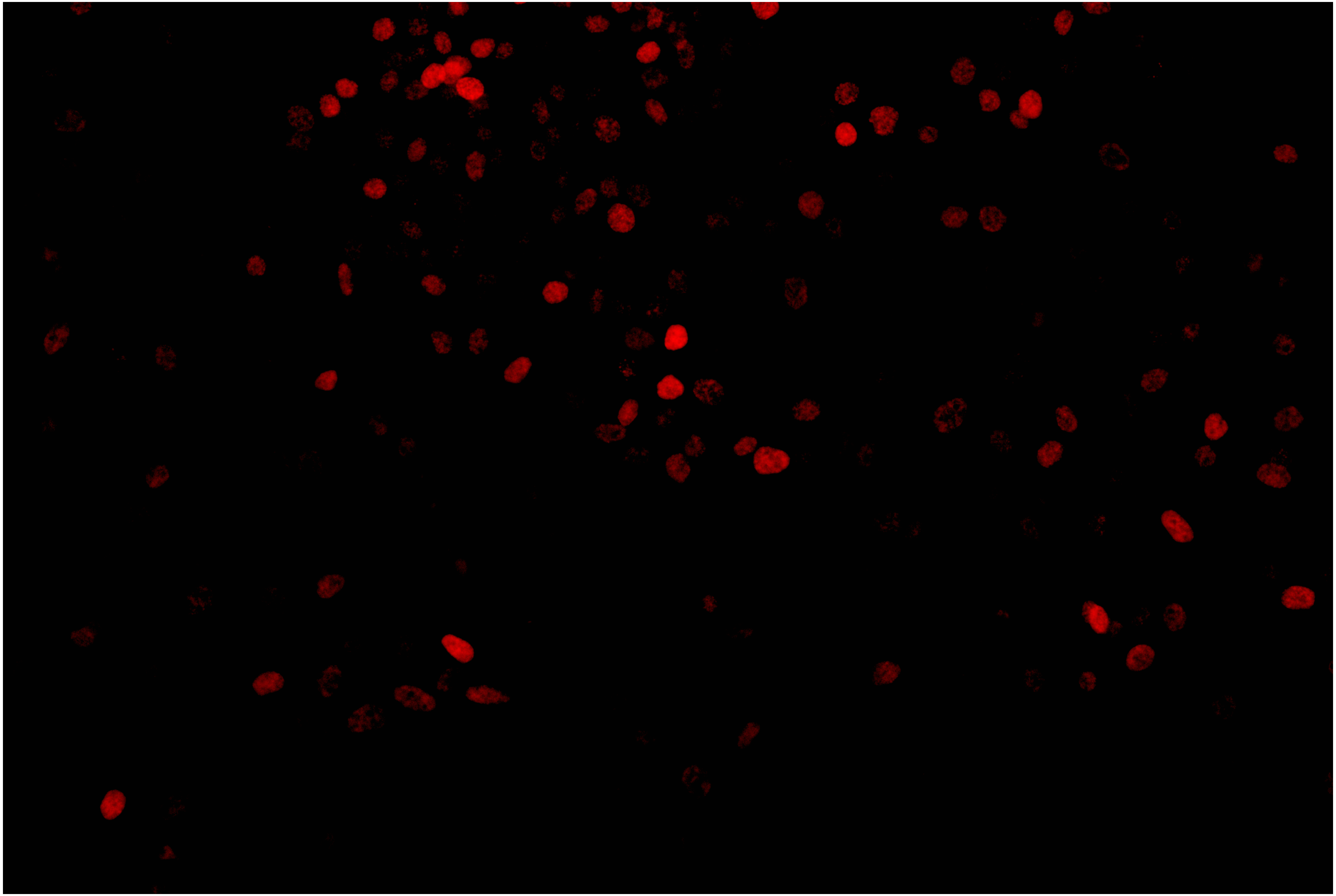

Huh7 Si-NC+anti-miR-NC EDU

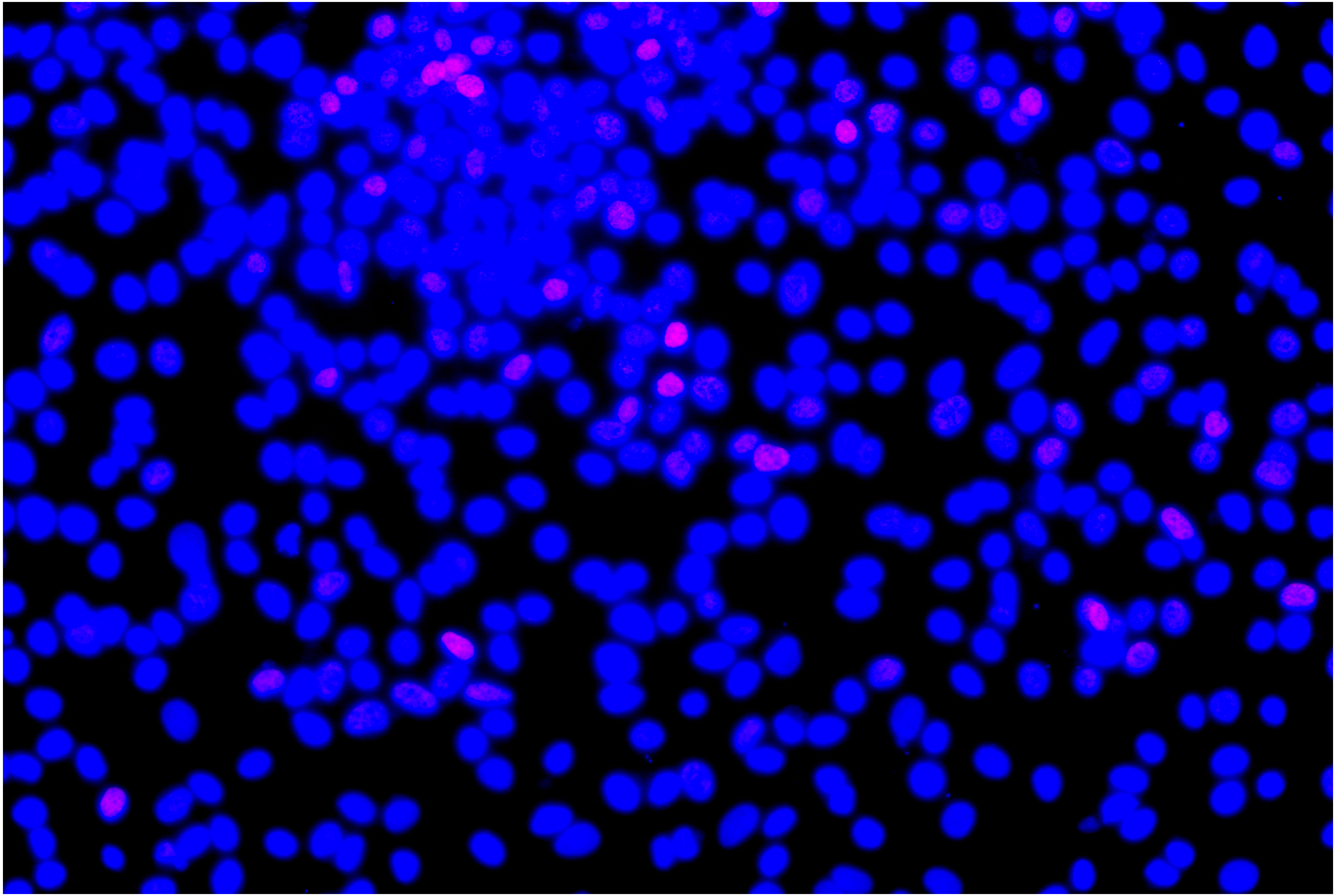

Huh7 Si-NC+anti-miR-NC Merge
